# Archaic ancestry inference in imputed ancient human genomes

**DOI:** 10.1038/s41467-026-76204-0

**Published:** 2026-08-06

**Authors:** Marco Rosario Capodiferro, Léo Planche, Emily M. Breslin, Linda Ongaro, María C. Ávila-Arcos, Flora Jay, Lara M. Cassidy, Emilia Huerta-Sanchez

**Affiliations:** 1 Smurfit Institute of Genetics, Trinity College Dublin, Dublin 2, Ireland; 2 Interdisciplinary Laboratory of Digital Sciences, Université Paris-Saclay, CNRS, INRIA, Orsay, France; 3 International Laboratory for Human Genome Research, Universidad Nacional Autónoma de México, Querétaro, México; 4 Ecology and Evolutionary Biology and Center for Computational and Molecular Biology, Brown University, Providence, RI USA

**Keywords:** Genome evolution, Evolutionary genetics, Molecular evolution, Evolutionary biology

## Abstract

When modern humans expanded from Africa into Eurasia, they interbred with archaic hominins such as Neanderthals and Denisovans. This introgression shaped human evolution, yet most insights have been gained from present-day genomes, leaving little known about how archaic variants evolved after interbreeding. Ancient genomes offer a direct view of this process, but low coverage and poor quality have limited their use. Recent advances in genotype imputation offer a way to overcome these challenges by reconstructing missing information from reference panels and recovering evolutionary signals from low-coverage data. Here, we show that imputation enables accurate detection and quantification of archaic introgression in ancient genomes, improves local archaic ancestry inference, and that regions of archaic ancestry are imputed with especially high accuracy. We further demonstrate that imputed genomes can reconstruct the trajectories of introgressed haplotypes, distinguish populations across time and geography, and identify both known and additional candidates for adaptive introgression.

## Abstract (web summary)

Genotype imputation enables accurate detection of Neanderthal and Denisovan ancestry in low-coverage ancient genomes and allows the identification of introgressed haplotypes as well as candidate genes of adaptive introgression across time and space.

## Introduction

Despite recent advances in identifying and quantifying archaic introgression in contemporary humans^1^, its inference in ancient genomes has been relatively neglected due to the poor quality of ancient DNA (aDNA)^2^. Analysis of ancient genomes could provide valuable insights into the number, magnitude and timing of introgression events, and the post-introgression (~40,000 years ago) evolution of archaic variants within modern human genomes, including the identification of adaptive introgression signals.

This deficiency is mainly attributed to low coverage and sequencing errors leading to large gaps in genomic coverage^2^. Consequently, many studies on ancient genomes resort to pseudohaploidization (i.e., selecting a single random allele or read at each site) rather than using genotypes. These limitations often render approaches developed for analyzing contemporary genomes unsuitable for aDNA. While archaic Global Ancestry Inference may still be suitable for ancient genomes, Local archaic Ancestry Inference (LAI) necessitates high-quality genotype data and is highly susceptible to missing data. Indeed, the only software specifically developed for this task, Admixfrog^3^, provides a useful option for working with aDNA. However, depending on the coverage of the ancient genomes, the amount of archaic segments may be underestimated^4^.

A promising solution is genotype imputation, which allows for the inference of missing or incorrect genotypes—caused by sequencing at low depth or damage in aDNA— by using a reference panel of present-day haplotypes, facilitating haplotype-based inference and increasing the depth of analysis^5–8^. The quality of the inferred genotypes is influenced not only by the coverage of ancient genomes, but also by the frequency of alleles in the reference dataset^9^. Given the small amounts of introgressed archaic segments in modern humans (0–6%)^10,11^, it is unknown whether imputation is particularly challenging in archaic segments, and whether introgressed regions can be reliably inferred in imputed ancient genomes.

**Fig. 1:**
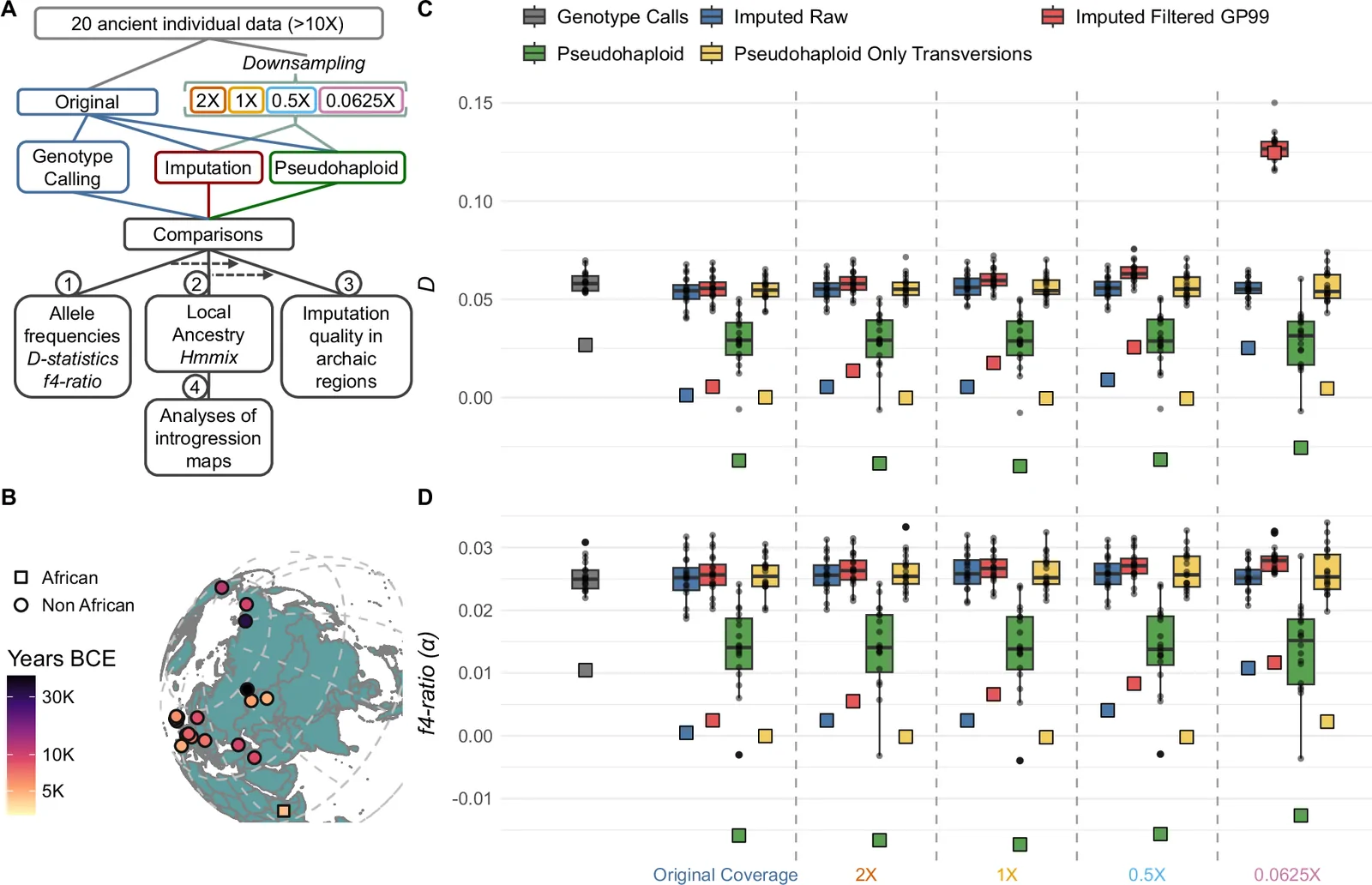
Overview of the analytical workflow and summary of allele frequency analyses. **A** Workflow illustrating the methods used to test the inference of archaic introgression in imputed ancient DNA. **B** Map showing the geographical locations of the individuals included in this study. The colors represent the estimated date of each individual in thousands of years before present (BCE). **C** Boxplot of D-statistics for the 20 individuals calculated using AdmixTools in the format D(Africa, Ancient Individual; Altai Neanderthal, Chimp), plotted against the sequencing depth before imputation (*x*-axis). **D** Boxplot of α values for the 20 individuals obtained from the f4-ratio in the format f4(Altai, Chimp; Ancient Individual, Africa) / f4(Altai, Chimp; Vindija, Africa), also plotted against sequencing depth before imputation. For panels (**C**) and (**D**), *n* = 20 biologically independent individuals per coverage level and data type. Box plots show median (center line), 25th and 75th percentiles (box bounds), and whiskers extending to 1.5X the interquartile range; individual data points beyond whiskers are shown as outliers. Each coverage level includes four boxplots, which depict the distribution of the respective statistics measured at the individual level, representing different data types. Two are based on imputed data: Blue, without quality filtering; Red, filtered for genotype probability (GP ≥ 99%). The other two reflect results from pseudohaploid data, where one random allele is selected per position: Green, without filtering; Yellow, retaining only transversions, to reduce the impact of post-mortem damage. The dots represent individuals, while the highlighted squares indicate the African individual Mota, used as a negative control.

Here, the main goal of this study is to investigate the feasibility of inferring archaic introgression in imputed ancient genomes and show that introgression estimates from imputed low-coverage ancient genomes are similar to those obtained from high-coverage ancient genomes. Our strategy is based on a comparative approach (Fig. 1A), in which we evaluate the results of specific analyzes across six different versions of previously published high-coverage shotgun-sequenced genomes. These include the original high-coverage data (Original; high-coverage considered >10X in aDNA studies), the imputed version of the same original high-coverage genome (Imputed OC), and imputed versions obtained after downsampling to various low and very low-coverage levels (2X, 1X, 0.5X and 0.0625X). We selected 20 high-coverage genomes representing ancient individuals from Eurasia, the Americas, and Africa, spanning a broad temporal range from approximately 44,000 to 5000 years Before Common Era (BCE) (Supplementary Data 1, and Fig. 1B)^5,12–24^. Imputation was performed using GLIMPSE^6^ with the 1000 Genomes Project dataset as the modern reference panel^25^.

As shown in Fig. 1A, our workflow allows us to examine three key aspects: the reliability of identifying archaic introgression, the ability to quantify the amount of introgression, and the precision in pinpointing the genomic locations of introgressed haplotypes in imputed ancient genomes. We first present results on the allele frequency analyses (D-statistics and f4-ratio). Then, we evaluate the local archaic ancestry inference (LAI). We also evaluate imputation performance in both archaic and non-archaic regions/variants. Finally, we illustrate applications enabled by these outputs. We find that imputation accuracy is higher in archaic introgressed regions compared to non-introgressed regions, and that metrics of introgression from imputed low-coverage ancient genomes are accurate. Notably, even in scenarios where high-quality ancient genomes are available, imputation offers a valuable avenue for inferring missing variants, refining and expanding our understanding of archaic introgression in ancient genomes. Our results provide evidence that it is possible to leverage the large and growing dataset of imputed ancient genomes to investigate introgressed archaic segments across time.

## Results

### Using allele sharing to identify and quantify archaic introgression

To test the ability to detect allele sharing between archaic and ancient (imputed and original) genomes (see “Methods”), we employed D-statistics in the form D(Africa, Test Individual; Archaic, Outgroup)^26^. Our results show comparable outcomes (*D* = 0.05–0.06) among the imputed genomes and the original ancient genome (i.e., “the truth”, Fig. 1C, and Supplementary Fig. 2). The significance of the signals, as indicated by the *Z*-score in Admixtools, further supports the results (Supplementary Fig. 1). The pseudohaploid data show an underestimation of the *D* value when all positions are considered, however, when only transversions are used, it is very consistent with the imputed and original data (Fig. 1C).

As in previous studies^9^, we applied filters for genotype probability (GP ≥ 99%) and minor allele frequencies (MAF) in the reference panel (MAF ≥ 5%), which are expected to yield the most reliable calls. While overall consistency is maintained down to 0.5X coverage, applying the GP filter at 0.0625X coverage causes the D-statistic to become unreliable, leading to an increase in the *D* values (Fig. 1C). In contrast, applying only the MAF filter does not produce this bias (Supplementary Fig. 1). One way to correct this is to weight the frequency of each allele by its GP values rather than being represented as a discrete frequency of 0, 0.5, or 1 (Supplementary Information). Supplementary Fig. 3 shows that this approach brings complete consistency across coverages to the values of the D-statistic in imputed genomes, even as low as 0.0625X, and better matches the results obtained from original genomes.

In the overall analysis, the African individual Mota (Ethiopia, 4470 BCE)—used as a negative control—shows some unexpected signals when only a reduced number of SNPs can be explored, such as positive Ds and significant *Z*-scores (see Supplementary Information). This could reflect either traces of archaic ancestry from back migrations or the well-known limitations in imputation accuracy for African genomes^9^.

To assess imputation’s ability to estimate the overall proportion of archaic introgression in ancient genomes, we applied the f4-ratio approach^27^ in the form α = f4(Archaic1, Chimp; Test, Africa)/f4(Archaic1, Chimp; Archaic2, Africa)^26^.

We observed consistent results across different coverages, with values ranging between 2% and 3%, comparable to those obtained from original genomes (Fig. 1D). Applying a GP ≥ 99% filter revealed a slight increase in archaic ancestry, which becomes more pronounced as sequencing depth decreases. However, the bias is small and we observe similar proportion results across individuals (Supplementary Fig. 4). The only exceptions to the overall consistency of the f4-ratio results arise with Mota and pseudohaploid data when using all sites. Mota shows a slight overestimation of archaic ancestry in both original and very low-coverage imputed genomes (0.0625X), while pseudohaploid data can even produce negative *α* values and more consistent estimates are obtained when restricting the analysis to transversions.

To test whether GAI is influenced by individual age or original coverage, we assessed their correlation with *α* in non-African individuals (excluding Mota) (Supplementary Fig. 5). As expected, *α* correlates with age but not with original coverage in any imputed dataset.

### Local archaic ancestry inference

**Fig. 2:**
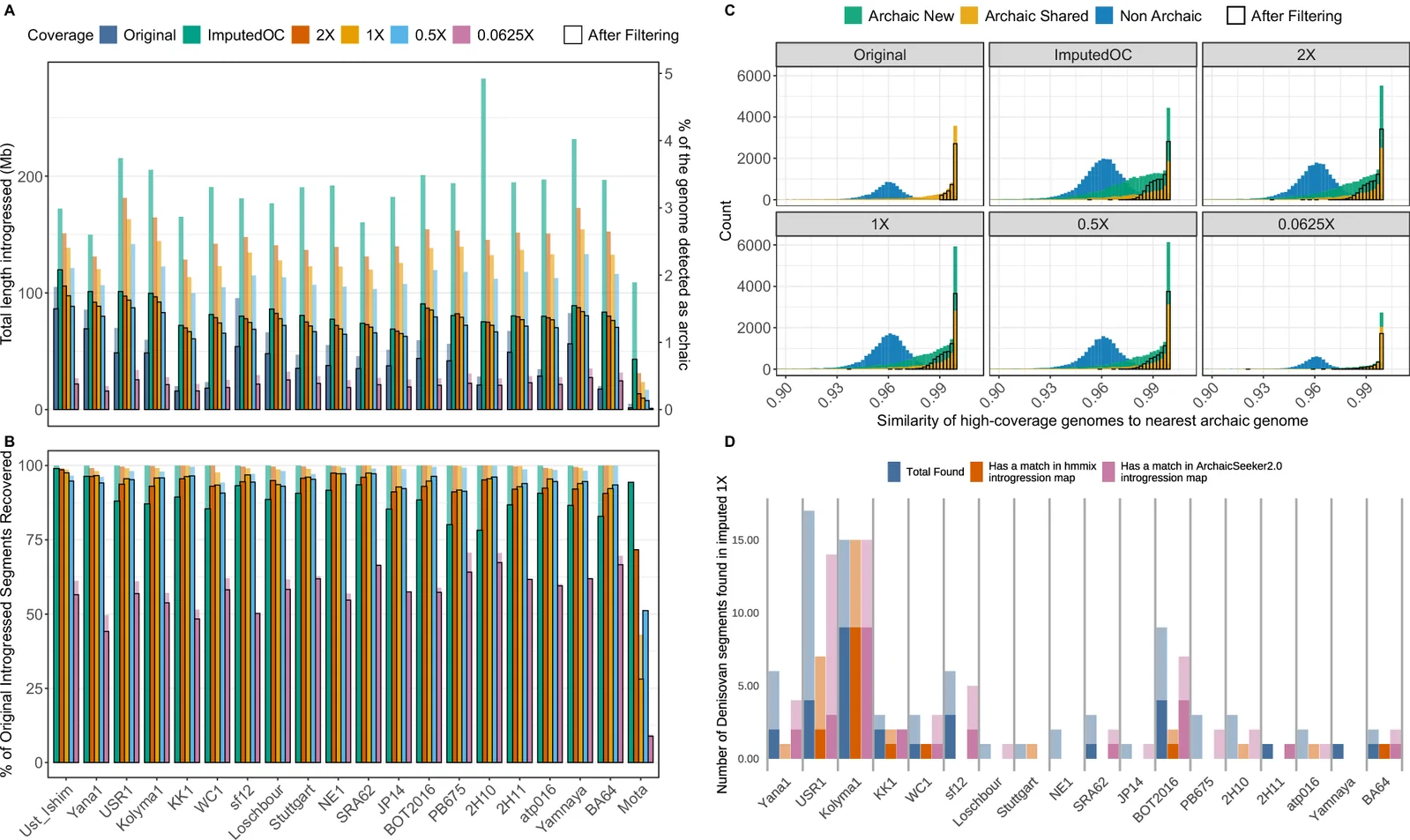
Local archaic ancestry inference. **A** Sum of the length of the segments identified in each individual for each of the dataset expressed in megabases (Mb) and also expressed on the right axis as the percentage of the genome detected as archaic. **B** Proportions of the archaic segments detected using the original genome that are also detected using imputed dataset. Over all the individuals, respectively 100%, 99%, 99%, 97% and 50% of the segments detected using the original genome are also detected using respectively the Imputed OC, 2X, 1X, 0.5X and 0.0625X genomes. The bordered bars for A and B represent the respective measures after filtering for quality, considering a minimum length of 40Kb and a similarity measured with the the imputed data of 0.99. **C** Similarity to the nearest archaic genome (between Vindija Neanderthal and Denisovan) of the segments detected in Imputed OC, 2X, 1X, 0.5X and 0.0625X. The similarity is computed by looking at the corresponding individual genotype in the original genome; this step is to avoid taking into account archaic variants which would have been wrongly introduced with imputation. In green, the segments that are only detected in the imputed genome (Archaic New), in orange the segments that are detected both in the imputed and original genome (Archaic Shared). The black boxes represent the proportion of archaic segments (Shared and New) after filtering. **D** Denisovan segments identified in the 1X dataset. In blue the total numbers of inferred Denisovan segments, in orange the number of such segments which matched a Denisovan segment found in hmmix^28^ introgression map and in pink the number of such segments which matched a Denisovan segment found in ArchaicSeeket2.0^31^ introgression map. The dark shade corresponds to the segments also found in the original genome, the light shade represents segments only found after imputation.

We evaluated the ability of imputation to improve the identification of archaic segments in ancient genomes. We used hmmix^28^ to detect introgressed segments because (i) it was developed for high-quality data and we want to show that such methods can be applied to imputed ancient genomes and (ii) it is reference-free, making it more suitable for detecting Denisovan ancestry^4^. We applied hmmix^28^ to identify archaic segments in all original and imputed (filtered for GP ≥ 99%) individual genomes. We observed that imputation significantly enhances the identification of introgressed segments in imputed aDNA. Both the number of introgressed regions and the percentage of the genome identified as introgressed are higher in imputed genomes than in the original data, except in cases of very low coverage (Fig. 2A, and Supplementary Fig. 6).

The imputed genomes exhibit less variation between individuals (except for Mota) in the detected amount of introgression compared to original genomes where it is proportional to the sequencing coverage of the genome (Supplementary Fig. 6). Remarkably, more than 97% of the archaic segments detected in original genomes are still identified when the data are downsampled to 0.5X and then imputed, and even at 0.0625X coverage, half of the archaic segments remain detectable (Fig. 2B).

We check whether imputation improves detection of introgressed segments in data generated with the 1240k capture technique. For the individual Stuttgart (Germany, 7168 BCE), we compared results from shotgun sequencing and 1240k capture datasets. At ≥2X coverage, imputation on 1240k capture data enhances detection of archaic introgression. Although the overall magnitude remains below that from shotgun sequencing, it recovers a substantial, slightly smaller, fraction of the segments present in the original dataset as identified in the imputed shotgun data (Supplementary Figs. 7 and 8).

### Quality and properties of inferred archaic segments

To confirm the introgressed segments identified are of archaic origin, we measured the genetic similarity of the original genome (not imputed) to the nearest archaic genome (Vindija Neanderthal or Denisova3) in genomic regions classified as introgressed in the imputed genomes (Fig. 2C). We categorized these archaic regions into two groups: Archaic Shared, which includes regions detected in both imputed and original genomes, and Archaic New, consisting of regions identified only in the imputed ones.

While the Archaic Shared segments exhibit the highest mean similarity to the archaic reference genome, the Archaic New segments also show significantly higher similarity compared to regions classified as non-archaic (Fig. 2C). This suggests that the original genomes do carry those introgressed segments, but they failed to be detected. In this case, it is important to consider that despite the confidence in the genotype calls from original genomes, sequencing errors, missing data, and regions of low coverage can still result in incorrect genotypes, which may impact the detection of introgressed segments. We further expanded this concept by introducing a distance measure, providing a more principled framework for classification, normalizing the similarity by dividing it by the average similarity of modern African individuals (Biaka from HGDP) to the archaic genomes in each segment. When evaluating segments using both similarity and distance, we observe a clear separation between the distributions of archaic and non-archaic segments (Supplementary Fig. 9).

Building on this, we applied additional stringent quality filters to exclude segments (potential false positives) that are too short (<40 kb^29^) and lack strong genetic affinity to archaic genomes (similarity <0.99). With these filters we observed that the proportion of introgressed segments identified in the imputed genomes, compared to the original, more closely aligns with the f4-ratio estimates obtained using original data (Supplementary Fig. 10). Moreover, considering only the filtered results (the bar with the black border in Fig. 2A), the two older individuals, Ust’Ishim (Russia, 44366 BCE) and Yana1 (Siberia, 31850 BCE), have a higher proportion of their genome inferred to be of archaic origin across all coverage levels. Among the more recent individuals, depending on the geographic location and ancestral composition, we see fluctuations in the archaic proportion. Kolyma1 (Siberia, 9775 BCE), USR1 (Alaska, 11425 BCE), as well as BOT2016 (Kazakhstan, 5396 BCE), all carrying Ancient East Asian (AEA) ancestry^17,24^, show a high archaic proportion, in line with continental expectations, as contemporary East Asians harbor more archaic ancestry than contemporary Europeans^4,30^. Overall, excluding Mota, we find between 1.1% (JP14, Ireland 5483 BCE) and 1.9% (Ust’Ishim) of archaic ancestry in Imputed OC individuals. For present-day Eurasians, this amount is estimated around 1.5-2.1%^11,28^ (Supplementary Fig. 12).

Moreover, we assessed the potential of imputation to improve the detection of Denisovan segments in ancient genomes, a task complicated by the reliance on a single high-coverage Denisovan reference and the complex history of Denisovan introgression^4,30^. To evaluate imputation performance, we applied conservative filters, classifying a segment as Denisovan if: (i) it was detected as archaic, (ii) had a length above 100 kb, (iii) had a similarity of at least 0.99 to one of the high coverage archaic genome, (iv) had a at least 10 private Denisovan variants and (v) had more Denisovan private variants than Neanderthal private variants.

We observed that Kolyma1 and USR1 harbored the most Denisovan segments in both original and imputed data, reflecting their Ancient North Siberian (ANS) and East Asian ancestry^24^, while the ANS Yana1 showed fewer segments (Fig. 2D, and Supplementary Fig. 11). Comparison with introgression maps from contemporary individuals (hmmix^28^; ArchaicSeeker^31^) showed that Kolyma1’s Denisovan segments aligned with previous studies (Supplementary Fig. 13). Therefore, using imputed genotypes, we recovered most original segments (down to 0.5X coverage) and identified additional Denisovan segments (Fig. 2D, and Supplementary Fig. 11). For 0.0625X some Denisovan segments are still detected, but the total number does fall as expected.

Regarding the length of the detected regions, the introgressed segment length in original data correlates with individual age, as expected^30,32^, with Ust’Ishim and Yana1 encompassing the longer segments, but imputation reduces this signal, making ancient genomes distribution of segment length more similar to that of modern ones (see Supplementary Information, Supplementary Fig. 14). To further explore this limitation, we analyzed segment length based on (i) similarity to the archaic genome and (ii) whether segments were Archaic Shared or Archaic New. Shared segments display length distributions more similar to original data (Supplementary Fig. 15A) and longer than Archaic New. Nevertheless, length  only marginally affects similarity: distributions of similarity remain comparable between shared and new segments (Supplementary Fig. 15B).

### Quality of imputation in archaic vs not archaic genomic regions

In the previous sections, we have shown that we can detect and identify introgressed regions in low-coverage imputed genomes and estimate the proportion of introgression using *f-statistics*. Encouraged by this, we wanted to check whether introgressed regions and variants are easier or harder to impute and understand why we do not detect some introgressed segments in the original genomes.

We first assessed how well imputation recovers archaic variants. We defined a variant as archaic if it is absent in African populations (Supplementary Data S2) and homozygous in Vindija Neanderthal, and archaic SNPs are positions carrying these variants.

For each individual, we compared archaic SNPs between original and imputed genomes (Table 1). We observe that imputation infers archaic variants in introgressed regions as expected—both when the original genome has the archaic variant and when the position is missing- and assigns non-archaic variants in non-introgressed regions. For example, in both Original and Imputed OC genomes, archaic variants appear at a rate of 0.3% in non-archaic regions. For Archaic Shared regions, the rates are 88% and 89% for Original and Imputed OC, respectively, while for Archaic New regions, these values are 75% and 79%. Notably, the Archaic New segments contain more missing positions in the original data (Supplementary Table 1). This pattern confirms that the missingness of archaic variants in the original data could prevent hmmix from classifying these regions as introgressed. Finally, we measured the error of imputation in recalling archaic SNPs, which is remarkably low at approximately 0.2%, even at very low coverage such as 0.0625X.

**Table 1:** Rate of archaic variant imputation in imputed ancient individuals

Segment classification	Original -> imputed	Imputed OC	2X	1X	0.5X	0.0625X
Non Archaic	0 - > 0	44% (143,262)	44% (124,982)	45% (111,579)	45% (96,132)	42% (20,371)
Non Archaic	. -> 0	55% (179,722)	55% (155,854)	55% (137,760)	55% (118,485)	57% (27,554)
Non Archaic	0 - > 1	0% (0)	0.001% (3)	0.002% (5)	0.001% (2)	0% (0)
Non Archaic	1 - > 1	0.1% (432)	0.2% (431)	0.1% (318)	0.2% (325)	0.07% (38)
Non Archaic	. -> 1	0.2% (577)	0.2% (554)	0.2% (451)	0.2% (424)	0.1% (61)
Non Archaic	1 - > 0	0.001% (3)	0.003% (10)	0.003% (7)	0.007% (14)	0.04% (20)
Archaic Shared	0 - > 0	6% (8415)	5% (7517)	4% (6687)	4% (5564)	1% (664)
Archaic Shared	. -> 0	6% (8346)	5% (8208)	5% (7394)	4% (6223)	2% (968)
Archaic Shared	0 - > 1	0.1% (166)	0.2% (364)	0.2% (372)	0.2% (352)	0.3% (140)
Archaic Shared	1 - > 1	43% (63,368)	42% (64,940)	43% (64,647)	44% (62,349)	46% (23,863)
Archaic Shared	. -> 1	45% (65,871)	46% (70,073)	47% (70,219)	48% (68,013)	50% (26,483)
Archaic Shared	1 - > 0	0.01% (12)	0.03% (49)	0.04% (65)	0.05% (74)	0.01% (6)
Archaic New	0 - > 0	9% (4584)	8% (4625)	7% (4142)	7% (3538)	4% (437)
Archaic New	. -> 0	12% (5929)	11% (6668)	11% (6102)	10% (5385)	7% (820)
Archaic New	0 - > 1	0.2% (92)	0.2% (165)	0.3% (163)	0.3% (154)	0.3% (33)
Archaic New	1 - > 1	27% (13,885)	27% (15,765)	27% (15,411)	27% (14,028)	26% (2845)
Archaic New	. -> 1	51% (26,003)	53% (31,008)	54% (30,546)	55% (28,337)	62% (6878)
Archaic New	1 - > 0	0.01% (4)	0.01% (4)	0.01% (8)	0.01% (5)	0.01% (1)

A variant is defined as archaic if it is absent in African populations (Yoruba, Mende and Esan from the 1000 Genomes Project, Supplementary Data S2), present in non-Africans and Vindija Neanderthal is homozygous for that variant. We look at the variants inferred by imputation in archaic SNPs positions, separately for regions detected as Non Archaic, Archaic Shared and Archaic new. The first column, in the form “x->y” gives the variant in the original ancient individual genome (x) and after imputation (y). The variant can take the value “0” (non archaic), “1” (archaic) and “.” (missing). We then count for each coverage the number of sites corresponding to each possible value and the numbers in the parenthesis indicate the absolute values for each category. The percentages in each column add up to 100% within each Segment Classification (i.e., Non Archaic, Shared Archaic and New Archaic) written in the first column. To make the numbers more comparable, the counts for “Non-Archaic” are computed from a set of random segments chosen to have the same length distribution as the Archaic segments. Results are shown for all imputed datasets.

**Fig. 3:**
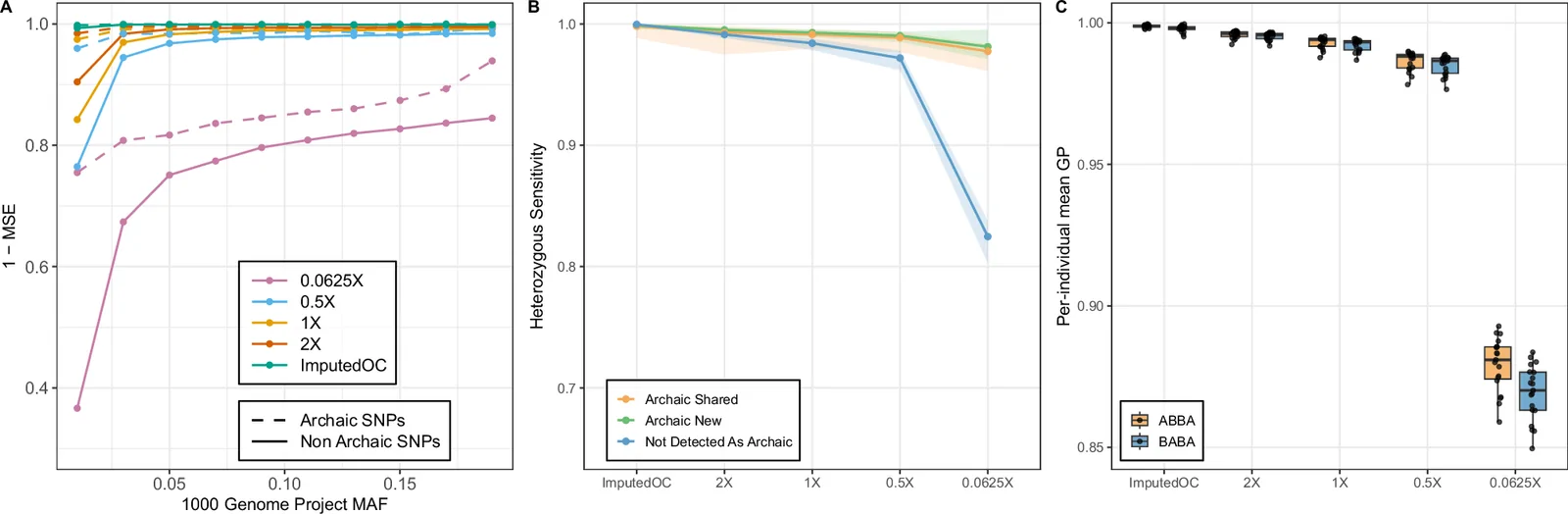
Imputation performance in archaic and non-archaic genomic regions. **A** Imputation concordance for archaic and non-archaic SNPs across different downsampled datasets and Minor Allele Frequency (MAF) in the reference panel. **B** Mean heterozygous sensitivity in the imputed downsampled datasets, measured with Picard, across three categories: (i) Archaic Shared—segments detected in both non imputed and imputed genomes; (ii) Archaic New—archaic segments identified only in imputed genomes; iii) Non Archaic. Colored shades represent the minimum and maximum values. **C** Per-individual average genotype probability for ABBA and BABA sites across different coverage levels, based on D-statistics of the form D(Africa, Ancient Individual; Altai Neanderthal, Chimp). *n* = 19 biologically independent individuals (excluding Mota) per coverage level and data type. Box plots show median (center line), 25th and 75th percentiles (box bounds), and whiskers extending to 1.5X the interquartile range; individual data points beyond whiskers are shown as outliers.

Imputation accuracy has been shown to be higher for alleles with a high frequency in the reference panel^9^. To assess the impact of MAF on the imputation of both archaic and non-archaic SNPs (independently of the classification of the region as archaic or non-archaic), we calculated the mean squared error between the imputed haplotype (using GP, see Supplementary Information) and the genotype in the original individual as a function of MAF in the 1000 Genomes. For non-archaic SNPs, our results are consistent with previous studies, showing similar values to those reported previously^9^ (Fig. 3A). As expected, the error is higher in genomes downsampled at lower coverage values and for variants with lower MAF, confirming that both coverage and allele frequency significantly influence imputation accuracy. Notably, there is a significant drop in accuracy between the 0.5X and 0.0625X genomes. This explains why we were able to infer nearly all archaic segments detected in the original genome using the 0.5X imputed data, but only 50% when using the 0.0625X imputed genome.

Strikingly, we observe a distinct pattern in the concordance of archaic SNPs (Fig. 3A). Although slightly influenced by MAF at very low frequencies, these SNPs consistently show higher concordance than non-archaic SNPs. Even at very low coverage, the accuracy difference between archaic and non-archaic SNPs remains significant, particularly for variants with low MAF (Supplementary Fig. 16A). One reason for the improved accuracy observed for archaic variants is that archaic SNPs tend to be in higher LD, particularly for older individuals^33^. We also note that due to their deeper evolutionary origin^34^, the archaic alleles show a lower proportion of variants at frequencies below 2% compared to non-archaic alleles, further improving imputation quality (Supplementary Fig. 17). When we compared the accuracy of archaic SNPs in inferred introgressed regions to their accuracy across the whole genome (Supplementary Fig. 16B), we observed that coverage and MAF have little to no impact at this level as the accuracy is consistently close to one. This indicates that introgressed segments are imputed with very high accuracy, and/or that LAI performs better in regions where imputation quality is high. The imputation accuracy of Denisovan SNPs shows a similar pattern, with consistently higher accuracy than non-introgressed SNPs (Supplementary Fig. 18).

We then expanded this analysis, examining heterozygous sensitivity across all variants considering three groups of segments: Archaic Shared, Archaic New and Non-Archaic. Once again, we observed higher sensitivity in archaic segments compared to non-archaic regions, with the difference between the two groups increasing as coverage decreases (Fig. 3B). This trend reaches its maximum in the 0.0625X downsampled imputed dataset, highlighting that the accuracy of imputation in archaic segments remains superior to that in non-archaic regions, especially under more challenging conditions. We do not observe a major difference between Archaic Shared and Archaic New SNPs, nor among individuals, with the exception of Mota (Supplementary Fig. 19).

To evaluate imputation in archaic regions that were low quality in the Original VCF and thus not directly assessed by concordance or heterozygous-similarity metrics, we focused on Archaic-New sites missing in the original calls and quantified read-level support in the BAMs. Imputed genotypes are largely supported by the reads; only a small fraction (≈5%) show poor support, and these cases are explained by low depth, typically <5 total reads (Supplementary Information, Supplementary Figs. 21–24).

Building on these findings, we revisited the allele frequency analyzes, where we previously observed an increase in *D* and *α* values when applying more stringent GP filters. To better understand this pattern, we examined how various factors—such as the genotype of the test individual, the ancestral alleles, and the amount of recoverable data—influence the D-statistics (Supplementary Information, Supplementary Fig. 16C). We observed that the average GP in ABBA sites (a proxy for archaic variants) is higher than in BABA sites (Fig. 3C, and Supplementary Fig. 20). Although this pattern is observed across all coverages, the difference becomes more pronounced at lower coverages, where imputation is inherently more challenging. Therefore, the increase in *D* values at low coverage and with a high GP filter (Fig. 1C, Filtered GP99) can be attributed to the fact that archaic variants are easier to impute than non-archaic variants, resulting in an enrichment of ABBA sites. This demonstrates that applying a GP filter—a standard quality control step in imputed data—can unintentionally introduce bias by over-representing ancestries that are more easily imputed, ultimately skewing the analysis.

### Analyses of archaic segments detected in ancient genomes

To further assess the quality of the identified archaic regions, we evaluated their utility in population genetic analyzes by comparing them to contemporary datasets and ancient individuals to determine how well they capture population structure, introgression patterns, genetic affinities between ancient and modern populations, and changes in the frequencies of potentially adaptive introgressed regions over time and space.

**Fig. 4:**
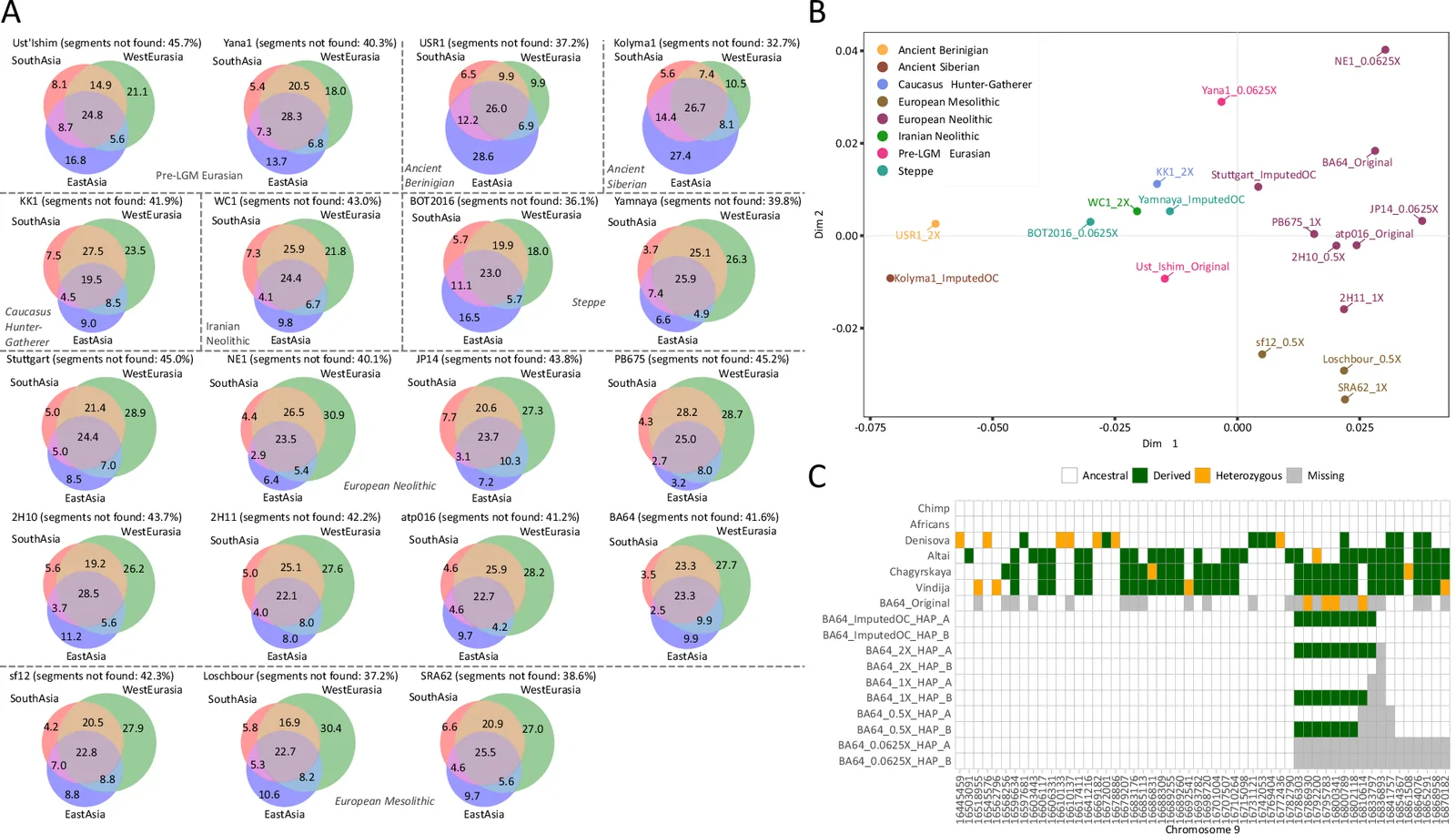
Comparative analysis of archaic segments in present-day and ancient individuals. **a** Venn diagram describing the proportion of shared archaic segments between imputed 1X ancient genomes and introgression maps from present-day individuals (Skov et al.^28^) **b** Multidimensional Scaling (MDS) analysis based on the count of introgressed segments among individuals from different imputed coverage datasets. **c** Haplotype-level heatmap of archaic-derived SNPs (Supplementary Data 5) in the *BNC2* gene region across chimpanzee, African, archaic, and non-imputed and imputed BA64 (Ireland, 3186 yBCE) genome after filtering for GP > 99. Each row represents a haplotype or diploid individual, and each column corresponds to a possible derived allele. Alleles are classified as Ancestral (white), Derived (green), Heterozygous (orange) and Missing (grey). Imputed genomes are split into phased haplotypes (HAP_A and HAP_B), while archaic and original genomes are unphased.

We looked at which contemporary populations contain the archaic segments detected in our ancient datasets. For this comparison, we used  the introgression maps from the hmmix^28^ and ArchaicSeeker2.0^29^ papers. For all six versions of our dataset we found a clear and consistent relationship between geography and amount of shared archaic segments between ancient and contemporary individuals (Fig. 4A, and Supplementary Figs. 25–29). For example, European Mesolithic and Neolithic individuals share most of their archaic segments with contemporary Europeans, while non-European individuals like Kolyma1 and USR1 show greater sharing with East and Central Asian populations, consistent with Denisovan introgression (Fig. 2D). These patterns remain consistent even at very low coverage (0.0625X), confirming that imputed genomes reliably capture geographic structure (Supplementary Figs. 25–27). This supports the use of imputed introgressed segments to trace past migrations and the spread of archaic ancestry over time.

This analysis shows that all ancient individuals, whether it is looking at original coverage, imputed 1X or imputed very low-coverage, have approximately 60% of their archaic segments shared with at least two groups (Fig. 4A, and Supplementary Figs. 28 and 29). Notably, Ust’Ishim, who is the oldest individual considered here, shows one of the highest proportions of archaic segments unshared with any contemporary individuals, reflecting its ancient position in the Eurasian genetic landscape. Interestingly, Ust’Ishim’s exhibits similar proportions of sharing with European and East Asian populations. Unlike other individuals, Ust’Ishim lacks a strong affinity toward any specific geographic group, with only around 15% of his segments shared across all regions and more than 50% found exclusively in a single region (though not consistently the same one). A similar pattern is observed for Yana1—the second oldest individual of our dataset—although Yana1 shares a little more exclusively with Europeans (~24–31%, Fig. 4A, and Supplementary Fig. 28). These results suggest that patterns of shared and unique archaic segments between Ust’Ishim and present-day population confirm the hypothesis that the population to which Ust’Ishim belonged diverged before the split of Europeans and East Asians^18^. Additionally, this suggests that differences in archaic introgression between Europeans and East Asians may not stem from multiple archaic sources but from ancient populations that once shared these variants^35^.

We then examined shared archaic ancestry among the ancient individuals analyzed in this work and found patterns consistent with those seen in modern populations, showing continuity between ancient individuals and their geographic regions. As expected, the oldest individuals do not preferentially share segments with any specific region, while European Neolithic and Mesolithic individuals share more with each other (Supplementary Figs. 30–32). This pattern is robust across all imputed coverages, including the very low-coverage dataset, though slightly less pronounced at the lowest depths.

To mimic real-world scenarios in which datasets are composed of individuals imputed from varying coverage levels, we created a mixed dataset of the 20 individuals analyzed, each randomly selected from either the imputed or original datasets available in this project. Using a multidimensional scaling (MDS) based on shared segment counts in this dataset, we observed consistent population structure, comparable to that of uniform-coverage datasets (Fig. 4C, and Supplementary Figs. 33–37). This structure aligns well with the geographic and temporal origins of individuals. Removing outliers (e.g., Mota and very low-coverage) further clarifies this structure (Supplementary Figs. 33–37), revealing a distinct East–West pattern. Eastern individuals carrying Denisovan segments cluster together, while Neolithic western individuals show proximity to Central Eurasian groups, reflecting their intermediate genetic profile. Overall, our results align with Iasi et al.^4^, confirming that archaic segments alone can recapitulate population structure—here demonstrated using a quantitative approach.

After demonstrating that imputed ancient genomes can be used to study genome-wide patterns of archaic ancestry, we assessed imputation performance at the gene level. Focusing on 14 candidate genes for adaptive introgression in modern humans^30^, we detected archaic segments overlapping 9 of these genes in one or more individuals from one or more coverage datasets (Supplementary Data 3 and 4). The number of carriers and the proportion of gene length covered by introgressed segments remain relatively consistent across coverages from 0.5X to imputed original (Fig. 4C, and Supplementary Data 3 and 4). Notably, imputation increased gene recovery from 6 (in non-imputed data) to 9. The *OAS1/2/3* genes are less prevalent in the past compared to other genes. Among the genes analyzed, *TLR6-1-10* show high similarity to Neanderthal haplotypes and are found at relatively high frequencies in Europe since the Mesolithic (2 out of 3) and Neolithic (5 out of 8), with the exception of the ancient Siberian Kolyma1 individual (Supplementary Data 4). *BNC2* exhibits the highest frequency, particularly in post-LGM West Eurasians. It is found in 2 out of 3 European Mesolithic individuals and 7 out of 8 European Neolithic individuals, and outside Europe, it appears in Caucasus Hunter-Gatherers and Steppe populations.

We directly examined the derived alleles in *BNC2* (Supplementary Data 5) and confirmed that the high proportion of missing positions prevents the identification of archaic introgression in the original genome even in the presence of derived alleles (Fig. 4C). This suggests that even in genomes sequenced at relatively high coverage, imputing missing genotypes can help resolve haplotypes in specific regions of interest.

**Fig. 5:**
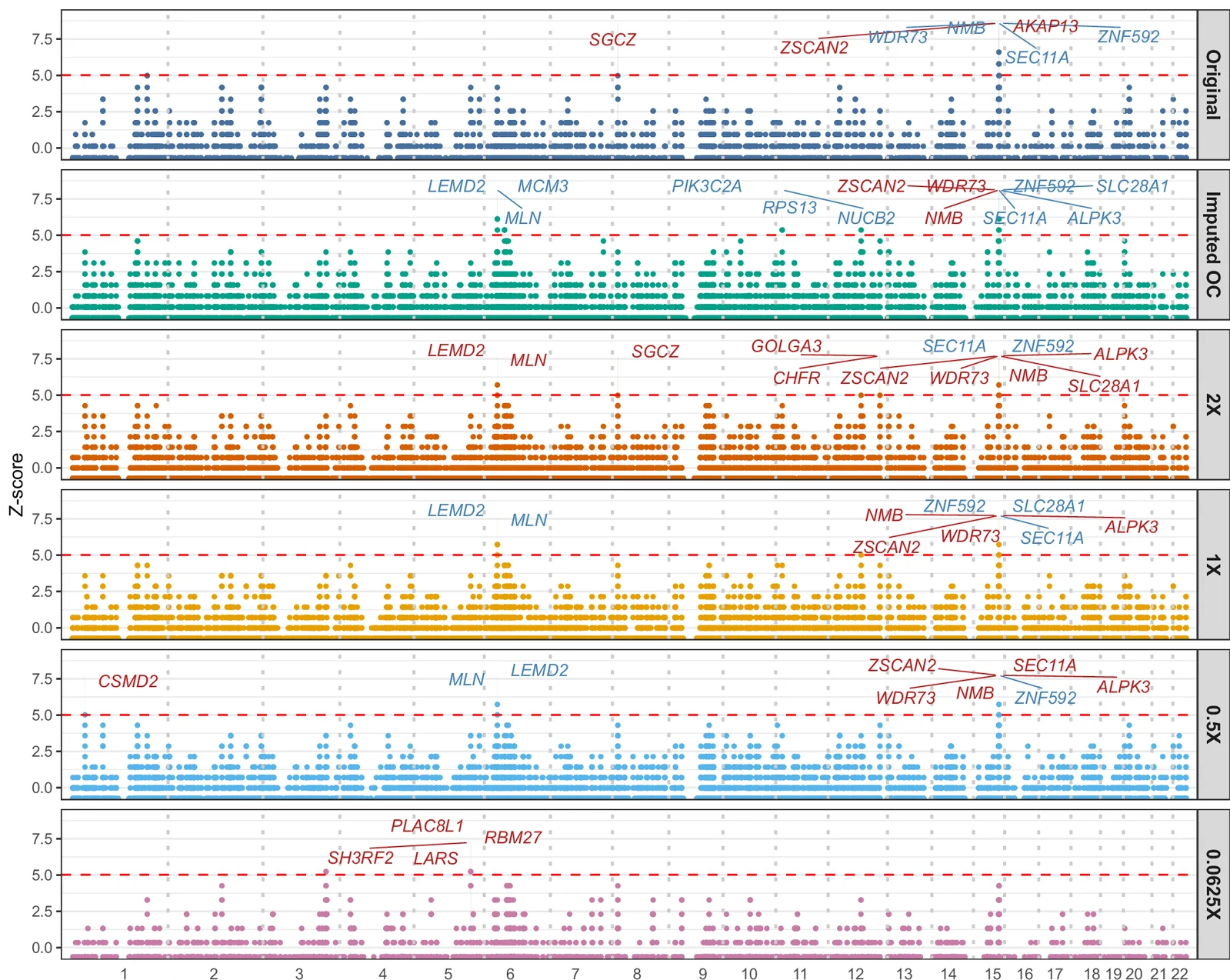
Overrepresented archaic introgressed segments in 11 ancient European individuals. Manhattan plot of frequencies of archaic introgressed regions in 11 ancient European individuals. The *Y*-axis shows *Z*-scores. *Z*-scores were computed for each genomic interval to measure deviations from the mean archaic segment-sharing frequency across individuals. One-tailed *p*-values were calculated to test for significantly elevated archaic segment sharing. Bonferroni correction was applied for multiple testing across all genomic intervals. The red dashed line indicates the significance threshold (adjusted *p*-value < 0.01). Gene labels (red text) mark regions with core significant intervals; gene labels (blue text) mark regions with suggestive evidence (adjusted *p*-value < 0.05) anchored by at least one core significant interval.

To explore the potential for identifying additional adaptive introgression candidate genes—despite the limited number of individuals analyzed—we examined regions statistically over-represented in the 11 European individuals included in our study. Significantly shared archaic segments were identified for each coverage level using *z*-scores of segment presence frequencies across individuals, with one-tailed *p*-values adjusted via Bonferroni correction. Regions with an adjusted *p*-value < 0.01 were considered over-represented and were expanded to include adjacent intervals with adjusted *p*-values < 0.05, to account for broader signals and the uncertainty in segment length typical of ancient DNA. These regions contain 17 protein-coding genes (Supplementary Table 3), mostly grouped into two main extended regions (Fig. 5). The peak distributions are consistent across coverage levels, though statistical significance varies depending on the number of segments per dataset. Datasets with fewer segments—Original and 0.0625X—may still reach significance due to missing data and reduced segment counts (Supplementary Data 6). One of these two regions, located on chromosome 15, contains genes already known to be introgressed, such as *SLC28A1*^36^, and *WDR73*. *AKAP13*^37^ is significant in the original data, but not in the imputed data. Specifically, AKAP13 is present in 7 out of 8 Neolithic Europeans but only in 1 out of 3 Mesolithic individuals, failing to meet the minimum threshold for significance in imputed datasets.

Another extended region, located on chromosome 6 at the very beginning of the 6p21.3 band, is not present in the Original data. When analyzing the genes in the region, *LEMD2* and *MLN*, as with *BNC2*, we observe the presence of archaic alleles (Supplementary Data 7) in the Original data, but there is a substantial amount of missing data (Supplementary Fig. 38). In the imputed genomes, we are able to resolve this issue, retaining heterozygous sites in all individuals except for individual sf12 (Sweden, 8895 BCE), who is homozygous in both the original and imputed data. The introgression is present in all the European individuals, except for Stuttgart. With imputation, we can shift the analysis to the haplotype level, where the introgressed haplotype is closer to the Vindija Neanderthal haplotype than to those present in the other high-coverage archaic genomes. This haplotype is completely absent in the 9 non-European individuals analyzed in this study (Supplementary Fig. 38B). These analyzes suggest *LMED2* and *MLN* as promising candidates for adaptive introgression that provide an example of the benefits of imputation.

## Discussion

In this study, we evaluate the effectiveness of inferring archaic ancestry from imputed low-coverage ancient genomes, showing a surprisingly higher effectiveness of the imputation in recovering archaic haplotypes compared to non-archaic haplotypes. This opens the possibility of using, in the context of ancient DNA, methods initially designed to study archaic introgression in high-coverage genomes. Specifically, we downsampled 20 high-coverage (>10X) ancient genomes to four low-coverage levels (2X, 1X, 0.5X, and 0.0625X), imputed them as well as the original high-coverage data, and compared patterns of archaic introgression — using D-statistics, f4-ratio and hmmix — between non-imputed and imputed datasets. The results obtained with the imputed dataset were consistent across all five coverage values for *D-* and f4-ratio statistics (Fig. 1, and Supplementary Fig. 1). The results on the imputed data are also comparable with those obtained from pseudohaploidized data when using a transversion-only filter. We show that applying stringent genotype probability filters to the imputed data (GP > 99%) biases the D-statistic values and produces a slight overestimation of the *α* at very low coverage (0.0625X). We discovered that this overestimation occurs because the quality of imputation of archaic variants is higher (higher GP) than non-archaic variants, especially at low coverage (Fig. 3C). To solve this problem, we extended the D-statistics to directly use GP as an input instead of the most likely genotype. This shows very stable results across coverages (Supplementary Fig. 3) and is a promising direction to adapt methods to imputed data. These results suggest that imputation can be effectively used in allele frequency analyzes, providing an alternative approach to pseudohaploidization methods. Notably, unlike pseudohaploid data, imputation enables the use of all SNPs, rather than being restricted to transversions only.

Using hmmix to identify archaic segments, we find that we can identify nearly all archaic fragments in imputed individuals (that are present in the original individual data) even when we impute from as low as 0.5X and as much as 50% of the segments when we impute from 0.0625X (Fig. 2B). Notably, we identify more archaic fragments in imputed individuals than in the original data, and we confirm the archaic origin of these “new” segments by looking at the similarity of the original genomes in these regions with the archaic references. We showed for LAI the importance of imputation in resolving some of the missing genotypes that make it harder to detect these segments in original genomes (Table 1, Fig. 4C, and Supplementary Fig. 38). Moreover, we also checked the ability to identify Denisovan segments in Siberians and Arctic Americans individuals, and inferred all the original segments and identify additional ones in the imputed genomes (Fig. 2D, and Supplementary Fig. 11).

Despite the effectiveness of imputation, we acknowledge that it underperforms in reconstructing the full length of archaic segments, especially in the oldest individuals (Ust’Ishim and Yana1), where the expected pattern of longer segments is reduced compared to original data (Supplementary Fig. 14). A similar bias is seen when comparing Archaic New and Archaic Shared segments (Supplementary Fig. 15A). Therefore, we caution against using segment length to estimate the timing of introgression using imputed genomes, and future work accounting for this bias may lead to more accurate estimates of the length of introgressed fragments. We also note that inferring introgressed segments from imputed data sometimes produces false positives, and it is important to filter carefully as we have done here. In particular, we applied stringent filters based on segment length, similarity to the archaic genomes, and the number of archaic alleles present in the introgressed segment. This approach removed a large proportion of false positives, though more specific filters can be applied depending on the scope of the analysis.

One surprising result is that imputation performs better in archaic versus non-archaic regions and variants, and imputation correctly assigns archaic variants within archaic regions and non-archaic variants within non-archaic regions (Table 1). Previous studies have shown that imputation accuracy improves with stricter GP filters (e.g., GP > 80%) and when focusing on common variants (MAF > 5%). We therefore tested the genotype accuracy inference in function of MAF (Fig. 3A) as well heterozygous sensitivity (Fig. 3B)—i.e., the imputation quality at heterozygous sites, which are typically more challenging to impute, we find that accuracy is higher for both archaic regions and archaic SNPs.

We also observed similar heterozygous sensitivity at both Archaic New and Archaic Shared segments, suggesting that SNP density in the original data has a reduced impact within archaic regions (Fig. 3B). In sum, our results highlight the potential of imputation as a reliable approach for analyzing archaic introgression, especially in the growing number of ancient genomes available from recent studies.

Finally, we assessed the potential applicability of the archaic segments identified in imputed genomes in real-case scenarios. First, we compared the introgression maps generated in this study with those obtained from present-day populations in previous works^28,31^. Across all six datasets (one non-imputed and five imputed), we observed consistent geographic patterns: ancient European individuals share more archaic segments with contemporary Europeans, while Kolyma1 and USR1 align more with East Asians (Fig. 4, and Supplementary Figs. 25–29). Ust’Ishim, as expected, shows no regional affinity and has a high proportion of unshared segments, supporting its placement before the West–East Eurasian split. This suggests that regional differences in archaic ancestry between Europeans and East Asians may have emerged from a shared ancestral pool rather than multiple admixture events. We found similar patterns of geographic structure among ancient individuals, stable across all coverage levels (Supplementary Figs. 33–36). To mimic real-world datasets, we assembled a dataset of 20 individuals, randomly selected from the 6 coverage datasets, and performed MDS on the shared segments (Fig. 4B, and Supplementary Fig. 37). The resulting structure aligns with temporal and geographic origins, demonstrating that population structure can be identified using only archaic segments and confirming that imputation preserves population signals, even in mixed-coverage scenarios.

We also assessed whether imputation could help detect regions of overrepresented archaic introgression, potentially reflecting adaptive events. Of 14 candidate genes, 10 were recovered in imputed genomes,4 of which were not detected in the original data (Supplementary Data 3). We show that Imputation helps to identify more archaic haplotypes, especially in Archaic New segments missed in original genomes due to lower SNP density (Fig. 4C, and Supplementary Fig. 38). Focusing on Neolithic and Mesolithic Europe, we identified two overrepresented gene clusters consistently across coverage levels, with significance varying by segment count (Fig. 5). We provide an example of a gene region (e.g., *LEMD2* and *MLN*) of how imputation improves the detection of a putative selected archaic haplotype present in 10 out of 11 Europeans, absent in the non-European individuals, and most similar to the Vindija Neanderthal haplotype (Supplementary Fig. 38).

We acknowledge that we have tested our ability to detect introgression using only 20 ancient individuals, which is not representative of all ancient human populations. However, these individuals cover a large time and geographic distribution, which suggests that imputation is likely to work on other ancient individuals. Here we have only used hmmix, which we know may have missed some regions of archaic ancestry because it does not use the archaic genomes for the inference. One future research direction may involve extending hmmix to also leverage the archaic human genomes in the inference step^38^ or including the archaic genomes in the imputation reference panel, which may improve the recall of genotypes with an archaic allele.

In summary, we show that, despite the limitations of relying on a predefined set of sites based on present-day variation (which may miss ancient private variants), imputation improves the inference of archaic introgression in ancient genomes. With imputed genomes, we can obtain reliable measures of the introgression proportion and identify introgressed fragments. and recapitulate broad temporal and geographic patterns. Imputation also helps resolve archaic haplotypes in ancient genomes, making it a promising strategy to study fine-scale signatures of adaptive introgression through time. As the number of low-coverage ancient genomes rapidly increases, imputation will vastly reduce the amount of missing data, and as imputation works better in introgressed regions, it will be useful to characterize archaic ancestry over time and space.

## Methods

### Ethics

This study benchmarks the use of imputed genomes to infer archaic ancestry in ancient individuals. Previously published ancient genomes were downsampled, and genotypes were subsequently imputed across the 22 autosomes using the 1000 Genomes Project dataset of publicly available genomes belonging to present-day individuals. As analyzes were restricted to autosomal data, sex and/or gender were not considered.

No new data were generated; accordingly, no additional ethical approval was required. All analyzes were conducted in accordance with the ethical guidelines and permissions reported in the original publications.

### Dataset

We assembled a dataset of 20 previously published^5,12–24^ high-coverage (>10X) ancient genomes (44,000–5000 BCE) from Eurasia, the Americas, and Africa (Supplementary Data 1). The reads were realigned with bwa^39^ to the hs37d5 reference genome. After alignment, the reads were sorted, and PCR duplicates were removed. Realignment around indels and addition of read group information were performed. To mitigate the impact of postmortem damage (PMD), two base pairs were clipped from both ends of each read. Only reads with a minimum mapping quality of 37 and length of at least 30 bp were retained. Each genome was downsampled to 2X, 1X, 0.5X, and 0.0625X coverage using samtools^40^.

For each individual sample, the genotype likelihood was obtained at ~78 million biallelic SNPs from the 1000 Genomes Project Phase III^25^ using bcftools^40^ and independently imputed with GLIMPSE^6^ using the same panel as reference. Pseudohaploid calls were also generated at the same sites using the *HaploCall* method implemented in ANGSD^41^. Genotypes for the same set of SNPs were called from the original data using bcftools (v1.10.2) with filtering parameters that excluded bases with mapping quality <30 (-Q 30), removed indels (-I) and extended BAQ (-E), retained annotations for depth (DP), strand bias (SP), and allele depth (AD), and set a maximum read depth of 700,000 (-d 700000). Two additional filters were added: Depth of Coverage (DP) ≥ 10, and Allele Balance ≥ 0.4.

The dataset additionally includes four high-coverage archaic genomes, Altai Neanderthal (Denisova 5), Vindija 33.19, Chagyrskaya 8, and Denisova 3^10,42–44^, 292 African genomes from 1000 Genomes (YRI, ESN, MSL, Supplementary Data 2), and chimpanzee (panTro6) as an outgroup.

For some analyzes, the individual VCF files were merged and filtered based on genotype probability (GP) and minor allele frequency (MAF) using bcftools. For allele frequency analyzes, only positions covered in both at least one archaic and the chimpanzee genome were retained. For AdmixTools analyzes, the merged VCF datasets were first converted to PLINK files^45^ while preserving reference and alternative allele information with the flag --keep-allele-order, and then converted to EIGENSOFT format using the convertf utility from the AdmixTools package^26^.

### Statistics & reproducibility

#### Allele frequencies analysis

D-statistics were computed with qpDstat from the AdmixTools package in the form D(Unadmixed modern humans, Test individual; Archaic individual, Outgroup). The Altai Neanderthal genome was used as an archaic reference, 292 African genomes from the 1000 Genomes Project Phase 3 as the modern reference, and chimpanzee as the outgroup. Analyzes included imputed, pseudohaploid, and original genotypes (Fig. 1C, and Supplementary Figs. 1 and 2, Source Data 1). For imputed data, three filters were applied: Raw, GP ≥ 0.99, and MAF ≥ 0.5%. For pseudohaploid data, both all SNPs and transversions-only sets were used (Fig. 1C, and Supplementary Figs. 1 and 2, Source Data 1). To mitigate the bias observed when using GP-filtered data, we computed *D*-statistics directly from imputed genotype probabilities (GP0|0, GP0|1, GP1|1), deriving allele frequencies as GP0|0 + ½GP0|1 and GP1|1 + ½GP0|1 (Supplementary Fig. 3, Source Data 1). All remaining components of the statistic were computed using standard definitions. The implementation is available at: https://github.com/LeoPlanche/arcAncientImputed/blob/main/src/AlleleFrequencies/dstat.py.

We quantified potential sources of bias in *D*-statistic estimation by measuring the proportion of features (total positions and genotype classes: homozygous reference, homozygous alternative, heterozygous) across genotype probability (GP) bins of width 10%. The RIPTA script^46^ was modified to extract GP values for ABBA and BABA sites. For each coverage level and individual, we plotted feature proportions across GP bins (Supplementary Fig. 16C), and calculated average GP values for ABBA and BABA sites (Fig. 3C, Source Data 2).

To estimate the proportion of Neanderthal archaic introgression, we applied the *f4-ratio* method from the AdmixTools package^26^ in the form: f4(Altai, Chimp; Test Individual, Africa) / f4(Altai, Chimp; Vindija, Africa) (Fig. 1D, and Supplementary Fig. 4, Source Data 3). For correlation analyzes, α (f4-ratio values) was tested against individual age (in thousands of years BCE) and original sequencing coverage (Supplementary Fig. 5). The Mota individual was excluded from this analysis.

#### Local archaic ancestry inference

Archaic segments were inferred using a custom version of hmmix^28^ using 292 African genomes from the 1000 Genomes Project as the outgroup (Supplementary Data 2). Analyzes were performed individually for each genome across six datasets (original plus five imputed). For imputed data, unphased genotypes filtered for GP ≥ 99% were used. Resulting introgression maps, including similarity and distances to archaic reference genomes (Vindija Neanderthal and Denisova 3), and number of private archaic SNPs are available in the Zenodo repository^47^ and at:

https://github.com/LeoPlanche/arcAncientImputed/tree/main/data/maps.

The proportion of the genome identified as introgressed was calculated as the total length of introgressed segments divided by the length of the hg19 reference genome (Fig. 2A). Segment recovery in imputed datasets was assessed by comparing overlap with segments identified in the original dataset, considering segments recovered with at least 1 base overlapping (Fig. 2B).

For each detected segment, similarity to the Vindija Neanderthal and Denisova 3 genomes was calculated by comparing all SNPs within the segment using both the original and imputed data, with a match defined when an allele in the archaic haplotypes matched an allele in the individual haplotypes. The normalized distance was calculated by computing the similarity between the individual’s original haplotypes and the archaic reference haplotypes, and subtracting the average similarity between Biaka individuals from the HGDP dataset and the same archaic references. To reduce false positives, we applied filters to the segments identified as introgressed with hmmix. Segments were retained if they showed a similarity (using the imputed data) ≥0.99 to the closest archaic genome (Vindija for Neanderthal, Denisova 3 for Denisovan) and had a length ≥ 40 kb^29^, corresponding to the threshold required to reject incomplete lineage sorting under moderate recombination rates^29^.

A segment was classified as Denisovan if it met five criteria: (i) it was detected as archaic, (ii) had a length above 100 kb, (iii) had a similarity of at least 0.99 to one of the high coverage archaic genome, (iv) had a at least 10 private Denisovan variants and (v) had more Denisovan private variants than Neanderthal private variants.

#### Imputation accuracy in archaic vs non archaic regions

Imputation accuracy was assessed at each SNP using genotype probabilities (GP) from imputed genomes, with genotypes defined as GP1|1 + ½ GP0|1. Accuracy was measured as 1—MSE relative to the original genotype. SNPs were classified as archaic or non-archaic, where archaic variants were defined as alleles absent in Yoruba, Mende, and Esan (1000 Genomes Project) and homozygous in the Vindija Neanderthal. For each individual, archaic positions were compared between imputed and non-imputed genomes (Source Data 4). Analyzes were also performed using identified archaic segments, with a random set of non-archaic segments of matched length distribution as control. Comparisons were further divided into Archaic New and Archaic Shared categories. Additionally, imputation was evaluated within archaic (Archaic New and Archaic Shared) and non-archaic segments using heterozygous sensitivity, measured with the GenotypeConcordance tool from Picard 3.0 by comparing original and imputed VCFs (Source Data 5-6).

#### Analysis of shared introgressed regions with contemporary individuals

For each ancient individual, we measured the overlap of archaic segments with contemporary populations. Contemporary introgression maps were obtained from the hmmix paper (Simons Genome Diversity Project, 40 Papuans from ref. ^48^, 35 Papuans from ref. ^49^) and the ArchaicSeeker2.0 paper (1000 Genomes Project and Papuans from SGDP^50^).

In the hmmix map, individuals were grouped into four regions (East Asia, West Eurasia, South Asia, Central Asia–Siberia). In the ArchaicSeeker2.0 map, individuals were assigned to 1000 Genomes populations, aggregated into continental regions (East Asia, West Eurasia, South Asia).

For heatmaps (Supplementary Figs. 25–27, Source Data 8), overlaps were defined as ≥80% segment length. Matches were counted per region, adjusted by the number of individuals in each region, and normalized by row. For Venn diagrams (Fig. 4A, and Supplementary Figs. 28 and S29, Source Data 9), a segment was considered present in a region if ≥3 contemporary individuals showed overlap in the hmmix map; otherwise, it was marked absent.

Denisovan segments were further compared with published introgression maps generated using hmmix and ArchaicSeeker to assess overlap (Supplementary Fig. 11). For imputed data, Denisovan segments were categorized as Denisovan Shared (detected in both original and imputed datasets) or Denisovan New (detected only in imputed data).

#### Analysis of shared introgressed regions across ancient individuals

To study the shared archaic segments among the individuals in our dataset, we applied a multi-intersection strategy to introgressed fragments inferred by local archaic ancestry analysis. For each coverage level, segments were grouped by chromosome and divided into non-overlapping genomic intervals defined by shared breakpoints across individuals. This standardized coordinate system was used to build binary matrices recording the presence/absence of archaic fragments per individual, analogous to bedtools multiintersect. Adjacent contiguous fragments shared by overlapping individuals were merged into larger blocks.

From this collapsed matrix, we computed a pairwise segment-sharing matrix by counting introgressed regions shared between each pair of individuals, then row-normalized it to represent the proportion of segments each individual shared with others.

To control for coverage differences, we subsampled a balanced set of individuals per coverage level. For this subset, we calculated the total length of introgressed fragments shared between pairs, defined as the sum of minimum overlapping lengths in shared intervals. The pairwise matrix was row-normalized by each individual’s total archaic introgressed length. Finally, we applied MDS to the normalized distance matrix, generating two-dimensional coordinates for scatterplots (Fig. 4B, and Supplementary Figs. 30–37).

### Adaptive introgression

To assess the presence of archaic introgressed fragments at previously proposed adaptive introgression candidate loci, we curated a list of 14 genes of interest^30^ and retrieved their genomic coordinates. We then intersected these gene regions with the introgression maps obtained from the ancient genomes, after quality filtering. For each gene, we computed per-individual, per-gene summary statistics, recording the average similarity and genetic distance to archaic references across all overlapping fragments, stratified by coverage level and individual (Supplementary Data 3 and 4).

As an example of a candidate gene, we further analyzed the *bnc2* locus at the VCF level. Informative SNPs were identified by comparing allele states among archaic, African, and chimpanzee genomes. SNPs were retained if at least one archaic carried the derived allele while all Africans were homozygous for the ancestral allele, with chimpanzee genotypes used to define ancestral and derived states. (Supplementary Data 5). Genotypes for these sites were extracted from the genome of the ancient individual BA64 and its imputed datasets, then converted into haplotype labels (Ancestral, Derived, Heterozygous, Missing) (Fig. 4C).

We conducted a genome-wide scan for overrepresented archaic fragments in Mesolithic and Neolithic European individuals, using both the original genomes and five imputed datasets. For each coverage level, a binary presence/absence matrix of archaic segments per interval was generated from the introgression maps after quality filtering. *Z*-scores were calculated for segment-sharing frequency, and one-tailed *p*-values were derived to identify intervals with significantly elevated archaic sharing. Bonferroni correction was applied, and significant intervals (adjusted *p* < 0.01) were expanded to include adjacent intervals with suggestive evidence (adjusted *p* < 0.05). Candidate regions were annotated using Ensembl to identify overlapping protein-coding genes, and gene hits were tracked across coverage levels (Supplementary Table 3, and Supplementary Data 6). Results were summarized in Manhattan plots with *Z*-scores and gene annotations stratified by coverage (Fig. 5). We then examined an extended region of enriched archaic fragments on chromosome 6 (6p21.3, spanning *LEMD2–MLN*). At this locus, SNPs were selected where Africans were homozygous ancestral (chimpanzee-defined) and at least one high-coverage archaic genome carried the derived allele. Twenty such positions were identified (Supplementary Data 7), and genotypes were encoded as ancestral, derived, heterozygous, or missing (Supplementary Fig. 38A, B).

### Reporting summary

Further information on research design is available in the Nature Portfolio Reporting Summary linked to this article.

## Supplementary information

Supplementary Information

Description of Additional Supplementary Files

Supplementary Data 1-8

Reporting Summary

Transparent Peer Review file

## Source data

Source Data

## Data Availability

This study does not generate new sequencing data. References to the original published sequencing datasets are provided in Supplementary Data 1. The ancient individual genotypes (original and imputed, VCF format) and introgression maps generated in this study are available on Zenodo^47^ (https://doi.org/10.5281/zenodo.16365478) and on GitHub (https://github.com/LeoPlanche/arcAncientImput). The modern reference panel used was the 1000 Genomes Project Phase III dataset, available at https://ftp.1000genomes.ebi.ac.uk/vol1/ftp/release/20130502/. Archaic genome genotypes were downloaded from the MPI-EVA FTP server https://ftp.eva.mpg.de/neandertal/Vindija/VCF/ and https://ftp.eva.mpg.de/neandertal/Chagyrskaya/VCF/. Introgression maps for contemporary individuals were obtained from Dataset S5 of hmmix^28^ https://doi.org/10.1371/journal.pgen.1007641.s012 and the GitHub repository of ArchaicSeeker2.0^31^ https://github.com/Shuhua-Group/ArchaicSeeker2.0/tree/master/IntrogressedSeg^47^. Source data are provided with this paper.

## Code availability

All scripts required to reproduce the figures and tables presented in this study are available under a Creative Commons Attribution 4.0 International license on Zenodo^47^ (https://doi.org/10.5281/zenodo.16365478) and GitHub.
