# Supplementary material for: Archaic ancestry inference in imputed ancient human genomes
Source: Nat Commun. 2026 Aug 6;17:9480. doi: 10.1038/s41467-026-76204-0 (PMC13542231; doi:10.1038/s41467-026-76204-0)
Supplement: Supplementary file 1 [file 41467_2026_76204_MOESM1_ESM.pdf]

Archaic ancestry inference in imputed ancient human genomes

Capodiferro M.R.[1+*], Planche L.[2+*], Breslin E.M.[1], Ongaro L.[1], Ávila-Arcos M.C.[3], Jay F.[2], Cassidy L.M.[1], Huerta-Sanchez E. [1,4*]

[1] Smurfit Institute of Genetics, Trinity College Dublin, D02 CX56 Dublin 2, Ireland

[2] Interdisciplinary Laboratory of Digital Sciences, Université Paris-Saclay, CNRS, INRIA, Orsay, 91400, France

[3] International Laboratory for Human Genome Research, Universidad Nacional Autónoma de México, Querétaro, México.

[4] Ecology and Evolutionary Biology and Center for Computational and Molecular Biology, Brown University, Providence, RI 02906, USA

[+]These authors contributed equally.

[*] Corresponding authors.

Supplementary Information

1. Dataset ... 2
2. Allele frequencies analysis - Archaic Global Ancestry Inference ... 3
3. Local archaic Ancestry Inference ... 9
4. Local archaic Ancestry Inference on 1240K SNP-capture data. ... 11
5. Similarity and distance to Archaic genomes ... 13
6. Filtering of introgressed segments ... 16
7. Identification of Denisovan origin introgressed segments ... 19
8. Summary of introgression in present-day individuals based on Skov et al. maps ... 21
9. Quality of the introgressed segments ... 23
10. Accuracy of archaic alleles vs MAF ... 26
11. BAM-derived support for imputed variants in Archaic New segments ... 31
12. Analysis of Shared Introgressed Regions with Contemporary Individuals ... 36
13. Analysis of shared introgressed regions across ancient individuals ... 40
14. Analysis of Known Candidate Genes ... 47
15. Selection Scan on Imputed and Non Imputed European Genomes ... 48

Supplementary References ... 52

1. Dataset

Our strategy to assess the feasibility of using imputed ancient genomes to study archaic introgression in humans is based on a comparative approach, evaluating results from specific analyses using imputed downsampled genomes alongside those obtained directly from non imputed original genomes (Figure 1A). We selected and downloaded 20 previously published high-coverage (>10X in ancient DNA, aDNA) shotgun genomes (Supplementary Data 1) from public repositories, representing ancient individuals from Eurasia, the Americas, and Africa, and spanning a broad temporal range—from approximately 44,000 to 5,000 years Before Common Era (BCE) (Figure 1B)[1–14].

The genomic data were realigned with bwa[15] to the same reference genome (hs37d5). First, reads were sorted and PCR duplicates were removed. Realignment around indels and addition of read group information were performed. To mitigate the impact of postmortem damage, two base pairs were clipped from both ends of each read. Only reads with a minimum mapping quality of 37 and length of at least 30 bp were retained. These genomes were then downsampled to four lower coverage levels (2X, 1X, 0.5X, and 0.0625X) using samtools[16].
Genotype likelihoods were called using bcftools[16] for approximately 78 million biallelic SNPs from the 1000 Genomes Project Phase III[17], both for the downsampled and original genomes. These served as input for imputation with GLIMPSE[18], using the 1000 Genomes Project Phase III panel as a modern reference. Each individual was imputed independently and after imputation they were merged based on the coverage in five, one from each of the four downsampled coverages, plus imputation of the original data.
Various filters, such as Genotype Probability (GP) and Minor Allele Frequency (MAF) in the reference panel, were applied in different analyses to minimize potential biases introduced by imputation, as shown in previous studies[19]. Additionally, genotype likelihoods were called from the original genomes at the same SNP positions and filtered using a Genotype Quality (GQ) ≥ 30, Depth of Coverage (DP) ≥ 10, and Allele Balance ≥ 0.4. These high-confidence genotypes were treated as the truth set for comparison with the imputed data to evaluate imputation accuracy.
From the same original and downsampled BAM files, we also generated pseudohaploid calls at the same ~78 million positions using the HaploCall method implemented in ANGSD[20]. This allowed us to compare the imputation results with the most commonly used data type in low-coverage aDNA studies.

Other published data were also included in various analyses. The four available high-coverage archaic genomes—three Neanderthals (Altai (Denisova 5), Vindija 33.19, and Chagyrskaya 8) and one Denisovan (Denisova 3)—were used as archaic references[21–24]. African individuals were represented by 292 genomes from the 1000 Genomes Project Phase 3, specifically from the Yoruba (YRI), Esan (ESN), and Mende (MSL) populations (Supplementary Data 2). The chimpanzee genome (panTro6) was used as an outgroup.

2. Allele frequencies analysis - Archaic Global Ancestry Inference

D-statistics

To identify archaic introgression, the most widely used approach is based on the D-statistics (also known as ABBA-BABA or Patterson's D-statistics[25]). This method compares the allele sharing between a test individual or population and an archaic individual, relative to the sharing observed in an unadmixed modern population (Africans) with the same archaic individual.

To evaluate the ability to detect allele sharing between archaic and imputed genomes, we applied D-statistics using the qpDstat software from the AdmixTools package [25], in the form:

D(Unadmixed modern humans, Test individual; Archaic individual, Outgroup).

For this analysis, we used the Altai Neanderthal genome as the archaic reference, a set of 292 unadmixed African individuals from the 1000 Genomes Project Phase 3 (Supplementary Data 2) as the modern reference population, and the chimpanzee genome as the outgroup.

The D-statistics were computed for the imputed, pseudohaploid, and original genotypes (Figure 1C, Supplementary Fig. 1-2). For the imputed data, we applied three different filtering strategies i) Raw (no filtering), ii) Filtered for GP ≥ 0.99, iii) Filtered for Minor Allele Frequency (MAF ≥ 5%) in the reference panel, following the approach of Sousa da Mota et al. 2023[19]. For the pseudohaploid data, we used two sets of variants: i) all SNP positions, ii) only transversions (Source Data 1).

The transversion-only filter was applied to all ancient genomes, regardless of whether UDG treatment had been used in library preparation to mitigate post-mortem damage (PMD) effects.

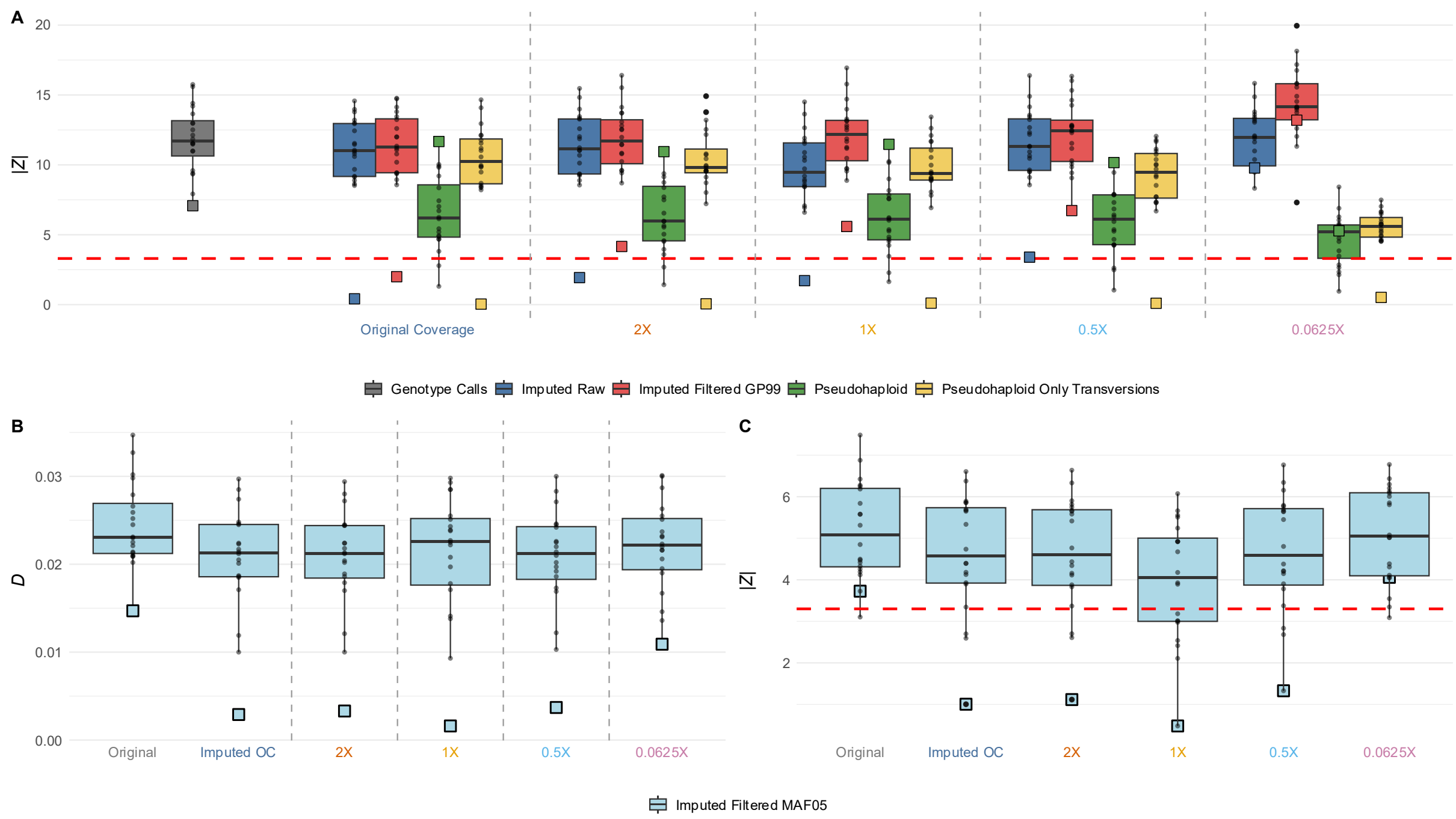

Supplementary Fig. 1. D-statistics. (A) Absolute Z-scores (|Z|) for the 20 individuals for different data processing methods and coverages, with highlighted "Mota" (black outlined points). The red dashed line represents the |Z| threshold of 3.3. (B) Distribution of D-statistics for the 20 individuals across coverages after filtering for Minor Allele Frequencies of 5% in the reference panel. (C) Absolute Z-scores (|Z|) for the 20 individuals after filtering for Minor Allele Frequencies of 5% in the reference panel, with "Mota" highlighted (black outlined points). Box plots show median (center line), 25th and 75th percentiles (box bounds), and whiskers extending to 1.5X the interquartile range; individual data points beyond whiskers are shown as outliers.

We observed broadly consistent results across datasets. In particular, the raw imputed and the pseudohaploid (transversions-only) datasets show very similar distributions, with comparable means across the 20 individuals. In contrast, for pseudohaploid (all SNPs) we observe an underestimation of the D-statistics.

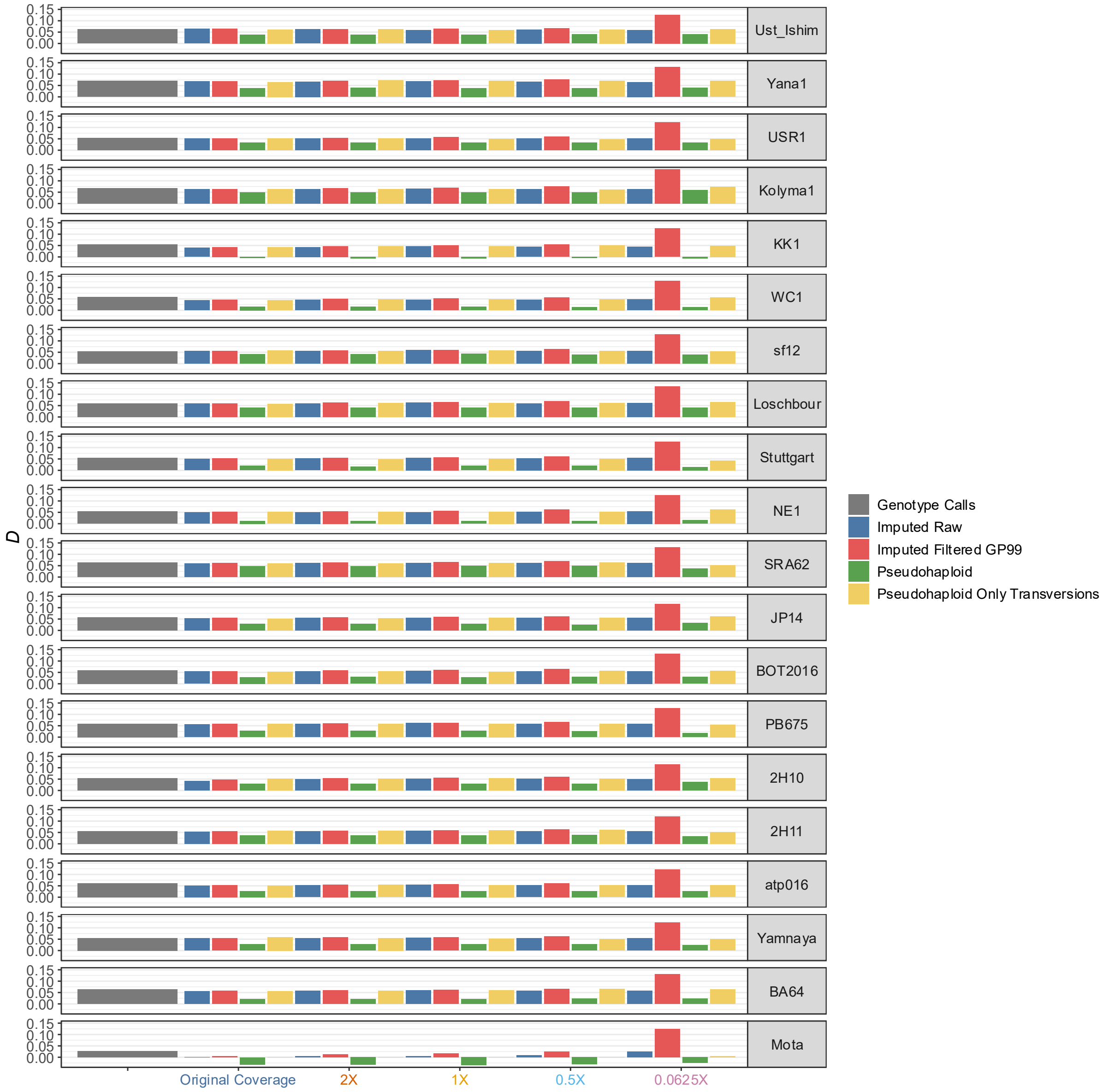

Supplementary Fig. 2. Barplot of D-statistics results as in Figure 1C, but facet per individual.

Despite the overall consistency of results across datasets, a notable bias emerges when analyzing the very low-coverage dataset (0.0625X) filtered with GP ≥ 99%. In this case, we observed an inflated D value, indicating that stringent genotype probability filtering may distort allele sharing patterns at extremely low coverage. To address this bias, we implemented a method to compute the D-statistics directly from the GP given by imputation (Supplementary Fig. 3). For each position, with the GP of the imputed genome as ($GP_{0|0}$,$GP_{0|1}$,$GP_{1|1}$), its allele frequencies at the position is $GP_{0|0} + \frac{1}{2} GP_{0|1}$ and $GP_{1|1} + \frac{1}{2} GP_{0|1}$. The rest of the D-statistics is computed as usual. The script used can be downloaded from GitHub: https://github.com/LeoPlanche/arcAncientImputed/src/dstat.py.

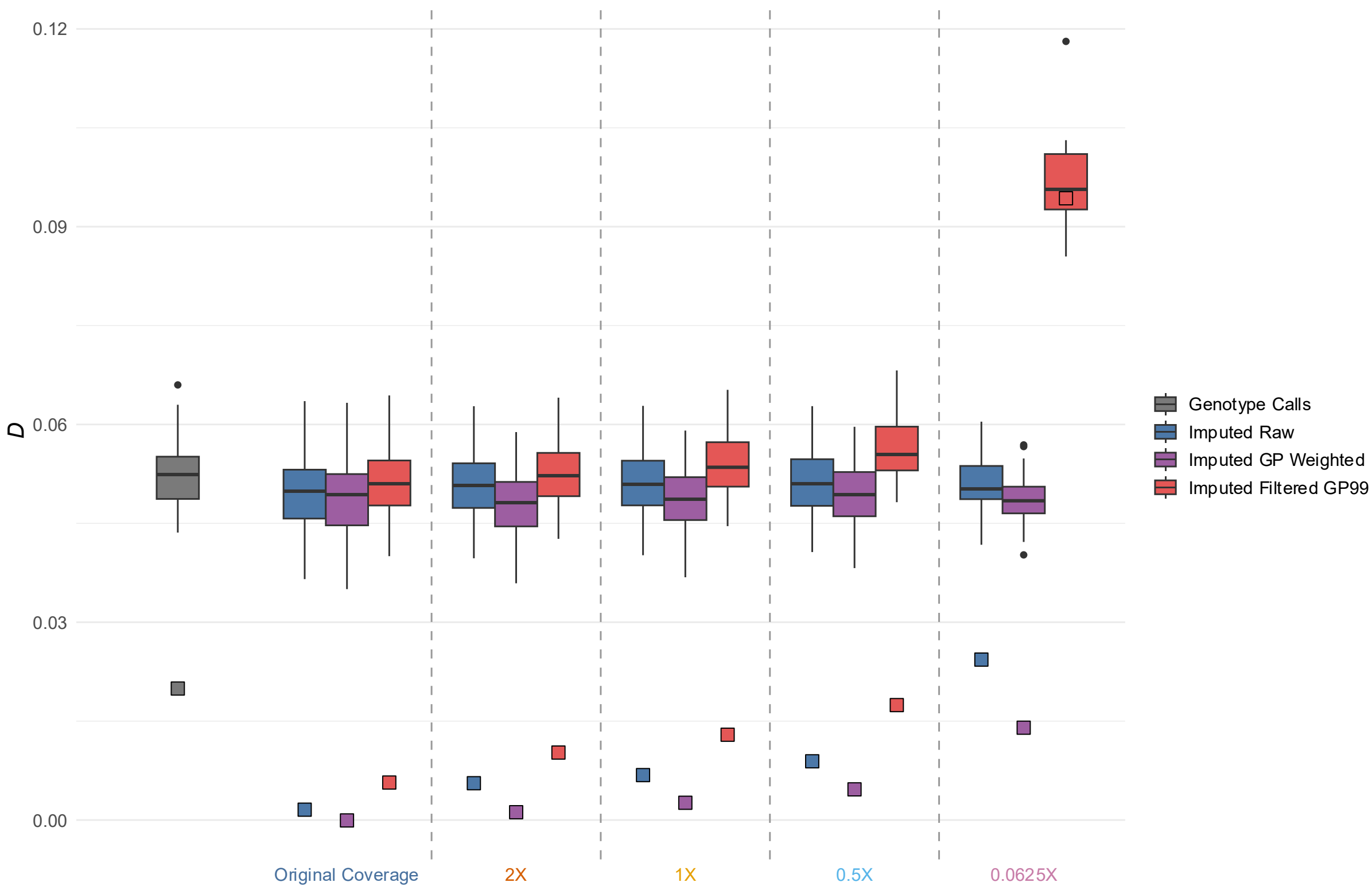

Supplementary Fig. 3. D values for the 20 individuals obtained using the new developed tool to include the Genotype Probability in the calculation of the allele frequencies. Box plots show median (center line), 25th and 75th percentiles (box bounds), and whiskers extending to 1.5X the interquartile range; individual data points beyond whiskers are shown as outliers.

To better understand the patterns that may bias the estimation of the *D*-statistic across different coverage levels, we measured the proportion of various features—such as total positions and genotype types (homozygous reference, homozygous alternative, and heterozygous)—across genotype probability (GP) bins of width 10%. In addition, we modified the RIPTA script[26] to extract the GP values associated with each ABBA and BABA site. By plotting how these patterns vary across GP bins—considering the average values across individuals and the observed variability—we found a consistent trend across coverages, with the notable exception of the lowest coverage (0.0625X) (Supplementary Fig. 16C). In general, all features show increasing proportions with higher GP values. However, at 0.0625X, both the homozygous and heterozygous alternative genotypes tend to decrease in the highest GP bin (≥ 90%), while the proportion of homozygous reference genotypes (grey) and the number of positions increase.

Although both ABBA and BABA counts decrease at high GP values, the difference between them (i.e., the numerator of the D-statistic) increases slightly, whereas the denominator—defined as the total number of ABBA and BABA sites (ABBA + BABA)—decreases (Supplementary Fig. 16C). As a result, the ratio between the two, and thus the D value, tends to increase. This suggests that, overall, ABBA sites are more likely than BABA sites to pass a stringent GP filter.

To further investigate this, we calculated the average GP for ABBA and BABA sites per individual and coverage level (Figure 3C, Source Data 2). We found that, across coverages, GP values were generally high, except at 0.0625X, where they dropped below 85%. Surprisingly, across all coverage levels, there was a consistent difference in GP between ABBAs and BABAs, with ABBA sites—representing the introgressed (archaic) positions—showing higher genotype probabilities. This difference increased as coverage decreased, reaching its maximum at 0.0625X.

These results indicate that filtering on stringent GP thresholds can systematically favour ABBA over BABA sites, introducing a bias when comparing archaic and non-archaic SNPs.

Aside from the bias observed with a very stringent GP filter, we observe that the ancient African individual Mota—included as a negative control— consistently displays outlier positions in terms of D in each dataset, with values usually, but not always, closer to zero. However, when we apply filters for GP >99%, the *D* values tend to increase. If we apply the MAF filter in the reference populations, then *D*s stay close to zero with the exception of when Mota is imputed from the lowest coverage considered here (0.0625X) (Supplementary Fig. 1B). Also the value we consider the truth, computed directly from the original genome shows a deviation from 0, even if it is still an outlier in the original genome *Ds* distribution. Statistical significance of these signals, represented in AdmixTools by the *Z-score* for each interaction, is less consistent. For example, we detect signals of introgression in Mota in some cases (Supplementary Fig. 1), and this may be due to the fact that Mota may actually have some archaic ancestry from back migrations from Europe or because imputation does not work as well in African individuals. In a previous study, it was shown that this African genome had the second lowest imputation accuracy among the ancient high-coverage genomes that were downsampled and imputed[19]. Also, in this case, we observe signals of introgression in Mota using the original data, using only transversion we do not see this signal anymore (Source Data 1). For all other interactions, the Z-score remains below |3.3|, allowing us to confidently exclude the presence of introgression in those individuals.

f4-ratio

For global ancestry inference, specifically to estimate the proportion of archaic introgression in each individual genome, we applied the f4-ratio method from the AdmixTools package[25]. The level of Neanderthal introgression was estimated using the f4-ratio in the form: f4(Altai, Chimp; Test Individual, Africa) / f4(Altai, Chimp; Vindija, Africa). Across all coverage levels (Figure 1D, Supplementary Fig. 4, Source Data 3), we observed that applying a GP ≥ 99% filter tended to increase the estimated archaic proportion. For instance, while the average proportion in the original genome, imputed raw and pseudohaploid transversion data is around 2.5%, the estimates increase with stricter filtering in the imputed data. This effect is particularly evident as coverage decreases. For the 0.0625X dataset, the distributions of f4-ratio estimates with and without GP filtering showed minimal overlap, highlighting a clear discrepancy introduced by stringent filters at very low coverage.

To assess whether the GAI correlates with individual-specific features and is independent of data processing variables, we tested correlations between α (f4-ratio values) and: i) the age of each ancient individual (in thousands of years BCE) and the original sequencing coverage (before downsampling).
For this analysis, the Mota individual was excluded.

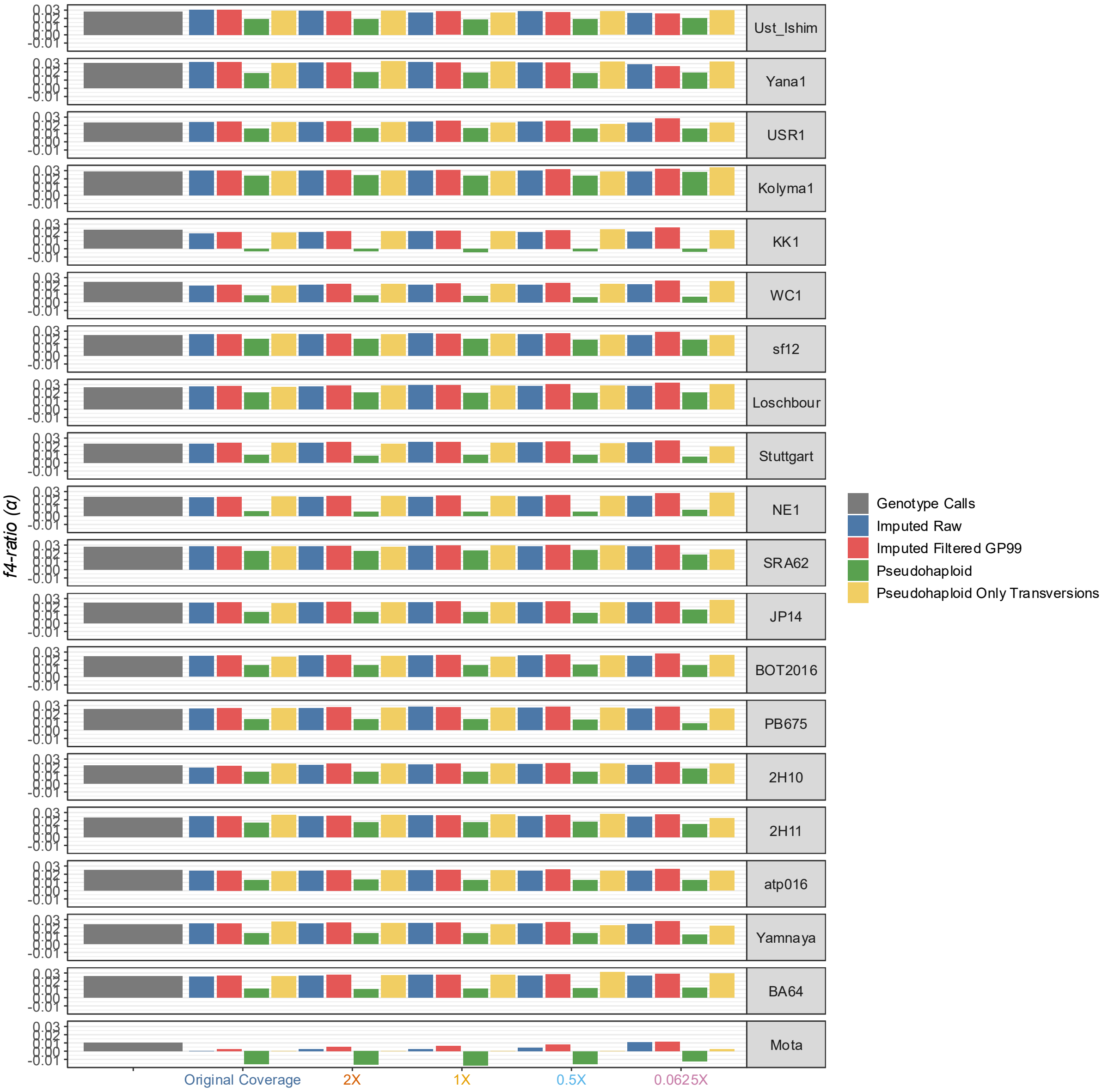

Supplementary Fig. 4. Barplot of f4-ratio, as in Figure 1D facet per individual.

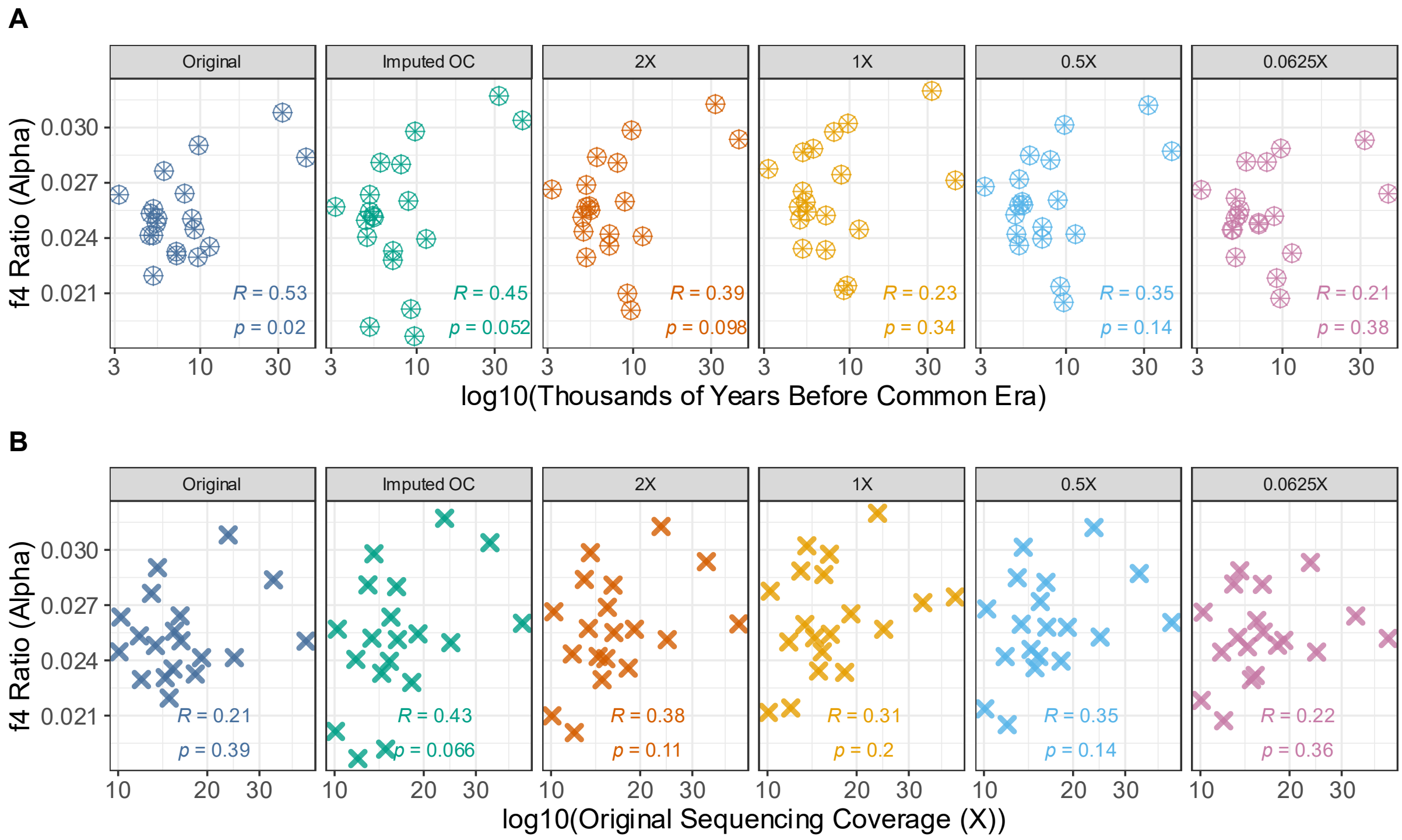

Supplementary Fig. 5. Relationship between the archaic proportion (α) and A) The age of the ancient individual (n=19) in Thousands years before common era, B) Coverage of the original sequencing coverage. For each of the comparisons the 6 different datasets have been analysed independently. The Pearson correlation coefficients are annotated on each plot. For this analysis, the Mota individual was excluded.

3. Local archaic Ancestry Inference

For archaic local ancestry inference we use our custom version of hmmix[27] that is a reference free method, meaning that it does not require archaic genomes as references, but it does require an outgroup composed of individuals without archaic ancestry. We used the same unadmixed outgroup of 292 African genomes from the 1000 Genomes Project (Supplementary Data 2) used in the allele frequencies analyses. This analysis was performed individually for each genome across all six datasets analyzed in this study—one corresponding to the original dataset and five to the imputed datasets. For the imputed data, we used unphased genotypes filtered for GP ≥ 99%. The resulting introgression maps, which include similarity measures, distances to archaic reference genomes, and the number of private archaic positions (Vindija Neanderthal and Denisova3; see below), are available on Zenodo (DOI: 10.5281/zenodo.16365479) and at https://github.com/LeoPlanche/arcAncientImputed/maps.

For Hidden Markov Models (HMMs) methods, missing information in one part of the genome does not affect the quality of inference in other distant regions with better coverage, missing data at one position affects inference only locally. This allows for the detection of archaic segments even in cases of very low-coverage data where there is a higher proportion of missing positions overall.

To estimate the proportion of the genome identified as introgressed, we summed the total length of introgressed segments, without any filters, for each individual and divided it by the length of the hg19 reference genome (Figure 2A). The amount of genome identified as introgressed depends on the downsampled coverage, with higher coverage resulting in a greater number of detected segments (Supplementary Fig. 6). Overall, we observed a probable overestimation of the proportion of introgressed genomes in some cases, which we then fixed using filters (see "Filtering of introgressed segments") based on similarity to the archaic genome. After filtering, Figure 2A shows a decreasing trend from older to younger individuals (right to left), as expected, alongside inter-individual fluctuation. For example, BOT2016 retains a higher proportion of introgressed sequence than temporally proximate individuals. Such variability is expected and mirrors inter-individual differences observed in present-day populations[28].

To assess the recovery of introgressed regions in the imputed data, we compared the segments identified in the original non imputed dataset to those in each imputed dataset. We say that a segment found in the original dataset has also been found in an imputed dataset, if at least 1bp of the segment overlaps a segment found with the imputed dataset. Across all individuals, we found that 100%, 99%, 99%, 97%, and 50% of the segments identified in the original data were also detected in the imputed original coverage, 2X, 1X, 0.5X, and 0.0625X datasets, respectively (Figure 2B).

Remarkably, even with the very low-coverage (0.0625X) imputed genomes, we were still able to recover half of the introgressed segments originally identified in the original data.

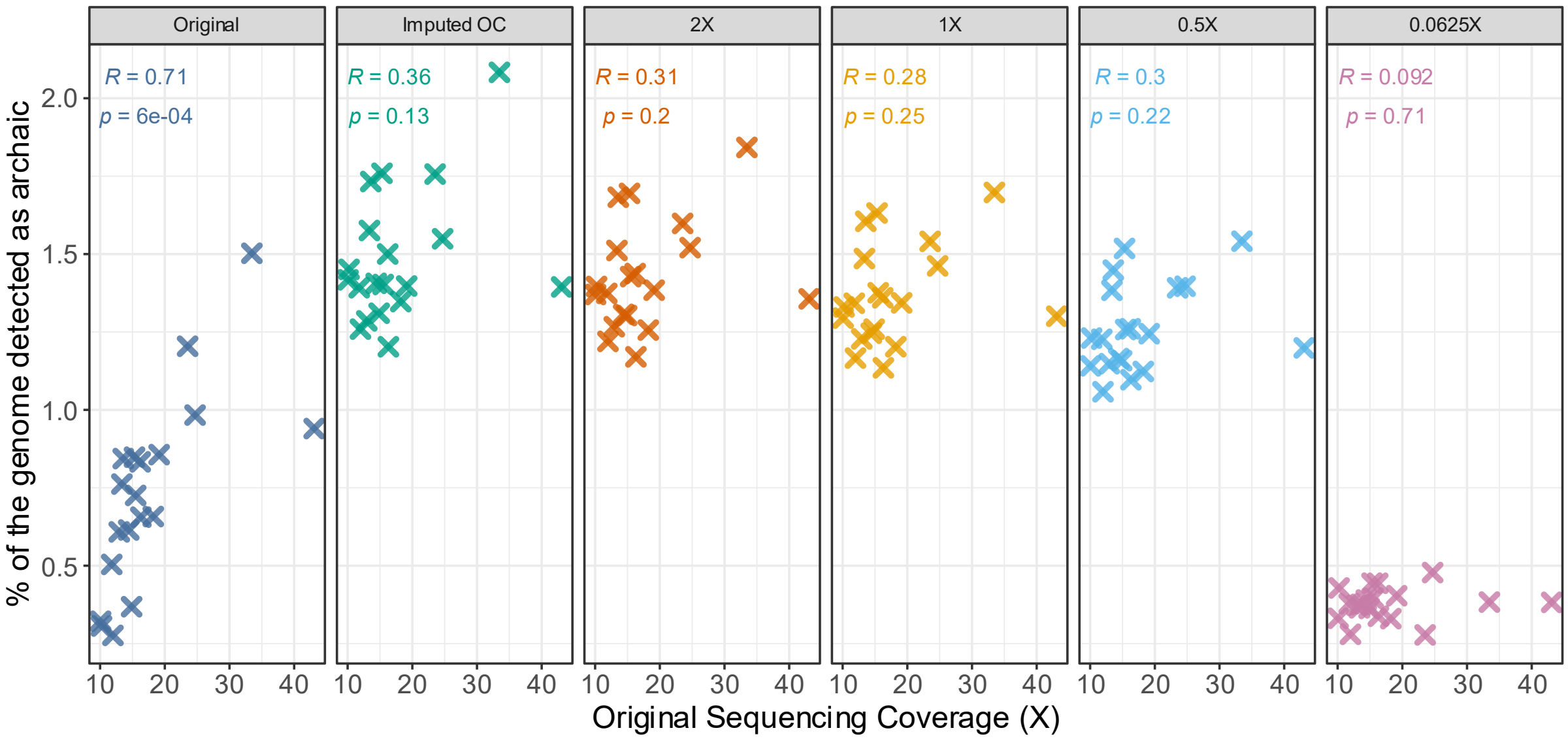

Supplementary Fig. 6. Correlation between the amount of archaic segments inferred for each individual (n=19) in each dataset and its coverage. The Pearson correlation coefficients are annotated on each plot. For this analysis, the Mota individual was excluded.

4. Local archaic Ancestry Inference on 1240K SNP-capture data.

Since many ancient genomes were generated with the 1240k SNP-capture panel, we asked whether imputation can also improve detection of archaic introgression for captured data. Prior works indicate that (i) imputation from capture data is generally less accurate than from shotgun sequencing[29], (ii) the 1240k panel is comparatively uninformative for archaic-introgression analyses[30], and (iii) capture and shotgun can differ due to allele-specific/ascertainment biases. To assess feasibility, we leveraged the only individual in our dataset with both shotgun and capture data (Stuttgart). We downsampled the capture data to match our shotgun down-coverages (2X, 1X, 0.5X, 0.0625X), imputed genotypes at 1000 Genomes biallelic SNPs, filtered for GP ≥ 0.99, and ran hmmix as described above, to obtain introgression maps. We then compared these maps to those from the original and imputed shotgun data (Supplementary Fig. 7-8). Imputed capture data recovered a substantial fraction of archaic segments when capture coverage was ~2X or higher, approaching the segments obtained from the original data; at ≤ 1X, recovery dropped markedly. As expected, notable differences remain between shotgun- and capture-derived maps.

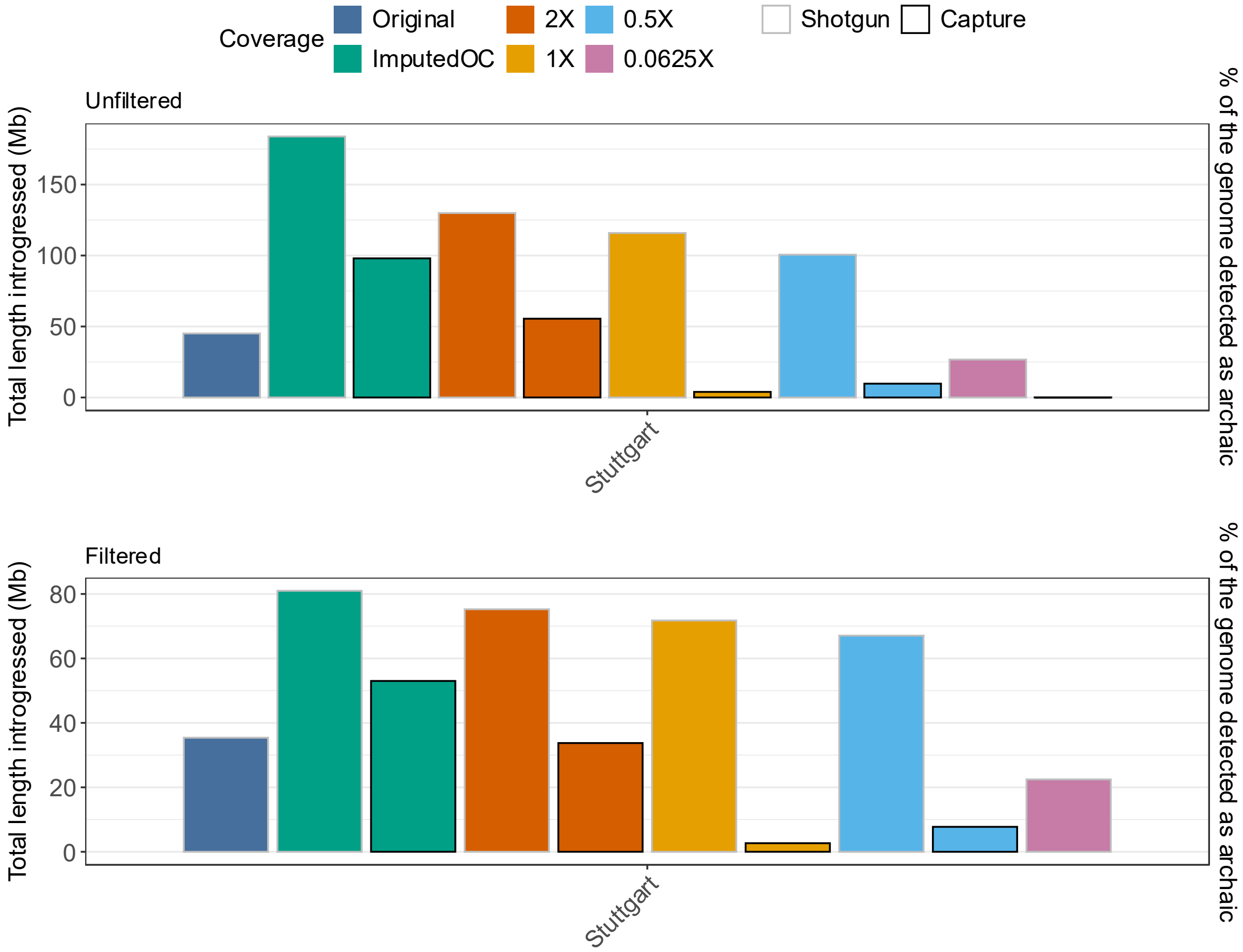

Supplementary Fig. 7. Total length of introgressed segments per dataset, reported in Megabases (Mb; left axis) and as the percentage of the genome detected as archaic (right axis). The top panel shows unfiltered results; the bottom panel shows results after filtering for segment length > 40 kb and similarity to the archaic reference genome ≥ 0.99. Bars with black outlines indicate 1240k capture data.

We also quantified how much of the original (shotgun) signal can be recovered from imputed capture genotypes (Supplementary Fig. 8). With capture coverage of ~2X or higher, imputation recovers ≥75% of the segments identified in the original data. In contrast, recovery drops sharply when coverage is < 2X.

Overall, while shotgun data confer a clear advantage for inferring archaic segments, capture data can still be used to identify archaic introgression, provided coverage is sufficient to support reliable genome-wide imputation.

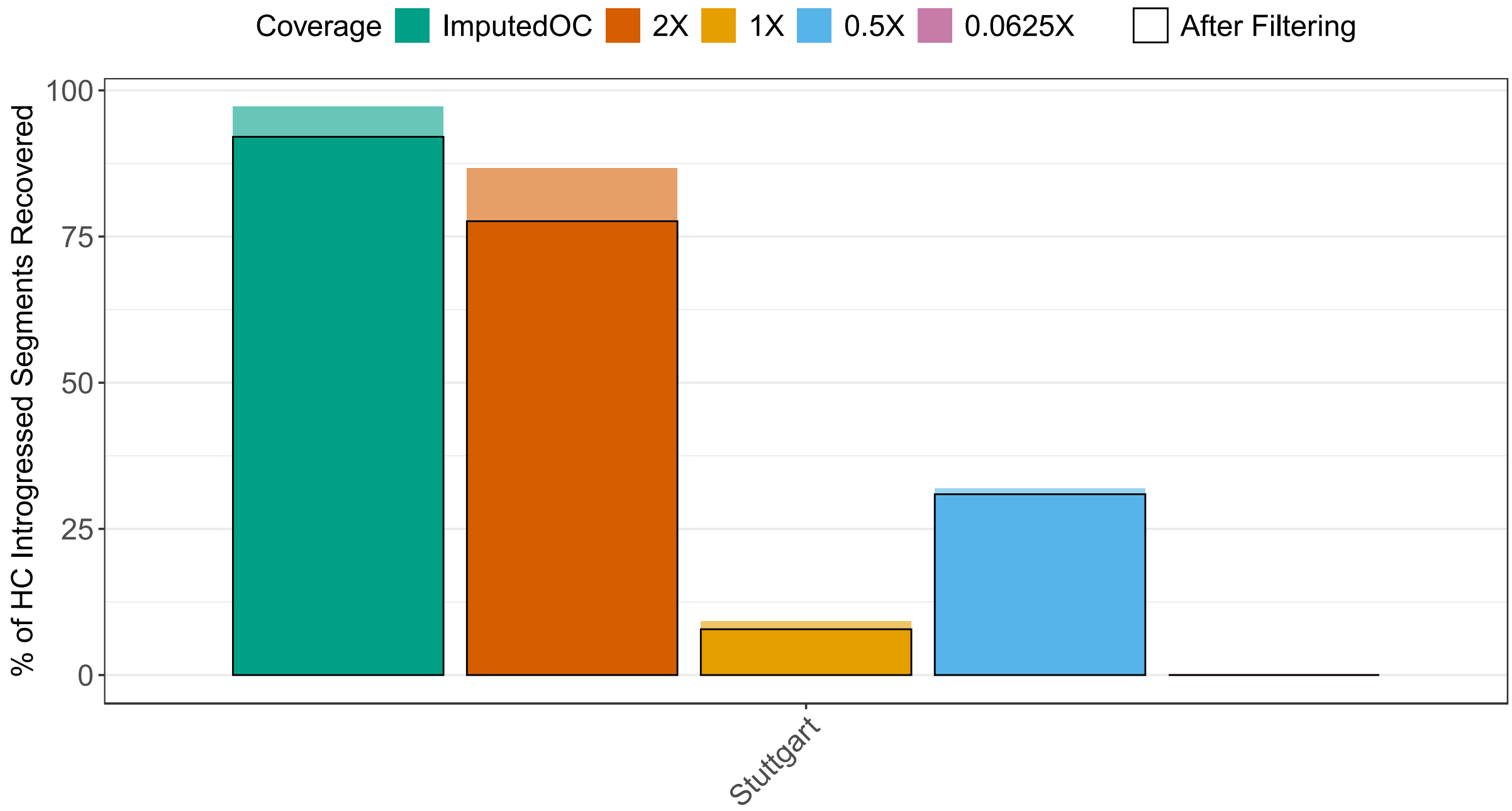

Supplementary Fig. 8. Proportions of the archaic segments detected using the High Coverage genome, that are also detected using capture imputed data.

5. Similarity and distance to Archaic genomes

To evaluate the segments detected as archaic and non archaic for all datasets, we first compute their similarity to archaic reference genomes (Vindija Neanderthal and Denisova3). The similarity for a segment is computed as such: we look at all the variant positions within the segment present in the archaic individuals and in the individual's original genome VCF. Then, there is a match if the archaic and the individual's original genome share an allele at this position. The similarity is the proportion of matches along all the variant positions in the intersection of the archaic humans and the individual original genome . More precisely, let a_1, a_2 be archaic reference haplotypes and s_1, s_2- original individual haplotypes. Define the following sets of variable positions for a segment:

$$S = \{\text{position } i : \exists k, j : a_k[i] = s_j[i]\} \quad (1)$$
$$\underline{S} = \{\text{position } i : \forall k, j : a_k[i] \neq s_j[i]\} \quad (2)$$

Then similarity is simply the ratio:

$$\text{similarity} = \frac{|S|}{|S|+|\underline{S}|} \quad (3)$$

By comparing the similarity of the original genomes in the regions identified as introgressed in the imputed genomes, we mitigate the potential bias introduced by imputation and confirm that the identified archaic segments exhibit significantly higher similarity compared to the non-archaic ones (Figure 2C). Additionally, we classified the identified archaic segments into two groups: Archaic Shared for segments identified in both the imputed and original data (with overlap between the two segments), and Archaic New for segments identified only in the imputed data. Using this approach, we observed a higher similarity to the nearest archaic genome in the Archaic Shared segments compared to the Archaic New segments, which, however, still show significantly greater similarity to the archaic genome than the non-archaic segments (Figure 2C).

To compare the distributions of similarity to the nearest archaic genome between segments labelled Archaic and Non-Archaic, we treated the individual as the unit of analysis to avoid pseudo replication from multiple segments per person. For each individual and coverage stratum, we computed the per-individual median similarity separately for Archaic and Non-Archaic segments, then formed a paired difference:

$$D_i = \text{median}_i(\text{Archaic}) - \text{median}_i(\text{Non-Archaic}) \quad (4)$$

We tested H_0 : typical shift = 0 using a Wilcoxon signed-rank test (two-sided), reporting the Hodges–Lehmann (HL) shift and its 95% CI. Because the signed-rank test ignores zero differences, we also report the number of informative pairs (non-zero D_i). As an effect size we provide the paired rank-biserial correlation (r_{rb}, range [−1,1]). P-values were adjusted across coverage strata using the Benjamini–Hochberg FDR (Source Data 1).

We then also define a normalized version of the similarity. For a given segment, we compute the similarity as defined above between the individual's original haplotype and the archaic reference haplotypes, but also the similarity between individuals from Biaka in the HGDP dataset and the archaic reference haplotypes. Finally we compute the normalized distance as:

$$\text{distance} = \frac{1-similarity}{1-average\_similarity\_hgdp} \quad (5)$$

This approach offers a more principled way to post-processing classification of segments, returning a normalized distance between each ancient individual and the archaic reference genomes (Supplementary Fig. 9).

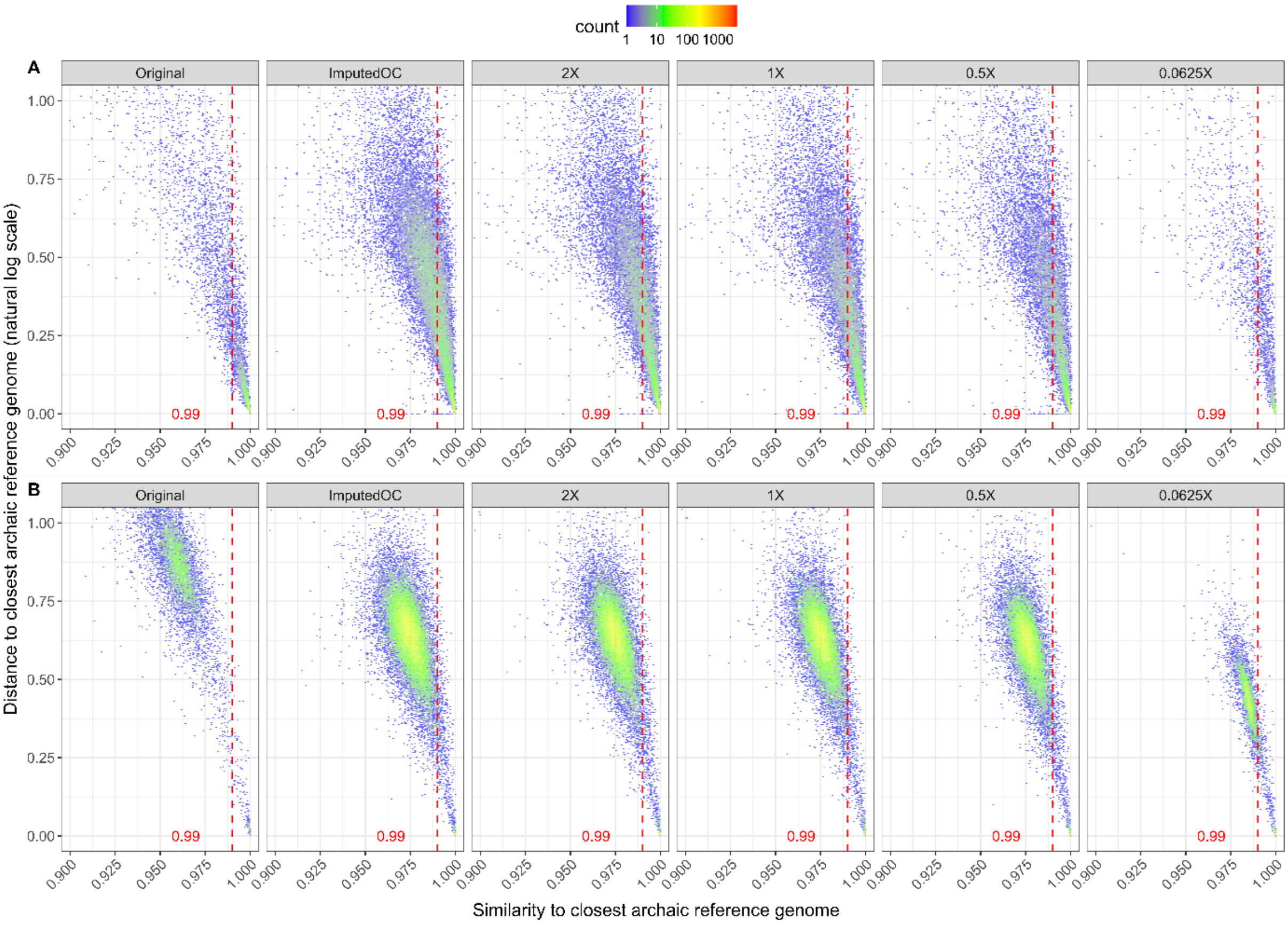

Supplementary Fig. 9. Density plots show the relationship between similarity to the closest archaic reference genome (x-axis) and distance to the closest archaic reference genome (log-transformed, y-axis) for (A) archaic segments and (B) non-archaic segments. Each hexagon's color reflects the log-scaled number of segments per bin. The vertical dashed red line marks the 0.99 similarity threshold.

We also measured the similarity between the imputed genomes themselves (in the regions identified as archaic) and the archaic genomes. This allows us to assess how closely the actual imputed data used to build the introgression maps resemble the archaic references. Rather than solely confirming the absence of bias introduced by imputation, this similarity measure serves as a valuable metric for evaluating the quality of archaic segment calls. It can be extended to larger datasets of ancient individuals, beyond artificially downsampled genomes.

Finally, to distinguish between Neanderthal and Denisovan segments, we computed the number of archaic private variants present in each segment. An archaic private variant is defined as a variant present in the individual, absent in the African outgroup and present in the archaic individual. More precisely, let s_1, s_2 be individual haplotypes, let *o[i]* be the merged outgroup genotype at position *i*. Define the following sets of variable positions for a segment:

$$O = \{ \text{position } i : \exists k : s_k[i] \notin o[i] \} \quad (6)$$

Note that this corresponds to the set of positions used as observations for *hmmix*. Let a_1, a_2 be archaic reference haplotypes, then the set of archaic private variant for a segment is:

$$S = \{ \text{position } i \in O : \exists k, j : s_k[i] = a_j[i] \} \quad (7)$$

By comparing the number of Denisovan private variants to the number of Neanderthal private variants we can classify a segment as having most like Denisovan or Neanderthal origin.

6. Filtering of introgressed segments

To reduce the possible bias/false positives and to keep only the candidate segments identified as introgressed in hmmix that have strong features of introgression, we proposed filters on the segments. We kept segments with a similarity to the closest ancient archaic genome (Vindija for Neanderthal and Denisova3 for Denisova) above 0.99 and with a length above 40Kb; that is above the length needed to reject incomplete lineage sorting for regions with moderate recombination rates[31]. With these filters we obtained results that are consistently close to the proportion measured with the f4-ratio (2-3%), for all coverages and individuals down to 0.5X (Supplementary Fig. 10), and in line with expectations, with the most ancient individual (on the left in Figure 2A) showing the highest proportion of archaic ancestry. Even with these quality filters the amount of introgressed segments that can be identified using imputed genomes is much higher than with the original non imputed data, and the variation among the different imputed coverage, with the exception of 0.0625X, is more stable (Supplementary Fig. 6).

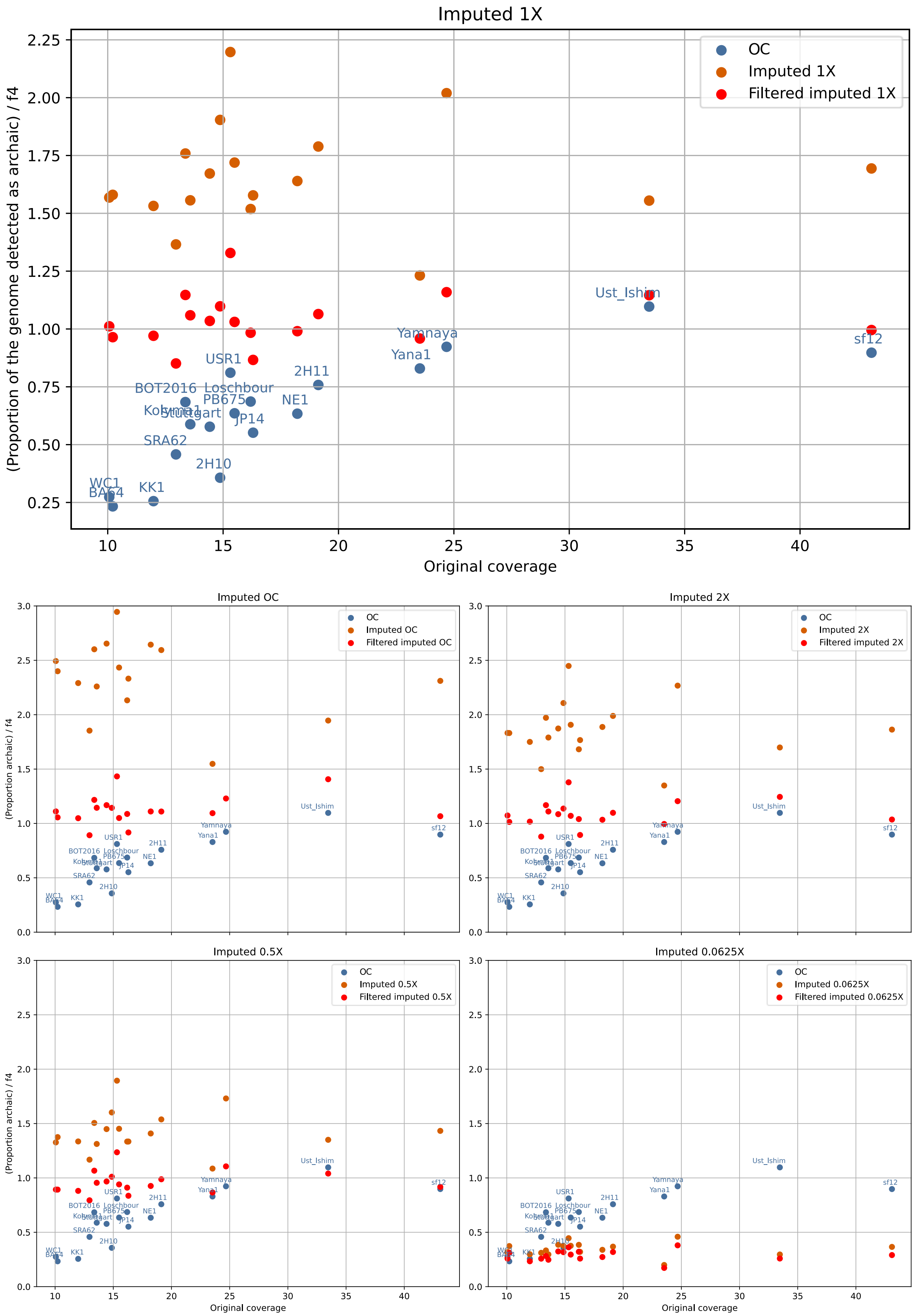

Supplementary Fig. 10. Filtering false positive segments. For each individual (n=20) and coverage, we compute the proportion of the genome detected as archaic by summing the length of segments inferred as archaic and dividing by total genome length of 3,000,000,000. We then divide this proportion by the f4

ratio computed for the same individual. As both these values should correspond to the proportion of archaic ancestry, we expect the ratio to be 1. For the non imputed genomes (blue dots), we see that this ratio depends on the original coverage of the individual, with coverage below 20X, the LAI method does not infer all archaic segments and the ratio is below 1. For imputed data (red dots), the ratio is above one for most individuals, except for imputed 0.0625X, hinting at the presence of false positives. After applying filters of similarity to archaic genome greater than 0.99 and minimum segment length of 40kb (orange dots), we see that the ratio is close to one - except for imputed 0.0625X - for all individuals, independently of the original coverage,, showing that imputation can improve the inference of archaic segments even when the original coverage is already considered good for ancient DNA, i.e 10-20X. For imputed 0.0625X, we get after filtering an average ratio of 0.29, showing that we recover a significant portion of archaic segments even at such low coverages.

7. Identification of Denisovan origin introgressed segments

We then assessed the potential of imputation to aid in the detection of Denisovan segments. This task is particularly challenging in ancient genomes when using a model that relies solely on the available high-coverage Denisovan genome[30], especially given the complex history of multiple Denisovan population introgression events[32].

We expect to identify Denisovan segments in the few individuals among those analyzed here who are not from West Eurasia or Africa. A segment was classified as Denisovan if it met five criteria: (i) it was detected as archaic, (ii) had a length above 100kb, (iii) had a similarity of at least 0.99 to one of the high coverage archaic genome, (iv) had a at least 10 private Denisovan variants and (v) had more Denisovan private variants than Neanderthal private variants.

This very conservative filtering approach ensures high confidence in Denisovan segment identification, as our objective is to analyze the performance of imputation on genomic regions that can be reliably classified as Denisovan.

When analyzing the introgressed Denisovan segments inferred from the original dataset, we find, as expected, the highest number of candidate Denisovan segments in USR1 (Alaska) and Kolyma1 (Siberia) (Figure 2D, Supplementary Fig. 11). This is consistent with the known ancestral history of these individuals, who, in addition to the Ancient North Siberian (ANS) ancestry, also have East Asian contributions[14]. In our dataset, Yana1 represents ANS ancestry[14] and shows a limited number of Denisovan segments (Figure 2D).

We then compared the Denisovan introgressed segments we identified in ancient genomes with two introgression maps for contemporary individuals: one generated using hmmix[27], and the other using ArchaicSeeker[33]. Almost all Denisovan segments, detected in Kolyma1, depending on the coverage dataset, were also identified in Skov et al. 2018[27] and Yuan et al 2021[33], whereas only ~50% of the segments found in USR1 matched those in Skov et al. 2018[27] and and higher match ~70% in Yuan et al 2021[33].

We then verified, as before, whether the introgressed segments obtained using imputed genotypes contained the Denisovan segments identified in the original dataset, as well as additional ones. Expanding the Archaic Shared and Archaic New categories to Denisovan Shared and Denisovan New.

Using imputed genotypes, we not only identified the segments present in the original dataset (at least down to 0.5X coverage) but also detected additional Denisovan segments (Figure 2D, Supplementary Fig. 11). Most of these newly identified segments were also present in introgression maps based on present-day individuals. However, a small excess of segments was identified exclusively in the ancient DNA and was absent from both the original dataset and the Skov et al.[27] introgression maps, which are based on 1000 Genomes Project individuals—the same dataset used for imputation.

These results demonstrate that imputation enhances the detection of Denisovan segments. It not only helps identify segments that persist in contemporary individuals but also enables the reconstruction of segments that were introgressed in ancient individuals but are no longer present in the present-day reference panel. Although the analysis was done on a much smaller set of segments compared to all archaic and might require further investigation.

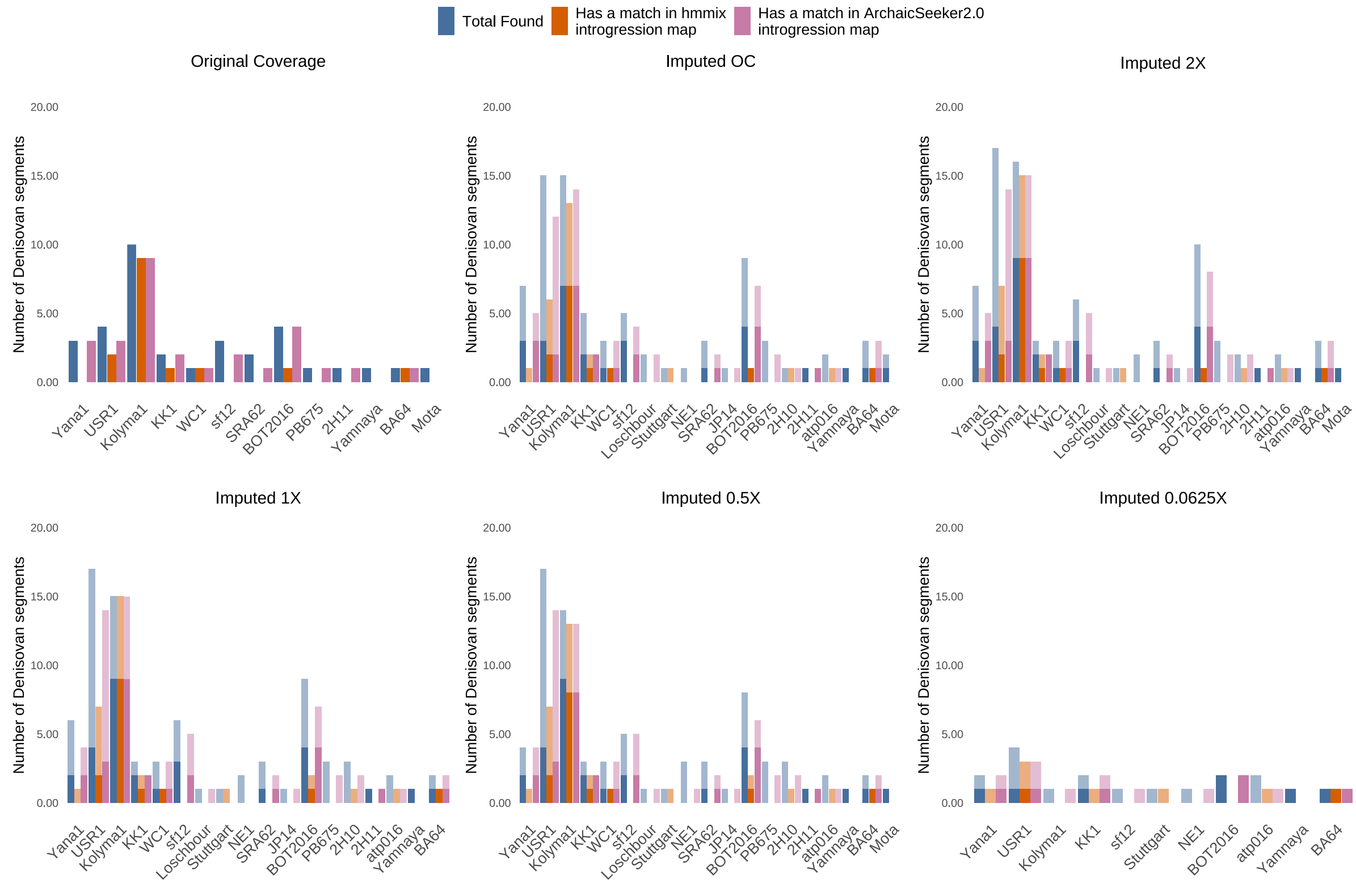

Supplementary Fig. 11. Number of Denisovan segments found in each dataset. We apply more stringent filters to classify a segment as Denisovan: minimum length of 100,000bp, similarity to archaic above 0.99, at least 10 Denisovan private variants and more Denisovan private variants than Neanderthal private variants. An archaic private variant is defined as a variant present in the individual, absent in the African outgroup and present in the archaic individual. In blue the total number of denisovan segments found, in orange the amount of these segments also found in the hmmix introgression map, in pink the amount found in ArchaicSeeker 2.0 map. The light shade corresponds to Archaic New segments, the dark shade, Archaic Shared.

8. Summary of introgression in present-day individuals based on Skov et al. maps

To enable a direct comparison between ancient and present-day groups, we summarized introgression maps for present-day individuals from the 1000 Genomes Project (1000G) and the HGDP, generated with the same hmmix-based approach as in Skov et al.[27]. For both datasets, we retained segments with mean_prob ≥ *0.8*. Segment length was computed as END − START using the map coordinates as provided. For each individual, total introgressed length was obtained by summing segment lengths across autosomes.

We then quantified the proportion of the genome inferred as introgressed for each individual as:

$$introgressed\ proportion\ = \frac{Total\ length\ per\ individual}{2\ X\ Length\ autosomes,GRCh38} \qquad (8)$$

Using the Ensembl GRCh38, and the factor of 2 accounts for diploidy. The resulting distributions of individual-level proportions are summarized by region as reported in the maps (Supplementary Fig. 12, Source Data 7).

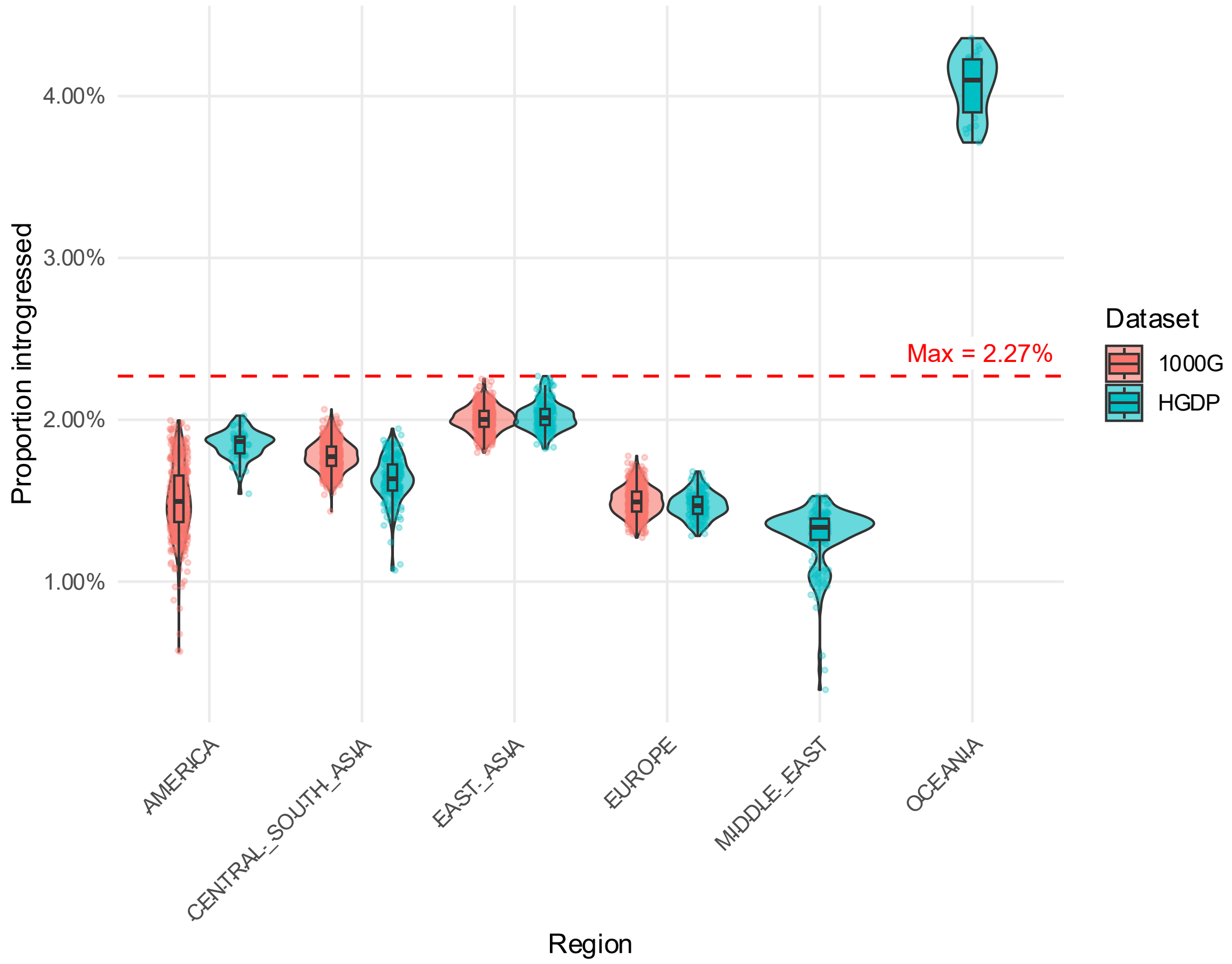

Supplementary Fig. 12. Distribution of introgressed genome proportion by geographic region for HGDP and 1000G based on the introgression maps published by Skov et al.[27] Violin plots show individual-level proportions; a red dashed line marks the global maximum outside Oceania. Proportions were computed as total introgressed length (sum of end − start across segments passing mean_prob ≥ 0.8) divided by 2 times Ensembl GRCh38 autosomal length. For each Region the number of individuals analysed is reported in Source Data 7.

To specifically assess Denisovan ancestry, we used the *ND_type* annotation provided in the maps, which indicates the archaic reference (Neanderthal, Denisova or both) sharing the largest number of derived alleles with each segment. We restricted to segments labeled *ND_type = "Denisova"* and repeated the

summary described above to obtain regional distributions of Denisovan introgression (Supplementary Fig. 13, Source Data 7).

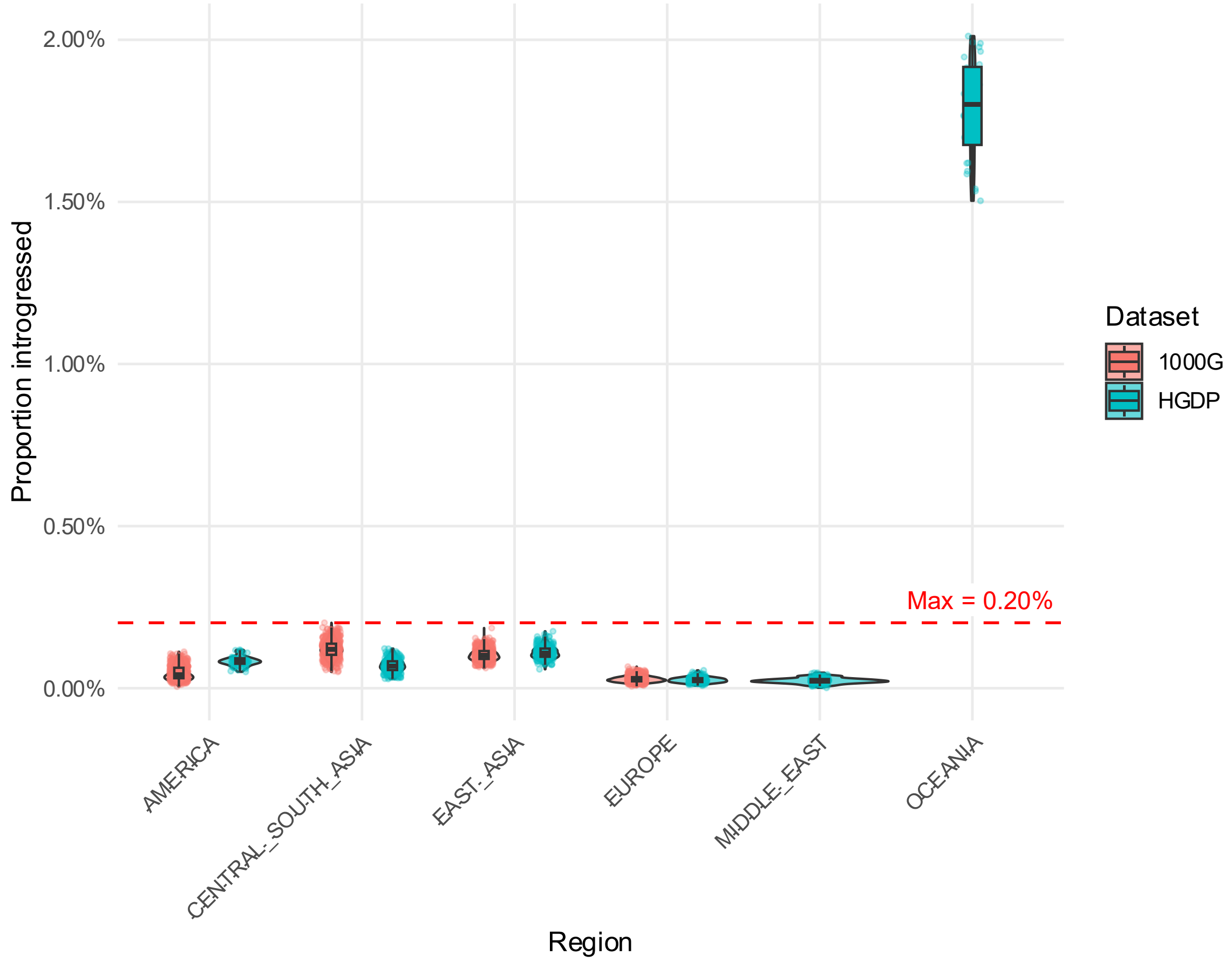

Supplementary Fig. 13. Distribution of Denisovan introgressed genome proportion by geographic region for HGDP and 1000G based on the introgression maps published by Skov et al.[27] Violin plots show individual-level proportions; a red dashed line marks the global maximum outside Oceania. Proportions were computed as total introgressed length (sum of end − start across segments passing mean_prob ≥ 0.8) divided by 2 times Ensembl GRCh38 autosomal length. For each Region the number of individuals analysed is reported in Source Data 7.

Summary statistics of overall and Denisovan introgression proportions by geographic region are shown in Source Data 7.

9. Quality of the introgressed segments

Supplementary Table 1. Extrapolation of data from Table 1 to highlight information derived from the original dataset.

Segment Classification	Allele	Imputed OC	2X	1X	0.5X	0.0625X
Non Archaic	Missing (".")	180299	156408	138211	118909	27615
		55.65%	55.50%	55.26%	55.21%	57.48%
Non Archaic	Non Archaic ("0")	143262	124985	111584	96134	20371
		44.22%	44.35%	44.61%	44.63%	42.40%
Non Archaic	Archaic ("1")	435	441	325	339	58
		0.13%	0.16%	0.13%	0.16%	0.12%
Archaic Shared	Missing (".")	74217	78281	77613	74236	27451
		50.77%	51.79%	51.96%	52.07%	52.66%
Archaic Shared	Non Archaic ("0")	8581	7881	7059	5916	804
		5.87%	5.21%	4.73%	4.15%	1.54%
Archaic Shared	Archaic ("1")	63380	64989	64712	62423	23869
		43.36%	43.00%	43.32%	43.78%	45.79%
Archaic New	Missing (".")	31932	37676	36648	33722	7698
		63.24%	64.70%	65.01%	65.55%	69.89%
Archaic New	Non Archaic ("0")	4676	4790	4305	3692	470
		9.26%	8.23%	7.64%	7.18%	4.27%
Archaic New	Archaic ("1")	13889	15769	15419	14033	2846
		27.50%	27.08%	27.35%	27.28%	25.84%

Length

The length of introgressed segments is expected to correlate with the age of the individuals, with older individuals having longer segments, as they are closer to the original admixture events[32,34]. When examining segment length, we observe that imputation has a deleterious effect, leading to ancient humans having values of length closest to what is expected in contemporary humans, i.e. 100,000-120,000bp for an introgression time 2400-2000 generations ago (Supplementary Fig. 14). Specifically, the two oldest individuals in our dataset, Ust'Ishim and Yana1, exhibit longer average segment lengths in the original dataset, +150,000bp and +74,000bp respectively compared to the more recent individuals. However, this trend diminishes in both Ust'Ishim and Yana1 when using imputed data (Supplementary Fig. 14). For individuals closer to the present, the differences in length between non imputed and imputed

genomes is more similar, only differing by on average +9,722bp for Imputed OC, to -13,384bp for imputed 0.0625X.

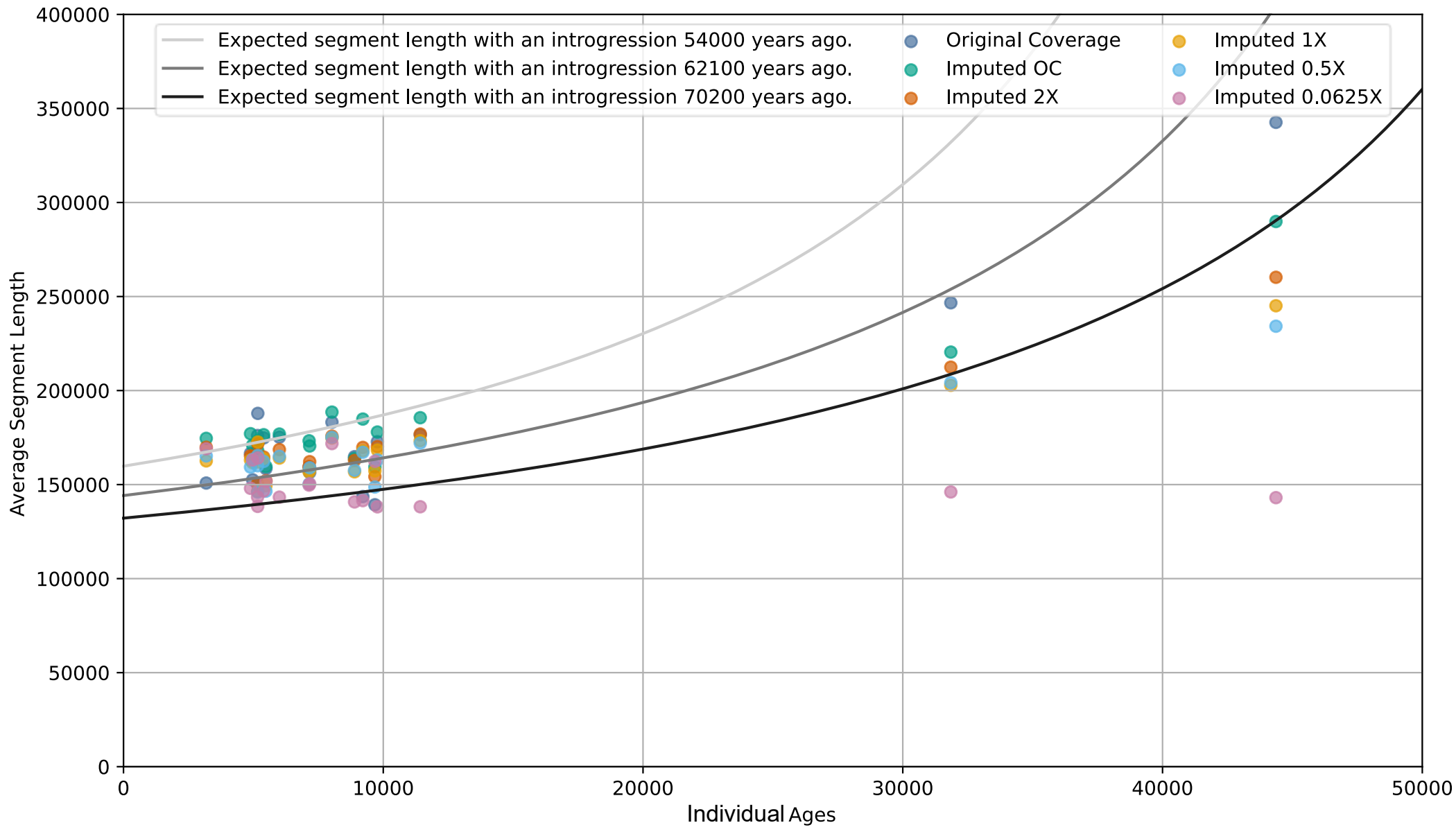

Supplementary Fig. 14. Average archaic segment length depending on the dataset used. For segments with an age below 12,000 years (all individuals but Yana1 and Ust Ishim), the average difference in length between the original coverage and imputed OC, imputed 2X, imputed 1X, imputed 0.5X and imputed 0.0625X segments are respectively +9,722bp, +988bp, -1,711bp, -3,635bp and -13,384bp. For Yana1 (31,850 years old), the differences are respectively -26,222bp, -34,241bp, -43,863 bp, -42,482bp and -100,586bp. For Ust Ishim (44,366 years old), the differences are respectively -52,731bp, -82,396bp, -97,524bp, -108,406bp and -199,563bp. For purely illustrative purposes, we give the expected segment length for three different introgression times, considering a constant recombination rate of $2.3*10^{-8}$, 27 years per generation and conditioned on a minimum segment length of 40kbp, corresponding to the filter we apply to the inferred archaic segments.

Considering the two groups, Archaic Shared and Archaic New, we observed that the decrease in segment length is more pronounced in the Archaic New group, while the Archaic Shared segments tend to be even longer (starting from 0.5X and above) compared to the original data. The Archaic New segments make up the largest portion and are generally slightly shorter than the shared ones. To investigate whether this difference in segment length is associated with similarity to the archaic genome, we analyzed the closest archaic match (Vindija Neanderthal or Denisovan3) for each segment. Even in this case, the length and similarity distributions of the Archaic Shared and Archaic New categories remain quite similar (Supplementary Fig. 15B). We can observe that the Imputed OC has longer segments compared to the other imputed datasets. These longer segments often are archaic segments which are wrongly "extended" in length, also overlapping a non archaic part of the genome, which decreases the similarity compared to the other imputed datasets. This can produce a loss of true positive segments after filtering.

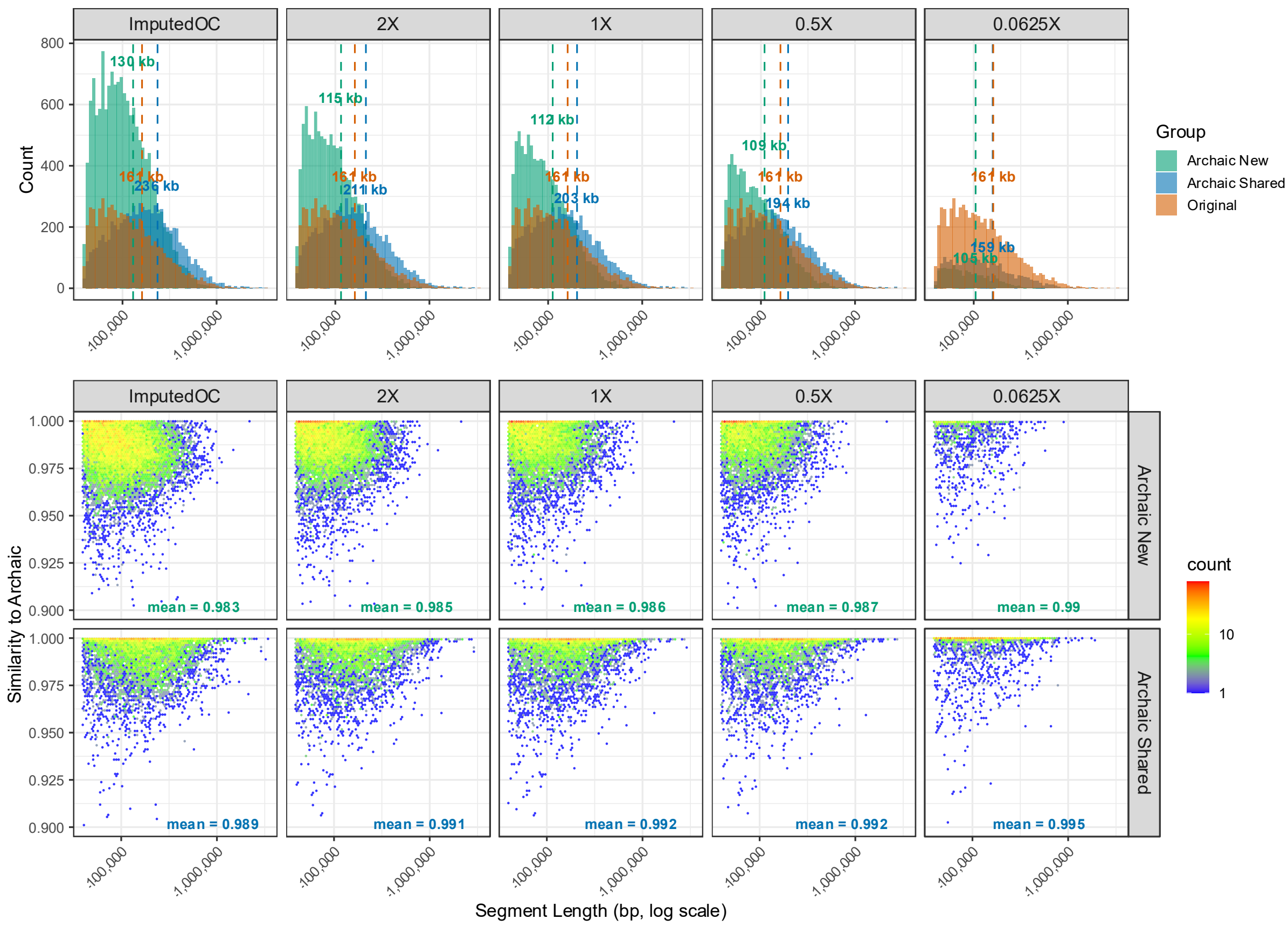

Supplementary Fig. 15. Comparison of archaic segment length distribution and similarity to archaic reference genomes across imputed and original coverage levels. A) Histogram of archaic segment lengths (log-scaled) across different imputed coverages. Each imputed group is compared against overlaid distributions of original coverage segments. Dashed vertical lines indicate the mean segment length per group, with corresponding mean values annotated. B) Hexbin density plots showing the relationship between segment length (log-scaled) and similarity to the closest archaic genome for each imputed coverage, separately for Archaic New (top) and Archaic Shared (bottom) segments. Mean similarity values per group are shown within each facet.

10. Accuracy of archaic alleles vs MAF

We assessed both how well imputation recovers archaic variants and how well it recovers archaic segments. The main question of interest is the ability of imputation to recover archaic segments, but the location of those segments is unknown and using the results of LAI methods in that context creates obvious biases. On the other hand, we are able to define archaic variants independently of the ancient individuals, eliminating such issues. A variant is defined as archaic if it is absent in African populations (Supplementary Data 2) and homozygous in Vindija Neanderthal. Archaic SNPs are positions carrying these variants.

For each individual, we compared archaic positions between non imputed and imputed genomes (Table 1) in function of their state: non-archaic, Archaic New or Archaic Shared. In order for the raw numbers to be comparable, for non archaic, we picked a set of random non-archaic segments chosen to have the same length distribution as the archaic segments (Supplementary Table 2). We then looked at every SNPs in the genome and computed their imputation accuracy depending on if they were archaic (as defined above) or not (Figure 3A). To do so, we directly use GP in the imputed genome and define a genotype between 0 and 1 as $GP_{1|1} + \frac{1}{2} GP_{0|1}$ and compute the mean square error to the original genotype at that position (Source Data 2). We then plot 1-MSE, which is equivalent to r² correlation.

The number of positions analysed in each bin is reported in the table below

Supplementary Table 2: Number of SNPs for each MAF bin for the Non-Archaic and Archaic categories used for Figure 3A.

Bins	Non Archaic SNPs	Archaic SNPs
**[0.0,0.02]**	30396	24882
**[0.02,0.04]**	16741	23004
**[0.04,0.06]**	17945	16134
**[0.06,0.08]**	19757	10340
**[0.08,0.1]**	24004	7060
**[0.1,0.12]**	26698	5537
**[0.12,0.14]**	32179	3294
**[0.14,0.16]**	32179	1564
**[0.16,0.18]**	35198	914
**[0.18,0.2]**	35434	988

We repeated this analysis using only the segment-level annotations from the introgression maps (i.e., without relying on per-SNP archaic/non-archaic labels). We compared imputation accuracy for SNPs located in archaic versus non-archaic segments and again observed higher accuracy in archaic segments than in non-archaic segments (Supplementary Fig. 16).

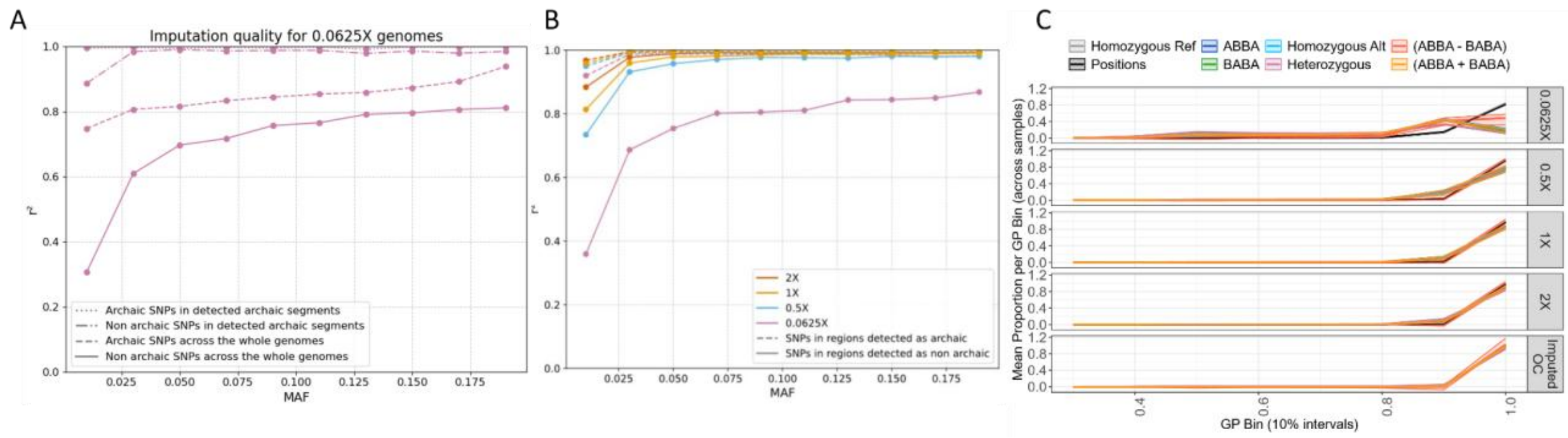

Supplementary Fig. 16. Imputation accuracy (1-MSE) as a function of minor allele frequency (MAF). A) Imputation performance for the 20 individuals downsampled to 0.0625X coverage, shown separately for different SNP categories: Archaic SNPs and Non-Archaic SNPs, in both archaic regions and across the whole genome. B) Imputation accuracy across different coverage levels for SNPs located in Archaic and Non-Archaic regions. C) Distribution of site and genotype characteristics across genotype probability (GP) bins at different coverage levels. The figure shows the mean proportion (solid line) and range (shaded area) of various patterns—including genotype categories (homozygous reference, homozygous alternative, heterozygous), ABBA/BABA signals, and total positions—across GP bins of width 0.1, stratified by coverage. Each curve represents the average across individuals.

We have shown that archaic SNPs are easier to impute at all MAF levels compared to non archaic SNPs at the same MAF. Yet archaic SNPs at very low MAF are still harder to impute than non archaic SNPs at higher MAF. For this reason, we studied the MAF distribution of archaic SNPs and non archaic SNPs (Supplementary Fig. 17), as expected [35].

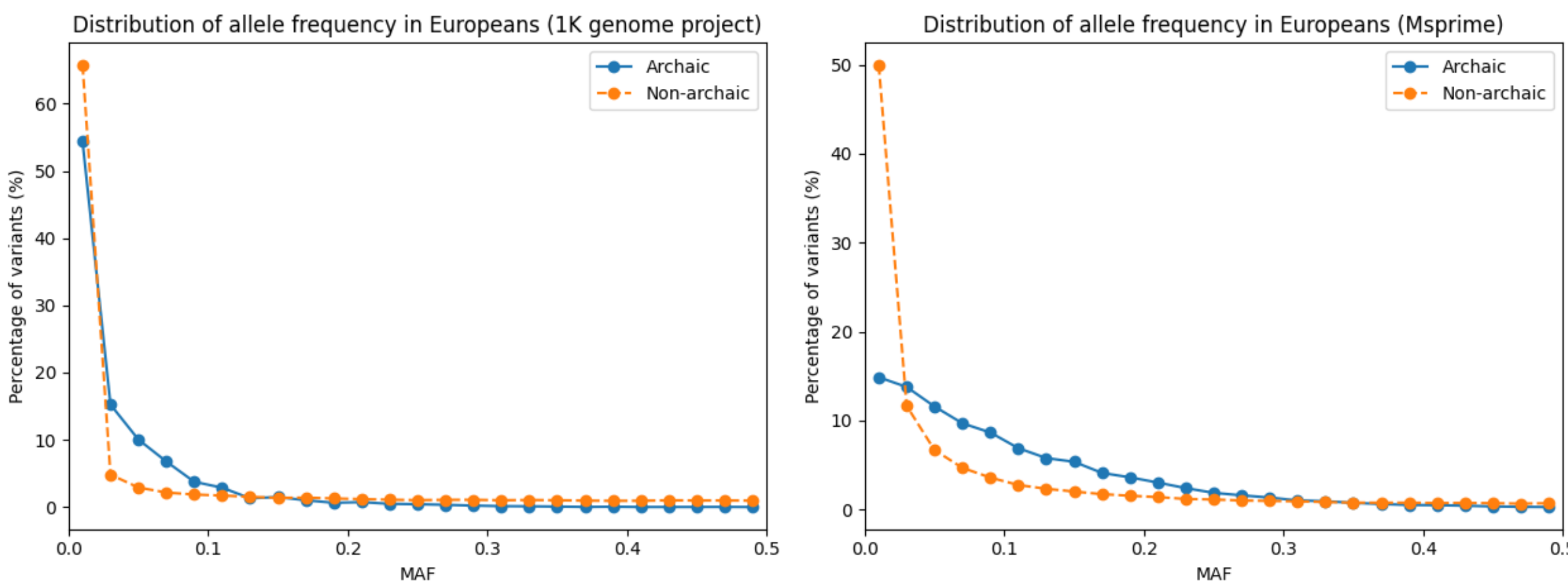

Supplementary Fig. 17: Distribution of allele frequency in Europeans from the 1000 genome project, chromosome 1 (left) and from msprime simulations (right), for archaic and non-archaic alleles. Alleles present in the 1000 genome project are defined as archaic if they are present in Vindija Neanderthal, but absent in Africans (Yoruba, Mende and Esan). Alleles in msprime are defined as archaic if the mutation happened in the Neanderthal population, the information being directly taken from the tree sequence. We observe an excess of archaic alleles in the allele frequency range 2-10%. The simulations assume Out of Africa 2000 generations ago, Neanderthal admixture (4%) 1850 generations ago, population sizes of 27000, 10000 and 30000 for respectively Africans, Europeans, Neanderthal. A sequence of size 250,000,000 is simulated for 150 diploid Europeans.

Because most ancient individuals in our panel are from West Eurasia, we expect the majority of informative SNPs to reflect Neanderthal introgression. To specifically assess Denisovan accuracy, we repeated the analysis restricting first to Denisovan SNPs and then to Denisovan-private SNPs, asking whether any effect was specific to Neanderthal-like sites. We define Denisovan SNPs as archaic SNPs identified using the Altai Denisovan genome in place of Vindija Neanderthal. Denisovan-private SNPs are Denisovan SNPs for which the Denisovan allele is absent not only from African populations but also from Vindija Neanderthal. The results hold (Supplementary Fig. 18): both Denisovan SNPs and Denisovan-private SNPs are imputed more accurately than non-archaic SNPs, although Denisovan-private SNPs are imputed less accurately than archaic SNPs defined using Vindija Neanderthal. These findings indicate that archaic SNPs—both genome-wide and within archaic segments—are imputed more accurately than non-archaic counterparts regardless of archaic origin, while additional analyses in other ancient populations would further validate this pattern.

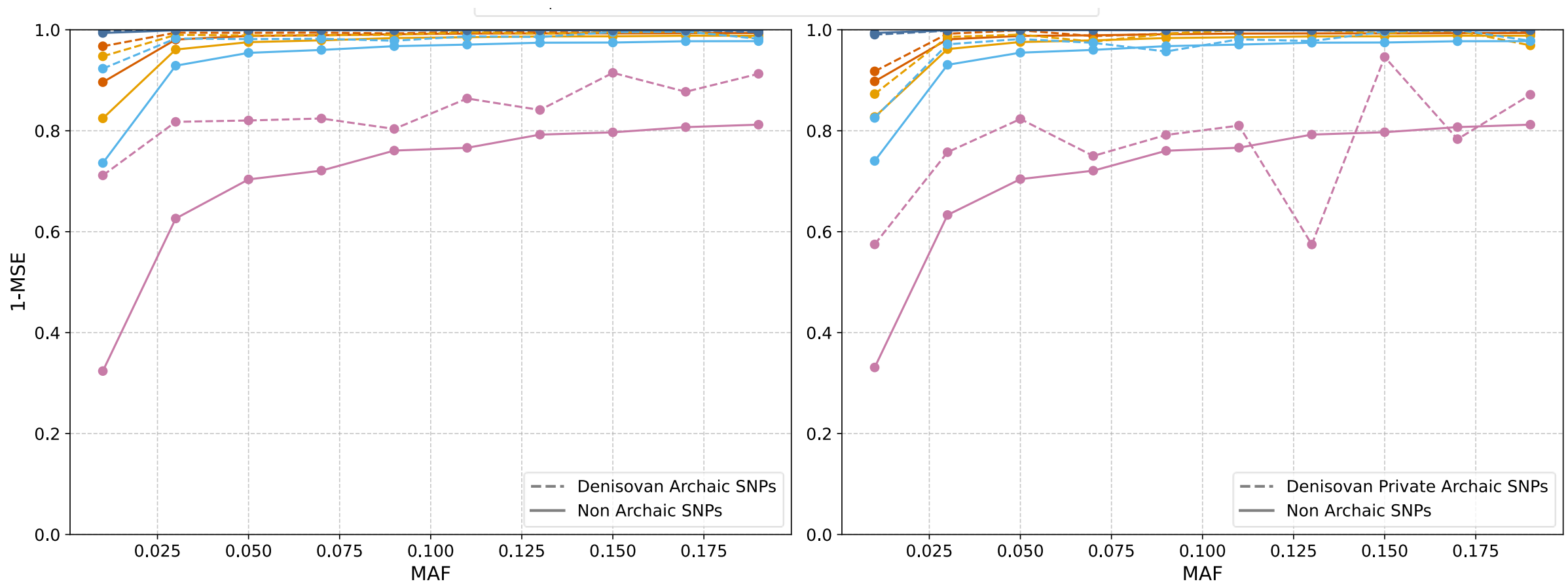

Supplementary Fig. 18. Imputation concordance for archaic and non-archaic SNPs across different downsampled datasets and Minor Allele Frequencies (MAF) in the reference panel. On the left, Denisovan Archaic SNPs are positions with a variant absent in African populations (Yoruba, Mende and Esan from the 1000 Genome Project) and homozygous in Altai Denisovan. On the right, Denisovan Private Archaic SNPs are positions with a variant absent in African populations as well as in Vindija Neanderthal, while being homozygous in Altai Denisovan.

We also compared the imputation performance of the Archaic New and Archaic Shared groups, this time using results from the archaic LAI and a different metric: heterozygous sensitivity. This metric measures sensitivity to the imputation of heterozygous sites, which are common at archaic positions and are the most challenging to impute. To assess heterozygous sensitivity, we used the GenotypeConcordance tool in Picard v3.0, comparing the original and imputed VCF files for each individual. We observed higher heterozygous sensitivity in archaic segments than in non-archaic regions, with no significant differences between the Archaic New and Archaic Shared groups or among individuals (Figure 3B, Supplementary Fig. 19). The tables underlying these plots are reported in Source Data 5-6. For consistency we also plotted per individual average genotype probability (GP) for ABBA and BABA sites (Supplementary Fig. 20).

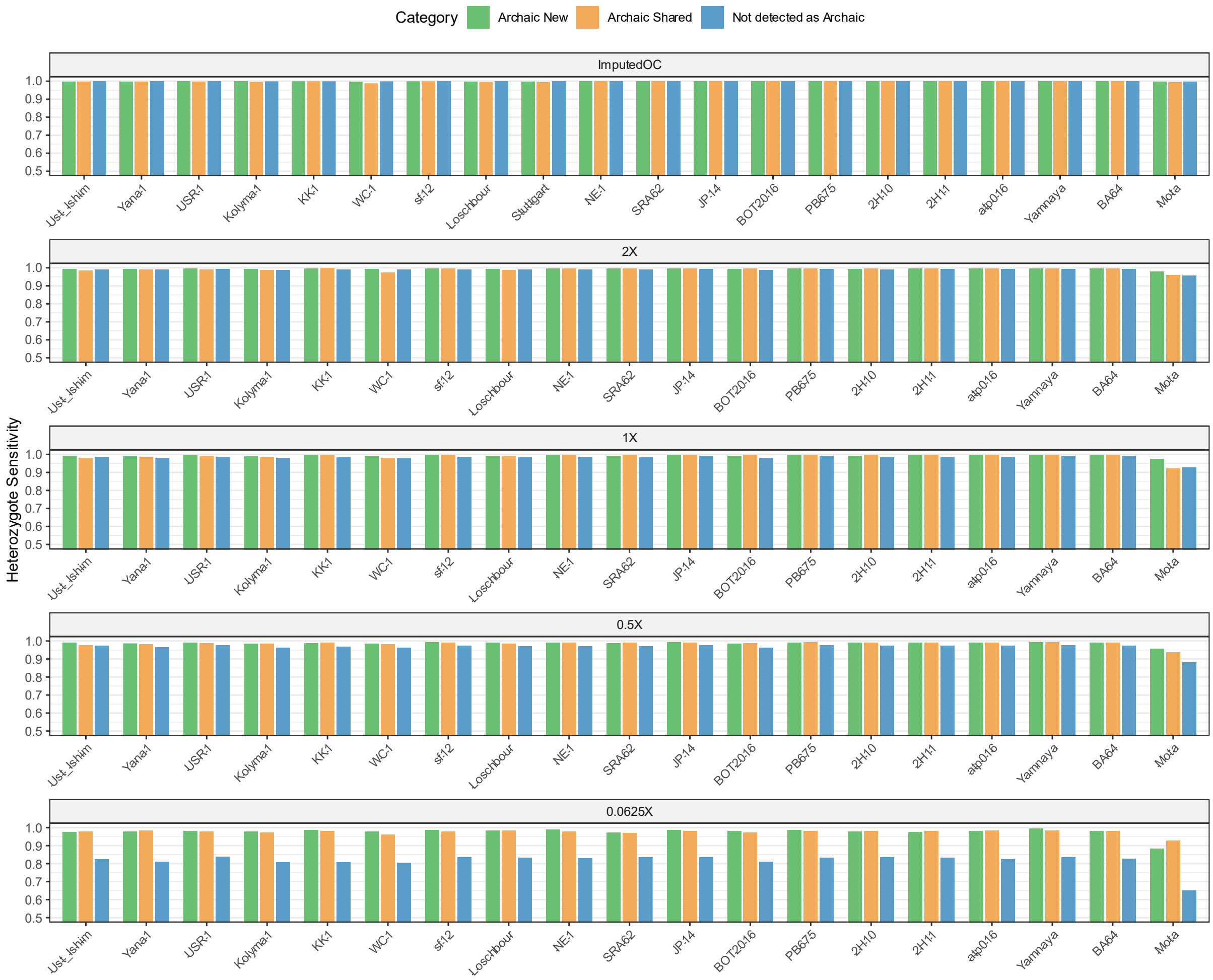

Supplementary Fig. 19. Per individual plot of the heterozygous sensitivity in the 3 categories Archaic New, Archaic Shared and Not detected as archaic.

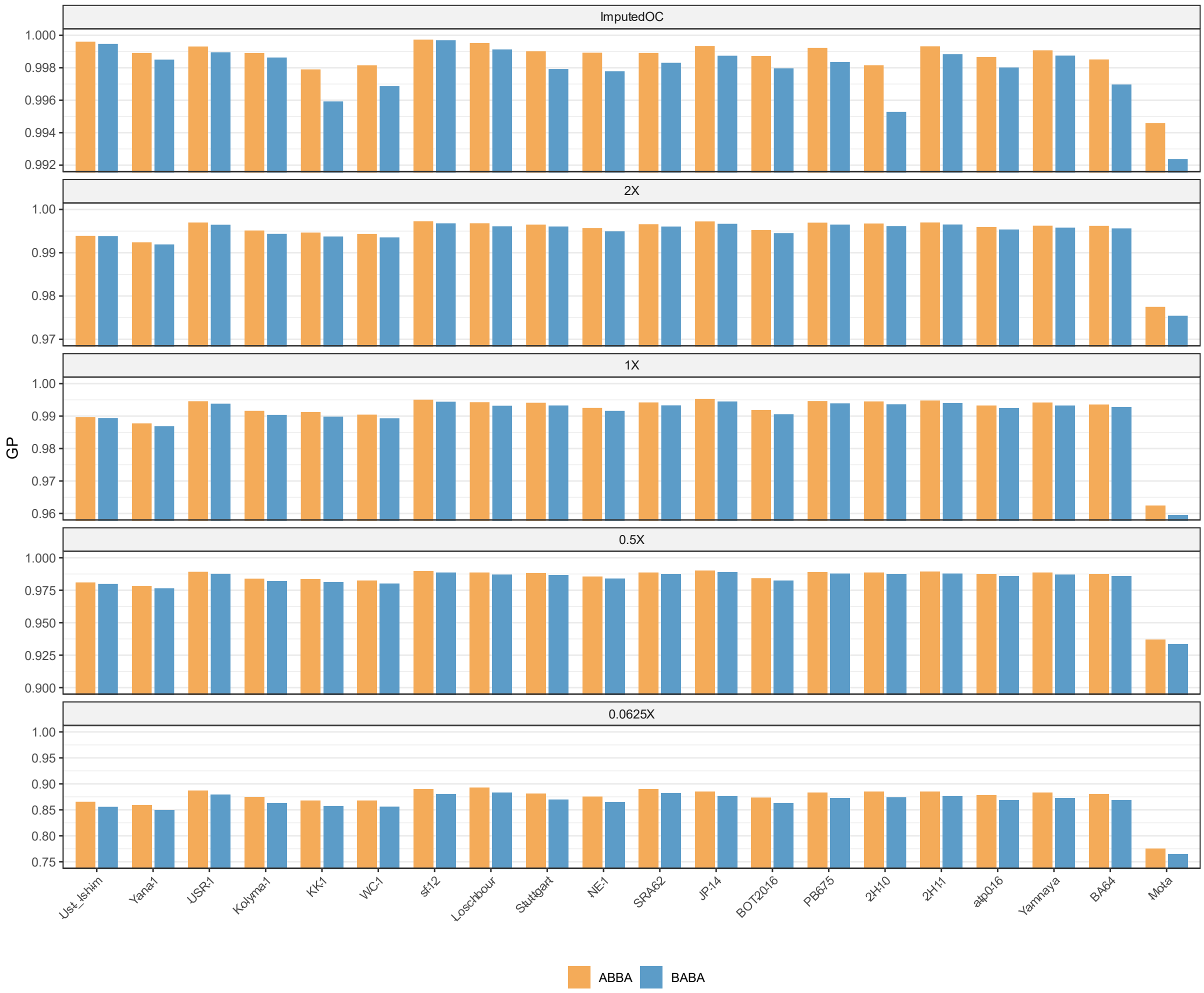

Supplementary Fig. 20. Per individual plot of the average genotype probability for ABBA and BABA sites across different coverage levels, based on D-statistics of the form D(Africa, Ancient; Altai Neanderthal, Chimp).

11. BAM-derived support for imputed variants in Archaic New segments

To assess whether imputation introduces bias, we first evaluated imputation concordance and heterozygous similarity; however, these tests rely on genotypes present in the original VCFs. Because the original VCF files contain more missing sites than the Imputed VCF files, our previous positive results are limited to variants that passed the original calling filters. Additional evidence resides in the source BAM files. To ensure that imputation does not introduce unsupported alleles—particularly within Archaic-New segments that pass our quality filters (archaic similarity ≥ 0.99 and segment length ≥ 40 kb), where missingness in the Original VCF is higher (Table 1, Supplementary Data 3)—we quantified read support for each allele.

Specifically, for each individual and each imputed coverage, we extracted sites, in Archaic New regions, present in the Imputed VCF but missing in the Original VCF. For this SNP set, we returned to the original BAM and using "Rsamtools" tools we enumerated all observed alleles, counting the number of reads supporting each allele. We then computed read support for the reference and alternative alleles and derived the proportion of alternative reads (ignoring any less-represented non-ref/non-alt alleles) as:

$$Proportion\ Alt = \frac{ALT\ reads}{REF\ reads + ALT\ reads} \qquad (9)$$

For every position and coverage, we also recorded the imputed genotype class (Homozygous Reference, Heterozygous, Homozygous Alternative).

Focusing on the alternative allele (Homozygous Alternative and Heterozygous), the class most prone to mapping issues due to reference-mapping bias, we first examined the distribution of the proportion of reads carrying the alternative allele (Supplementary Fig. 21). Most imputed genotypes that are missing in the Original VCF are supported by the expected read proportions: 1 for Homozygous Alternative and 0.5 for Heterozygous sites. For Heterozygous sites, however, the actual mean proportion is consistently below 0.5. This downward shift is consistent with the known reference bias. Moreover we can observe that the distribution for the heterozygous sites often follows a binomial distribution, with a heavier mode below 0.5. The presence of two modes could be explained by the Allele Balance filter (40–60%) applied during the original variant calling, which would exclude true heterozygotes whose alternative-allele proportion falls <40% or >60%.

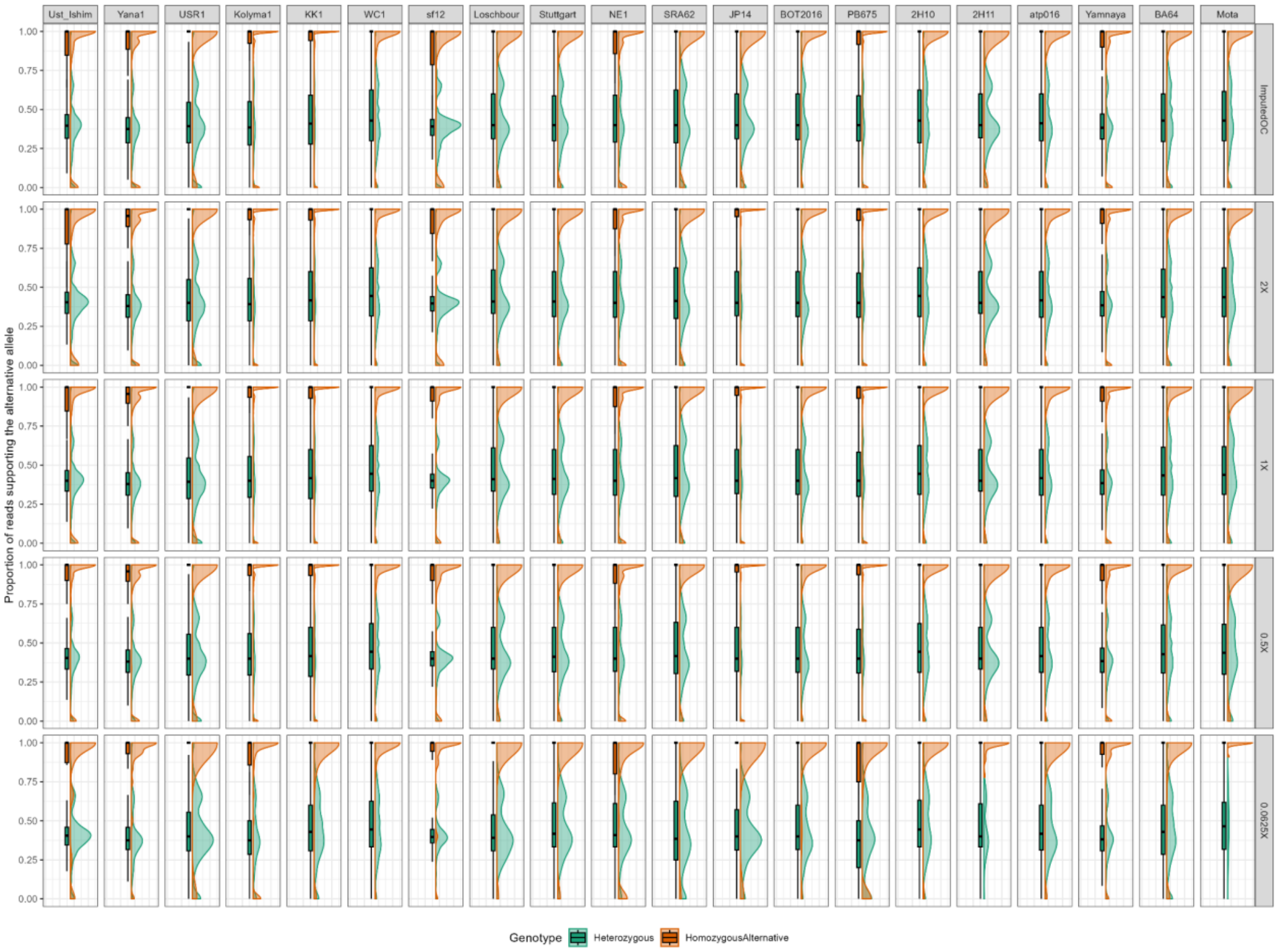

Supplementary Fig. 21. Distribution of the proportion of reads at positions missing in the original VCF and located within Archaic New regions that carry the alternative allele, stratified by genotype, coverage level, and individual. For each panel (rows = coverage category; columns = individual), rainclouds show the distribution of the alternative allele fraction across sites, split by genotype (heterozygous versus homozygous alternative). Each raincloud combines a half-violin (density) with an embedded boxplot (median and interquartile range), with individual points omitted for clarity. As expected, Homozygous Alternative clusters near high ALT fractions, while Heterozygous centres around intermediate values; shifts or spread reflect coverage effects and individual-specific variability.

Moreover, we observe a small number of heterozygous and homozygous-alternative sites with an alternative-allele proportion near zero. To investigate these, we plotted the alternative-allele proportion as a function of total read depth (Supplementary Fig. 22). As expected, sites with very low alternative support are largely explained by limited coverage, most have <5 total reads, and many have zero reads. Notably, the two most challenging genomes to impute (Ust'Ishim and Mota) do not deviate from this pattern; their behaviour closely matches that of the other individuals.

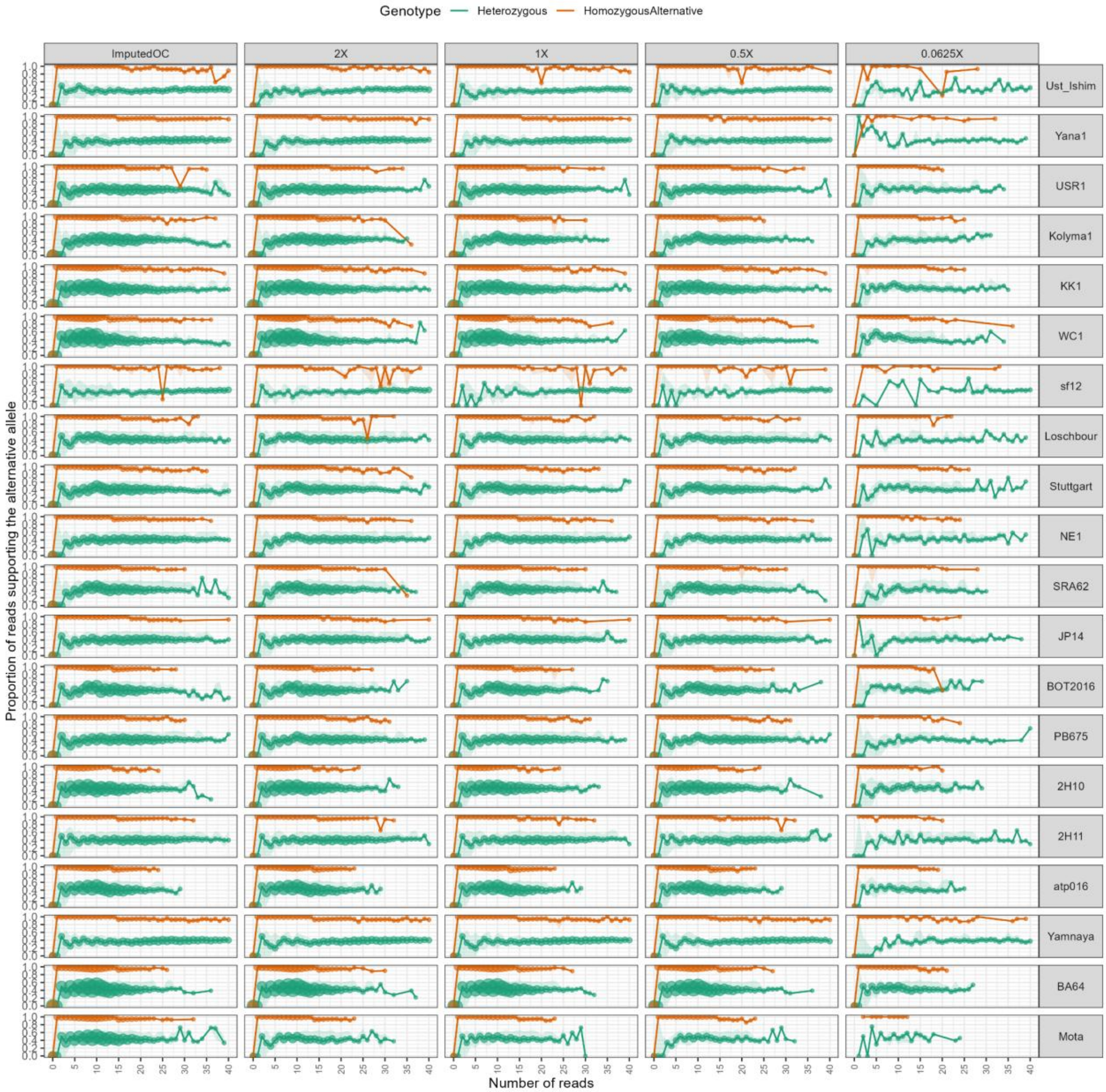

Supplementary Fig. 22. Proportion of reads, at positions missing in the original VCF and located within Archaic New regions, carrying the alternative allele as a function of read depth by genotype. For each individual and coverage, lines show the median ALT proportion across sites at each read depth, with shaded IQR (25–75%). Point size indicates the number of sites contributing at that depth. Depths >40 are omitted.

We further quantified how sequencing gaps contribute to the fraction of missing sites in the Original VCF within Archaic-New segments (Supplementary Fig. 23). Across individuals and coverages, 0–10.8% of sites have no reads in the BAM, and 2.6–20.7% of sites show an alternative-allele proportion <10%. Restricting to sites with depth >0, 2.3–11.1% have alternative-allele proportion <10%, closely matching the share of sites with zero ALT reads (2–10.7%).

Low alternative support at sites with <10% ALT reads is largely driven by depth: these sites are dominated by positions with no reads (0–71%) or <10 total reads (66.7–100%). Differences across individuals (rows) and imputed coverages (columns) are minor, including for Ust'Ishim and Mota. Overall, the majority of sites that are missing in the Original VCF but imputed as carrying an alternative allele are supported by reads in 80–97% of cases.

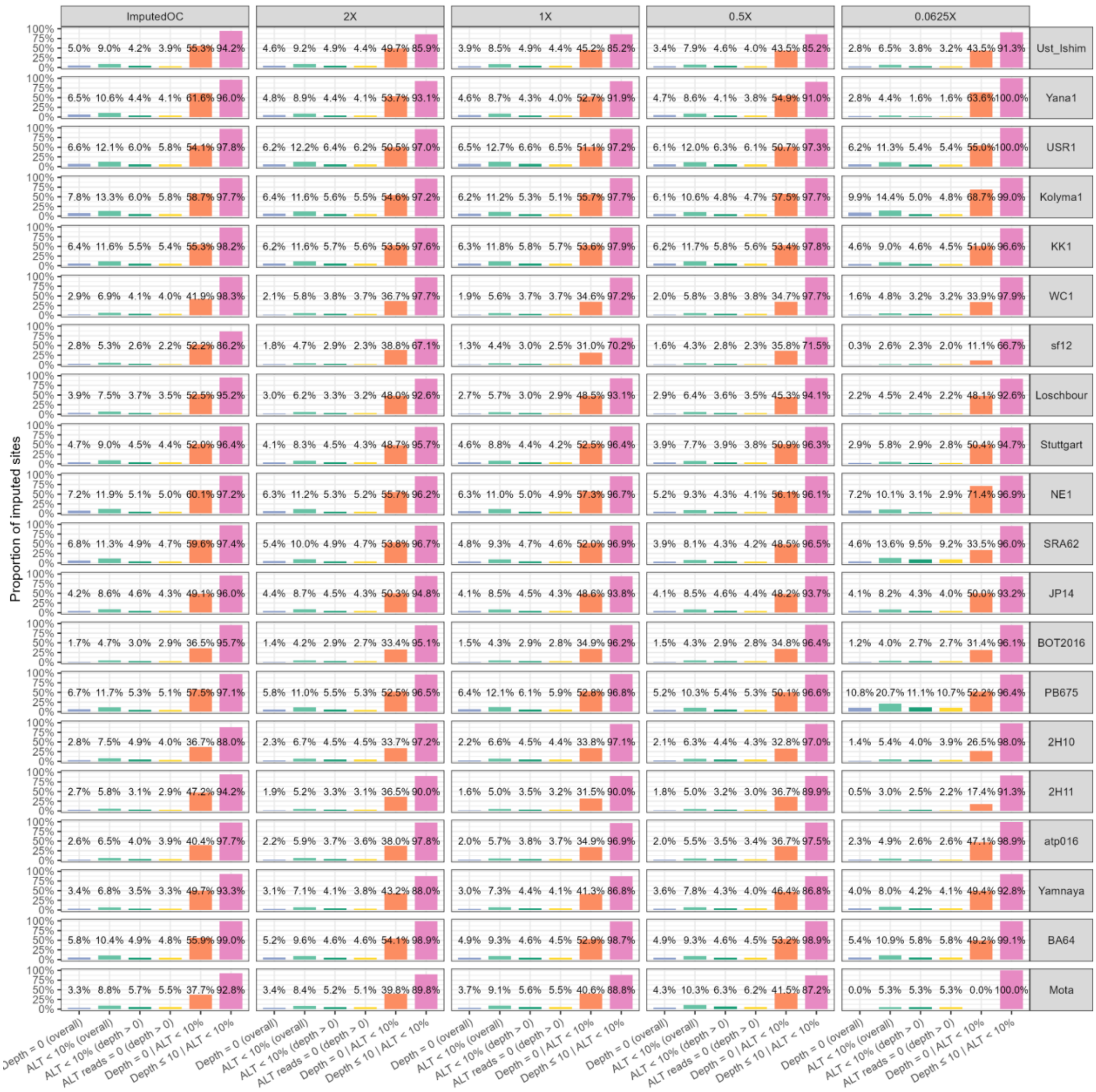

Supplementary Fig. 23. Summary of depth and ALT-allele proportion metrics per Individual × Coverage, restricted to sites whose imputed genotype carries an ALT allele (Heterozygous or Homozygous-ALT) at positions missing in the original VCF and located within Archaic New regions. Bars (left→right): (i) fraction of sites with total depth =0; (ii) fraction of sites with ALT proportion <10%; (iii) fraction with ALT proportion <10% among sites with depth > 0; (iv) fraction with zero ALT reads among sites with depth >0; (v) fraction with depth =0 among sites with ALT proportion <10%; and (vi) fraction with depth ≤10 among sites with ALT proportion <10%.

Most sites with no ALT-supporting reads occur at low depth. Examining total depth at positions where the ALT allele constitutes <10% of reads, we find that the majority have 1–10 total reads and are overrepresented among heterozygous sites, a comparatively small class (hundreds of SNPs) relative to the thousands analyzed overall (Supplementary Fig. 24). Taken together, these results indicate that the vast majority of SNPs in Archaic-New segments are correctly imputed and supported by the underlying reads; the few discordant cases arise primarily at low coverage, where genotype calls are only partially supported.

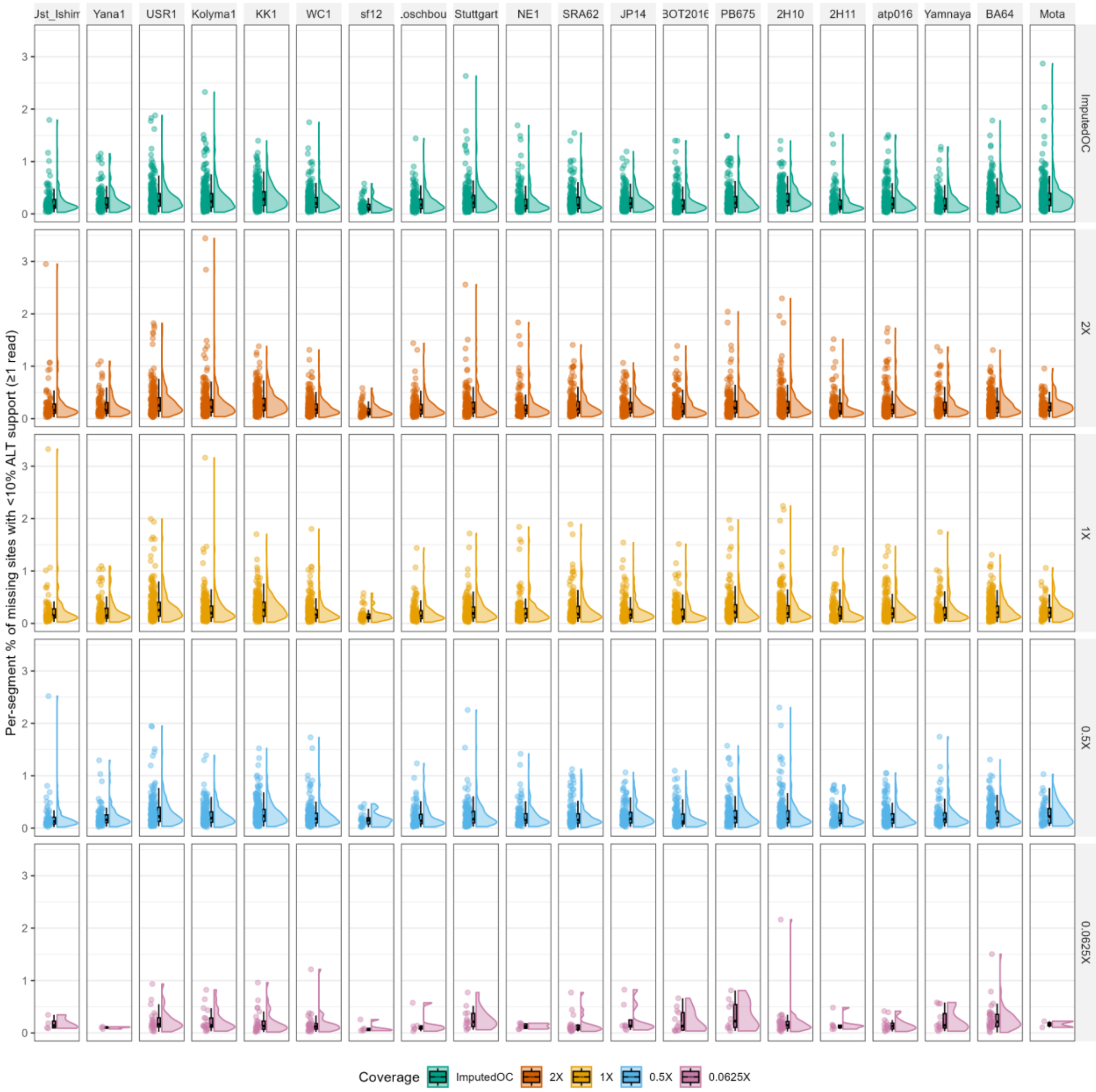

Supplementary Fig. 24. Raincloud plots showing the distribution of the SNPs that are represented by at least 1 read and with less than <10% of the reads that support the alternative allele, as total of all the missing positions in the Archaic New segments.

12. Analysis of Shared Introgressed Regions with Contemporary Individuals

For contemporary datasets we use hmmix's map, containing individuals from Simons Genome Diversity Project (SGDP), 40 Papuans from[36] and 35 Papuans from[37] as well as ArchaicSeeker2.0's map containing individuals from the 1000 Genome Project and Papuans from SGDP[38].

We look for each individual at the amount of archaic segments they share with each contemporary population. In hmmix's map, contemporary individuals are assigned to one of four regions: East Asia, West Eurasia, South Asia, or Central Asia–Siberia. In ArchaicSeeker2.0's map, individuals first belong to a local population as defined in the 1000 Genomes Project, which in turn belongs to a continental region: East Asia, West Eurasia, or South Asia.

To compute heatmaps (Supplementary Fig. 25-S27, Source Data 8), for each individual we count the number of their archaic segment that is found in each contemporary region. We consider that two segments are matching if they overlap for 80% of their length. If a segment is found multiple times within a region, we count it as many times as it is found. As within introgression maps some contemporary regions contain more individuals than others - especially in hmmix's map - we then divide the match count for each region by the number of individuals in that region and normalize by row.

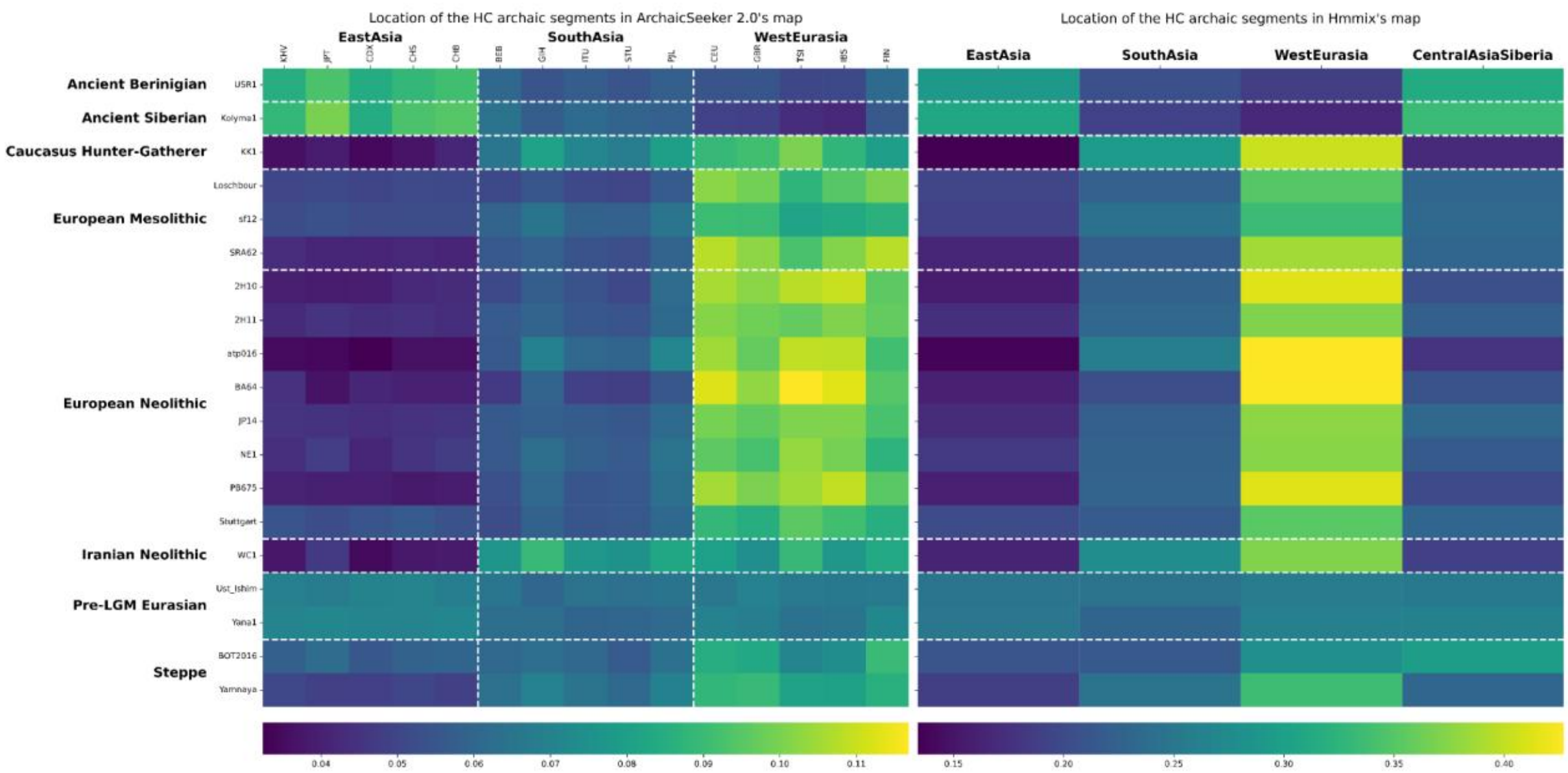

Supplementary Fig. 25. Geographical location of the archaic segments detected in the original coverage genomes within introgression maps of contemporary individuals. For each archaic segment detected in the imputed genomes, we count the number of overlaps with previously published introgression maps from the HGDP and 1000 Genomes Project. We then divide this count by the total number of segments of the same origin in the introgression map. Finally, the values are row-normalized.

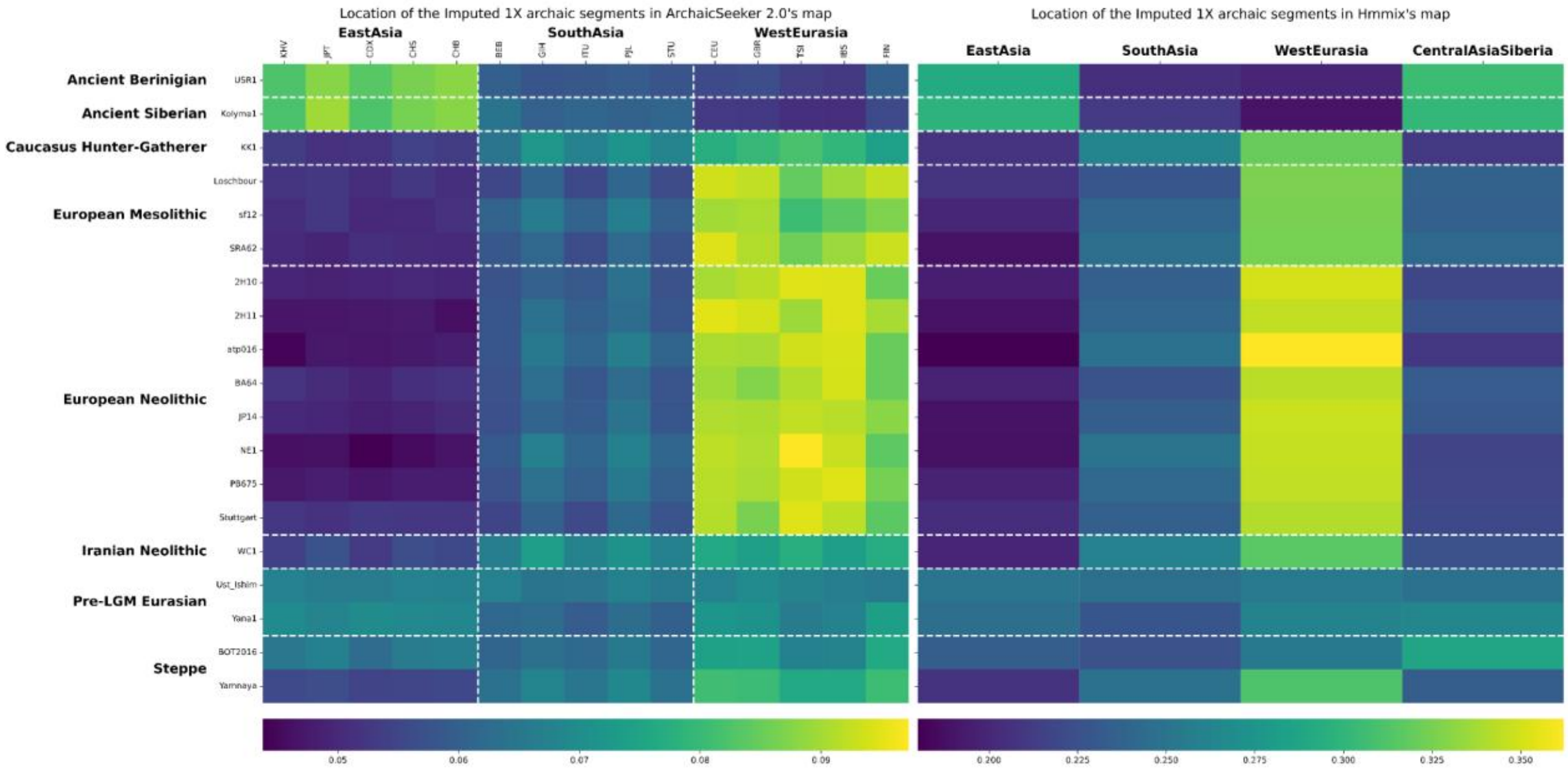

Supplementary Fig. 26. Geographical location of the archaic segments detected in the imputed 1X genomes within introgression maps of contemporary individuals. For each archaic segment detected in the imputed genomes, we count the number of overlaps with previously published introgression maps from the HGDP and 1000 Genomes Project. We then divide this count by the total number of segments of the same origin in the introgression map. Finally, the values are row-normalized.

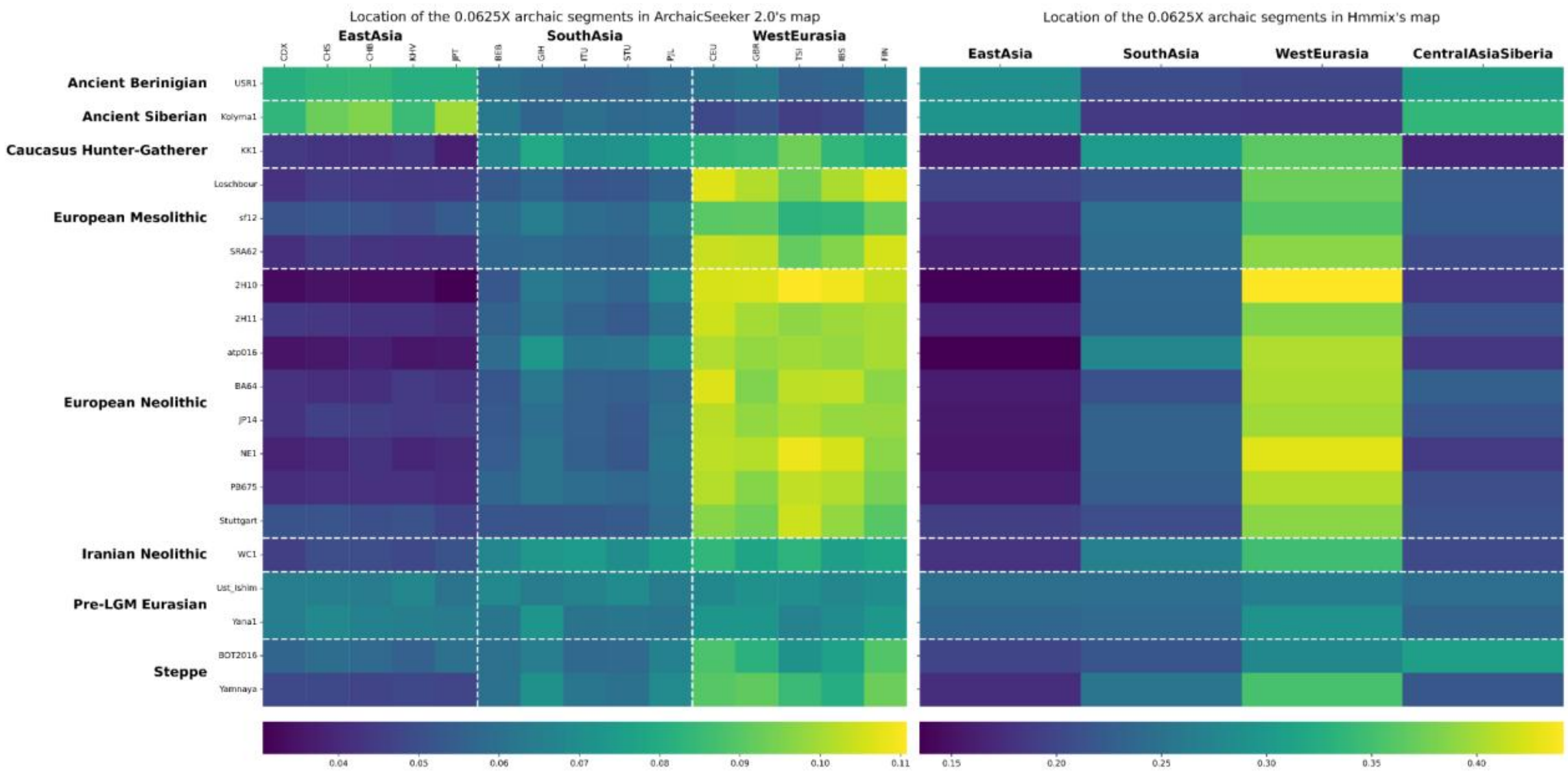

Supplementary Fig. 27. Geographical location of the archaic segments detected in the imputed 0.0625X genomes within introgression maps of contemporary individuals. For each archaic segment detected in the imputed genomes, we count the number of overlaps with previously published introgression maps from the HGDP and 1000 Genomes Project. We then divide this count by the total number of segments of the same origin in the introgression map. Finally, the values are row-normalized.

To compute Venn diagrams (Figure 4A, Supplementary Fig. 28-29, Source Data 9), for each individual and for each of their archaic segments, we look for each region if there are at least two matches in

contemporary individuals present in hmmix' map, if so we mark that the segment is present in the region. If there is no match in any region, we mark the segment as not found. The Venn diagram is then the summary statistic for all archaic segments in the individual.

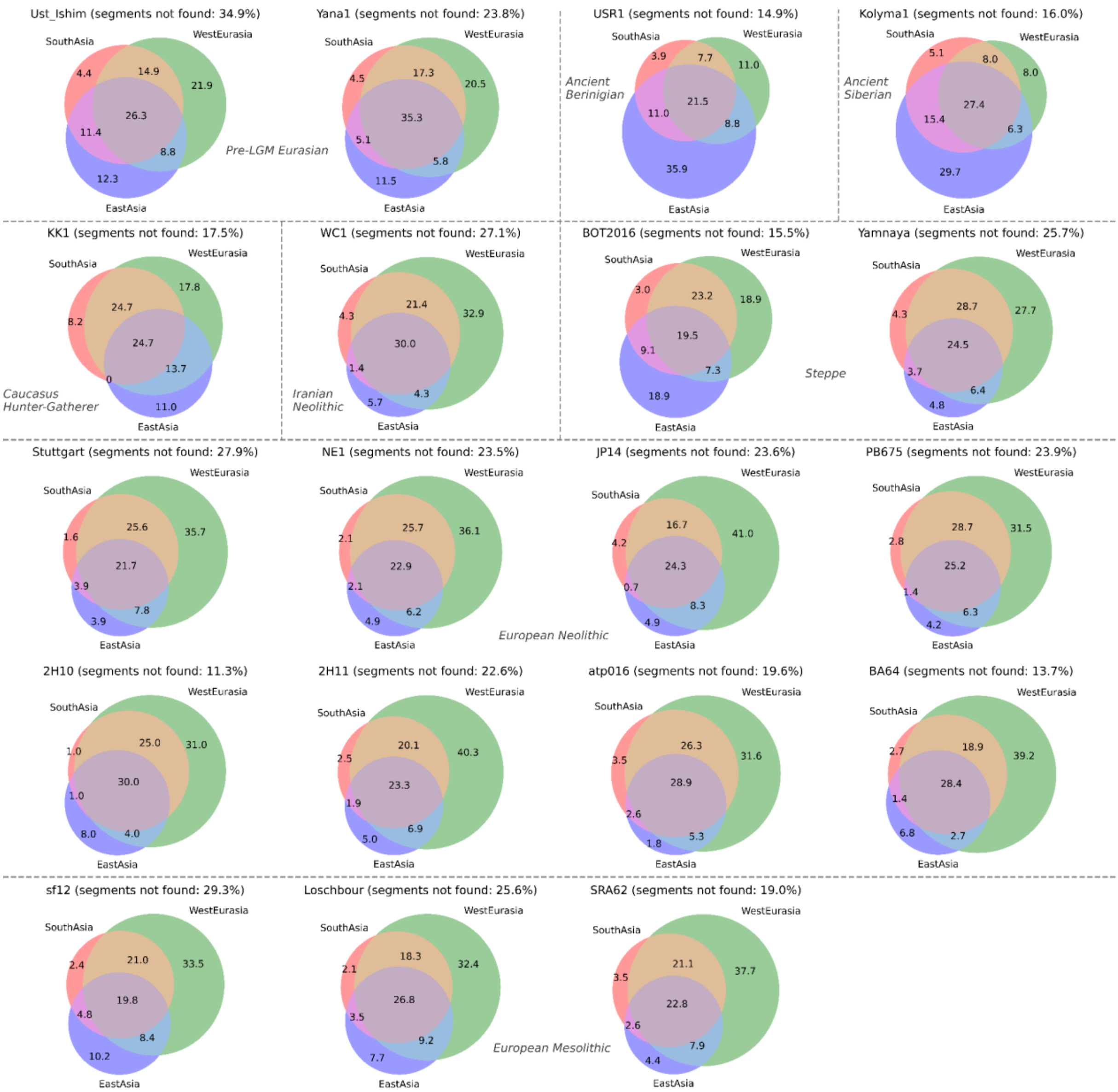

Supplementary Fig. 28. Proportion of the inferred archaic segments detected in the original coverage genomes that are uniquely found in West Eurasia, South Asia, East Asia regions, using contemporary individuals in[27] introgression maps.

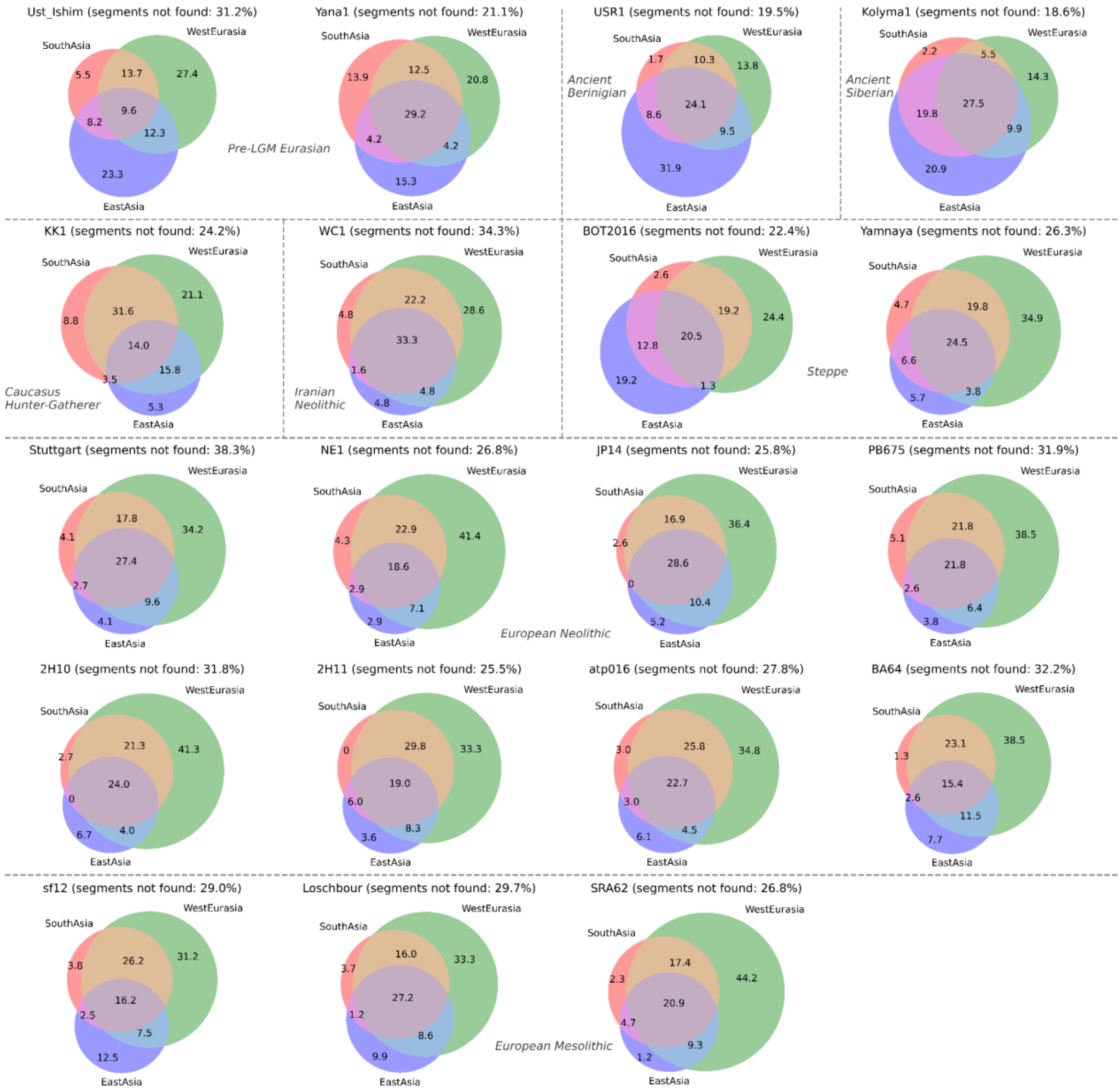

Supplementary Fig. 29. Proportion of the inferred archaic segments detected in the imputed 0.0625X genomes that are uniquely found in West Eurasia, South Asia, East Asia regions, using contemporary individuals in[27] introgression maps.

13. Analysis of shared introgressed regions across ancient individuals

To investigate patterns of haplotype sharing among ancient individuals, we applied a multi-intersection strategy to the set of introgressed fragments previously inferred by archaic local ancestry analysis. For each coverage level, segments were grouped by chromosome and divided into discrete, non-overlapping genomic intervals defined by unique start and end positions (breakpoints) across all individuals. This allowed us to standardize the genomic coordinate system and create binary matrices recording the presence or absence of archaic fragments per individual in each interval, analogous to the multiintersect function in bedtools. Adjacent segments that were contiguous on the genome and shared by overlapping individuals were merged into larger blocks to reduce redundancy.

Using this collapsed matrix, we computed a pairwise segment-sharing matrix by counting the number of introgressed regions shared between each pair of individuals. This matrix was then row-normalized to represent, for each individual, the proportion of their introgressed segments shared with all others.

The resulting matrix was visualized as a clustered heatmap using the pheatmap package in R. Row clustering was based on Spearman correlation with Ward's linkage, and the same order was applied to columns to ensure symmetry. Population labels were added as annotations, and matrix values were displayed as percentages representing the proportion of shared introgressed segments relative to each row individual's total (Supplementary Fig. 30-32).

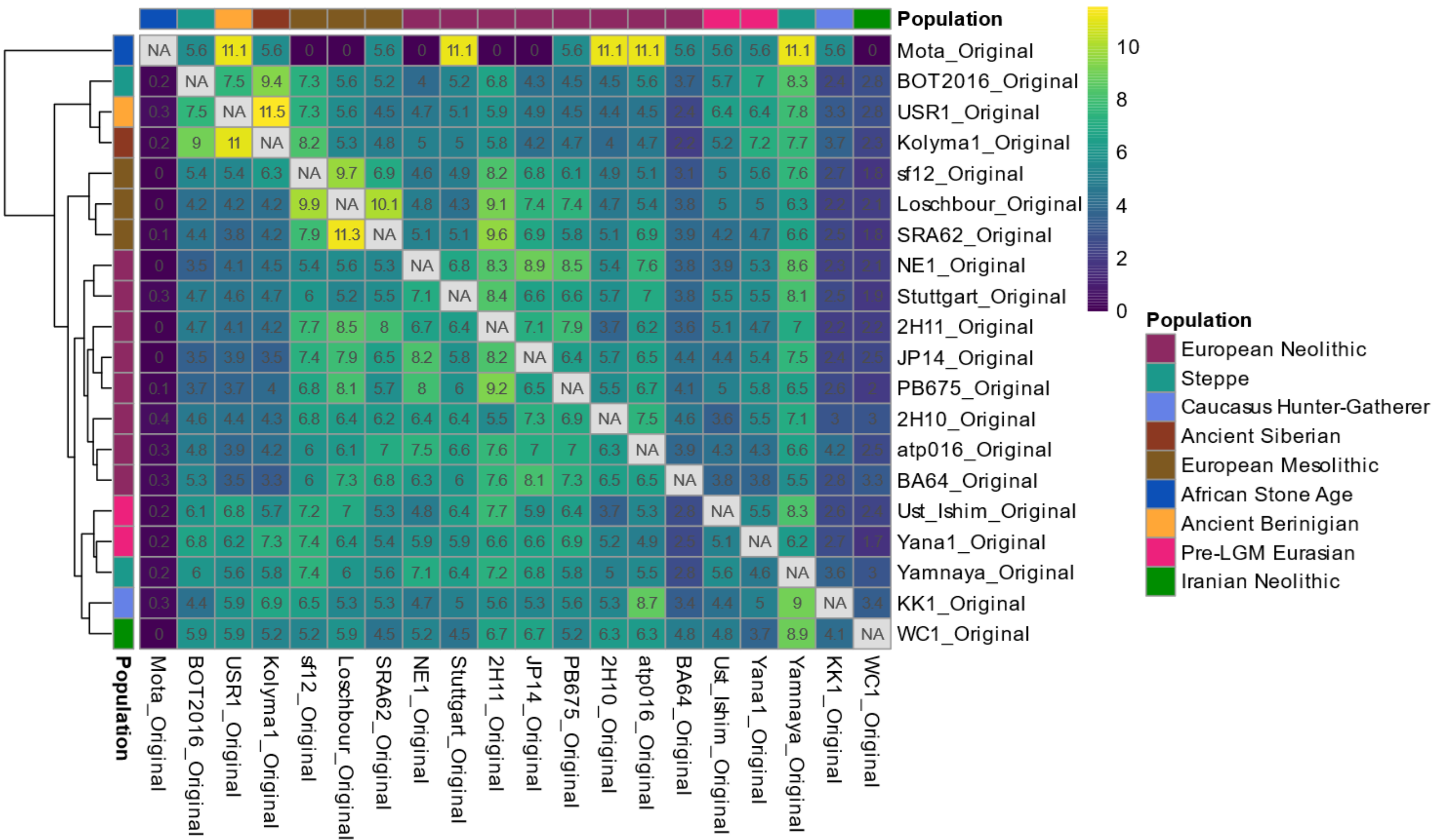

Supplementary Fig. 30. Heatmap of pairwise sharing of post-filtering introgressed genomic segments among ancient individuals for the original coverage dataset. Each cell shows the percentage of introgressed regions in the row individual that are also present in the column individual, based on collapsed shared segments. The matrix is normalized by row totals to account for differences in the amount of introgressed material per individual. Row clustering was performed using Spearman correlation and Ward's linkage, and the same order was applied to columns to preserve symmetry. Population affiliations are indicated by color-coded annotations.

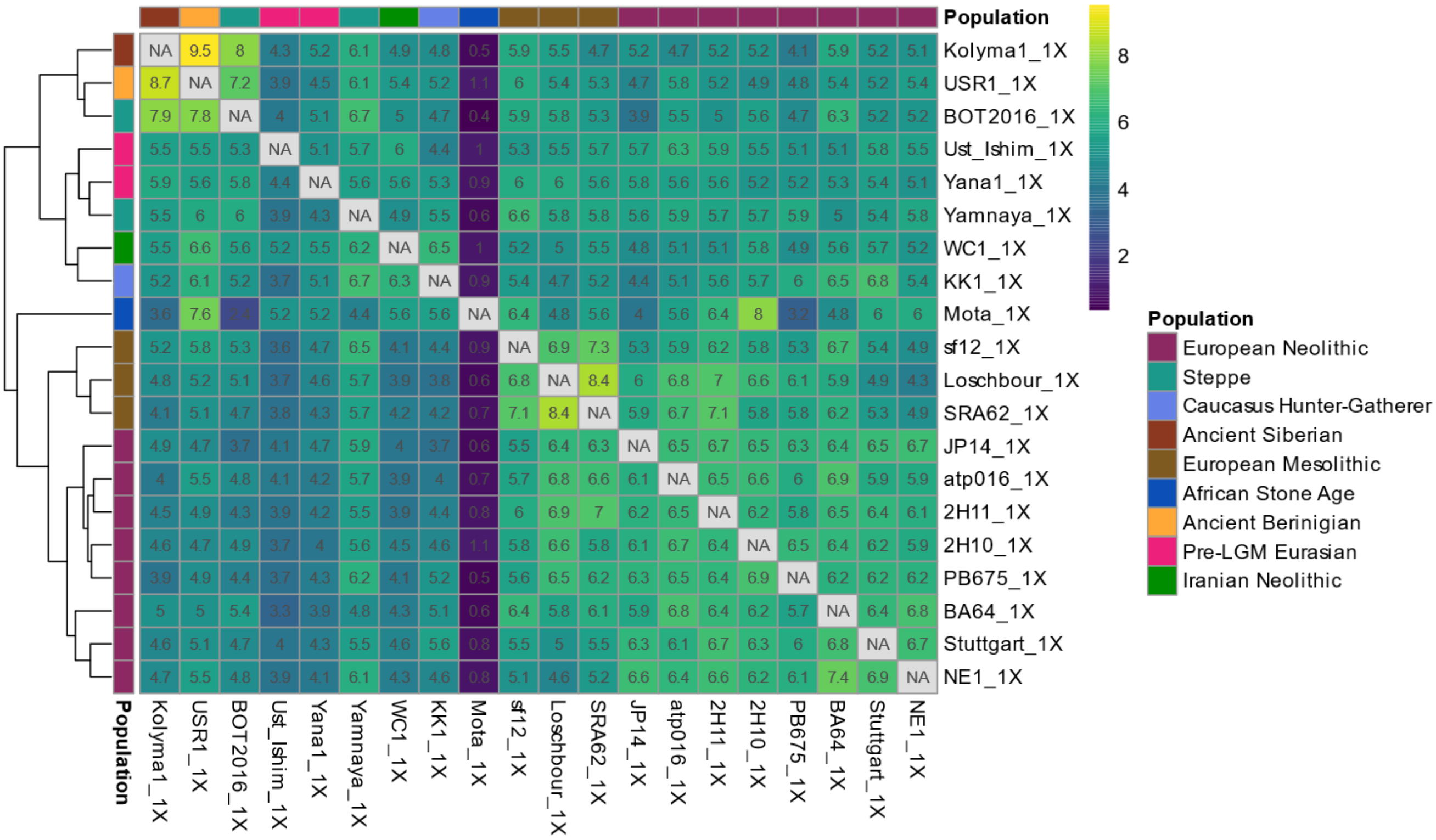

Supplementary Fig. 31. Heatmap of pairwise sharing of post-filtering introgressed genomic segments among ancient individuals for the imputed 1X dataset. Each cell shows the percentage of introgressed regions in the row individual that are also present in the column individual, based on collapsed shared segments. The matrix is normalized by row totals to account for differences in the amount of introgressed material per individual. Row clustering was performed using Spearman correlation and Ward's linkage, and the same order was applied to columns to preserve symmetry. Population affiliations are indicated by color-coded annotations.

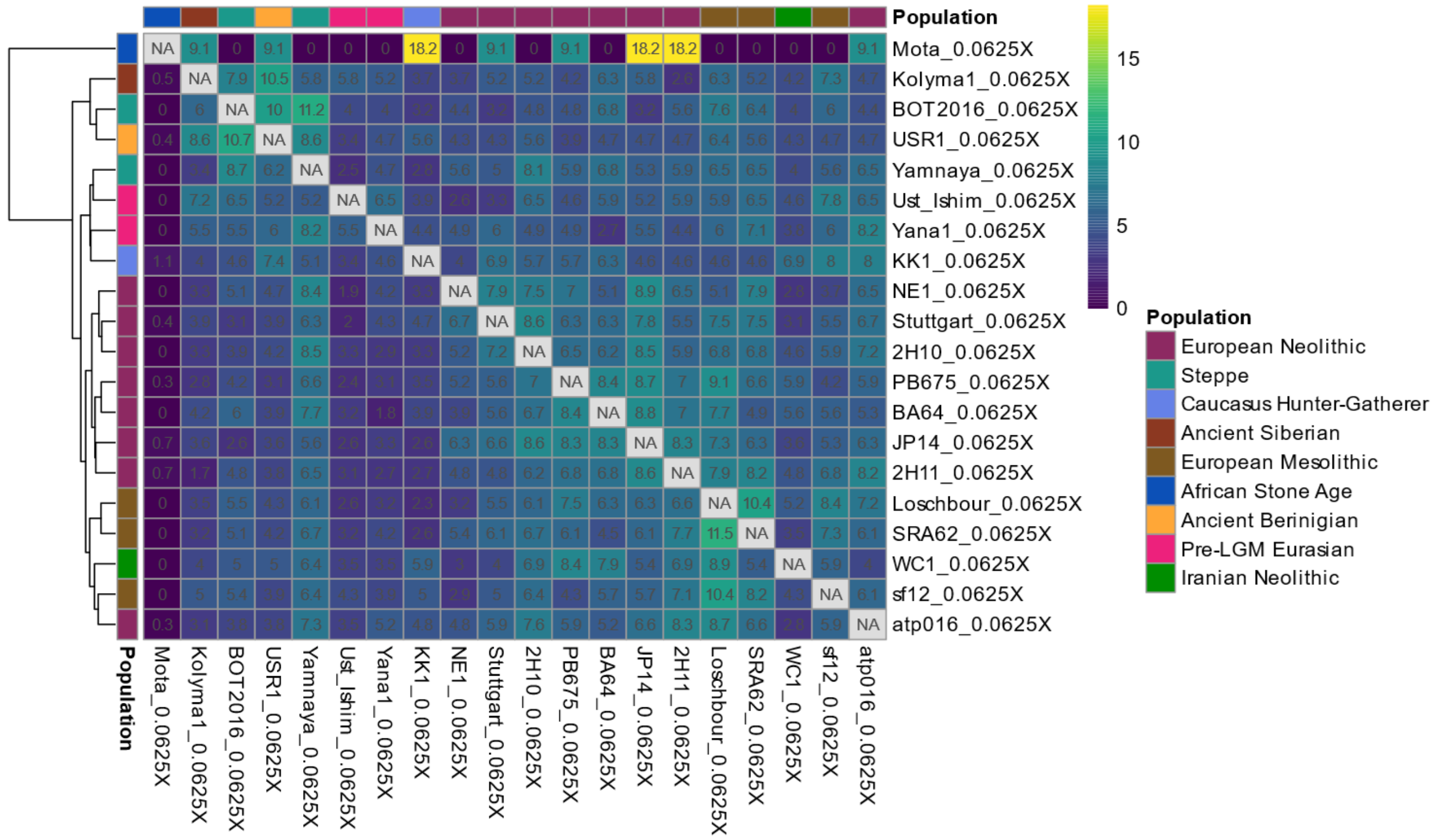

Supplementary Fig. 32. Heatmap of pairwise sharing of post-filtering introgressed genomic segments among ancient individuals for the imputed 0.0625X dataset. Each cell shows the percentage of introgressed regions in the row individual that are also present in the column individual, based on collapsed shared segments. The matrix is normalized by row totals to account for differences in the amount of introgressed material per individual. Row clustering was performed using Spearman correlation and Ward's linkage, and the same order was applied to columns to preserve symmetry. Population affiliations are indicated by color-coded annotations.

To ensure comparability across coverages and reduce sampling bias, we randomly selected a balanced subset of individuals from each coverage level. For this subset, we computed the total length of introgressed fragments shared between every pair of individuals. Shared length was defined as the sum of minimum overlapping lengths across all intervals where both individuals carried a fragment. To account for differences in the total amount of introgressed sequence per individual, we normalized the resulting pairwise matrix row-wise by each individual's total introgressed length. This normalization reflects, for each individual, the proportion of their archaic segments that are shared with others, providing a more interpretable measure of relative haplotype similarity. To visualize population structure and shared ancestry patterns based on archaic content, we applied multidimensional scaling (MDS) to the normalized distance matrix derived from the pairwise sharing values (Figure 4B, Supplementary Fig. 33-37). The resulting two-dimensional coordinates were used to generate scatter plots, in which individuals were color-coded by population and annotated by individual. This allowed us to explore clustering patterns and potential affinities among individuals based on the structure of their shared introgressed genome. These plots highlight both inter-individual similarity and broader group-level trends, offering insight into the extent to which archaic fragments are shared across populations and coverage levels.
Before applying random individual selection, we first compared datasets and introgression maps to assess how category choices affect the inferred pattern of shared archaic ancestry. We constructed a matrix including all individuals and all coverages (Supplementary Fig. 33) and found that Mota consistently

behaves as an outlier across datasets. Conversely, the remaining individuals tend to arrange along a continuum, whose orientation varies by coverage set, from Europeans to "Beringian" individuals. We also observe that individuals from similar time periods cluster together, indicating that introgression maps can recapitulate the genomic history of these populations; in other words, archaic-ancestry structure broadly mirrors geographic and temporal clustering.

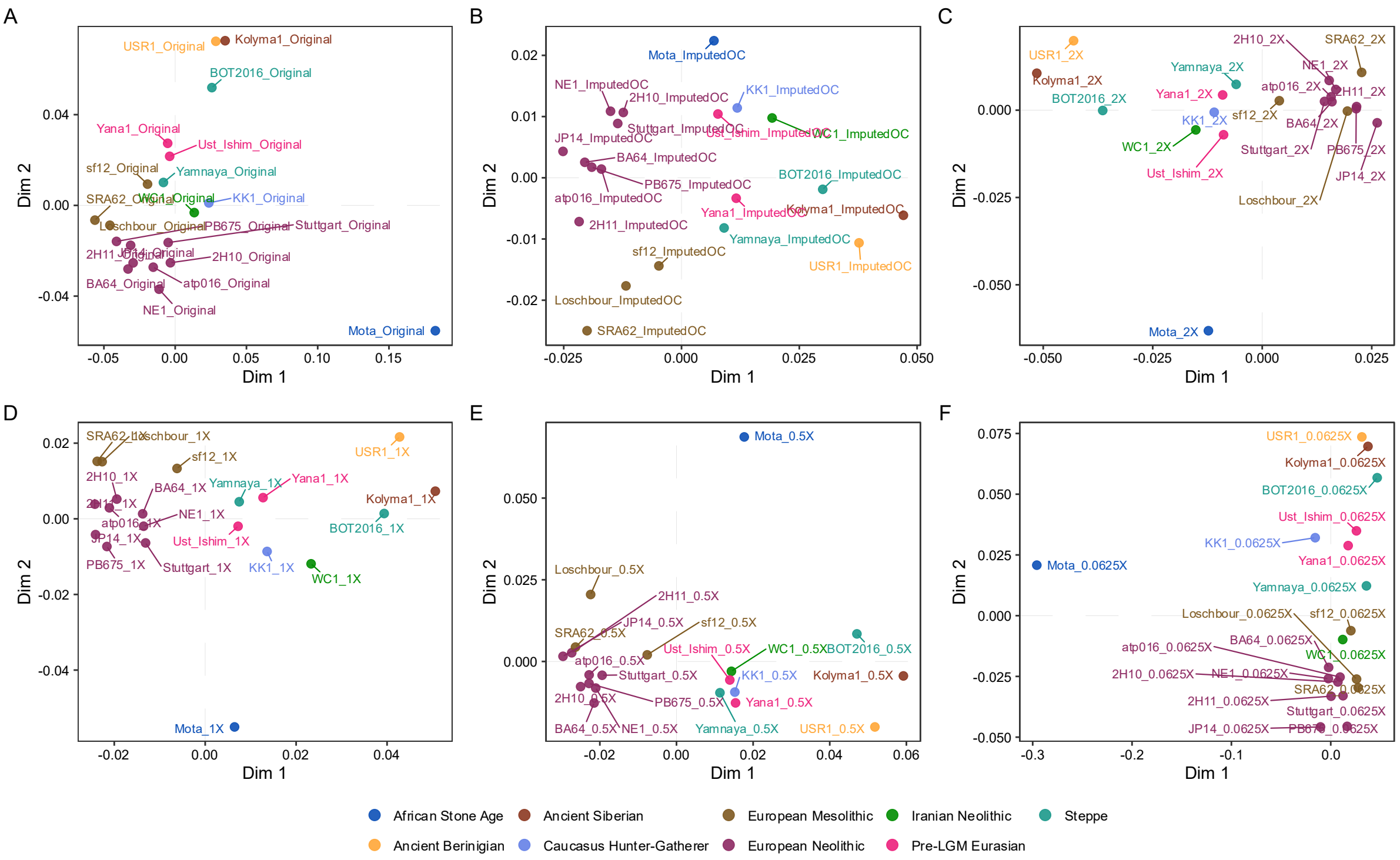

Supplementary Fig. 33. Multidimensional scaling (MDS) plots based on segment count overlap of shared introgressed segments, shown separately for each coverage level: A) Original, B) Imputed OC, C) 2X, D) 1X, E) 0.5X, and F) 0.0625X.

Removing the African individual Mota (Supplementary Fig. 34) preserves the same broad division, likely driven by both inter-cluster sharing and the presence of eastern individuals with Denisovan-enriched ancestry. Without Mota, which compresses the configuration and opens the MDS space, we observe a clearer separation between European Mesolithic and European Neolithic individuals. The Neolithic lie closer to Central Eurasian than do the Mesolithic ones, and the oldest genomes in our panel (Ust'Ishim and Yana1) cluster near the center of the MDS space.

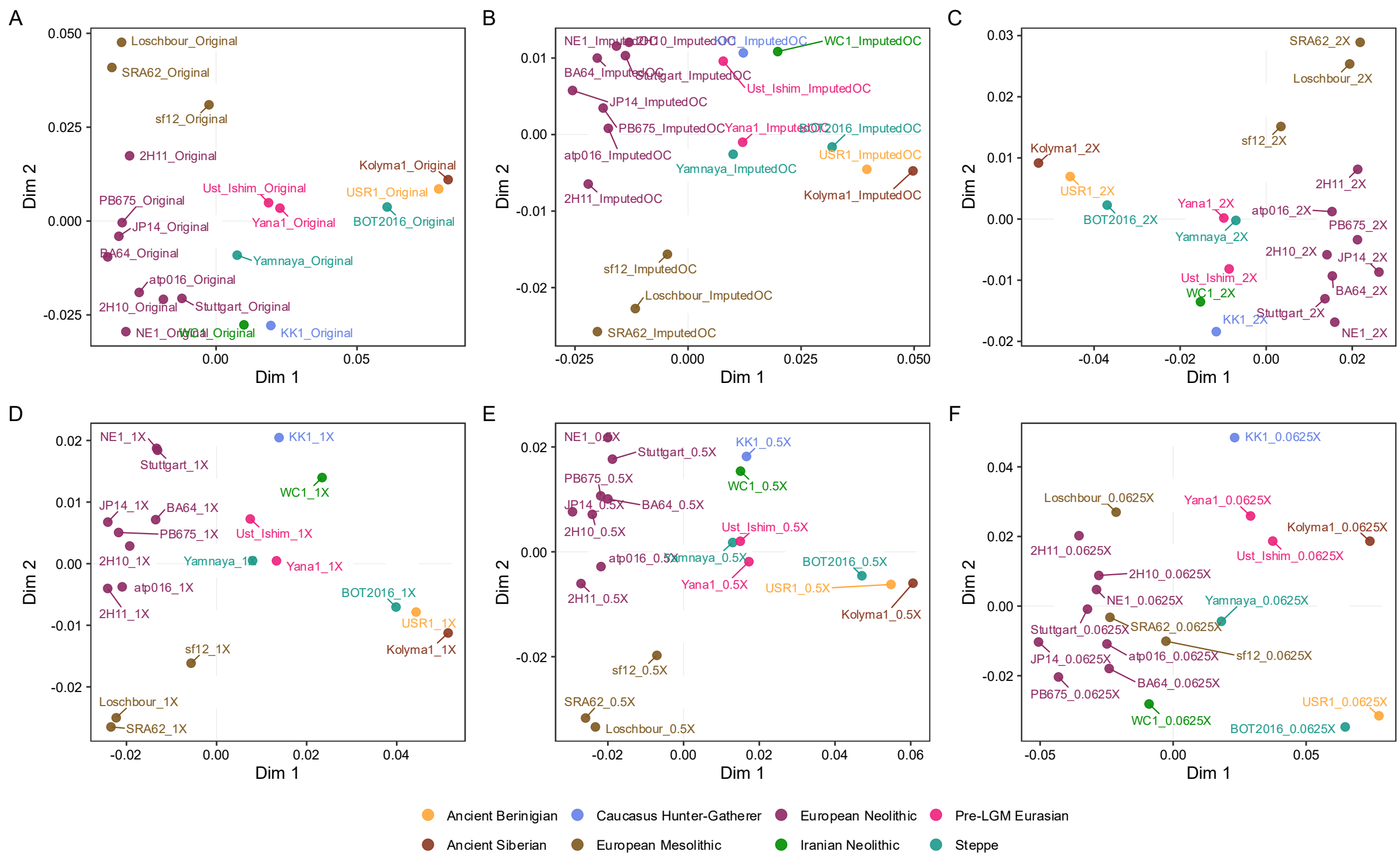

Supplementary Fig. 34. Multidimensional scaling (MDS) plots based on segment count overlap of shared introgressed segments, shown separately for each coverage level: A) Original, B) Imputed OC, C) 2X, D) 1X, E) 0.5X, and F) 0.0625X. In this analysis the individual Mota has been excluded.

To test whether considering only one category, Archaic-Shared or Archaic-New, alters the observed patterns (and to probe any imputation-driven bias, especially in Archaic-New segments), we repeated the analyses by category. Note that the original coverage can influence whether a tract is classified as Shared vs New. Using only Archaic-Shared segments (Supplementary Fig. 35) and excluding the outlier Mota, we recover the same overall structure seen with all segments, with the exception that KK1 and WC1 are less central in the configuration. Using only Archaic-New segments (Supplementary Fig. 36), the broad pattern also persists, but individuals are more dispersed (i.e., less tightly clustered). With the exception of the very low-coverage set (0.0625X), population structure is still maintained in the Archaic-New subset, suggesting no major imputation-induced bias in these tracts.

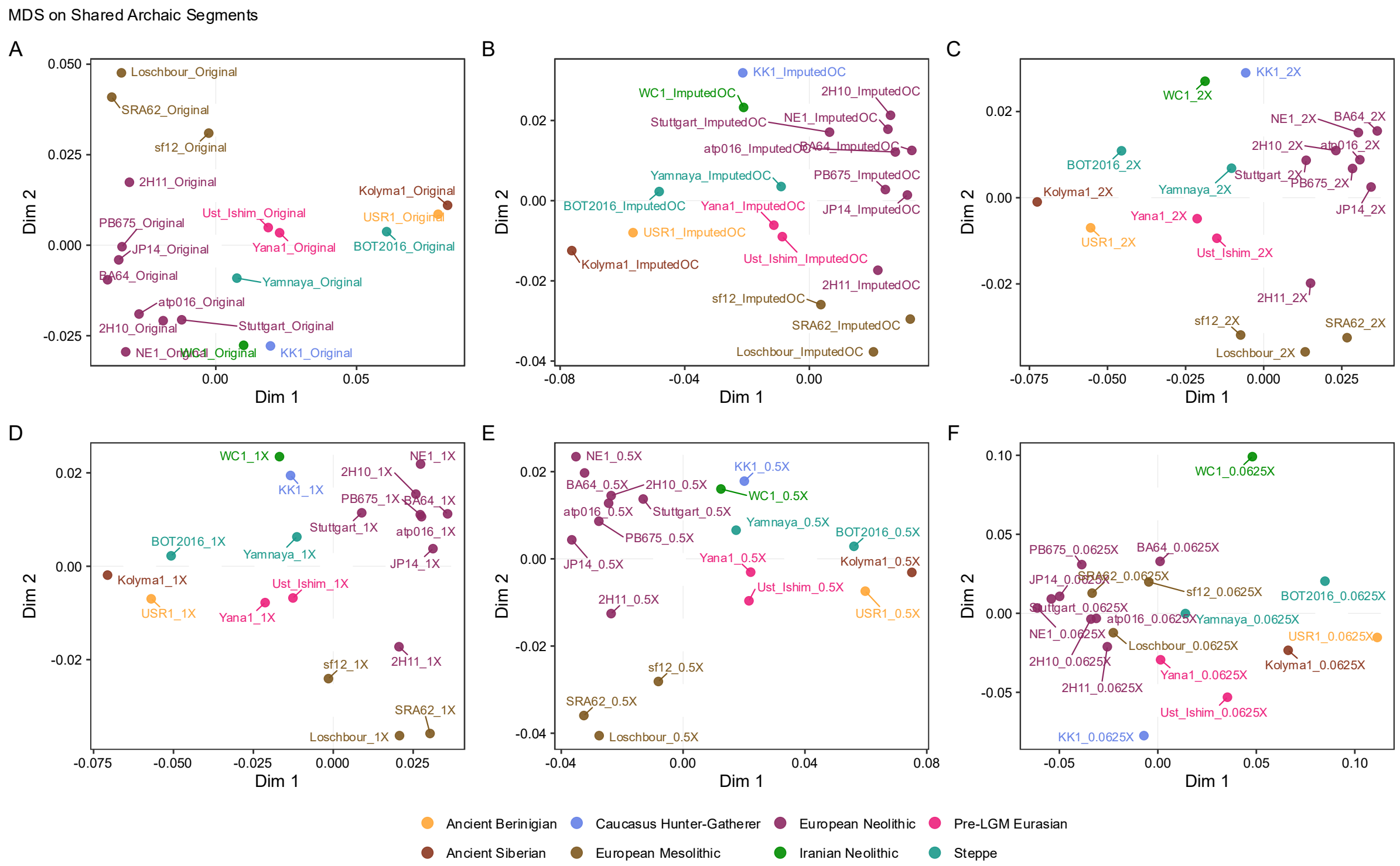

Supplementary Fig. 35. Multidimensional scaling (MDS) plots based on "Archaic Shared" segment count overlap of shared introgressed segments, shown separately for each coverage level: A) Original, B) Imputed OC, C) 2X, D) 1X, E) 0.5X, and F) 0.0625X. In this analysis the individual Mota has been excluded.

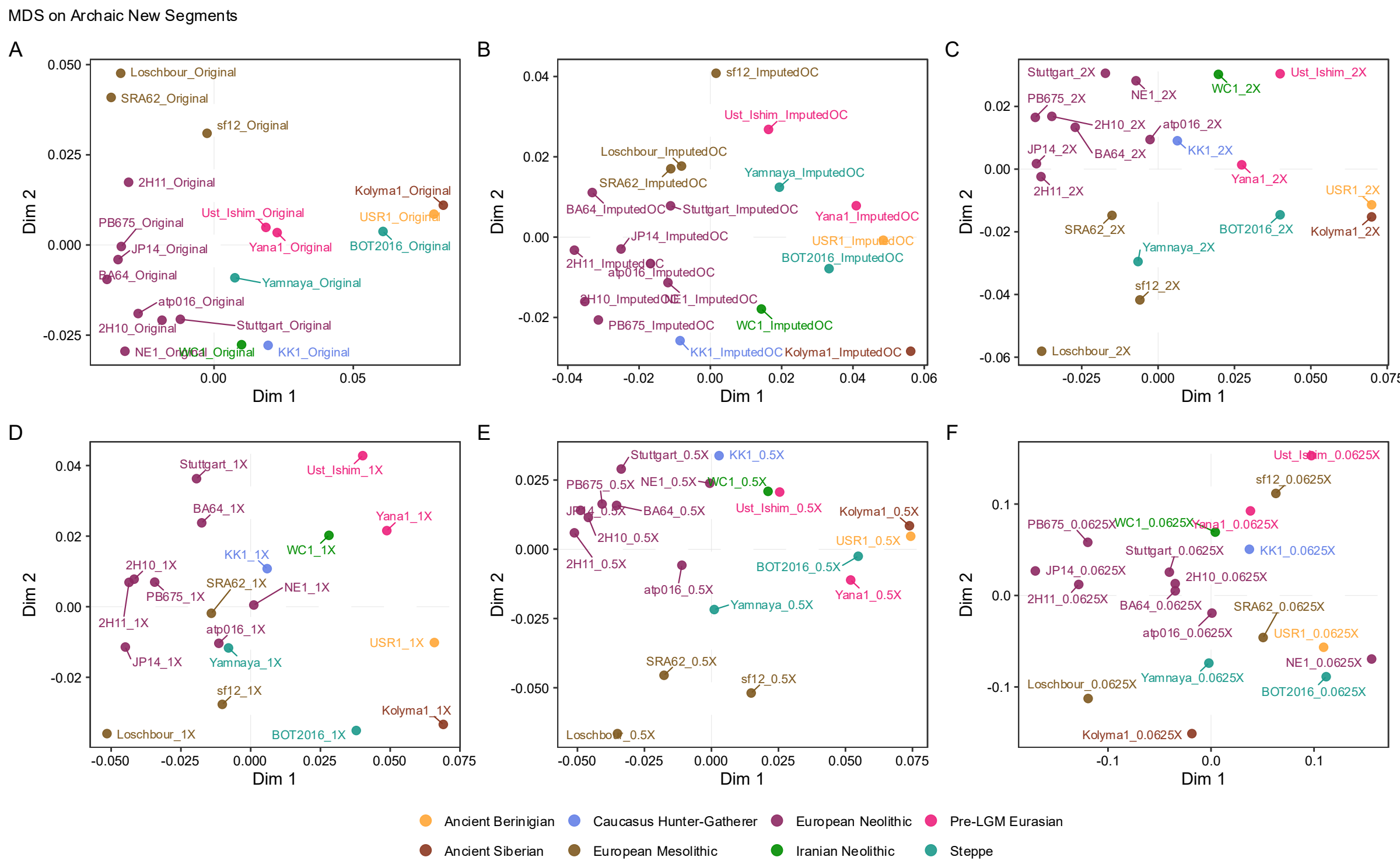

Supplementary Fig. 36. Multidimensional scaling (MDS) plots based on "Archaic New" segment count overlap of shared introgressed segments, shown separately for each coverage level: A) Original, B) Imputed OC, C) 2X, D) 1X, E) 0.5X, and F) 0.0625X. In this analysis the individual Mota has been excluded.

Finally, we tested whether population structure can be recovered when mixing individuals across coverage datasets (Supplementary Fig. 36). Even in this cross-coverage setting, after removing outliers—Mota and very-low-coverage (0.0625X) individuals—the expected structure is robustly reconstructed from introgressed segments.

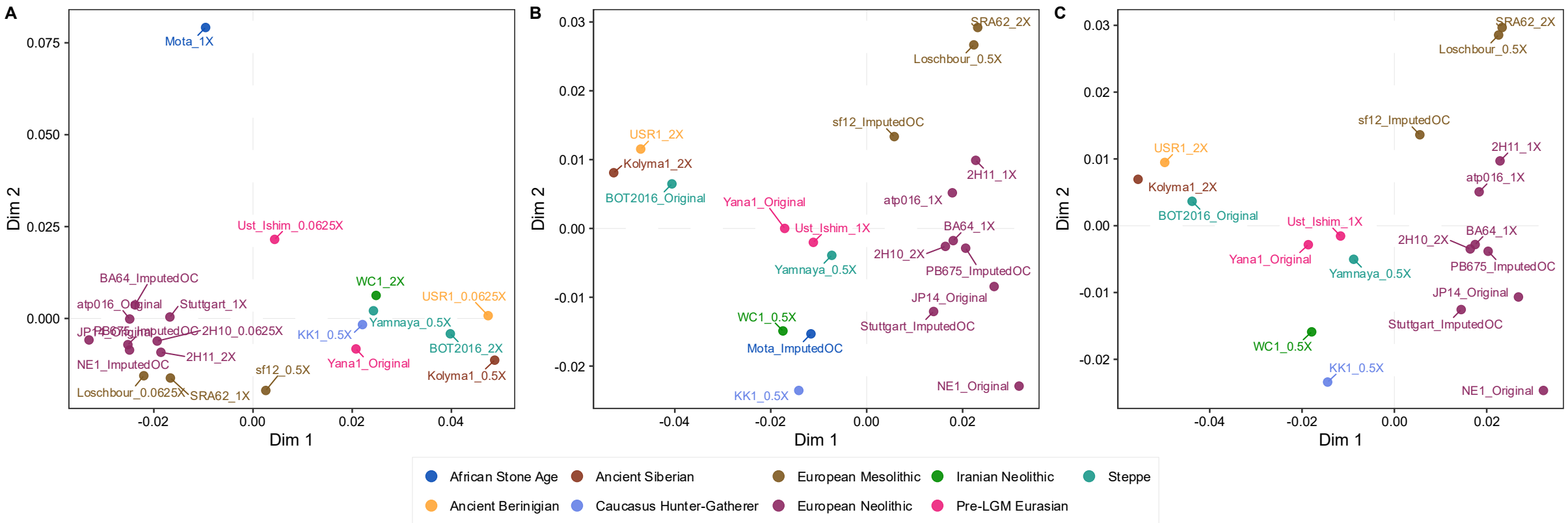

Supplementary Fig. 37. Multidimensional scaling (MDS) plots of shared introgressed segments across randomized individual-coverage combinations. (A) Includes all randomly assigned individuals and coverages. (B) Same procedure, excluding 0.0625X coverage samples. (C) Same procedure, excluding 0.0625X coverage samples and the Mota individual.

14. Analysis of Known Candidate Genes

To assess the presence of archaic introgressed fragments at previously proposed candidate loci, we curated a list of 14 genes of interest[32] and retrieved their genomic coordinates. Coordinates were mapped to the GRCh37 reference genome, and gene boundaries were extended to include their full transcriptional span. Using Ensembl BioMart, we confirmed the identity of overlapping protein-coding genes within these intervals. We then intersected these gene regions with archaic fragments previously identified through LAI, after quality filtering. This allowed us to determine whether individual fragments overlapped candidate genes, and to compute per-individual, per-gene summary statistics (Supplementary Data 3-4). For each gene, we recorded the average similarity and genetic distance to the archaic references across all overlapping fragments, stratified by coverage level and individual (Supplementary Data 4). Genes with no detected overlap were retained in the final output to distinguish true negatives from missing data. Results were summarized in a table indicating which individuals carried archaic segments at each gene, and how often such overlaps occurred across different sequencing coverages.

We extracted a subset of informative SNPs from the *BNC2* locus based on allele state comparisons among archaic, African, and chimpanzee genomes (Supplementary Data 5). Using vcfR, we read phased (imputed genotypes after filtering for GP > 99%) and unphased VCF files for archaic individuals (Altai Neanderthal, Vindija Neanderthal, Denisova, Chagyrskaya), African individuals (YRI, ESN, MSL), and a chimpanzee reference genome (panTro6). SNPs were retained if they fulfilled two conditions: (i) at least one archaic genome carried the derived allele, and (ii) all African individuals were homozygous for the ancestral allele. Ancestral and derived alleles were inferred based on chimpanzee genotypes (Supplementary Data 5). We then extracted genotypes for these filtered sites from the original ancient genomes and in the five imputed datasets for the individual BA64. Genotypes were converted into haplotype labels ("Ancestral", "Derived", "Heterozygous", "or "Missing") based on their match to the inferred allele states.

To visualize haplotype structure, we constructed a matrix where each row represented a haplotype or diploid individual and each column a filtered SNP. In the figure each allele states is encoded by color (Figure 4C).

15. Selection Scan on Imputed and Non Imputed European Genomes

To investigate signals of selection in introgressed regions, we conducted a genome-wide scan for overrepresented archaic fragments in ancient (Mesolithic and Neolithic) European individuals for the original genome and the five imputed datasets. We focused on segments previously identified with the LAI as likely archaic in origin after filtering for the length (>40Kb) and the similarity to reference Neanderthal and Denisovan genomes above 0.99. Introgressed fragments were projected across the genome and divided into discrete non-overlapping intervals based on their breakpoints. For each coverage level, we quantified the frequency of archaic segment presence across individuals, producing a binary presence/absence matrix per genomic interval, like the output of multiintersect in *bedtools*. Z-scores were computed for each interval to measure deviations from the mean segment-sharing frequency, and one-tailed p-values were calculated to identify intervals with significantly elevated archaic segment sharing. Bonferroni correction was applied to account for multiple testing. We retained regions where at least one interval showed significant enrichment (adjusted *p*-value < 0.01) and subsequently expanded these to include adjacent intervals with suggestive evidence (adjusted *p*-value < 0.05), allowing for the identification of broader genomic regions potentially affected by selection and due to the length uncertain that there is with ancient DNA. This "anchored" expansion strategy aimed to capture extended signals beyond single isolated peaks. To annotate the candidate regions, we queried the Ensembl database (GRCh37) and identified overlapping protein-coding genes. We tracked whether genes overlapped "core" significant regions (strict threshold) or extended regions (anchored by core segments), and assessed coverage-specific recurrence of gene hits (Supplementary Table 3, Supplementary Data 6). Finally, we summarized the presence of these genes across coverage levels and visualized the results in Manhattan plots with Z-scores and gene annotations stratified by coverage (Figure 5).

Supplementary Table 3. Presence of significant genes across coverages in core and extended introgressed regions. The table lists all protein-coding genes overlapping significant archaic-introgressed segments, categorized into core and extended regions. Core regions are defined as segments where at least one subregion has a Bonferroni-adjusted one-tailed *p*-value < 0.01 (testing for elevated archaic segment sharing with Bonferroni correction for multiple testing). Extended regions are composed of adjacent segments that do not meet the core significance threshold individually (*p_adj*_bonferroni ≥ 0.01), but are contiguous with core segments and show elevated frequencies (*p_adj* < 0.05), suggesting potential broader introgression.

Gene	Original	Imputed OC	2X	1X	0.5X	0.0625X
AKAP13	core					
ALPK3		extended	core	core	core	
CHFR			core			
CSMD2					core	
GOLGA3			core			
LARS						core
LEMD2		extended	core	extended	extended	
MCM3		extended				
MLN		extended	core	extended	extended	

NMB	extended	core	core	core	core	
NUCB2		extended				
PIK3C2A		extended				
PLAC8L1						core
RBM27						core
RPS13		extended				
SEC11A	extended	extended	extended	extended	core	
SGCZ	core		core			
SH3RF2						core
SLC28A1		extended	core	extended		
WDR73	extended	core	core	core	core	
ZNF592	extended	extended	extended	extended	extended	
ZSCAN2	core	core	core	core	core	

We then directly tested the genotypes of one of the two extended regions where we found a significant overrepresentation of archaic segments in Europeans. Since the region on chromosome 15 contains genes already identified as candidates for adaptive introgression, we focused on the region on chromosome 6, as this region was not significant in the original dataset. We concentrated on the region from the beginning of the *LEMD2* gene and the end of *MLN*. This region is located in the 6p21.3 band, which includes the MHC, a region known for its very high variability. As for *BCN2*, we reconstructed a heatmap showing positions where Africans are homozygous for the ancestral allele (defined using the Chimp genome), and at least one of the four high-coverage archaic genomes has a derived allele. We identified 20 positions, of which only three are not present in the 1000 Genomes Project strict mask (Supplementary Data 7). In the plot, the chimpanzee and African rows represent the ancestral allele, while the archaic high-coverage and original non-imputed genotypes are colored based on the presence of the ancestral (white), derived (green), heterozygous (yellow), and missing (grey) alleles. If the cell is green or white, it indicates homozygosity for the derived or ancestral allele, respectively. Next, we plotted phased imputed genomes from European (Supplementary Fig. 38A) and non-European (Supplementary Fig. 38B) individuals. Each row represents a haplotype (single allele, not the genotype), so when an individual is heterozygous, both haplotypes are considered. What we observed is that in the original data, despite a large number of missing positions, all European individuals have at least one derived allele, except for Stuttgart (Supplementary Fig. 38A). Only the individual sf12 is homozygous for the derived allele. We observed the same situation in all imputed datasets with coverage up to 0.5X, with the missing positions being resolved as haplotypes from Vindija Neanderthal, but not from the other archaic humans. All individuals were heterozygous, except for sf12, and no derived alleles were present in Stuttgart. This means that 10 out of 11 Europeans in our dataset show a derived allele in the original data, which we can resolve as an archaic haplotype in the imputed data. In contrast, when considering the non-European individuals (Supplementary Fig. 38B), none of them has a derived allele in the original data or an archaic haplotype in the imputed genomes. This illustrates that without imputation, this signal is not significant, primarily due to missing positions. However, with imputation, we can detect the signal and reconstruct the haplotypes.

In the 1000 Genomes Project individuals, this haplotype occurs at relatively high frequency in Europeans (~25%) and in the Americas (~15%), and is less represented in other geographic regions (Africa ~2%, confined to admixed individuals; East Asia ~3%; South Asia ~9%).

LEMD2 (LEM domain nuclear envelope protein 2). Encodes an inner nuclear membrane protein with a LEM domain (LAP2–Emerin–MAN1 family). *LEMD2* interacts with chromatin and lamins and is implicated in nuclear envelope integrity, chromatin organization, gene regulation, and signaling. Variants have been associated with cardiomyopathy, cataracts, and other laminopathies.

MLN (motilin). Encodes the motilin peptide hormone, secreted by the small intestine, which regulates gastrointestinal motility, including rhythmic contractions and gastric emptying. It is produced as prepro-motilin and processed to the mature hormone. Alterations in motilin signaling are mainly linked to gastrointestinal motility disorders. Motilin release during the fasted, interdigestive state triggers gastric phase III of the migrating motor complex, which is associated with the "return of hunger" after a meal[39]. Administration of motilin agonists (e.g. erythromycin) increases hunger ratings in experimental settings[40]. Further analyses including a larger number of ancient individuals and the application of explicit selection tests are needed to determine whether this region was truly under selection in the past. Such analyses would also help to reconstruct the haplotype trajectory and to understand the selective advantage that may have driven its increase before the Last Glacial Maximum and the Neolithic period, followed by its decline to the frequencies observed in present-day individuals.

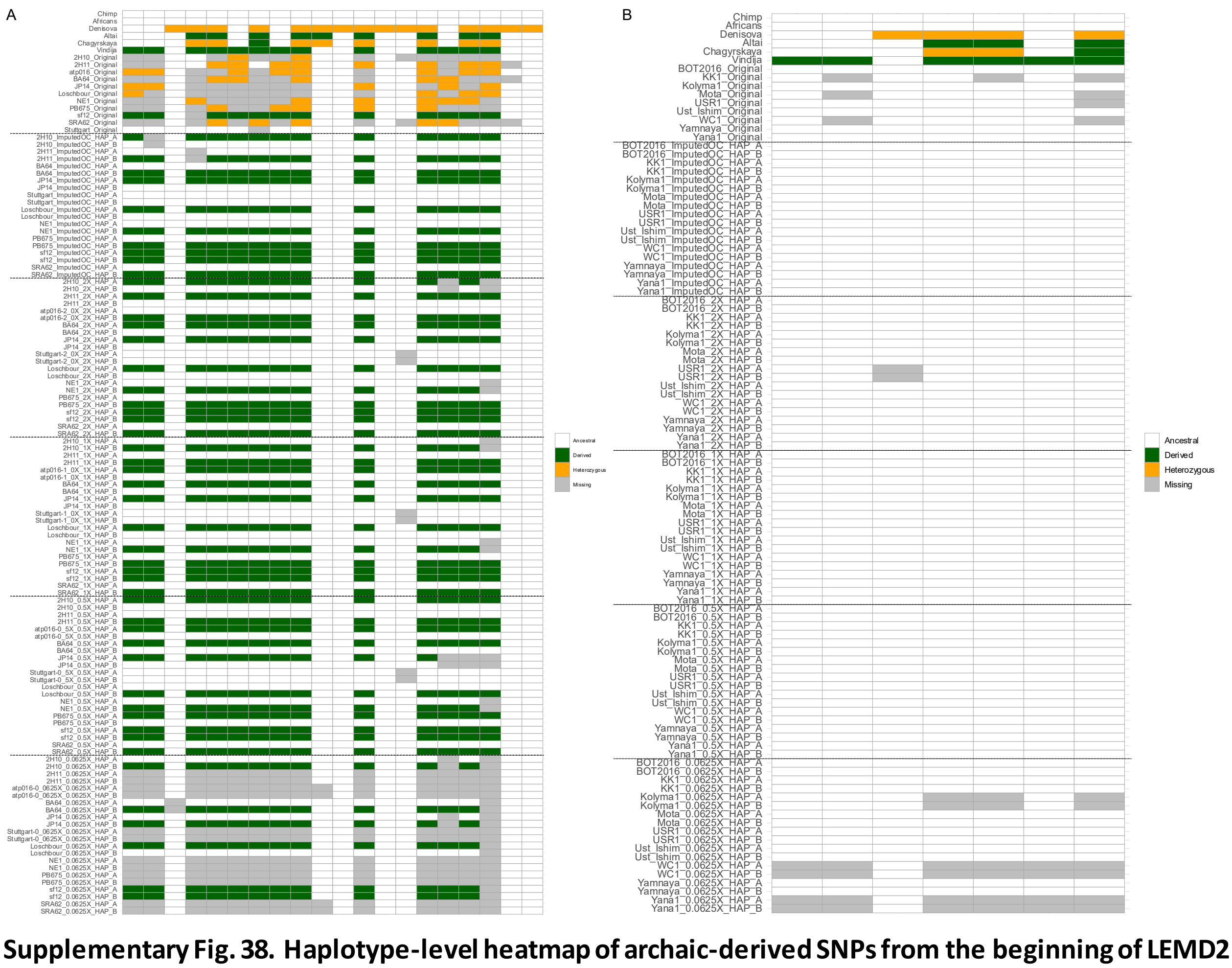

Supplementary Fig. 38. Haplotype-level heatmap of archaic-derived SNPs from the beginning of LEMD2 to the end of MLN genes region across chimpanzee, African, archaic, and non-imputed and imputed ancient human genomes after filtering for GP > 99%. A) for European individuals and B) for not european

individuals. Each row represents a haplotype or diploid individual, and each column corresponds to a possible derived allele. Alleles are classified as Ancestral (white), Derived (green), Heterozygous (orange) and Missing (grey). Imputed genomes are split into phased haplotypes (HAP_A and HAP_B), while archaic and original genomes are unphased.

Supplementary References

1. Seguin-Orlando, A. *et al.* Heterogeneous Hunter-Gatherer and Steppe-Related Ancestries in Late Neolithic and Bell Beaker Genomes from Present-Day France. *Curr Biol* **31**, 1072–1083.e10 (2021).
2. Valdiosera, C. *et al.* Four millennia of Iberian biomolecular prehistory illustrate the impact of prehistoric migrations at the far end of Eurasia. *Proc Natl Acad Sci U S A* **115**, 3428–3433 (2018).
3. Broushaki, F. *et al.* Early Neolithic genomes from the eastern Fertile Crescent. *Science* **353**, 499–503 (2016).
4. Cassidy, L. M. *et al.* Neolithic and Bronze Age migration to Ireland and establishment of the insular Atlantic genome. *Proc Natl Acad Sci U S A* **113**, 368–373 (2016).
5. Cassidy, L. M. *et al.* A dynastic elite in monumental Neolithic society. *Nature* **582**, 384–388 (2020).
6. de Barros Damgaard, P. *et al.* The first horse herders and the impact of early Bronze Age steppe expansions into Asia. *Science* **360**, (2018).
7. Fu, Q. *et al.* Genome sequence of a 45,000-year-old modern human from western Siberia. *Nature* **514**, 445–449 (2014).
8. Gallego Llorente, M. *et al.* Ancient Ethiopian genome reveals extensive Eurasian admixture throughout the African continent. *Science* **350**, 820–822 (2015).
9. Gamba, C. *et al.* Genome flux and stasis in a five millennium transect of European prehistory. *Nat. Commun.* **5**, 5257 (2014).
10. Günther, T. *et al.* Population genomics of Mesolithic Scandinavia: Investigating early postglacial migration routes and high-latitude adaptation. *PLoS Biol* **16**, e2003703

(2018).

11. Jones, E. R. *et al.* Upper Palaeolithic genomes reveal deep roots of modern Eurasians. *Nat Commun* **6**, 8912 (2015).
12. Lazaridis, I. *et al.* Ancient human genomes suggest three ancestral populations for present-day Europeans. *Nature* **513**, 409–413 (2014).
13. Moreno-Mayar, J. V. *et al.* Terminal Pleistocene Alaskan genome reveals first founding population of Native Americans. *Nature* **553**, 203–207 (2018).
14. Sikora, M. *et al.* The population history of northeastern Siberia since the Pleistocene. *Nature* **570**, 182–188 (2019).
15. Li, H. & Durbin, R. Fast and accurate short read alignment with Burrows-Wheeler transform. *Bioinformatics* **25**, 1754–1760 (2009).
16. Danecek, P. *et al.* Twelve years of SAMtools and BCFtools. *Gigascience* **10**, (2021).
17. 1000 Genomes Project Consortium *et al.* A global reference for human genetic variation. *Nature* **526**, 68–74 (2015).
18. Rubinacci, S., Ribeiro, D. M., Hofmeister, R. J. & Delaneau, O. Efficient phasing and imputation of low-coverage sequencing data using large reference panels. *Nat. Genet.* **53**, 120–126 (2021).
19. Sousa da Mota, B. *et al.* Imputation of ancient human genomes. *Nature Communications* **14**, 1–17 (2023).
20. Korneliussen, T. S., Albrechtsen, A. & Nielsen, R. ANGSD: Analysis of Next Generation Sequencing Data. *BMC Bioinformatics* **15**, 356 (2014).
21. Prüfer, K. *et al.* The complete genome sequence of a Neanderthal from the Altai Mountains. *Nature* **505**, 43–49 (2014).
22. Prüfer, K. *et al.* A high-coverage Neandertal genome from Vindija Cave in Croatia.

Science **358**, 655–658 (2017).

23. Mafessoni, F. *et al.* A high-coverage Neandertal genome from Chagyrskaya Cave. *Proc. Natl. Acad. Sci. U. S. A.* **117**, 15132–15136 (2020).
24. Meyer, M. *et al.* A high-coverage genome sequence from an archaic Denisovan individual. *Science* **338**, 222–226 (2012).
25. Patterson, N. *et al.* Ancient admixture in human history. *Genetics* **192**, 1065–1093 (2012).
26. Peede, D., Vecchyo, D. O.-D. & Huerta-Sánchez, E. The Utility of Ancestral and Derived Allele Sharing for Genome-Wide Inferences of Introgression. *bioRxiv* 2022.12.02.518851 (2022) doi:10.1101/2022.12.02.518851.
27. Skov, L. *et al.* Detecting archaic introgression using an unadmixed outgroup. *PLoS Genet.* **14**, e1007641 (2018).
28. Witt, K. E., Funk, A., Añorve-Garibay, V., Fang, L. L. & Huerta-Sánchez, E. The Impact of Modern Admixture on Archaic Human Ancestry in Human Populations. *Genome Biol. Evol.* **15**, (2023).
29. Ringbauer, H. *et al.* Accurate detection of identity-by-descent segments in human ancient DNA. *Nat. Genet.* **56**, 143–151 (2024).
30. Iasi, L. N. M. *et al.* Neanderthal ancestry through time: Insights from genomes of ancient and present-day humans. *Science* **386**, eadq3010 (2024).
31. Huerta-Sánchez, E. *et al.* Altitude adaptation in Tibetans caused by introgression of Denisovan-like DNA. *Nature* **512**, 194–197 (2014).
32. Ongaro, L. & Huerta-Sanchez, E. A history of multiple Denisovan introgression events in modern humans. *Nat Genet* **56**, 2612–2622 (2024).
33. Yuan, K. *et al.* Refining models of archaic admixture in Eurasia with ArchaicSeeker 2.0.

Nat Commun **12**, 6232 (2021).

34. Reilly, P. F., Tjahjadi, A., Miller, S. L., Akey, J. M. & Tucci, S. The contribution of Neanderthal introgression to modern human traits. *Curr. Biol.* **32**, R970–R983 (2022).
35. Kimura, M. SOLUTION OF A PROCESS OF RANDOM GENETIC DRIFT WITH A CONTINUOUS MODEL. *Proc Natl Acad Sci U S A* **41**, 144–150 (1955).
36. Malaspinas, A. S. *et al.* A genomic history of Aboriginal Australia. *Nature* **538**, (2016).
37. Vernot, B. *et al.* Excavating Neandertal and Denisovan DNA from the genomes of Melanesian individuals. *Science* **352**, 235–239 (2016).
38. Mallick, S. *et al.* The Simons Genome Diversity Project: 300 genomes from 142 diverse populations. *Nature* **538**, 201–206 (2016).
39. Tack, J. *et al.* The gastrointestinal tract in hunger and satiety signalling. *United European Gastroenterol. J.* **9**, 727–734 (2021).
40. Zhao, D. *et al.* The motilin agonist erythromycin increases hunger by modulating homeostatic and hedonic brain circuits in healthy women: a randomized, placebo-controlled study. *Sci. Rep.* **8**, 1819 (2018).
