# Supplementary material for: Archaic ancestry inference in imputed ancient human genomes
Source: Nat Commun. 2026 Aug 6;17:9480. doi: 10.1038/s41467-026-76204-0 (PMC13542231; doi:10.1038/s41467-026-76204-0)
Supplement: Supplementary file 2 [file 41467_2026_76204_MOESM2_ESM.pdf]

Description of Additional Supplementary Files

Supplementary Data 1: Metadata for the ancient individuals analyzed in this study.

Supplementary Data 2: Metadata for the African genomes used as unadmixed reference.

Supplementary Data 3: Summary of individual counts per coverage level with overlapping introgressed fragments in 14 known candidate genes.

Supplementary Data 4: Per-sample overlap of archaic introgressed fragments with 14 known candidate genes.

Supplementary Data 5: List of SNPs used to investigate archaic introgression at the BNC2 locus. Each site is biallelic and annotated with the reference (REF), alternative (ALT), and inferred ancestral and derived alleles. Only SNPs where archaic genomes carry the derived allele and African genomes are homozygous for the ancestral allele were retained.

Supplementary Data 6: Segment-level annotation of introgressed genes across core and extended regions. This table lists all protein-coding genes overlapping archaic introgressed segments across all coverage levels.

Supplementary Data 7: List of SNPs used to investigate archaic introgression from the start of the LEDM2 gene to the end of MLN gene. Each site is biallelic and annotated with the reference (REF), alternative (ALT), and inferred ancestral and derived alleles. Only SNPs where archaic genomes carry the derived allele and African genomes are homozygous for the ancestral allele were retained. In the last column is reported if the position is in the Strict Mask region.

Supplementary Data 8: Paired Wilcoxon signed-rank test (two-sided) comparing per-individual similarity to the nearest archaic genome between Archaic and Non-Archaic segments across coverage levels.
