# Supplementary material for: Archaic ancestry inference in imputed ancient human genomes
Source: Nat Commun. 2026 Aug 6;17:9480. doi: 10.1038/s41467-026-76204-0 (PMC13542231; doi:10.1038/s41467-026-76204-0)
Supplement: Supplementary file 3 [file 41467_2026_76204_MOESM4_ESM.pdf]

Corresponding author(s): Marco Rosario Capodiferro
Léo Planche
Emilia Huerta-Sanchez

Last updated by author(s): 20/04/2026

Reporting Summary

Nature Portfolio wishes to improve the reproducibility of the work that we publish. This form provides structure for consistency and transparency in reporting. For further information on Nature Portfolio policies, see our Editorial Policies and the Editorial Policy Checklist.

Statistics

For all statistical analyses, confirm that the following items are present in the figure legend, table legend, main text, or Methods section.

n/a	Confirmed	
☐	☒	The exact sample size (*n*) for each experimental group/condition, given as a discrete number and unit of measurement
☐	☒	A statement on whether measurements were taken from distinct samples or whether the same sample was measured repeatedly
☐	☒	The statistical test(s) used AND whether they are one- or two-sided<br>*Only common tests should be described solely by name; describe more complex techniques in the Methods section.*
☒	☐	A description of all covariates tested
☐	☒	A description of any assumptions or corrections, such as tests of normality and adjustment for multiple comparisons
☐	☒	A full description of the statistical parameters including central tendency (e.g. means) or other basic estimates (e.g. regression coefficient) AND variation (e.g. standard deviation) or associated estimates of uncertainty (e.g. confidence intervals)
☐	☒	For null hypothesis testing, the test statistic (e.g. F, t, r) with confidence intervals, effect sizes, degrees of freedom and P value noted<br>*Give P values as exact values whenever suitable.*
☒	☐	For Bayesian analysis, information on the choice of priors and Markov chain Monte Carlo settings
☒	☐	For hierarchical and complex designs, identification of the appropriate level for tests and full reporting of outcomes
☒	☐	Estimates of effect sizes (e.g. Cohen's *d*, Pearson's *r*), indicating how they were calculated

Our web collection on statistics for biologists contains articles on many of the points above.

Software and code

Policy information about availability of computer code

Data collection	No software was used for the data collection of this study

Data analysis	bwa 0.7.17<br>GATK 4.4.0.0<br>SAMtools 1.7<br>Glimpse 1.1.0<br>ANGSD 0.940<br>bcftools 1.20<br>Plink 1.9<br>AdmixTool 7.0.2<br>RIPTA<br>hmmix<br>Picard 3.0.0<br>R 4.5.2<br>Python 3.11.7<br><br>All other scripts required to reproduce the figures and tables are available at DOI: 10.5281/zenodo.16365479, and updates will be released via the following GitHub repository:<br>https://github.com/LeoPlanche/arcAncientImputed

For manuscripts utilizing custom algorithms or software that are central to the research but not yet described in published literature, software must be made available to editors and reviewers. We strongly encourage code deposition in a community repository (e.g. GitHub). See the Nature Portfolio guidelines for submitting code & software for further information.

Data

Policy information about availability of data

All manuscripts must include a data availability statement. This statement should provide the following information, where applicable:

- Accession codes, unique identifiers, or web links for publicly available datasets
- A description of any restrictions on data availability
- For clinical datasets or third party data, please ensure that the statement adheres to our policy

This study does not generate new sequencing data. References to the original published sequencing datasets are provided in Supplementary Data 1. The ancient individual genotypes (original and imputed, VCF format) and introgression maps generated in this study are available on Zenodo [39] (https://doi.org/10.5281/zenodo.16365478) and on GitHub (https://github.com/LeoPlanche/arcAncientImput). The modern reference panel used was the 1000 Genomes Project Phase III dataset, available at https://ftp.1000genomes.ebi.ac.uk/vol1/ftp/release/20130502/. Archaic genome genotypes were downloaded from the MPI-EVA FTP server https://ftp.eva.mpg.de/neandertal/Vindija/VCF/ and https://ftp.eva.mpg.de/neandertal/Chagyrskaya/VCF/. Source data are provided with this paper and are also available on the Zenodo repository [39].

Research involving human participants, their data, or biological material

Policy information about studies with human participants or human data. See also policy information about sex, gender (identity/presentation), and sexual orientation and race, ethnicity and racism.

Reporting on sex and gender	We analysed only the autosomal chromosomes.
Reporting on race, ethnicity, or other socially relevant groupings	N/A
Population characteristics	Twenty publicly available ancient genomes from different geographic regions were included: 1 from Africa, 1 from the Americas, 11 from Europe, 5 from West Asia, and 2 from the Steppe.<br><br>All individuals from the 1000 Genomes Project were used.
Recruitment	N/A
Ethics oversight	N/A

Note that full information on the approval of the study protocol must also be provided in the manuscript.

Field-specific reporting

Please select the one below that is the best fit for your research. If you are not sure, read the appropriate sections before making your selection.

[x] Life sciences [ ] Behavioural & social sciences [ ] Ecological, evolutionary & environmental sciences

For a reference copy of the document with all sections, see nature.com/documents/nr-reporting-summary-flat.pdf

Life sciences study design

All studies must disclose on these points even when the disclosure is negative.

Sample size	We analysed 20 ancient genomes with coverage greater than 10×, which were subsequently downsampled.<br>A total of 2,504 individuals from the 1000 Genomes Project were included.
Data exclusions	We analysed only the 22 autosomal chromosomes and the ~78 million biallelic SNPs present in the 1000 Genomes Project Phase 3 dataset.For some analyses, we restricted the SNPs to sites with a genotype probability >0.99 and a minor allele frequency (MAF) of at least 5% in the 1000 Genomes Project Phase 3 dataset.
Replication	All data and software are publicly available, and the analyses can be replicated using the scripts deposited in the Zenodo repository (DOI: 10.5281/zenodo.16365479) and the GitHub repository:https://github.com/LeoPlanche/arcAncientImputed
Randomization	No experimental group allocation was performed.
Blinding	This aspect is not relevant to this study.

Reporting for specific materials, systems and methods

We require information from authors about some types of materials, experimental systems and methods used in many studies. Here, indicate whether each material, system or method listed is relevant to your study. If you are not sure if a list item applies to your research, read the appropriate section before selecting a response.

Materials & experimental systems

n/a	Involved in the study
☒	☐ Antibodies
☒	☐ Eukaryotic cell lines
☐	☒ Palaeontology and archaeology
☒	☐ Animals and other organisms
☒	☐ Clinical data
☒	☐ Dual use research of concern
☒	☐ Plants

Methods

n/a	Involved in the study
☒	☐ ChIP-seq
☒	☐ Flow cytometry
☒	☐ MRI-based neuroimaging

Palaeontology and Archaeology

Specimen provenance	All the specimens have been analysed in previous studies.
Specimen deposition	N/A
Dating methods	N/A

☐ Tick this box to confirm that the raw and calibrated dates are available in the paper or in Supplementary Information.

Ethics oversight	N/A

Note that full information on the approval of the study protocol must also be provided in the manuscript.

Plants

Seed stocks	*Report on the source of all seed stocks or other plant material used. If applicable, state the seed stock centre and catalogue number. If plant specimens were collected from the field, describe the collection location, date and sampling procedures.*
Novel plant genotypes	*Describe the methods by which all novel plant genotypes were produced. This includes those generated by transgenic approaches, gene editing, chemical/radiation-based mutagenesis and hybridization. For transgenic lines, describe the transformation method, the number of independent lines analyzed and the generation upon which experiments were performed. For gene-edited lines, describe the editor used, the endogenous sequence targeted for editing, the targeting guide RNA sequence (if applicable) and how the editor was applied.*
Authentication	*Describe any authentication procedures for each seed stock used or novel genotype generated. Describe any experiments used to assess the effect of a mutation and, where applicable, how potential secondary effects (e.g. second site T-DNA insertions, mosiacism, off-target gene editing) were examined.*
