# Supplementary material for: Archaic ancestry inference in imputed ancient human genomes
Source: Nat Commun. 2026 Aug 6;17:9480. doi: 10.1038/s41467-026-76204-0 (PMC13542231; doi:10.1038/s41467-026-76204-0)
Supplement: Supplementary file 4 [file 41467_2026_76204_MOESM5_ESM.pdf]

nature portfolio

Peer Review File

Archaic ancestry inference in imputed ancient human genomes

Corresponding Author: Dr Marco Rosario Capodiferro

This file contains all reviewer reports in order by version, followed by all author rebuttals in order by version.

Version 0:

Reviewer comments:

Reviewer #1

(Remarks to the Author)
This study focuses on using genotype imputation to identify archaic introgressed regions in ancient genomes. The authors thoroughly benchmark their approach by first downsampling high-coverage ancient genomes and comparing the results of their analyses with the original ground truth calls. Using allele-frequency- and haplotype-based methods, the authors demonstrate that they can recover most of the archaic regions in ancient individuals' genomes. Furthermore, they detected even more archaic segments that they found in their ground truth dataset, which is impacted by some amount of missingness, postmortem damage and sequencing errors. A shortcoming of this approach is that the true lengths of the segments may not be recovered, particularly for the older ancient genomes, which hinders estimates of introgression time. To assess whether these imputed segments are shared with present-day populations, the authors compare them against present-day reference datasets. Depending on the ancient individuals' age and ancestry, the segments show varying levels of sharing with different modern populations.

They also found that Neanderthal-specific variants can be accurately imputed, and that they are imputed more accurately than non-archaic variants. As a consequence, the genotype posteriors (GP, a measure of how confidently imputed a variant is and is well calibrated with error rate) for these variants tend to be greater than for non-archaic variants. As a result, when applying strict quality control filters as GP>99%, they observed a bias in D-statistics. This is an important observation, because, depending on how well different ancestries can be imputed, strict GP filtering can introduce bias.They then adapted D-statistics calculations to take imputed genotype dosages as input instead of hard calls, which is a promising approach for many other methods.

Finally, the authors apply their method to gain biological insights. Through enrichment analyses, they identify genes enriched for archaic haplotypes. This analysis replicates known introgression signals and also uncovers three new candidate genes.

In my view, this work is technically sound. It makes use of state-of-the-art methods in the field and is clearly described and logically structured (even though it needs some rewriting to be more concise, particularly in the discussion section). It is a valuable contribution to the ancient DNA community. To the best of my knowledge, the question of how well archaic introgressed regions can be recovered from imputed ancient genomes has not been systematically assessed before and it is of great interest in the field. I have some noncritical comments and suggestions, which I have listed below.

Major comments:

1) My first general comment is that I missed some direct comparisons with present-day data. For instance, for Figure 2D, I do not know what to expect regarding Denisovan segments in present-day Europeans. Also, with a high-quality high-coverage genome (downsampled to intermediate coverages to match the ancient data), it would be easier to pinpoint to what extent the detection of Archaic New segments is the result of missingness in the original coverage genome (and how many of these are potentially false positives). That said, I understand that suggesting new analyses is easier than performing them. For the first point in particular, I would be satisfied with an explicit, well-referenced sentence in the text to clarify expectations.

2) Upon generating the high-confidence dataset, did you apply any methods to reduce postmortem damage (PMD), such as read trimming? Generating a high-quality ground truth for ancient genomes is always challenging, as PMD can introduce

artificial differences between the imputed calls and the ground truth.

3) On page 7, it says “Moreover, considering only the filtered results (the bar with the black border in Figure 2A), the older individuals (shown on the left) have a higher proportion of their genome inferred to be of archaic origin across all coverage levels, consistent with expectations.” There are some fluctuations for total introgressed length (for example, BOT2016 seems to have more more introgressed segments (in length). Why do you think this is the case? One potential issue may come from measuring length in base pairs instead of centimorgans. Please convert these lengths to cM to better compare introgressed segment lengths across the different samples. Furthermore, please provide a similar figure to S8, where the total introgressed length is plotted against sample age.

4) In Figure 2B, after filtering, why do we observe a higher proportion of recovered introgressed segments in the imputed 1x or 2x genomes compared to the imputed OC genomes for most individual samples? Furthermore, in Figure S22, there are more significant hits in the imputed 2x dataset (10 genes in red and 2 in blue) than in the imputed OC dataset (10 in blue and 3 in red). Is this difference in the enrichment analysis driven by a difference in the number of detected introgressed segments?I have the intuition that PMD may have a greater impact when imputing higher coverage genomes, because genotype likelihoods can appear more confident when more reads cover a site, even if a substantial fraction of those reads carry deaminated bases at that position.

5) The authors show that imputation allows us to detect new archaic segments that we could not detect in the original high-coverage genome. Moreover, their filtering approach seems to be efficient at removing potential false positives, as shown in Figure S6. However, I would like to see further sanity checks. First, I suggest splitting the archaic segments into the two categories Archaic Shared and Archaic New. Then, we can apply the same approach as in Figures 4A and 4B to verify if these two types of introgressed segments share the same pattern with present-day populations (Figure 4A) and if they both preserve the expected population structure of the individual samples (Figure 4B). Furthermore, regarding the segments not found in present-day populations, are they enriched in one of the two categories?

6) To evaluate imputation performance in introgressed regions, the authors restricted the analysis to alleles absent in African populations and homozygous in the Vindija Neanderthal genome. While this is a reasonable approach to assess imputation performance for Neanderthal-introgressed regions, it does not inform us about how well Denisovan-specific SNPs or regions are imputed. Could you apply the same approach to evaluate this?

In that sense, the statement that archaic segments are more accurately imputed should be made more precise. In other words, when we read sentences as “One surprising result is that imputation performs better in archaic versus non-archaic regions and variants [...]” (Discussion), we should in fact read “imputation performs better in Neanderthal-introgressed regions”. I suspect Denisovan-specific variants may be more challenging to impute due to: (1) distinct archaic population histories, and (2) the smaller amount of Denisovan DNA present in the imputation reference panel.

7) In Figure 3A, how many SNPs does each bin have? In other words, are there wildly different numbers of SNPs in the low-frequency bins that would artificially drive differences in imputation accuracy? While it is good that the authors tried to make the archaic and non-archaic variants comparable by sampling non-archaic segments with the same length distribution, it might be even more important to ensure that these two sets of variants have similar allele frequency distribution, being one of the most impactful factors for imputation accuracy. What is the allele frequency distribution in 1000G of the archaic-specific SNPs? Additionally, please provide the raw data used to generate this plot (and also Figure 3B) as well as the number of SNPs per curve per bin and their average MAF in a supplementary table.

8) I would like to see a supplementary figure showing the imputation error rate (or preferably heterozygosity sensitivity or non-reference discordance) per individual across different coverage levels. The reason for this request is that Figure 3 presents aggregate imputation accuracy across individuals, which may mask variation due to ancestry. I am interested in whether the specific ancestry context plays a major role. For example, if a Neanderthal-introgressed segment is found in a well-represented genetic background (e.g., European), would it be more accurately imputed? Conversely, if I attempt to extract Neanderthal-introgressed segments from an ancient Papuan genome, should I expect similar performance, or would imputation accuracy drop due to limited representation in the reference panel?

9) On page 8 of the Supplementary Information file, the authors described how they classified introgressed segments in ancient individuals as shared with present-day individuals (Figure 4A). “We consider that two segments are matching if they overlap for 80% of their length.” I would like to understand the rationale behind choosing this threshold. How sensitive are the results to this 80% overlap threshold? I am concerned that this approach excludes segments with a smaller but still relevant amount of overlap. This could affect the number of segments attributed to a particular population and the segments found to be shared with none of the present-day individuals. Have you considered classifying shared segments based on the amount of overlap between them?

10) I suggest making Figure S22 part of the main article, as it illustrates how this approach can be applied and presents new results. For the new candidate introgressed genes, why have these not been found in present-day populations? Are these regions enriched in segments not found in present-day people (Figure 4A)? Additionally, could you assess the frequency at which these introgressed genes occur in present-day European populations?

Minor comments:

1) To have a full understanding of the data spread, it is good practice to overlay all the data points with jitter on box plots. I

would like to see these data points in figures containing box plots.

2) On page 5 under “Global Ancestry Inference[...]”, there is a typo in the f4-ratio expression. Do you mean f4(Archaic1, Chimp; Test, Africa) / f4(Archaic1, Chimp; Archaic2, Africa)? Furthermore, in the same context, please explicitly define alpha.

3) For Figures S2 and S8, I recommend plotting the log10 of the sample ages. As most individual samples are from more recent periods, this would allow to more easily visualize how the different variables are affected by sample age.

4) When mentioning specific individual samples in the text for the first time, please indicate the mean sample age. For example, on page 8, “We observed that Kolyma1 (Siberia, XXX ybp) and USR1 (Alaska, XXX ypb)”.

5) The sentence “While the Archaic Shared segments exhibit the highest mean similarity to the archaic reference genome, the Archaic New segments also show significantly higher similarity compared to regions classified as non-archaic (Figure 2C)” appears to use “significantly” in a qualitative sense, as I did not find any mention of a statistical test. If that is indeed the case, I recommend replacing “significantly” with a more appropriate term such as “considerably” or “substantially.”

That being said, based on the visual representation of the similarity distributions, it is difficult to assess whether the non-archaic distribution might have heavy tails or skewness toward high similarity values (although I trust the authors that the distributions are distinct). To support this claim more rigorously, please consider providing summary statistics, such as medians and quantiles, for each category to quantify the differences.

6) On page 8, “Notably, the Archaic New segments contain more missing positions in the original data.” This statement does not seem to be backed-up by any figure or quantification. How exactly missingness varies between Archaic New vs. Archaic Shared segments?

7) On page 8, “We measured the error rate of imputation in archaic SNPs, which is remarkably low at approximately 0.2% [...]”, please specify how you calculated the imputation error rate. Typically, for imputation experiments, one tends to focus on error rates at heterozygous sites or non-reference discordance. Please indicate what imputation error rate you found for non-archaic SNPs.

8) In Figure 4C, please add a label for the x axis, as well as the start and the end positions.

9) On page 13, the term “gene clusters” is used. Could you clarify what is meant by this? Are these genes physically close to each other in the genome (i.e., clustered by genomic location), or are they grouped based on functional similarity or shared biological pathways?

10) In the Supplementary Information file, page 8, under "Accuracy of archaic alleles vs MAF", you defined a genotype (“between 0 and 2”) as GP(1/1)+0.5*GP(0/1). However, this varies between 0 and 1. I suppose that to plot 1-MSE, you are scaling both imputed and the ground truth genotypes to vary between 0 and 1. This can be more explicitly described.

11) At the end of the same paragraph, it reads: “We then plot 1-MSE, which is equivalent to r^2 correlation.” If you calculate 1-MSE, please write that in the y-axis of your plots and not r^2, the squared Pearson correlation, which I would say are not strictly equivalent.

12) Across the main text, I have occasionally found it difficult to follow the details of the analytical procedures, as some key information appears to be missing or insufficiently explained, but included in the Supplementary Information file.
For example:
- “We kept segments [...] with a length above 40Kb; that is above the length needed to reject incomplete lineage sorting for regions with moderate recombination rates38.” This is an important justification for the filtering threshold. Please consider adding this explanation or at least the relevant reference to the main text.
- “We say that a segment found in the original dataset has also been found in an imputed dataset, if at least 80% of the segment overlaps a segment found with the imputed dataset.” As a reader, I need a clearer explanation of how segment classification and overlap matching are defined and applied in the main text.

On the other hand, I noticed that some parts of the main text explicitly mention standard tools (e.g., admixtools) that might be more appropriate for the Methods section. I suggest relocating such technical details to the Methods section and simply cite the relevant publication in the main text.

13) My final suggestion is to split Materials and Methods into Materials and Methods and Supplementary Notes (to be referenced in the main text) when appropriate, as there is important discussion of analyses and approaches mixed with methodology descriptions.

(Remarks on code availability)

Reviewer #2

(Remarks to the Author)
The authors study the use of imputation on downsampled ancient genomes to assess the application of imputation with the

specific aim to identify areas of archaic integressions. I find the work to be thorough and compelling. The choice of methods is sound and the presentation in terms of figures and experiments is highly convincing.

One possible weakness would be the extent to which it is possible to use solely modern references. I have long been an advocate of this for aDNA imputation in general, but there could still be doubts. This especially applies to the (otherwise interesting) analysis of unshared segments in Ust'Ishim. In this context, I would have been curious on a discussion on to what extent it would be possible to augment the reference set with fully archaic genomes (possibly themselves imputed, depending on practical constraints on the nature of a reference). This would not replace the use of a fully modern reference, but the addition of a single or a few highly distinct genomes that would better cover archaic segments heavily diluted or missing in modern genomes would illustrate the relative stability of the results, beyond the comparison already done based on the comparison between imputed and original reads.

I do have several concerns about the article outline. The abstract is almost 800 words, while Nature Communications seem to indicate a limit of 200 words. The section "Results and discussion" has a somewhat misleading title, which could rather be "Results and Methods". The proper author instructions for Nature Communications do indicate the need for a specific Methods section. The subsection structure could also be enhanced. When reading the manuscript in full, I was highly convinced, but I found it challenging when I wanted to go back and check some detail to locate where it was actually mentioned. I also think the choice of references is somewhat incosistent.

(Remarks on code availability)
The code provided only covers a limited part of the total context, creating final summary statistics.

The code is far from enough to reproduce the results in the paper. The running of the imputation process itself (using the pre-existing GLIMPSE tool) isn't covered at all, for example. Some resulting data is probided in CSV form.

Reviewer #3

(Remarks to the Author)
General comments:

The imputation in ancient genomes was shown to work well given a certain MAF > 5%, coverage > 0.5 x and closeness to the reference panel (Sousa da Mota 2023) and is done frequently now. However, it was never assessed how it affects the archaic ancestry in the imputed individuals. The manuscript closes this gap and focuses on the imputation of archaic ancestry in ancient genomes. Ancient genomes can often only be sequenced to very low coverage since the endogenous amount of DNA is very low. A common method to overcome this is to enrich the endogenous amount of DNA by only capturing specific sites in the genome. The manuscript concludes that archaic ancestry can be imputed for shotgun genomes even better compared to random sites and to lower coverage. The manuscript is concerned with this very significant question and there has been a lot of work put into it. It touches on multiple things: testing imputation and subsequent global and local ancestry inference. Where the local inference is the main focus using hmmix. Most of the evaluation seems to be done on Neandertal ancestry (since most genomes are West-Eurasians without Denisovan ancestry). Denisovan ancestry is checked. Then the authors conclude that population structure is preserved after imputation. Finally, the imputed genomes were used for searching for candidates of adaptive introgression, for which two new candidate genes were found. My main comment is that I am not quite sure about the scope of this manuscript and feel the manuscript could benefit from some streamlining. Since a lot of things are touched upon but sometimes not discussed in detail, what is the core aim of the manuscript?
For example:
If the paper is a paper about how to best do imputation for local ancestry inference one would need to check the best reference set for GLIMPSE to do that (especially for Denisovan ancestry, the 1 k Genomes has no populations with high Denisovan ancestry), how ascertainments (i.e. captured data) influences imputation, how it works using different LA callers and what are the best filter parameters. And in the end have a set of recommended parameter settings.

If however the focus is more one power increase for calling candidates of adaptive introgression e.g. only for Neandertal variants, then why not expand the number of individuals since now you can use low coverage and potentially also all the thousands of captured samples.

Or is the aim to find Denisovan ancestry in single ancient individuals which is hard for “ancestry painting” LA callers like admixfrog or CRF since they might be biased to the reference available. So hmmix is the way to go. However it needs high quality data which is not available for most ancient genomes (especially for non-West-Eurasian genomes) so one can potentially impute genomes to refine the detection of Denisovan ancestry.

Also, since a lot of ancient individuals are only captured it would be very interesting and relevant to know how the imputation works on captured data.

Specific comments:

Line numbers would have be useful to directly refer to parts in the manuscript.

GILMPSE provides phased haplotypes, do you use this information and do you think it works, since having phased archaic haplotypes makes distance measures and TMRCA estimation easier in downstream analysis?

Using the 1k Genomes sites I assume these are sites where humans are polymorphic, that also means you never look at sites where present day modern humans have fixed differences to the archaics. Do you keep this ascertainment for all analysis? You could imagine that ancient individuals do carry fixed different sites between archaics and present day humans.

Concerning captured data, which is most of the ancient data. Some of these capture arrays are not specifically designed to capture archaic ancestry (like the widely used 1240k and twist capture), it would be very interesting if one can successfully impute the archaic ancestry there (e.g. filter the test ancient genomes to sites overlapping the capture array similar to Sousa de Mota 2023).

Fig. 1 What are the boxplots showing? All individuals and their D or alpha? A supplement figure showing the values (y axis) per individual (x-axis) for the original and imputed estimates, for each downsampled (facet) might be informative. Why has Mota a very high D in the original data (which is very significant as Fig S1 shows). Why does it vanish when using imputed OC? Maybe check the setup of the D stats, it could be that Mota has very deep relations to the other Africans which might introduce the imbalance.

One point of concern for me with imputation would be false positives, one would really not want to impute archaic ancestry segments into individuals which are not there. For example in the African individual Mota, which probably only has very few if any Neandertal segments, after imputation it has many more (Fig 2A), but is that not your negative control (i.e. where you don't 'want' to see archaic ancestry imputed)? You use the original data as ground truth and apply a cutoff per genotype of 10 reads or more, which will lose you a lot of data especially for the individuals close to 10 x (e.g. Mota). I guess this is one reason why you consistently impute more archaic ancestry in imputed OC genomes. Even after filtering it is usually twice as much and for your 'negative control' it is more than 10 times as much. To gauge if it is a false positive, you could look at the reads covering sites where you impute archaic variants at sites with missing genotypes in the original (since for a lot of them you should have reads covering them but no genotypes). How often do the reads support the imputed variant and how often not?

Why do the authors not use a recombination map to base the filtering on recombination distances? The filter is 40 kb (or 100 kb for Denisovan segments), but they could be in low recombination regions.

Fig S5. It seems after imputation your non-archaic segments consistently all shift to be closer to the archaic genome. Why would that be? If you are better in calling archaic ancestry after imputation would you not assume that the non-archaic segments would be even more distant to the archaic since you 'filtered' them from misassigned segments? Do you introduce variants which are closer to the archaic or do you correct variants in the original. Table 1 does not show signs for either (e.i." . -> 0" is big and ". -> 1" small).

Fig3 B. I would also plot the specificity of the heterozygous calls.

Denisovan ancestry: You do not find any Denisovan segment in Ust'Ishim in the original data. After imputation you do find 4 segments with one matching the hmmix map and one to the ArchaicSeeker map. At 1x you find 6 segments 2 matching hmmix map 4 matching the other. Are these additional 2 segments, compared to imputed OC? Or did the 4 OC segments break into 6 shorter ones? If the 1x really has additional non overlapping segments compared to the OC imputed that would be worrisome, since then the imputation would have introduced Denisovan segments where there are none. Do you think the segments in Ust'Ishim are true? You find evidence that Ust'Ishim predates the split between Europeans and East-Asians (in concordance with earlier papers), so if you find Denisovan segments in Ust'Ishim they would predate the split. You also find that the segments are shared with present-day populations. That would suggest that the Denisovan admixture happened before Ust'Ishim lived. I think it would be nice if the authors could interpret the results a bit more. Do you think these are genuine Denisovan introgressed segments or false positives from either the imputation or hmmix?
In short, how confident are you to reliably call Denisovan segments?

The manuscript says: "AKAP1329 is significant in the original data, but not in the imputed data. Specifically, AKAP13 is present in 7 out of 8 Neolithic Europeans but only in 1 out of 3 Mesolithic individuals, failing to meet the minimum threshold for significance in imputed datasets." So what changed in comparison to the original data? Did you 'lose' segment in the Mesolithic individuals?

You identified LMED2 and MLN as promising candidates for adaptive introgression. Are they also in high frequency in present-day Europeans? If yes, why did no selection scan in present-day Europeans find them? Maybe you can provide more information on these genes, what are they doing?

(Remarks on code availability)

Version 1:

Reviewer comments:

Reviewer #1

(Remarks to the Author)
The authors addressed my comments and concerns and I recommend the publication of this study, provided that the authors fix some broken links (see below) and provide more information on how to use their scripts (README page on github). I do still think that the article is longer than it should be, and, particularly, the discussion can be shortened; it reads a bit repetitive when taking the results' section into account.

I found some github links that did not lead to the right pages (main text): Page 22, line 38 and Page 23, line 57.

(Remarks on code availability)
I did not review the code on github, but I tried to assess how straightforward would be to run the scripts. Particularly for Local archaic ancestry inference instructions on the README file (github), I missed instructions of how to prepare the input files: what are and should contain the files *name_vcf_archaic*.vcf.gz and *name_vcf_sample*.vcf.gz? Should all chromosomes be included into a single vcf? Why does it matter to specify a name for the dataset? Do the input files have to be vcfs, meaning not bcf? What do the different steps enumerated in the first code box do?

These pieces of information are essential for anyone trying to use these scripts for their work, and the authors should provide them. My suggestion is to have a small example dataset and the commands the authors would use for that example, but I do not think that is essential, as long as more information is provided in the first place.

Reviewer #2

(Remarks to the Author)
I highly appreciate the authors integration of the combined comments from the reviewers. I think the text is now easier to follow.

(Remarks on code availability)
Scripts for managing the data, including notebooks for reproducing some figures.

Reviewer #3

(Remarks to the Author)
I appreciate that the authors have addressed the comments raised in the previous round and the substantial effort that has gone into improving the manuscript. The analyses based on the 1240k capture data are exciting and have the potential to enable to use many more genomes to be analyzed in terms of LAI calling in future work. The more stringent filtering strategy (e.g., using privately shared SNPs), particularly for Denisovan segments, appears appropriate, and the additional analyses support the robustness of the reported calls.

As a general suggestion, it may nevertheless be helpful for the authors to more explicitly highlight in the Discussion that careful filtering is important when working with imputed data and newly identified archaic segments. The additional segments identified here tend to be shorter and somewhat less similar to the archaic references than those present in the original genome; for example, without filtering, the ratio between the proportion of genome inferred as archaic from segment calls and estimates based on f4 statistics increases substantially, whereas comparable levels are recovered after filtering (as noted in the Results). Adding one or two sentences in the Discussion providing such guidance on best practices would be particularly valuable for readers who may apply similar approaches in future studies and will strengthen the manuscript as a useful reference for the community.

Overall, I am satisfied with the current state of the manuscript.

(Remarks on code availability)

Open Access This Peer Review File is licensed under a Creative Commons Attribution 4.0 International License, which permits use, sharing, adaptation, distribution and reproduction in any medium or format, as long as you give appropriate credit to the original author(s) and the source, provide a link to the Creative Commons license, and indicate if changes were made.
In cases where reviewers are anonymous, credit should be given to 'Anonymous Referee' and the source.
The images or other third party material in this Peer Review File are included in the article's Creative Commons license, unless indicated otherwise in a credit line to the material. If material is not included in the article's Creative Commons license and your intended use is not permitted by statutory regulation or exceeds the permitted use, you will need to obtain permission directly from the copyright holder.
To view a copy of this license, visit https://creativecommons.org/licenses/by/4.0/

Archaic ancestry inference in imputed ancient human genomes

Capodiferro M.R.[1+*], Planche L.[2+*], Breslin E.M.[1], Ongaro L.[1], Ávila-Arcos M.C.[3], Jay F.[2], Cassidy L.M.[1], Huerta-Sanchez E. [1,4*]

[1] Smurfit Institute of Genetics, Trinity College Dublin, D02 CX56 Dublin 2, Ireland
[2] Interdisciplinary Laboratory of Digital Sciences, Université Paris-Saclay, Orsay, 91400, France
[3] International Laboratory for Human Genome Research, Universidad Nacional Autónoma de México, Querétaro, México.
[4] Ecology and Evolutionary Biology and Center for Computational and Molecular Biology, Brown University, Providence, RI 02906, USA

[+]These authors contributed equally and their names may be listed in either order as co-first authors.
[*] Corresponding authors.

Point by Point Response

We thank the reviewers for their comments which have strengthened the manuscript. Please find point-by-point responses (in blue) to each of the reviewers' comments. We often supplement our responses with figures which have captions written in green text.

Reviewer #1 (Remarks to the Author)

This study focuses on using genotype imputation to identify archaic introgressed regions in ancient genomes. The authors thoroughly benchmark their approach by first downsampling high-coverage ancient genomes and comparing the results of their analyses with the original ground truth calls. Using allele-frequency- and haplotype-based methods, the authors demonstrate that they can recover most of the archaic regions in ancient individuals' genomes. Furthermore, they detected even more archaic segments that they found in their ground truth dataset, which is impacted by some amount of missingness, postmortem damage and sequencing errors. A shortcoming of this approach is that the true lengths of the segments may not be recovered, particularly for the older ancient genomes, which hinders estimates of introgression time. To assess whether these imputed segments are shared with present-day populations, the authors compare them against present-day reference datasets. Depending on the ancient individuals' age and ancestry, the segments show varying levels of sharing with different modern populations.

They also found that Neanderthal-specific variants can be accurately imputed, and that they are imputed more accurately than non-archaic variants. As a consequence, the genotype posteriors (GP, a measure of how confidently imputed a variant is and is well calibrated with error rate) for these variants tend to be greater than for non-archaic variants. As a result, when applying strict quality control filters as GP>99%, they observed a bias in D-statistics. This is an important observation, because, depending on how well different ancestries can be imputed, strict GP filtering can introduce bias.They then adapted D-statistics calculations to take imputed genotype dosages as input instead of hard calls, which is a promising approach for many other methods.

Finally, the authors apply their method to gain biological insights. Through enrichment analyses, they identify genes enriched for archaic haplotypes. This analysis replicates known introgression signals and also uncovers three new candidate genes.

In my view, this work is technically sound. It makes use of state-of-the-art methods in the field and is clearly described and logically structured (even though it needs some rewriting to be more concise, particularly in the discussion section). It is a valuable contribution to the ancient DNA community. To the best of my knowledge, the question of how well archaic introgressed regions can be recovered from imputed ancient genomes has not been systematically assessed before and it is of great interest in the field. I have some noncritical comments and suggestions, which I have listed below.

Major comments:

1) My first general comment is that I missed some direct comparisons with present-day data. For instance, for Figure 2D, I do not know what to expect regarding Denisovan segments in present-day Europeans.

Answer:
In present-day data, modern day Eurasians possess a small amount of DNA (1.5–2.1%) from Neanderthals, while modern East Asians possess around 0.1-0.2% from Denisovans and modern Europeans exhibit close to none (Racimo et al. 2015).

To confirm these results, we analysed the introgression maps obtained using *hmmix* in the original Skov et al. paper (Skov et al. 2018). The results are reported in the Supplementary Materials (Figure S12-S13, Tables S16), in the section "Summary of introgression in present-day individuals based on Skov et al. maps".

In our Imputed OC individuals, we find between 1.1% (JP14) and 1.9% (Ust'Ishim) of the DNA with an archaic origin, and between 1% and 1.5% for the Imputed 0.5X which is within the expectations for present-day individuals.

Regarding Denisovan introgression, we find that Kolyma1 exhibits the largest amount of Denisovan ancestry (0.04%), but this is likely an underestimate as we applied very stringent filters to classify a segment as Denisovan (see Methods).

We added to the main text, in "Quality and Properties of Inferred Archaic Segments":

"Overall, excluding Mota, we find between 1.1% (JP14, Ireland 5483 BCE) and 1.9% (Ust'Ishim, Russia 44366 BCE) of archaic ancestry in imputed original coverage (Imputed OC) individuals. For present-day Eurasians, this amount is estimated around 1.5-2.1%[1,2]."

Also, with a high-quality high-coverage genome (downsampled to intermediate coverages to match the ancient data), it would be easier to pinpoint to what extent the detection of Archaic New segments is the result of missingness in the original coverage genome (and how many of these are potentially false positives). That said, I understand that suggesting new analyses is easier than performing them. For the first point in particular, I would be satisfied with an explicit, well-referenced sentence in the text to clarify expectations.

Answer:
Table 1 was our attempt to justify that the reason we find new archaic segments (Archaic New) in the imputed genomes that are absent in the original coverage genome was due to missing data in the original coverage genomes. To make the connection between missingness in original coverage genomes and the Archaic New segments, we created Table S3 which contains the number of missing positions in the original coverage individuals (using their VCF files) under different categories (Non Archaic, Archaic Shared and Archaic New) in Table 1. Table S3 shows that the "Archaic New" category exhibits a higher proportion (~63–70%) of missing data than "Archaic shared" (~50–52%) or "Non Archaic" (~55%).

To make this claim stronger, we went back to the original BAM files and verified that all Archaic New segments had read support for the imputed genotypes at sites that are missing (i.e. no genotypes were called, or genotypes were filtered out by quality filtering) in the original VCF file. As we expected, we find that in positions where we imputed a homozygous alternative genotype, the proportion of reads with the alternative allele is almost 100%, and ~50% (on average) at imputed heterozygous genotypes (Figure S21). Only a small fraction of sites (2-10%) show deviations from expected proportions where the proportion of reads with the alternative-allele is < 10%. When we look at the absolute number of reads, we find that these sites have the lowest read-depth (between 1 and 10 reads, Figure S22) which explains why genotype calls at these sites were filtered out of the original VCF file. Together, these results show that imputation is working properly in the Archaic New segments, therefore, the archaic alleles present in the "New Archaic" segments are correctly imputed as those alleles have read support in the BAM files. We thank the reviewer for noting that this was previously

unclear; we have expanded the discussion in the Supplementary Material in a new paragraph named "*BAM-derived support for imputed variants in Archaic New segments*".

2) Upon generating the high-confidence dataset, did you apply any methods to reduce postmortem damage (PMD), such as read trimming? Generating a high-quality ground truth for ancient genomes is always challenging, as PMD can introduce artificial differences between the imputed calls and the ground truth.

Answer:
We clipped the reads to reduce postmortem damage which is effectively the same as trimming. We provide a detailed description of the filtering performed in the Materials and Methods section:
"First, reads were sorted, and PCR duplicates were removed. Realignment around indels and addition of read group information were performed. To mitigate the impact of postmortem damage, two base pairs were clipped from both ends of each read. Only reads with a minimum mapping quality of 37 and a length of at least 30 bp were retained."

3) On page 7, it says "Moreover, considering only the filtered results (the bar with the black border in Figure 2A), the older individuals (shown on the left) have a higher proportion of their genome inferred to be of archaic origin across all coverage levels, consistent with expectations." There are some fluctuations for total introgressed length (for example, BOT2016 seems to have more more introgressed segments (in length). Why do you think this is the case? One potential issue may come from measuring length in base pairs instead of centimorgans.

Answer:
Fluctuations in inferred archaic ancestry are expected in present-day individuals, as reported in prior studies (Witt et al. 2023), so the variation we see is consistent with normal stochastic fluctuations, and continental differences. We note that BOT2016 has been shown to harbor considerable East Eurasian ancestry (~45%) (de Barros Damgaard et al. 2018) which will also increase the amount of archaic ancestry in this individual. We do agree with the reviewer that the sentence mentioned can create some confusion. Note that only Ust'Ishim and Yana1 are at significantly different time periods than the others, which is why they are the only ones expected to exhibit strong signals related to their age, such as admixture proportion, but also segment length as seen in Figure S9. We have now added this explanation to the Supplementary Information. For this reason, we rewrote the sentence cited by the reviewer (in "Quality and Properties of Inferred Archaic Segments") to make it clearer:
"Moreover, considering only the filtered results (the bar with the black border in Figure 2A), the two older individuals, Ust'Ishim (Russia, 44366 BCE) and Yana1 (Siberia, 31850 BCE), have a higher proportion of their genome inferred to be of archaic origin across all coverage levels. Among the more recent individuals, depending on the geographic location and ancestral composition, we see fluctuation on the archaic proportion. Kolyma1 (Siberia, 9775 BCE), USR1 (Alaska, 11425 BCE), as well as BOT2016 (Kazakhstan, 5396 BCE), all carrying Ancient East Asian (AEA) ancestry, show a high archaic proportion, in line with continental expectations, as contemporary East Asians harbor more archaic ancestry than contemporary Europeans Overall, excluding Mota, we find between 1.1% (JP14, Ireland 5483 BCE) and 1.9% (Ust'Ishim, Russia 44366 BCE) of archaic ancestry in imputed original coverage (Imputed OC) individuals. For modern day Eurasians, this amount is estimated around 1.5-2.1%."

Please convert these lengths to cM to better compare introgressed segment lengths across the different samples. Furthermore, please provide a similar figure to S8, where the total introgressed length is plotted against sample age.

Answer:
For each segment, centiMorgans (cM) are now reported in the introgression maps.
After converting segment lengths to centiMorgans (cM), we observe the same pattern (Figure R1.3.1).

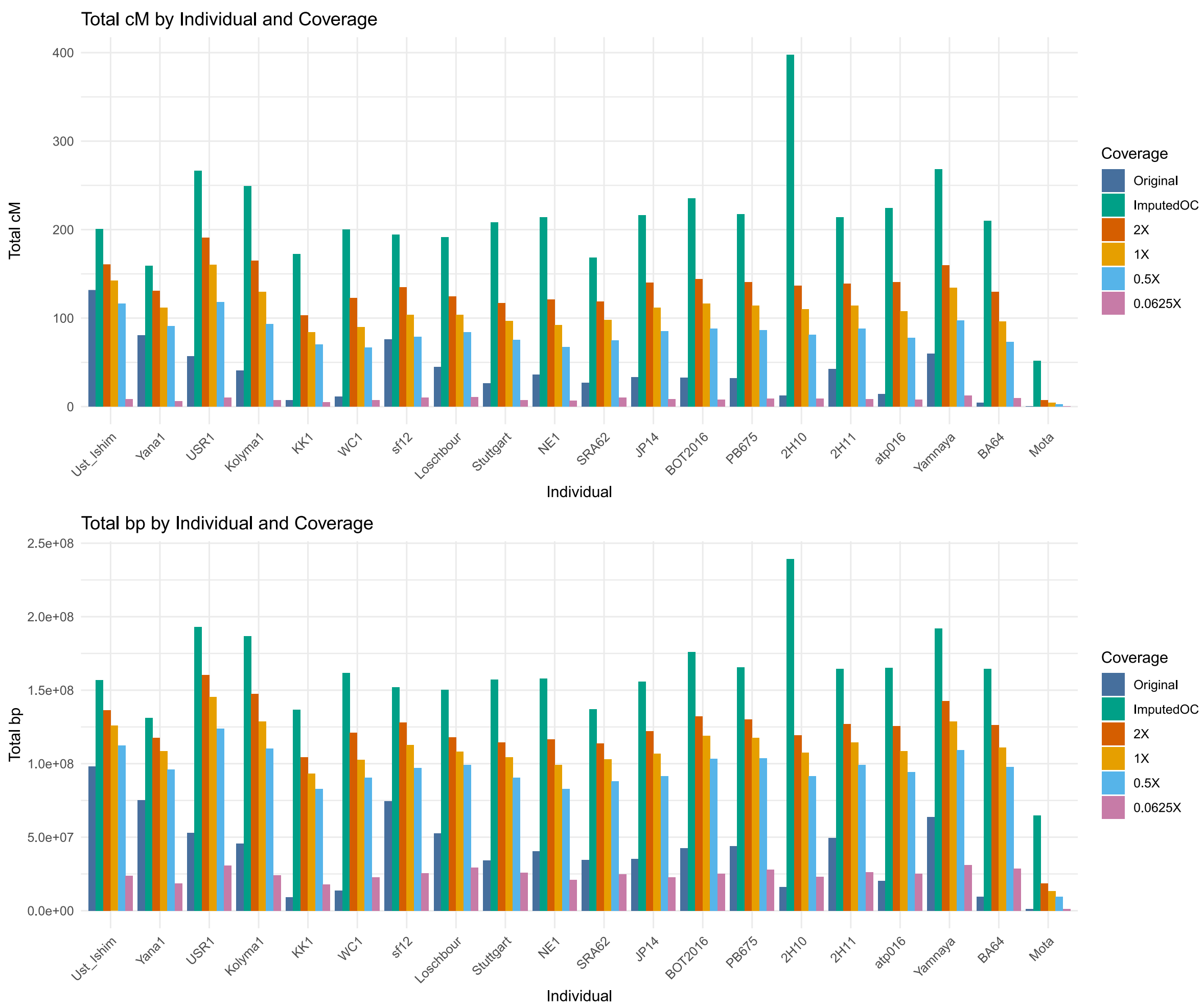

Figure R1.3.1. *Comparison between cM (top) and bp (bottom) for the total amount of archaic introgression in each individual and coverage, as in Figure 2A. Note that these values are shown before applying quality filters.*

4) In Figure 2B, after filtering, why do we observe a higher proportion of recovered introgressed segments in the imputed 1x or 2x genomes compared to the imputed OC genomes for most individual samples?

Answer:
We attribute the effect observed in Figure 2B, to segment length in the Imputed OC dataset which leads to a slightly lower similarity on average. For example, if we condition on the “Archaic Shared” segments that were removed in the ImputedOC and inspect their length and similarity in the other coverages, we find that these segments are on average slightly longer in the ImputedOC (Figure R1.4.1 below). If we randomly choose one of these segments and

visualize their lengths, we can see that the segments are shorter in the downsampled imputed (Figure R1.4.2 below). As shown in Figure S15A, the average segment length in Imputed OC is higher than in the imputed downsampled coverages, which are otherwise very similar to one another. This pattern is also illustrated in one of the selected genes, which was raised in a comment by Reviewer 3 for the APK13 gene.

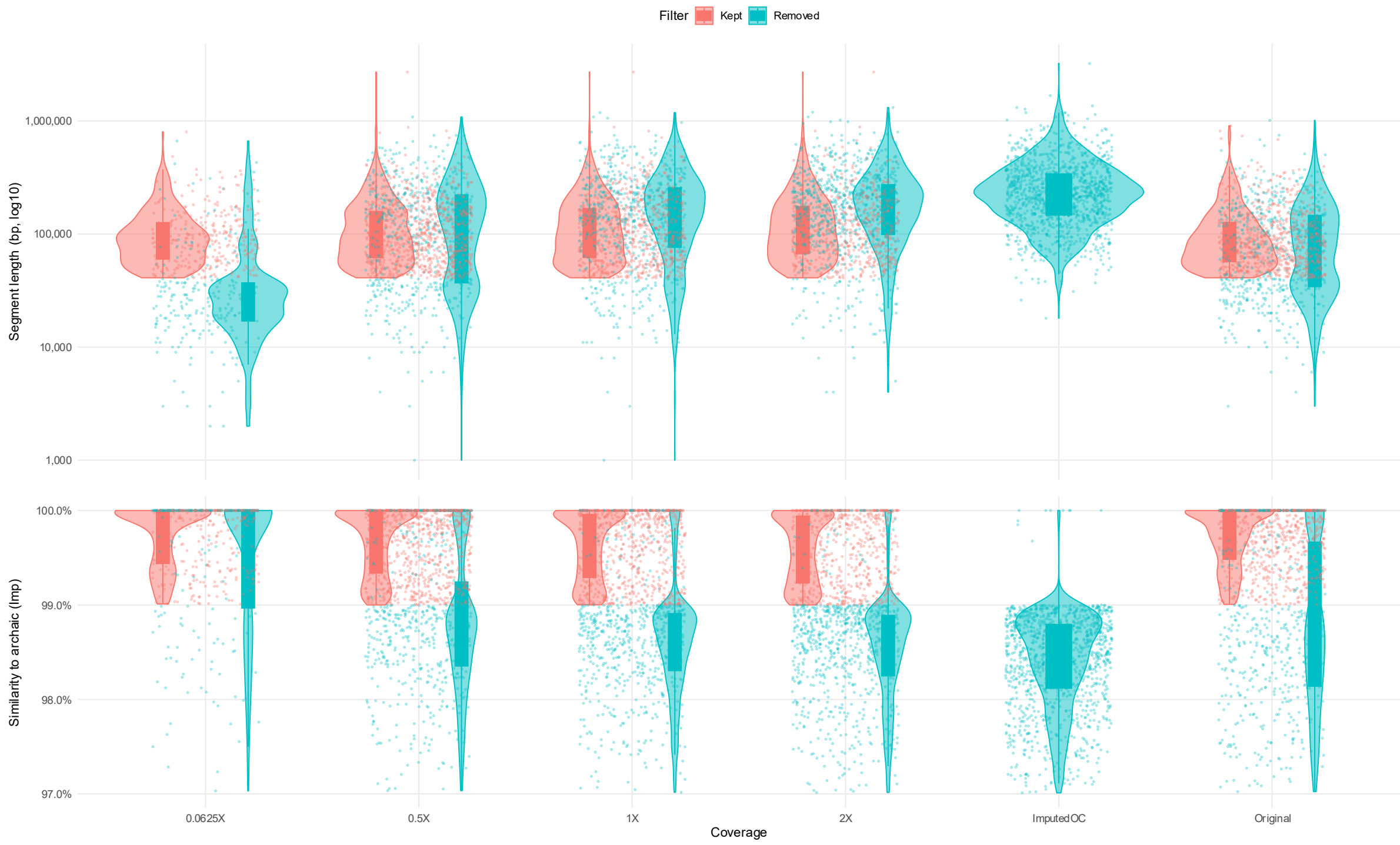

Figure R1.4.1. *Segment length (top, in log scale) and similarity to the closest archaic reference genome (bottom). Segments used here are the ones that are filtered out in the ImputedOC that were present in the set of "Archaic Shared" between original genomes and ImputedOC before filtering. We then ask if these segments overlap with segments present in the corresponding downsampled imputed individual. Columns 1-5 show results for each coverage of the downsample individual, and in the last column we consider the original coverage individual. The colors represent if the segment passes (red) or not (green) the quality filters.*

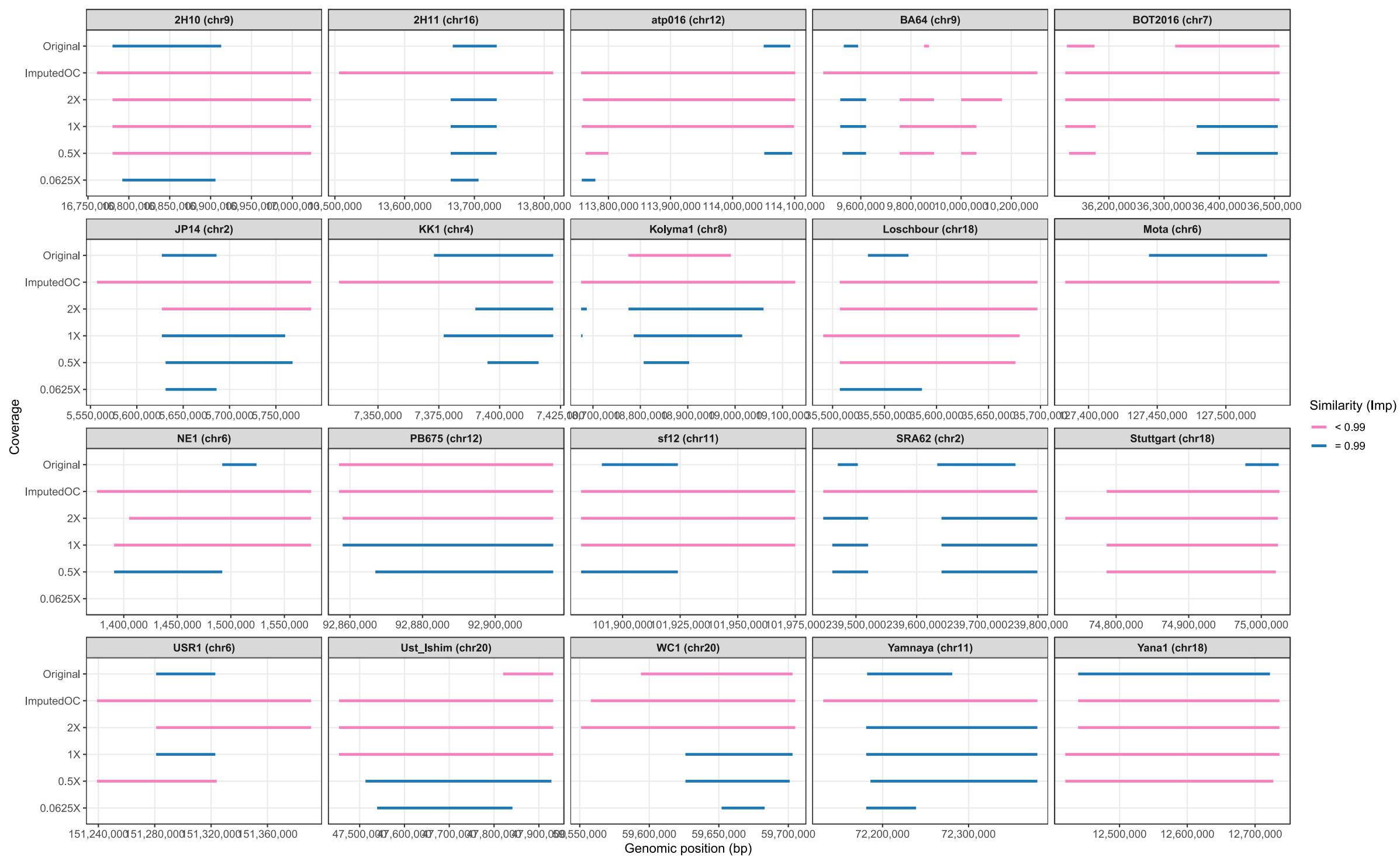

Figure R1.4.2: *Randomly selected genomic regions per Individual that contain an archaic segment that is shared with the Original individual and is filtered out in Imputed OC but kept in some other coverages. In pink segments that are removed after filtering, in blue segments that are kept. Segments in ImputedOC often span larger regions than in other coverages which affects their similarity to the closest archaic genome.*

4) bis. Furthermore, in Figure S22, there are more significant hits in the imputed 2x dataset (10 genes in red and 2 in blue) than in the imputed OC dataset (10 in blue and 3 in red). Is this difference in the enrichment analysis driven by a difference in the number of detected introgressed segments? I have the intuition that PMD may have a greater impact when imputing higher coverage genomes, because genotype likelihoods can appear more confident when more reads cover a site, even if a substantial fraction of those reads carry deaminated bases at that position.

Answer:
The differences in significance across coverages reflect both the number of tested segments and the degree of segment sharing across individuals. As the number of candidate segments increases, the multiple-testing burden grows, and the Bonferroni threshold becomes more stringent. Consequently, more individuals must share a segment at a locus for it to reach significance. Because the Imputed OC has more segments, the number of individuals that must share the segment is higher.

5) The authors show that imputation allows us to detect new archaic segments that we could not detect in the original high-coverage genome. Moreover, their filtering approach seems to be efficient at removing potential false positives, as shown in Figure S6. However, I would like to see further sanity checks. First, I suggest splitting the archaic segments into the two categories Archaic Shared and Archaic New. Then, we can apply the same approach as in Figures 4A and 4B to verify if these two types of introgressed segments share the same pattern

with present-day populations (Figure 4A) and if they both preserve the expected population structure of the individual samples (Figure 4B).

Answer:
We have to clarify that in a real case scenario we do not have Archaic-Shared and Archaic New. These categories depend on the amount of archaic introgression detectable in the Original VCF, which, as shown in Figure S6 (first panel), is positively correlated with the original coverage. Consequently, analyses restricted to these categories can be biased because they preferentially include segments present in the Original (i.e., not dependent on imputation).

We conducted the reviewer's suggested analyses, by comparing the Venn diagrams for Archaic New (Figure R1.5.1) and Archaic Shared (Figure R1.5.2) segments. In both cases the results are qualitatively comparable, although using only Archaic New segments, we observe differences particularly among European Neolithic individuals: some share fewer segments with present-day West Eurasians than in Figure 4.

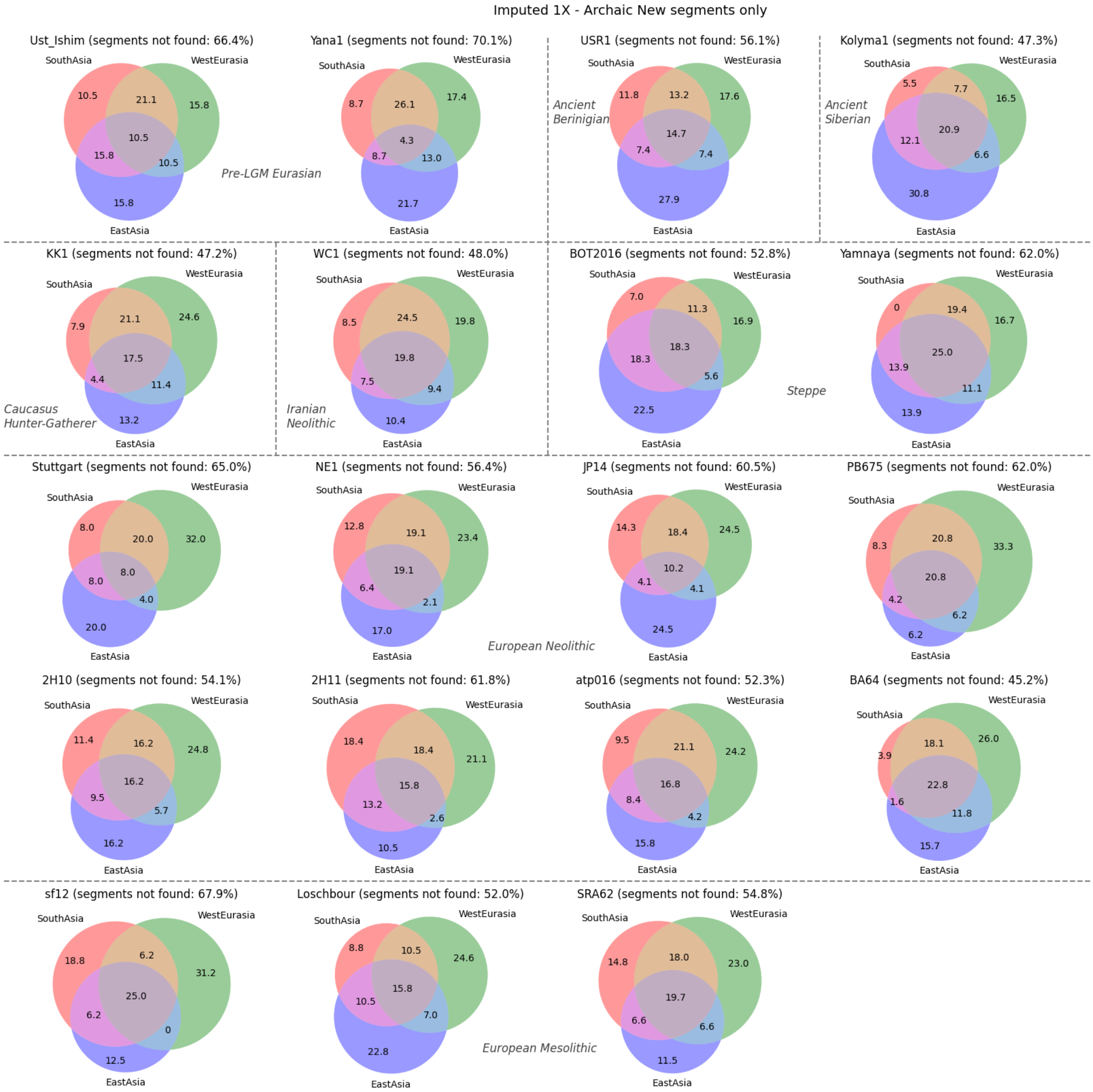

Figure R1.5.1. *Venn diagram describing the proportion of shared archaic segments between imputed 1X ancient genomes and introgression maps from present-day individuals (Skov et al. 2018) as in Figure 4A in the main text. Here we restricted the analyses on the Archaic New segments.*

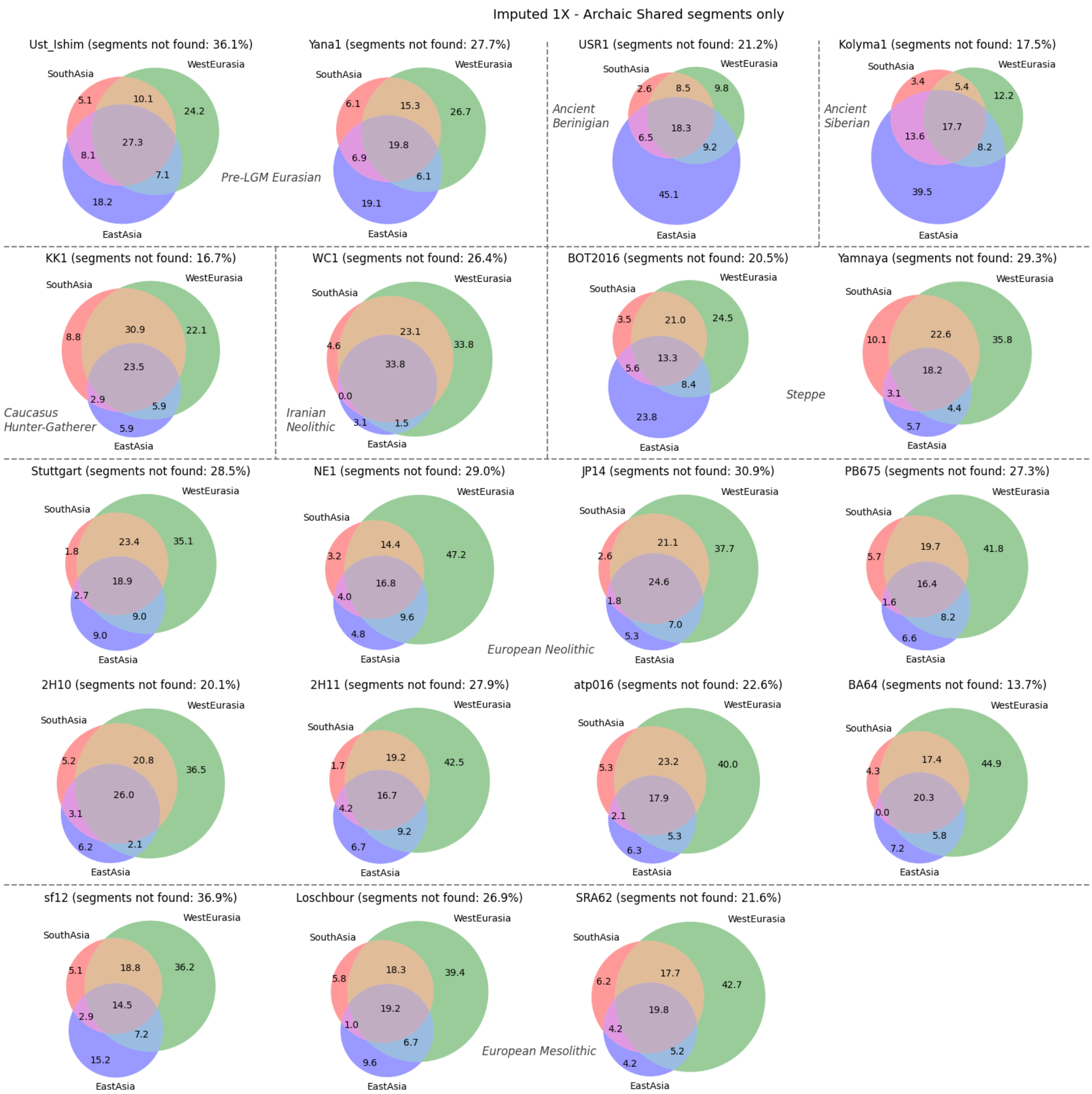

Figure R1.5.2. Venn diagram describing the proportion of shared archaic segments between imputed 1X ancient genomes and introgression maps from present-day individuals (Skov et al. 2018) as in Figure 4A in the main text. Here we restricted the analyses on the Archaic Shared segments.

Finally, MDS analyses based solely on Archaic Shared (Figure R1.5.3) or Archaic New (Figure R1.5.4) segments show that population structure is preserved even when each segment category is analyzed in isolation. Using only Archaic Shared segments and excluding the outlier Mota, we recover the same overall structure seen with all segments. Using only Archaic-New segments, the broad pattern also persists, but individuals are more dispersed (i.e., less tightly clustered). With the exception of the very low-coverage set (0.0625×), population structure is still maintained in the Archaic New subset, suggesting no major imputation-induced bias in these tracts. These figures are now reported in the Supplementary Materials as Figures S35 and S36.

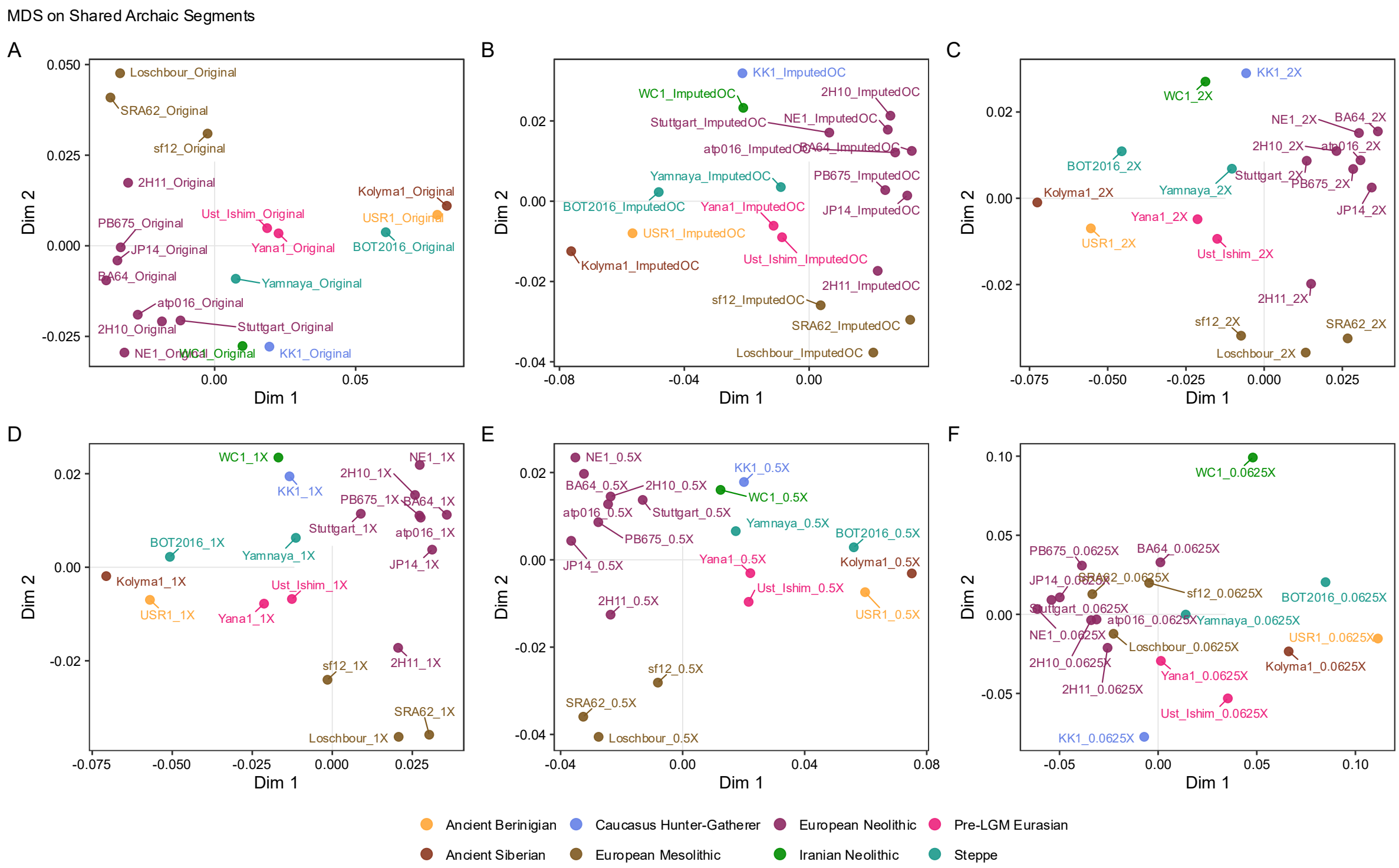

Figure R1.5.3. *Multidimensional scaling (MDS) plots of shared "Archaic Shared" introgressed segments across coverage levels (A. Original, B Imputed OC, C 2X, D 1X, E 0.5X and F 0.0625X), based on segment count overlap. In this analysis the individual Mota has been excluded.*

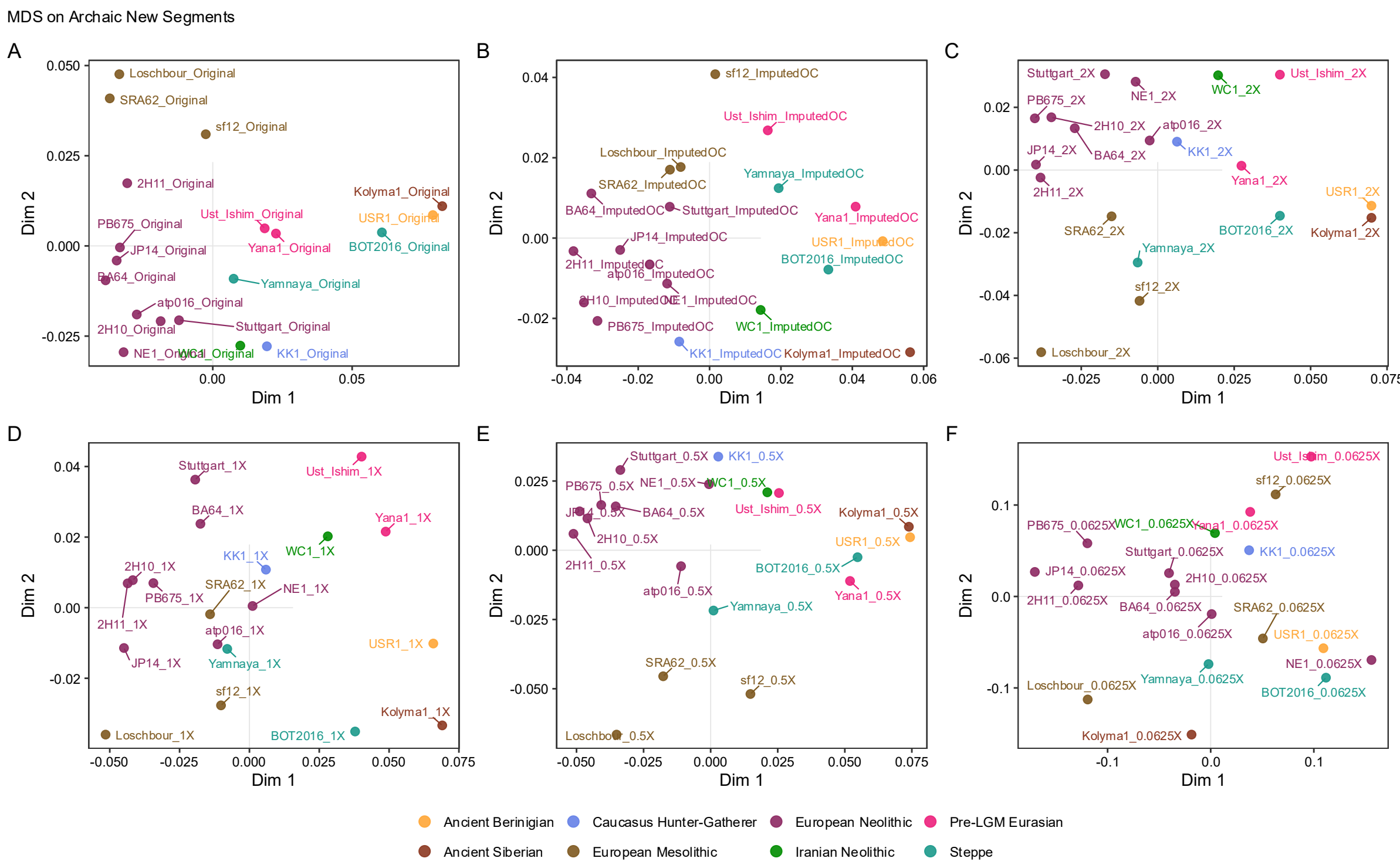

Figure R1.5.4. *Multidimensional scaling (MDS) plots of shared "Archaic New" introgressed segments across coverage levels (A. Original, B Imputed OC, C 2X, D 1X, E 0.5X and F 0.0625X), based on segment count overlap. In this analysis the individual Mota has been excluded.*

5) bis. Furthermore, regarding the segments not found in present-day populations, are they enriched in one of the two categories?

Answer:
The proportion of segments not found in present-day populations is listed at the top of each Venn diagram for each category of *Archaic Shared* and *Archaic New*. The proportion varies by individual because the Shared and New categories depend on the original coverage of each ancient individual. On average, there is a higher proportion of *Archaic New* not shared with present-day populations. However, we have inspected these *Archaic New* segments in the original genome and we know that 1) imputation works in these segments by going back into the original bam files (see Figure S21-S24), 2) we are using a minimum length of 40Kb, 3) these segments harbor archaic alleles (see introgression maps) and 4) the genetic similarity between the archaic human and the original genome at these segments is higher than for the rest of the genome (Figure 2C). Therefore, we are confident that the vast majority of these segments are real archaic segments.

6) To evaluate imputation performance in introgressed regions, the authors restricted the analysis to alleles absent in African populations and homozygous in the Vindija Neanderthal genome. While this is a reasonable approach to assess imputation performance for Neanderthal-introgressed regions, it does not inform us about how well Denisovan-specific SNPs or regions are imputed. Could you apply the same approach to evaluate this?

In that sense, the statement that archaic segments are more accurately imputed should be made more precise. In other words, when we read sentences as “One surprising result is that imputation performs better in archaic versus non-archaic regions and variants [...]” (Discussion), we should in fact read “imputation performs better in Neanderthal-introgressed regions”. I suspect Denisovan-specific variants may be more challenging to impute due to: (1) distinct archaic population histories, and (2) the smaller amount of Denisovan DNA present in the imputation reference panel.

Answer:
Defining Denisovan SNPs as absent in African populations and homozygous in the Denisovan, we plot the imputation accuracy for Denisovan SNPs (Figure R1.6.1 below and now reported in Figure S18). Even in this case, the accuracy of the Denisovan SNPs is higher compared to the non-archaic SNPS. We also measured the accuracy of the SNPs that are private to Denisova, meaning not shared with Neanderthal as well. Even here we observe a higher accuracy (Figure R1.6.2 below and now reported in Figure S18), but considering the low amount of SNPs, in the 0.0625X downsample individual, we can observe a higher variance than compared to the previous case.

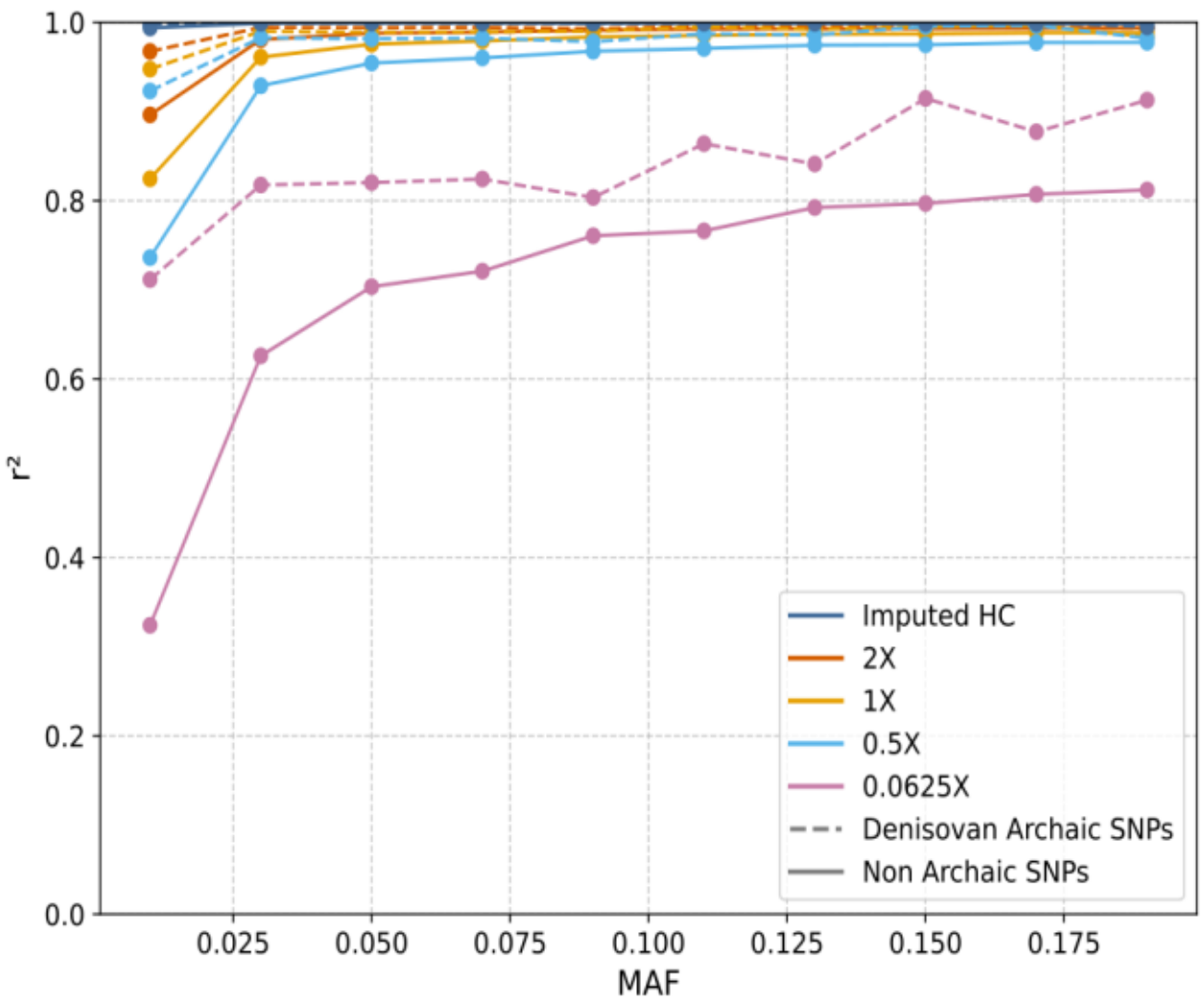

Figure R1.6.1. *We compute the accuracy of imputation for Denisovan Archaic SNPs. We consider a SNP to be Denisovan if one of its alleles is absent in Africa and homozygous in Denisova. Note that such an allele can be present in Neanderthals as the split between Neanderthal and Denisovan is more recent than the split of these two populations with Sapiens.*

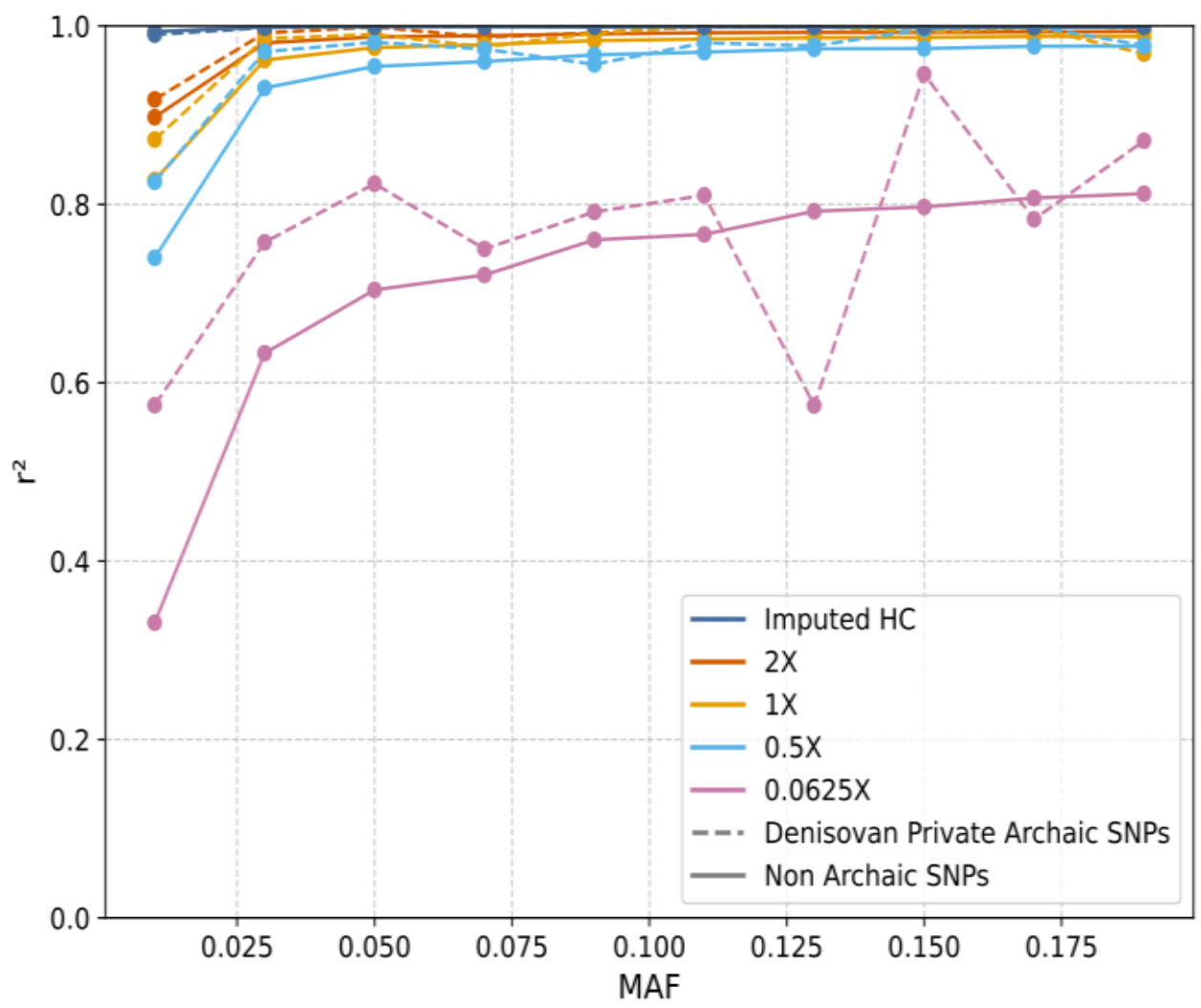

Figure R1.6.2. *We compute the accuracy of imputation for Denisovan Private Archaic SNPs. We consider SNPs to be Denisovan Private if the allele is absent in Africa, absent in Neanderthal and homozygous in Denisova.*

7) In Figure 3A, how many SNPs does each bin have? In other words, are there wildly different numbers of SNPs in the low-frequency bins that would artificially drive differences in imputation accuracy?

Answer:
We checked the number of Archaic and non-archaic SNPs for each bin category, and we find that the numbers are not wildly different for the low frequency bins (see Table R1.7.1 and Table S20).

Table R1.7.1. *Number of SNPs in the 20 Original Coverage ancient genomes for each MAF bin in the 1000 Genomes Project. Here we partitioned the SNPs into two categories (e.g. Non-Archaic and Archaic) used for Figure 3A.*

Bins	[0.0,0.02]	[0.02,0.04]	[0.04,0.06]	[0.06,0.08]	[0.08,0.1]
Non Archaic SNPs	30396	16741	17945	19757	24004
Archaic SNPs	24882	23004	16134	10340	7060

[0.1,0.12]	[0.12,0.14]	[0.14,0.16]	[0.16,0.18]	[0.18,0.2]
26698	32179	32179	35198	35434
5537	3294	1564	914	988

While it is good that the authors tried to make the archaic and non-archaic variants comparable by sampling non-archaic segments with the same length distribution, it might be even more important to ensure that these two sets of variants have similar allele frequency distribution, being one of the most impactful factors for imputation accuracy. What is the allele frequency distribution in 1000G of the archaic-specific SNPs?

Answer:
We agree with the reviewer that allele frequency is important for imputation. Linkage disequilibrium is also important since LD between variants will help the imputation process. To address the reviewer's comment below we provide 1) the frequency distribution of archaic and non-archaic SNPs present in the 1000 Genomes Project and from simulations using msprime (Figure R1.7.1) under a simple demographic model, 2) the table of the number of SNPs (in the 1000 Genomes Project) for the set of archaic and non-archaic SNPs present in the *20 Original High Coverage* individuals for the different MAF bins, and 3) the LD between archaic and non-archaic SNPs.

Observations:
Compared to archaic alleles, there is (Figure R1.7.1) an excess of non-archaic alleles in the allele frequency range of 0-2% and above > 12%. Considering that the most difficult alleles to impute are in the 0-2% MAF range (Figure 3.A), this observation further consolidates the conclusions from Figure 3 that non-archaic alleles are harder to impute than archaic ones.
Finally, we also observe much stronger LD between archaic alleles than between non-archaic alleles, and this is a probable explanation for why archaic positions are easier to impute (Figure R1.7.2).

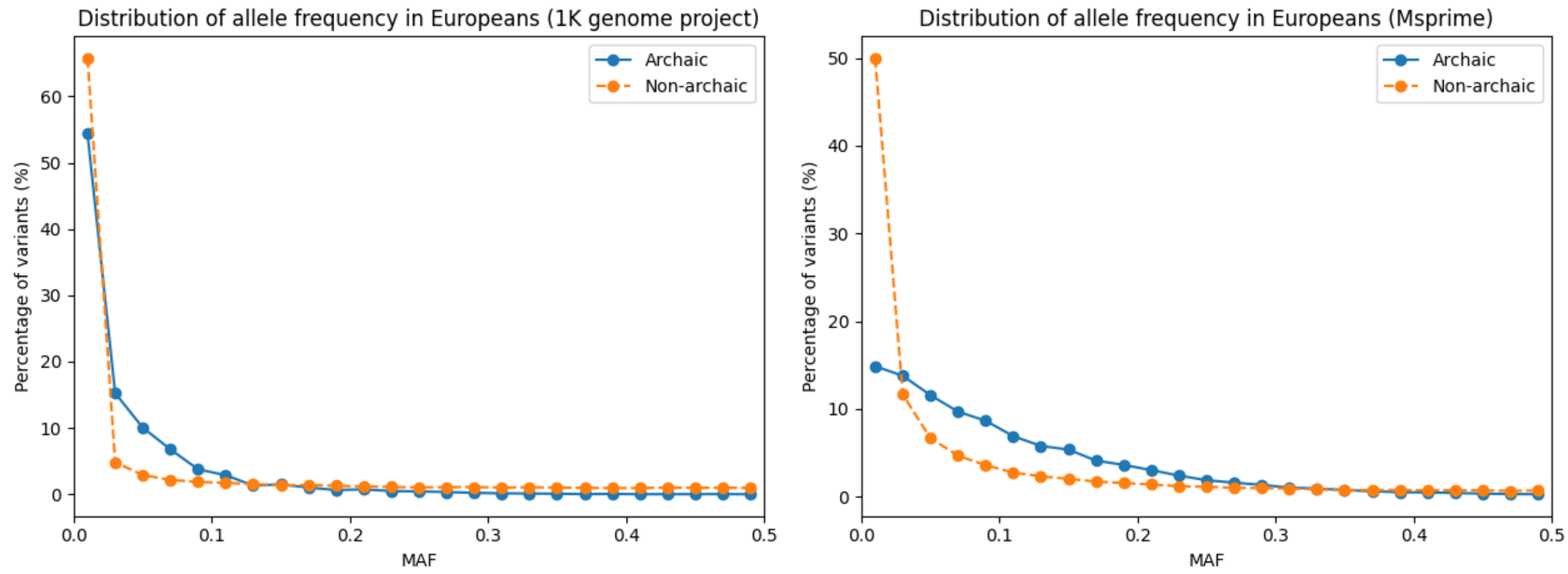

Figure R1.7.1. *Allelic frequency in Europeans, for chromosome 1 of 1000 genome project on the left, and msprime simulations on the right, for archaic and non archaic positions. Archaic positions in the 1000 genome project are defined as positions containing an allele present in Vindija Neanderthal, absent in Africans (Yoruba, Mende and Esan) and present in the 1000 genome project. The simulations assume Out of Africa 2000 generations ago, Neanderthal admixture (4%) 1850 generations ago, population sizes of 27000, 10000 and 30000 for respectively Africans, Europeans, Neanderthal.*

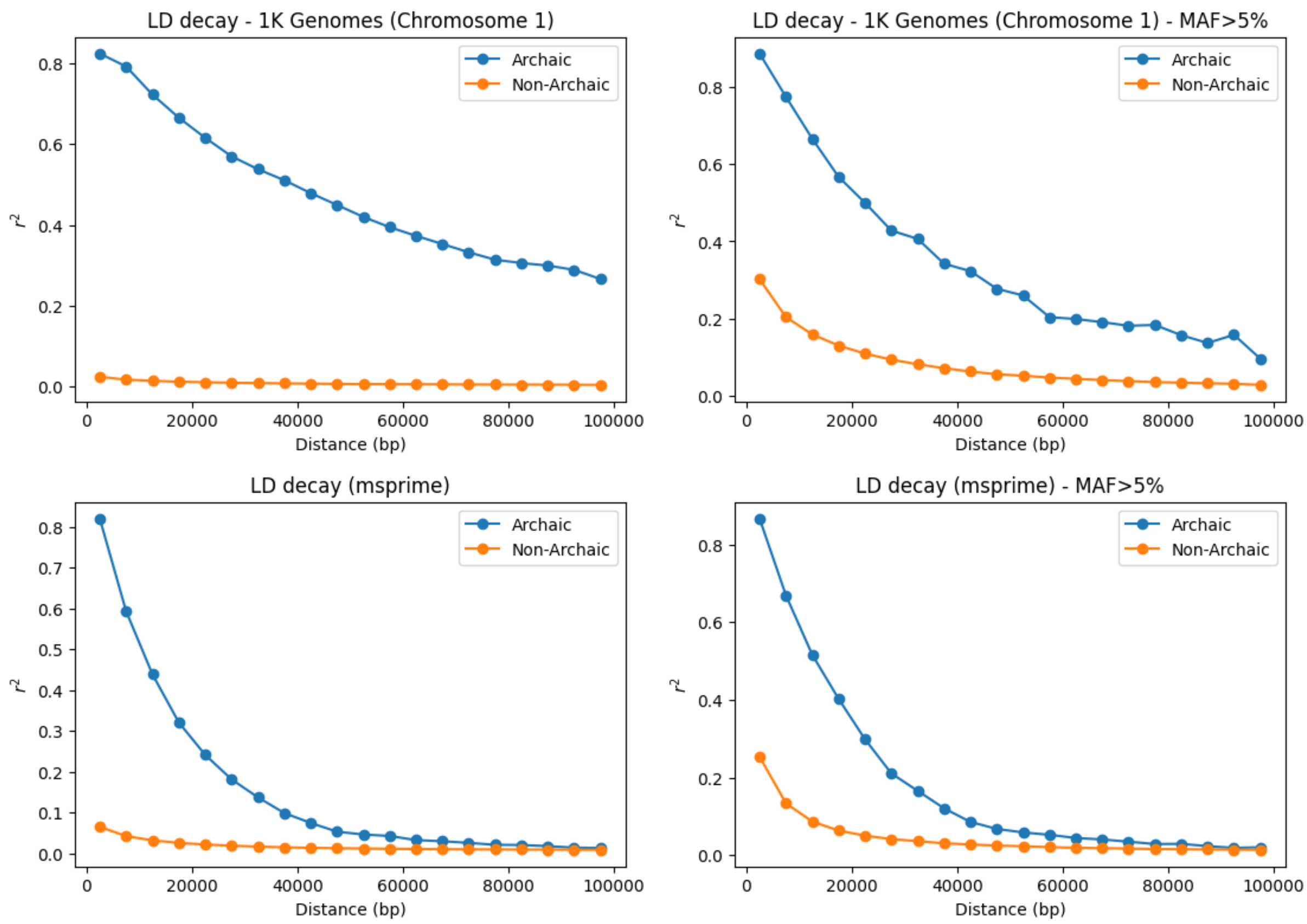

Figure R1.7.2. *LD in Eurasians (British, Iberian, Finnish, Toscani, Utah residents with Northern and Western European ancestry, Japanese, Han Chinese) from the 1000 genome project, chromosome 1 and LD from msprime simulations for archaic and non archaic positions. Left taking all the positions, right with positions with MAF > 5%. Archaic positions in the 1000 Genomes Project are defined as positions containing an allele present in Vindija*

Neanderthal, absent in Africans (Yoruba, Mende and Esan) and present in the 1000 Genome Project. For msprime, archaic positions are positions where the mutation happened in the Neanderthal population, the information being directly taken from the tree sequence. The simulations assume Out of Africa 2000 generations ago, Neanderthal admixture (4%) 1850 generations ago, population sizes of 27000, 10000 and 3000 for respectively Africans, Europeans, Neanderthal. A sequence of size 250,000,000 is simulated for 150 Europeans.

We added the first figure in supplementary materials with following sentences to section "Quality of Imputation in archaic vs not archaic genomic regions":
"One reason for the improved accuracy observed for archaic variants is that archaic SNPs tend to be in higher LD, particularly for older individuals (Sankararaman et al. 2012). We also note that due to their deeper evolutionary origin (Kimura 1955), the archaic alleles show a lower proportion of variants at frequencies below 2% compared to non-archaic alleles, further improving imputation quality (Figure S17)."

Additionally, please provide the raw data used to generate this plot (and also Figure 3B) as well as the number of SNPs per curve per bin and their average MAF in a supplementary table.

The raw data used to generate the Figure 3A (Table R1.7.1) is now presented in Table S13 and Table S20.

8) I would like to see a supplementary figure showing the imputation error rate (or preferably heterozygosity sensitivity or non-reference discordance) per individual across different coverage levels. The reason for this request is that Figure 3 presents aggregate imputation accuracy across individuals, which may mask variation due to ancestry. I am interested in whether the specific ancestry context plays a major role. For example, if a Neanderthal-introgressed segment is found in a well-represented genetic background (e.g., European), would it be more accurately imputed? Conversely, if I attempt to extract Neanderthal-introgressed segments from an ancient Papuan genome, should I expect similar performance, or would imputation accuracy drop due to limited representation in the reference panel?

Answer:
The per-individual, per-coverage heterozygosity sensitivity plot is now included as Figure S19. In addition, the data used to generate the plot are provided in Table S14-S15. These analyses do not show any kind of differential accuracy, sensitivity or imputation quality based on the ancestry (with the exception of Mota) compared to what we would expect from the normal imputation. We now also provide the average genotype probability per individual for the ABBA and BABA sites (Figure S20, Table S11). In our dataset we have Ust'Ishim as a perfect representative of a lineage that is not well represented in present-day individuals, belonging to a parallel branch to the one that has given rise to the west and east upper-paloeolithic branches of modern humans. Even in this case, imputation accuracy is working very well in introgressed regions. Therefore, we expect that this approach would likely perform well in regions containing introgressed segments in Papuans. This prediction could be rigorously evaluated using present-day Papuan genomes, although such an analysis lies beyond the scope of the current study.

9) On page 8 of the Supplementary Information file, the authors described how they classified introgressed segments in ancient individuals as shared with present-day individuals (Figure

4A). “We consider that two segments are matching if they overlap for 80% of their length.” I would like to understand the rationale behind choosing this threshold. How sensitive are the results to this 80% overlap threshold? I am concerned that this approach excludes segments with a smaller but still relevant amount of overlap. This could affect the number of segments attributed to a particular population and the segments found to be shared with none of the present-day individuals. Have you considered classifying shared segments based on the amount of overlap between them?

Answer:
The reviewer makes an interesting remark, the reason why we decided for such an overlap is that we ideally wanted to detect segments which have the same introgressed ancestry and not two regions with archaic ancestry which by chance overlap. Here we are comparing a single ancient individual to a large sample of individuals in the 1000 Genomes Project. As around a third of the genome is covered with archaic ancestry in West Eurasian, East Asians and South Asians, these “random” overlaps are bound to happen. By constraining the segments to overlap on a significant portion of their length, we tried to avoid this effect.
In any case, it is true that what the reviewer proposes, looking at the shared segment based on the amount of overlap, is a less arbitrary measure and we have now done it following their advice; the result for Imputed 1X is given below. To do so, we simply went through all introgressed segments in the ancient individual and counted - in length - which parts of the segments were shared with the archaic segments present within each region in the 1000 Genomes Project. What can be observed is that - as expected - the amount that is shared between regions increases, but the overall pattern stays the same (Figure R1.10.1).
In general, we feel more work is needed to evaluate what is the best way to measure sharing. We believe that not requiring a strict overlap will create a lot of shared signals that are not necessarily significant. However, these signals can be double checked by computing a pairwise similarity between the two archaic segments found within the same region in two different individuals. We haven’t done this last step, but it would be very informative for an indepth study of haplotype trajectory.

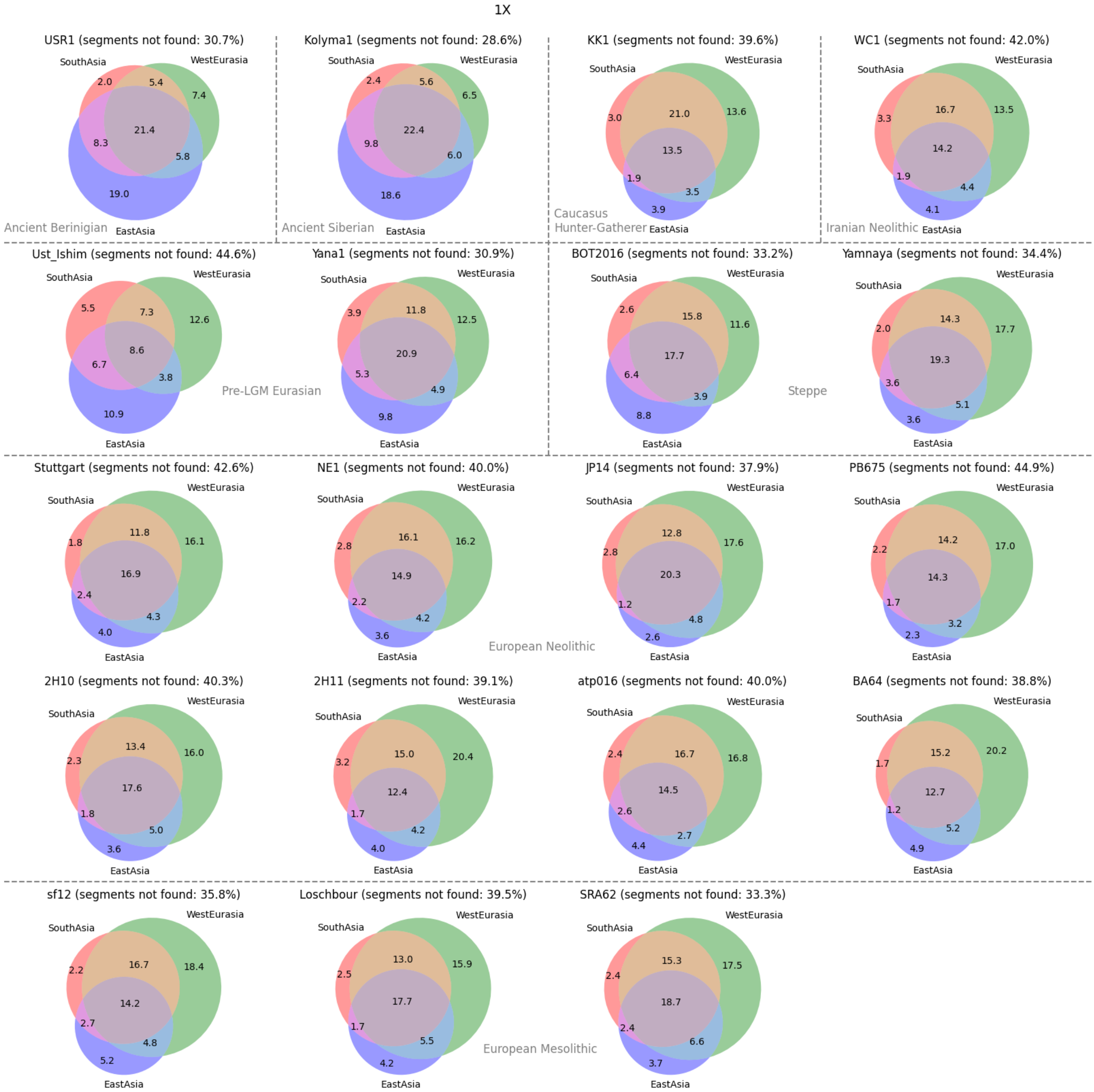

Figure R1.10.1. *Venn diagram describing the proportion of shared archaic segments between imputed 1X ancient genomes and introgression maps from present-day individuals (Skov et al.). Differently from Figure 4A we here are not using an overlap threshold to label segments as shared. Instead, we directly compute the length of the overlap whatever the segment sizes. The main pattern is not changing.*

10) I suggest making Figure S22 part of the main article, as it illustrates how this approach can be applied and presents new results. For the new candidate introgressed genes, why have these not been found in present-day populations? Are these regions enriched in segments not found in present-day people (Figure 4A)? Additionally, could you assess the frequency at which these introgressed genes occur in present-day European populations?

Answer:
The Manhattan plot is now reported as Figure 5. We measured the frequency of the introgressed haplotype in the 1000 Genomes Project and observed frequencies of 25% in Europeans, 16% in Americans, and 10% in South Asians. Therefore, these introgressed segments exist in present-day humans but the haplotype frequency is not high enough to be

considered adaptive. One explanation could be that the selection strength changed over time. This may explain why these two genes have not been reported as candidates so far. Of course, extending the approach to larger datasets will provide a more complete picture.

Minor comments:

1) To have a full understanding of the data spread, it is good practice to overlay all the data points with jitter on box plots. I would like to see these data points in figures containing box plots.

Answer:
The boxplots now report the data points.

2) On page 5 under "Global Ancestry Inference[...]", there is a typo in the f4-ratio expression. Do you mean f4(Archaic1, Chimp; Test, Africa) / f4(Archaic1, Chimp; Archaic2, Africa)? Furthermore, in the same context, please explicitly define alpha.

Answer:
We thank the reviewer for noticing this typo. The corrected sentence now reads:
"To assess imputation's ability to estimate the overall proportion of archaic introgression in ancient genomes, we applied the f4-ratio approach in the form α = f4(Archaic1, Chimp; Test, Africa) / f4(Archaic2, Chimp; Archaic2, Africa)."

3) For Figures S2 and S8, I recommend plotting the log10 of the sample ages. As most individual samples are from more recent periods, this would allow to more easily visualize how the different variables are affected by sample age.

Answer:
Figure S5 has now the log10 for the individual ages.
While the reasons for suggesting a log scale are perfectly understandable, we believe there are also good reasons not to do it. First is that log scales are better for phenomena with different order of magnitudes, this is not the case here. Even more importantly, for "segment length against age", the older an individual, the more susceptible the segment length is to a variation in age, meaning that if some regions should be looked at with a finer grain it is the oldest ones and not the most recent. If anything, a 1/x scale would make the analysis easier.

4) When mentioning specific individual samples in the text for the first time, please indicate the mean sample age. For example, on page 8, "We observed that Kolyma1 (Siberia, XXX ybp) and USR1 (Alaska, XXX ypb)".

Answer:
We modified the text accordingly.

5) The sentence "While the Archaic Shared segments exhibit the highest mean similarity to the archaic reference genome, the Archaic New segments also show significantly higher similarity compared to regions classified as non-archaic (Figure 2C)" appears to use "significantly" in a qualitative sense, as I did not find any mention of a statistical test. If that is indeed the case, I recommend replacing "significantly" with a more appropriate term such as "considerably" or "substantially."

That being said, based on the visual representation of the similarity distributions, it is difficult to assess whether the non-archaic distribution might have heavy tails or skewness toward high similarity values (although I trust the authors that the distributions are distinct). To support this claim more rigorously, please consider providing summary statistics, such as medians and quantiles, for each category to quantify the differences.

Answer:
We tested the significance of the distributions using a Wilcoxon signed-rank test on both filtered and unfiltered data. In both cases, the distributions are statistically different, and the median difference increases when using the filtered data. The significance is now reported in Table S19).

6) On page 8, "Notably, the Archaic New segments contain more missing positions in the original data." This statement does not seem to be backed up by any figure or quantification. How exactly missingness varies between Archaic New vs. Archaic Shared segments?

Answer:
We thank the reviewer for pointing out that this was unclear. Although this information can be inferred from Table 1, we have now added a supplementary table (Table S3) reporting the summary of the original genotypes. In this table, "Archaic Shared" shows ~50–53% missingness, whereas "Archaic New" shows ~63–69%, depending on coverage. We cite this table in the relevant section to support the statement.

7) On page 8, "We measured the error rate of imputation in archaic SNPs, which is remarkably low at approximately 0.2%[...]", please specify how you calculated the imputation error rate. Typically, for imputation experiments, one tends to focus on error rates at heterozygous sites or non-reference discordance. Please indicate what imputation error rate you found for non-archaic SNPs.

Answer:
As with the previous question, this part of the manuscript is based on the assumptions related to Table 1. Since the reviewer correctly noted a misinterpretation of the term error rate, we now refer to it as "*error of imputation in recalling archaic SNPs*."

8) In Figure 4C, please add a label for the x axis, as well as the start and the end positions.

Answer:
The positions are now shown in the figure for ease of reference.

9) On page 13, the term "gene clusters" is used. Could you clarify what is meant by this? Are these genes physically close to each other in the genome (i.e., clustered by genomic location), or are they grouped based on functional similarity or shared biological pathways?

Answer:
To avoid confusion, we have replaced the term "gene clusters" with "extended regions" throughout the manuscript and supplementary materials.

10) In the Supplementary Information file, page 8, under "Accuracy of archaic alleles vs MAF", you defined a genotype ("between 0 and 2") as GP(1/1) +0.5*GP(0/1). However, this varies between 0 and 1. I suppose that to plot 1-MSE, you are scaling both imputed and the ground truth genotypes to vary between 0 and 1. This can be more explicitly described.

Answer:
The reviewer is right; it should have been written “between 0 and 1” as this is indeed what the formula used outputs and what is used to compute the imputation accuracy.

11) At the end of the same paragraph, it reads: “We then plot 1-MSE, which is equivalent to r^2 correlation.” If you calculate 1-MSE, please write that in the y-axis of your plots and not r^2, the squared Pearson correlation, which I would say are not strictly equivalent.

Answer:
The label has now been modified in Figure 3A.

12) Across the main text, I have occasionally found it difficult to follow the details of the analytical procedures, as some key information appears to be missing or insufficiently explained, but included in the Supplementary Information file.
For example:
- “We kept segments [...] with a length above 40Kb; that is above the length needed to reject incomplete lineage sorting for regions with moderate recombination rates38.” This is an important justification for the filtering threshold. Please consider adding this explanation or at least the relevant reference to the main text.
- “We say that a segment found in the original dataset has also been found in an imputed dataset, if at least 80% of the segment overlaps a segment found with the imputed dataset.” As a reader, I need a clearer explanation of how segment classification and overlap matching are defined and applied in the main text.

On the other hand, I noticed that some parts of the main text explicitly mention standard tools (e.g., admixtools) that might be more appropriate for the Methods section. I suggest relocating such technical details to the Methods section and simply cite the relevant publication in the main text.

Answer:
The “Material and Method” section has been added.

13) My final suggestion is to split Materials and Methods into Materials and Methods and Supplementary Notes (to be referenced in the main text) when appropriate, as there is important discussion of analyses and approaches mixed with methodology descriptions.(Remarks on code availability)

Answer:
We have now added a “Material and Method” section with the most important information about the methods used to obtain the results. The Supplementary section provides additional details including the rationale behind the chosen methodologies and description of secondary findings.

Reviewer #2 (Remarks to the Author):

The authors study the use of imputation on downsampled ancient genomes to assess the application of imputation with the specific aim to identify areas of archaic integressions. I find the work to be thorough and compelling. The choice of methods is sound and the presentation in terms of figures and experiments is highly convincing.

One possible weakness would be the extent to which it is possible to use solely modern references. I have long been an advocate of this for aDNA imputation in general, but there could still be doubts. This especially applies to the (otherwise interesting) analysis of unshared segments in Ust'Ishim. In this context, I would have been curious on a discussion on to what extent it would be possible to augment the reference set with fully archaic genomes (possibly themselves imputed, depending on practical constraints on the nature of a reference). This would not replace the use of a fully modern reference, but the addition of a single or a few highly distinct genomes that would better cover archaic segments heavily diluted or missing in modern genomes would illustrate the relative stability of the results, beyond the comparison already done based on the comparison between imputed and original reads.

Answer:
We understand the reviewer's concerns and agree with the general point. The main limitation of imputing based on archaic genomes as a reference is that it most of the time requires phasing the archaic genomes. This would require testing how well we can phase archaic genomes. We suspect that regions that are introgressed in present-day humans would be easily phased in the sequenced archaic humans, but that's approximately ~30% of the archaic genome. While the reviewer suggests first imputing the archaic genomes (and possibly phasing them), phasing itself would again require present-day haplotype datasets, which introduces redundancy.

Notably, our work shows that even when using only the 1000 Genomes Project as a reference, we recover almost all the archaic segments that are present in the high coverage ancient genomes using the downsampled imputed genomes. In general, adding more diverse individuals to the reference panel should help improve imputation especially in underrepresented ancient populations harboring archaic ancestry.

We do not doubt that approaches such as MetaGlimpse (Kumar et al. 2025), which will incorporate additional (also unphased) references in the imputation panel, will further improve the detection of archaic segments. Indeed, phasing archaic genomes is something we are actively exploring, although it remains technically challenging and requires more time.
Per the reviewer's request, we now added some discussion about the inclusion of archaic genomes to improve imputation in the discussion:
"One future research direction may involve extending hmmix to also leverage the archaic human genomes in the inference step or including the archaic genomes in the imputation reference panel which may improve the recall of genotypes with an archaic allele."

I do have several concerns about the article outline. The abstract is almost 800 words, while Nature Communications seem to indicate a limit of 200 words. The section "Results and discussion" has a somewhat misleading title, which could rather be "Results and Methods". The proper author instructions for Nature Communications do indicate the need for a specific Methods section. The subsection structure could also be enhanced. When reading the

manuscript in full, I was highly convinced, but I found it challenging when I wanted to go back and check some detail to locate where it was actually mentioned. I also think the choice of references is somewhat incosistent.

Answer:
We thank the reviewer for pointing this out. We have reformatted the manuscript by adding a dedicated Methods section to make the technical aspects of the work easier to follow. In addition, in the workflow shown in Figure 1A, we have added numbering to link each step to the corresponding paragraph in the manuscript. Following the Nature Communications guidelines, we removed secondary subheadings, and therefore numbering within subheadings could not be used.

Reviewer #2 (Remarks on code availability):

The code provided only covers a limited part of the total context, creating final summary statistics.

The code is far from enough to reproduce the results in the paper. The running of the imputation process itself (using the pre-existing GLIMPSE tool) isn't covered at all, for example. Some resulting data is probided in CSV form.

Answer:
We have improved the code and data sharing part. We have added the complete code to Zenodo (DOI: 10.5281/zenodo.16365479) together with the data (original and imputed VCFs, introgressed maps, Dstat values, read counts, etc.), and detailed instructions to run the different software and generate all the figures used in the main and supplementary text.
The introgression maps and all future updates are also available in the following GitHub repository: https://github.com/LeoPlanche/arcAncientImputed

Reviewer #3 (Remarks to the Author):

General comments:

The imputation in ancient genomes was shown to work well given a certain MAF > 5%, coverage > 0.5 x and closeness to the reference panel (Sousa da Mota 2023) and is done frequently now. However, it was never assessed how it affects the archaic ancestry in the imputed individuals. A common method to overcome this is to enrich the endogenous amount of DNA by only capturing specific sites in the genome. The manuscript concludes that archaic ancestry can be imputed for shotgun genomes even better compared to random sites and to lower coverage. The manuscript is concerned with this very significant question and there has been a lot of work put into it. It touches on multiple things: testing imputation and subsequent global and local ancestry inference. Where the local inference is the main focus using hmmix. Most of the evaluation seems to be done on Neandertal ancestry (since most genomes are West-Eurasians without Denisovan ancestry). Denisovan ancestry is checked. Then the authors conclude that population structure is preserved after imputation. Finally, the imputed genomes were used for searching for candidates of adaptive introgression, for which two new candidate genes were found. My main comment is that I am not quite sure about the scope of this manuscript and feel the manuscript could benefit from some streamlining. Since a lot of things are touched upon but sometimes not discussed in detail, what is the core aim of the manuscript?

For example:
If the paper is a paper about how to best do imputation for local ancestry inference one would need to check the best reference set for GLIMPSE to do that (especially for Denisovan ancestry, the 1 k Genomes has no populations with high Denisovan ancestry), how ascertainments (i.e. captured data) influences imputation, how it works using different LA callers and what are the best filter parameters. And in the end have a set of recommended parameter settings.

If however the focus is more one power increase for calling candidates of adaptive introgression e.g. only for Neandertal variants, then why not expand the number of individuals since now you can use low coverage and potentially also all the thousands of captured samples.

Or is the aim to find Denisovan ancestry in single ancient individuals which is hard for “ancestry painting” LA callers like admixfrog or CRF since they might be biased to the reference available. So hmmix is the way to go. However it needs high quality data which is not available for most ancient genomes (especially for non-West-Eurasian genomes) so one can potentially impute genomes to refine the detection of Denisovan ancestry.

Also, since a lot of ancient individuals are only captured it would be very interesting and relevant to know how the imputation works on captured data.

Answer:
We thank the reviewer for recognizing the impact and breadth of our work.

The main goal of our research program is to study archaic ancestry through time and to understand how both demographic history and natural selection have shaped the trajectory of archaic variants. As the reviewer notes, there are now thousands of available ancient genomes; however, the vast majority are of low coverage or derived from capture data, which often limits the amount of data available for population genetic analyses. A recently widely adopted approach to overcome this limitation is to impute ancient genomes, thereby maximizing the amount of data available for downstream analysis. To date, no previous study (to our knowledge) has evaluated whether archaic genomic regions can be accurately inferred from imputed low-coverage ancient genomes. Before imputing thousands of genomes, a labor-intensive process, we therefore needed to benchmark the reliability of archaic variant imputation. *The central goal of this study is to demonstrate that imputation of archaic variants is feasible and reliable.*

Our work provides what we believe are novel and unexpected findings about how imputation performs on archaic versus non-archaic variants. We include a series of complementary analyses to illustrate that imputed archaic variants yield robust results and, in some cases, can even enhance resolution when inspecting introgressed regions of interest (e.g. adaptive introgression). Considering the thousands of imputed ancient genomes available and knowing that it works, we can move forward to the evolutionary analysis of archaic variants in a larger data set which we hope to present in a forthcoming study.

To address the reviewer's concerns regarding the scope of the manuscript, we made a few changes to the introduction to make the purpose of this study clear. The scope and organisation are now stated explicitly at the end of the Introduction, and the order of sections and their interconnections are clarified in Figure 1A (workflow).

Specific comments:

Line numbers would have be useful to directly refer to parts in the manuscript.

Answer:
Line numbers have been now added

GILMPSE provides phased haplotypes, do you use this information and do you think it works, since having phased archaic haplotypes makes distance measures and TMRCA estimation easier in downstream analysis?

Answer:
We didn't use phased haplotypes in the downstream analyses, with the exception of Figures 4C and S38 when we look at specific candidate regions. Previous studies suggest that imputation improves phasing accuracy (Medina-Tretmanis et al. 2024), so we expect that phasing after imputation should work well. Furthermore, because introgressed segments introduce structure in their genomic locations (increasing divergence between the archaic and non-archaic haplotypes), we expect that phasing at archaic regions will also be easier than in regions with no introgressed segments.

Using the 1k Genomes sites I assume these are sites where humans are polymorphic, that also means you never look at sites where present day modern humans have fixed differences to the archaics. Do you keep this ascertainment for all analysis? You could imagine that ancient individuals do carry fixed different sites between archaics and present day humans.

Answer:

The reviewer is correct that we do not account for fixed differences between the 1000 Genomes Project populations and the archaic genomes. We do keep this ascertainment throughout. We think it is important to consider fixed differences to obtain accurate estimates of divergence. For these types of estimates, sites where modern humans are fixed for one allele and archaic humans are fixed for another allele (e.g. fixed differences), the user can always add them in later (after imputation) to consider sites with fixed differences between the ancient individual and the archaic humans.

Concerning captured data, which is most of the ancient data. Some of these capture arrays are not specifically designed to capture archaic ancestry (like the widely used 1240k and twist capture), it would be very interesting if one can successfully impute the archaic ancestry there (e.g. filter the test ancient genomes to sites overlapping the capture array similar to Sousa de Mota 2023).

Answer:

To address the reviewer's comment, we focus on one individual (LBK/Stuggart) in our dataset who was captured (using the 1240K capture array, coverage 8X) and shotgun sequenced (coverage of 14X). To assess performance, we downsampled the 1240K capture data for LBK to the same coverage (2X, 1X, 0.5X and 0.0625X) used in our study and compared them with the original high-coverage shotgun data. We then generated plots analogous to Figures 2A and 2B, directly comparing capture and shotgun data (Figure S7-S8). Our results show that, with sufficient coverage (>=2X), the imputed capture data can recover more archaic segments than the shotgun original data, thereby confirming the value of the imputation approach we propose in this work. However, for coverages less than 2X, imputing the capture data does not help recover the introgressed segments identified in the original high coverage whole genome. Therefore, imputation of the low coverage whole-genome data performs better than imputing the low coverage capture data. Since most capture data is low-coverage, it might not be the best data to consider the evolution of archaic ancestry through time. It might be due to the limited number of archaic sites in the 1240K panel mentioned by Iasi et al. (2024).

Fig. 1 What are the boxplots showing? All individuals and their D or alpha? A supplement figure showing the values (y axis) per individual (x-axis) for the original and imputed estimates, for each downsampled (facet) might be informative. Why has Mota a very high D in the original data (which is very significant as Fig S1 shows). Why does it vanish when using imputed OC? Maybe check the setup of the D stats, it could be that Mota has very deep relations to the other Africans which might introduce the imbalance.

Answer:

The individual plots are now included in Figure S2, and the data used to generate them are provided in Table S10. To address the significant D-statistics observed for Mota in the original data, we restricted the analysis to transversions only and obtained a negative Z-score (Table S10). This suggests that the previously observed values in Figures 1 and S1 were likely driven by typical ancient DNA misincorporations due to deamination. Furthermore, this result supports the conclusion that imputation can also help correct some of the classical errors associated with ancient DNA.

One point of concern for me with imputation would be false positives, one would really not want to impute archaic ancestry segments into individuals which are not there. For example

in the African individual Mota, which probably only has very few if any Neandertal segments, after imputation it has many more (Fig 2A), but is that not your negative control (i.e. where you don't 'want' to see archaic ancestry imputed)? You use the original data as ground truth and apply a cutoff per genotype of 10 reads or more, which will lose you a lot of data especially for the individuals close to 10 x (e.g. Mota). I guess this is one reason why you consistently impute more archaic ancestry in imputed OC genomes. Even after filtering it is usually twice as much and for your 'negative control' it is more than 10 times as much. To gauge if it is a false positive, you could look at the reads covering sites where you impute archaic variants at sites with missing genotypes in the original (since for a lot of them you should have reads covering them but no genotypes). How often do the reads support the imputed variant and how often not?

Answer:
It is true that when using the original high-coverage data to obtain high-quality genotypes, we applied a stringent cutoff (see Methods) which will affect the genomes with lower coverage (~10X). This tends to decrease the number of SNPs available and as a consequence we have a reduction of the segments that we can identify, and the amount of that is proportional to the original coverage (Figure S6).

We thank the reviewer for their suggestion, following their comment we also analyzed the underlying reads. We analysed all the missing positions in the Original VCF that are imputed in the Archaic New segments. We observed that most of these positions have reads that support the alternative allele (Figure S23, in Mota ~94%), suggesting that the imputation worked. When we ask, what is the proportion of missing sites with low read support per Archaic New segment, we find that it is quite small (<1%, see Figure S24) most of the time. Even if these sites were imputed incorrectly, their occurrence is so rare on Archaic New segments, that it will not significantly influence the inference of the introgressed haplotypes that harbor many variant sites. We do acknowledge that the sequencing coverage will affect the number of imputable sites (e.g., we can impute more sites with a 1X coverage genome than a 0.0625X coverage genome), but the imputation accuracy is very high across coverages (Figure 4C). We add multiple evidence in a new paragraph in the Supplementary Material named "BAM-derived support for imputed variants in Archaic New segments."

Regarding Mota, the reviewer raises an important point. Mota represents an extreme case for the identification of archaic introgression, particularly when using an archaic-reference-free approach such as hmmix, in which introgressed regions are inferred from windows enriched in SNPs that are absent from present-day African populations.
In this context, an African individual such as Mota can contain genomic regions with a high number of variant sites where the allele is not present in the African populations used as the outgroup in hmmix. After inspecting these regions, we find that indeed, Mota contains regions where they have many heterozygous sites suggesting that they harbor a haplotype not present in the African populations considered as outgroup for this study. The reason these positions pass our filters is because Mota is heterozygous which will always lead to a match with Neanderthals when computing the pairwise similarity, even though these regions do not correspond to haplotypes present in known archaic genomes. Indeed, when we examine in detected segments the number of private alleles matches (instead of pairwise similarity) with the Vindija Neanderthal or the Denisova genome (Figure R3.1), these regions show a lower proportion of shared alleles in Mota compared to other individuals, although in some cases they still display a relatively high number of matches.

Importantly, these patterns are consistent when comparing results obtained using the Original genotypes and the Imputed genotypes. This indicates that the observed signal is not driven by imputation artefacts but rather reflects the intrinsic difficulty of identifying introgressed regions in extreme cases such as Mota.

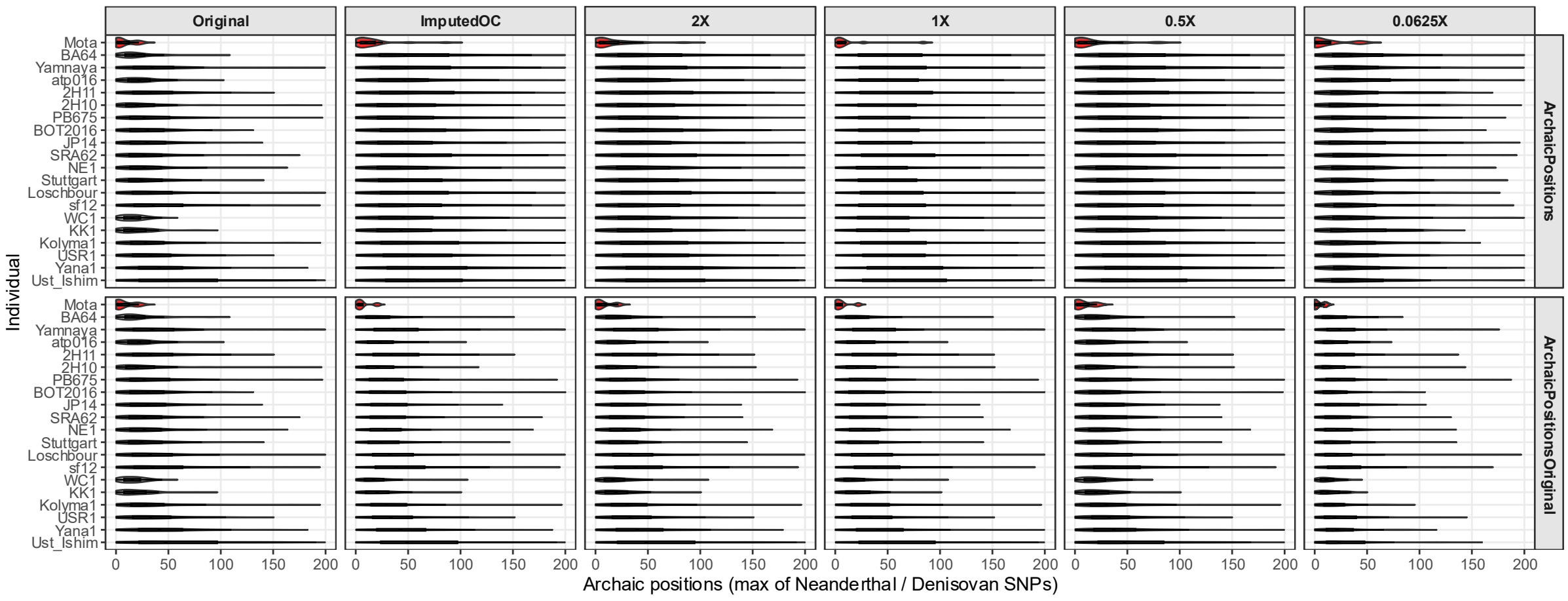

Figure R3.1. *For each coverage, the violin plots of the distributions of the number of archaic-supporting SNPs per fragment identified in each individual. This number is defined as the maximum number of Neanderthal or Denisovan SNPs within each segment, either using the imputed genotypes (ArchaicPositions row) or the original genotypes (ArchaicPositionsOriginal). The African individual Mota is highlighted in red, while all other individuals are shown in grey.*

Why do the authors not use a recombination map to base the filtering on recombination distances? The filter is 40 kb (or 100 kb for Denisovan segments), but they could be in low recombination regions.

Answer:
We applied a length filter to minimize incomplete lineage sorting (ILS), following prior work that used bp thresholds. Specifically, we set 40 kb (as in Racimo et al. 2017) because Prüfer et al. 2014 reported a mean introgressed haplotype length of 44,078 bp, and 40 kb comfortably exceeds the length required to reject ILS under moderate recombination rates (Huerta-Sánchez et al. 2014).
We agree that using centimorgans (cM) could be more rigorous. Accordingly, we re-evaluated Figure 2 with recombination maps and cM distances. The resulting estimates (Figure R3.2) show no significant differences in total introgression when using segment length in bp versus cM. The length in cM for each segment has been added to the introgression maps.

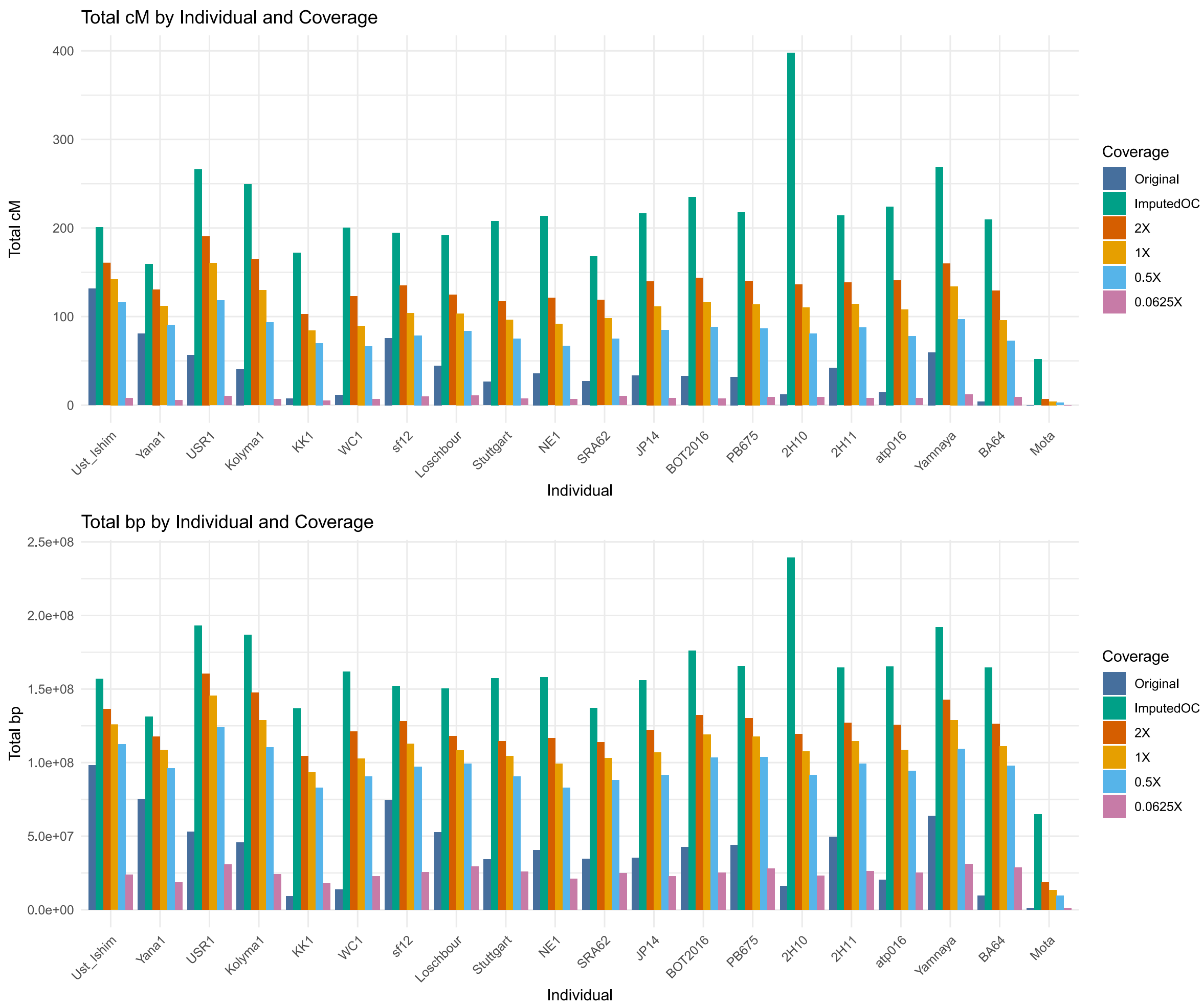

Figure R3.2. *Comparison of the sum of the length of the identified archaic segments as centimorgans (Top) and base pairs (bottom).*

Fig S5. It seems after imputation your non-archaic segments consistently all shift to be closer to the archaic genome. Why would that be? If you are better in calling archaic ancestry after imputation would you not assume that the non-archaic segments would be even more distant to the archaic since you 'filtered' them from misassigned segments? Do you introduce variants which are closer to the archaic or do you correct variants in the original. Table 1 does not show signs for either (e.i." . -> 0" is big and ". -> 1" small).

Answer:
The point raised by the reviewer is valid. For segments that are not classified as archaic, we observe a shift toward higher similarity to the archaic reference genomes when using imputed data. In addition to the well-known correction of sequencing errors caused by ancient DNA deamination, imputed data also contains more positions, and the GP 99 filter can bias which type of positions (archaic/nonarchaic, heterozygous/homozygous etc.) are kept and removed, as we have already shown with the *D-statistic* value increase for low coverage imputed data. How does this exactly influence the pairwise similarity between imputed data and archaic genome is still to be further studied.

We therefore checked whether this effect could introduce any bias on the LAI task and filtering schemes. We repeated the same analysis using only filtered segments and calculated similarity and distance based on the Original genotypes (Figure R3.3) (see "Similarity and

distance to Archaic genomes" section for the definition of these scores). This plot confirms what is shown in Figure 2C: a clear separation between archaic and non-archaic segments is still observed. Even with the colour scale displayed on a logarithmic scale, the two classes remain well separated.

These results indicate that our filtering strategy is robust and that archaic and non-archaic segments can be reliably distinguished.

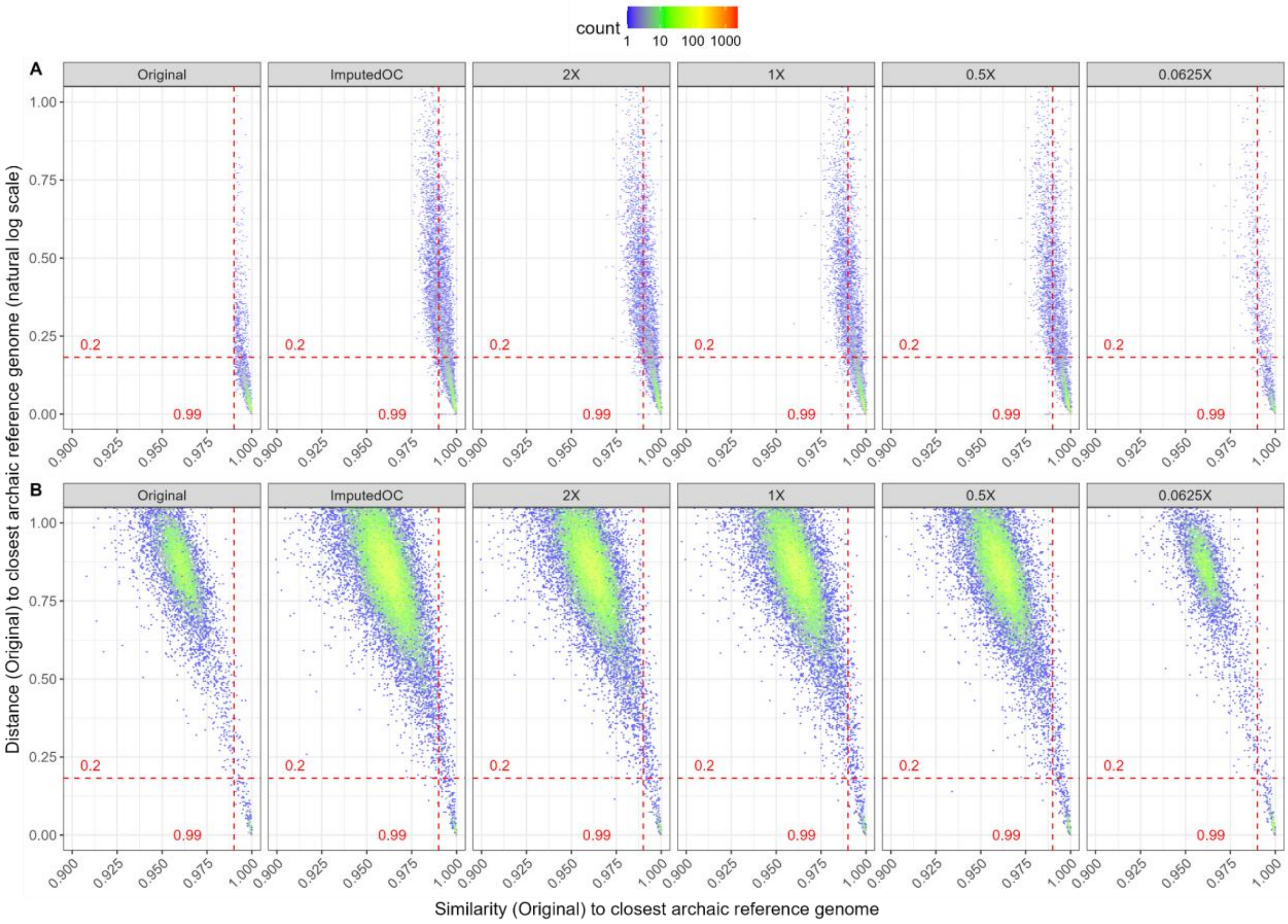

Figure R3.3. *Density plots showing the relationship between similarity to the closest archaic reference genome (x-axis) and distance to the closest archaic reference genome (log-transformed, y-axis) for (A) archaic segments and (B) non-archaic segments. Each hexagon's colour reflects the log-scaled number of segments per bin. The vertical dashed red line marks the 0.99 similarity threshold. Only filtered segments longer than 40 kb were retained, and archaic segments were further restricted to those with similarity > 0.99 based on the imputed genotypes.*

Fig3 B. I would also plot the specificity of the heterozygous calls.

Answer:

To standardize some baseline analyses, we used widely adopted software. For heterozygous sensitivity (TP / [TP + FN]), we employed Picard's GenotypeConcordance. This tool does not report in the summary results the specificity stratified by genotype class (i.e., TN / [FP + TN] per homozygous reference/heterozygous/homozygous alternative); it provides only an overall specificity. The resulting plot (Figure R3.4) shows higher specificity in archaic segments than in non-archaic segments. Moreover, we examined the BAM reads for the Archaic New segments and confirmed that all imputed alternative alleles used to identify these segments

are supported by the sequencing reads. Please see the section titled “BAM-derived support for imputed variants in Archaic New segments” in Supplementary material.

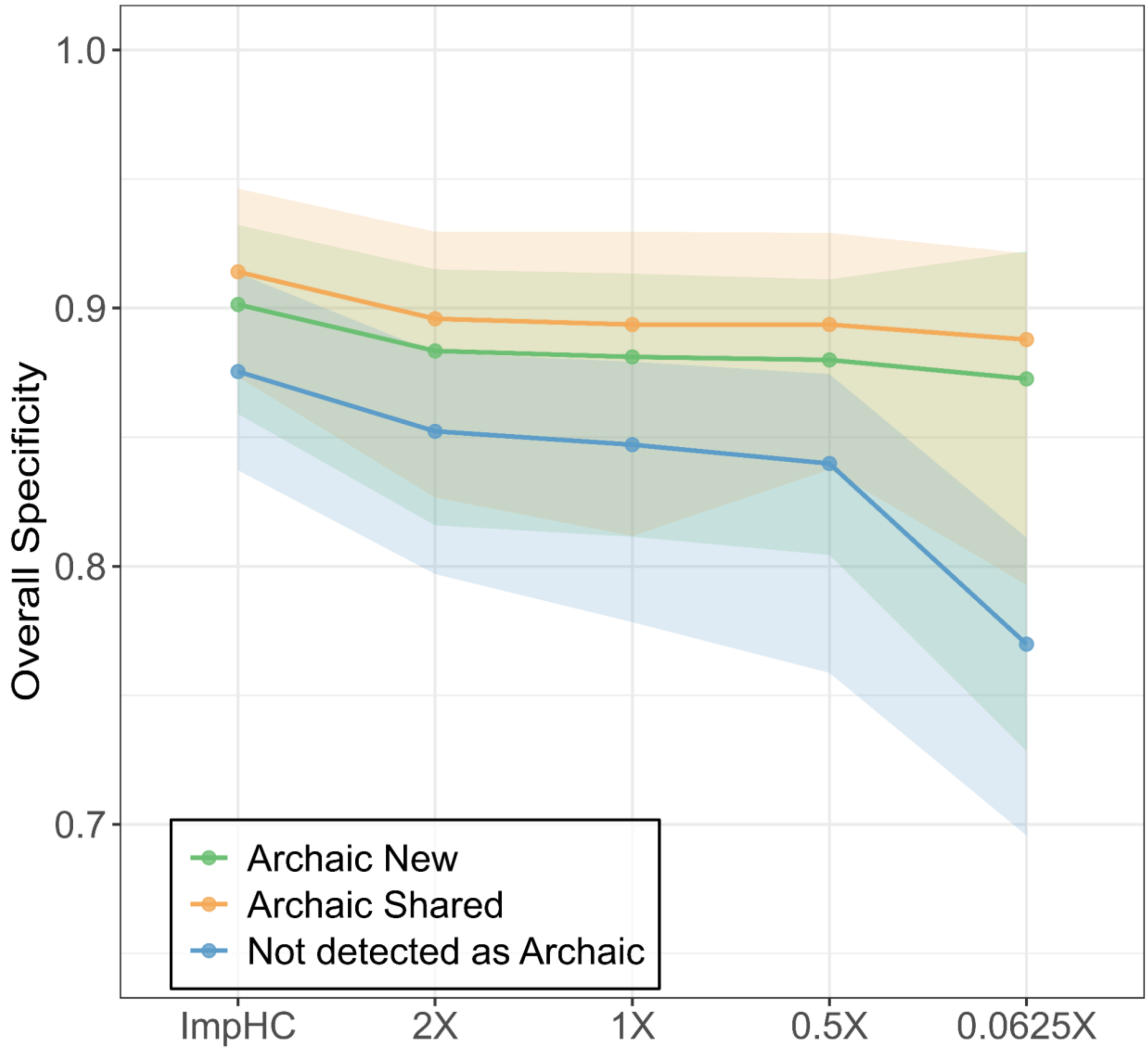

Figure R3.4. *Overall specificity (y-axis) for different downsampled coverages (x-axis).*

Denisovan ancestry: You do not find any Denisovan segment in Ust’Ishim in the original data. After imputation you do find 4 segments with one matching the hmmix map and one to the ArchaicSeeker map. At 1x you find 6 segments 2 matching hmmix map 4 matching the other. Are these additional 2 segments, compared to imputed OC? Or did the 4 OC segments break into 6 shorter ones?

Answer: All six segments identified in the 1X coverage are also initially detected in the Imputed OC dataset. The reason there is a difference in the final number of Denisovan segments called between 1X and the Imputed OC is due to whether individual segments pass or fail the filtering thresholds used to classify Denisovan introgression.

If the 1x really has additional non overlapping segments compared to the OC imputed that would be worrisome, since then the imputation would have introduced Denisovan segments where there are none. Do you think the segments in Ust’Ishim are true? You find evidence

that Ust'Ishim predates the split between Europeans and East-Asians (in concordance with earlier papers), so if you find Denisovan segments in Ust'Ishim they would predate the split. You also find that the segments are shared with present-day populations. That would suggest that the Denisovan admixture happened before Ust'Ishim lived. I think it would be nice if the authors could interpret the results a bit more. Do you think these are genuine Denisovan introgressed segments or false positives from either the imputation or hmmix?
In short, how confident are you to reliably call Denisovan segments?

Answer:
We thank the reviewer for requesting a more detailed investigation of these segments. We inspected all inferred Denisovan segments (8 in total across all coverages) that passed the filters (length > 100 000, Distance to Denisova < 0.2 and Distance to Neanderthal - Distance to Denisova > 0.3). We first checked the imputation by checking the genotypes inferred in the downsampled individuals to the Original Ust'Ishim genome. As shown in Figure R3.5 (below), in these eight regions, we find that the genotypes inferred in the downsampled genomes match the genotypes in Original Ust'Ishim. In particular, imputation correctly calls the heterozygous sites, with the exception of the lowest coverage genome (0.0625X) where there are some missing genotypes. These results indicate that imputation performs well in these regions for most coverages (> 0.0625X).

To infer introgressed segments, *hmmix* leverages variants that are absent in African populations and are present in non-African populations. These 8 segments are highly divergent from African genomes and are therefore classified as introgressed by *hmmix*, in accordance with the underlying logic of the method. After hmmix, we classify segments into Denisovan or Neanderthal by using a set of filters which includes a similarity measure. The similarity metric used to compare inferred segments with archaic reference genomes treats heterozygous sites as allele matches. What is clear from the figure, is that Ust'Ishim harbors two divergent haplotypes given the number of heterozygous sites present in this individual which sometimes inflates the number of "matches" with the Neanderthal or Denisovan genomes even when Neanderthals and Denisovans do not harbor the divergent haplotype present in Ust'Ishim.

Regarding the reviewer's question of whether these segments are truly of Denisovan origin, we observe that in six of these eight regions, at the positions showing divergence from African genomes, Ust'Ishim does not share private archaic alleles with any of the previously published high-coverage archaic genomes and instead appears highly divergent from all available archaic references. However, one region on chromosome 15, Ust'Ishim shares a larger portion of the haplotype with Vindija, more so than with Denisova. In addition, thanks to our colleague Dr. Stéphane Peyrégne, who kindly shared unpublished data from a newly sequenced high-coverage Denisovan genome (Denisova 25)(Peyrégne et al. 2025), we were able to extend this comparison to this additional archaic individual. Notably, in the region on chromosome 10, Ust'Ishim shares more private archaic alleles with Denisova 25 than with Denisova 03 or with any of the Neanderthals.

In conclusion, our inspection of these 8 segments indicates that imputation performs well in these regions and that *hmmix* correctly identifies segments that are not present in African genomes. However, we cannot confidently assign these segments to a Denisovan origin. Clearly, Ust'Ishim harbors a divergent haplotype (in 6 of these regions) not present in African or archaic genomes, and this could simply represent a private haplotype that was lost in

African populations or possibly introgression from an as-yet unknown archaic hominin. Consequently, depending on the scope of the analysis and the aims of a given study, different filtering strategies may be more appropriate to classify and retain the subset of segments most relevant to the specific research question.

These results also show that the filters we previously used to define Denisovan segments, although stringent, were not sufficient to avoid misclassification of the source population as in Ust'Ishim. For this reason, we revised our methodology and adopted what we believe is a more robust approach to classifying introgressed segments as Denisovan or Neanderthal. For each segment in the introgression map, we now include the number of private archaic variants for each of the four published high-coverage archaic genomes. A private archaic variant is defined as a variant present in the ancient individual, absent in the African outgroup, and present in the archaic genome.
We now classify a segment as Denisovan using a slightly different set of criteria, more in line with the literature: a length greater than 100 kb (as before), a similarity to the archaic genome above 0.99 (as before and as required for any archaic segment), at least 10 archaic (present in Denisovan or Neanderthal) private variants (a filter used in hmmix(Skov et al. 2018)), and a greater number of Denisovan than Neanderthal private variants. This filtering scheme is closer to what is commonly applied in the literature(Skov et al. 2018). We have updated the manuscript accordingly, and the revised results are shown in Figure 2D and Figure S12. Using this approach, the number of Denisovan segments in Ust'Ishim is zero across all coverages, whereas Siberian individuals such as Kolyma1 and USR1 harbor the highest number of Denisovan segments, accounting for approximately 0.1% of their genomes. Notably, this pattern remains stable down to 0.5X coverage, indicating that Denisovan introgression can be reliably detected even in low-coverage ancient genomes.

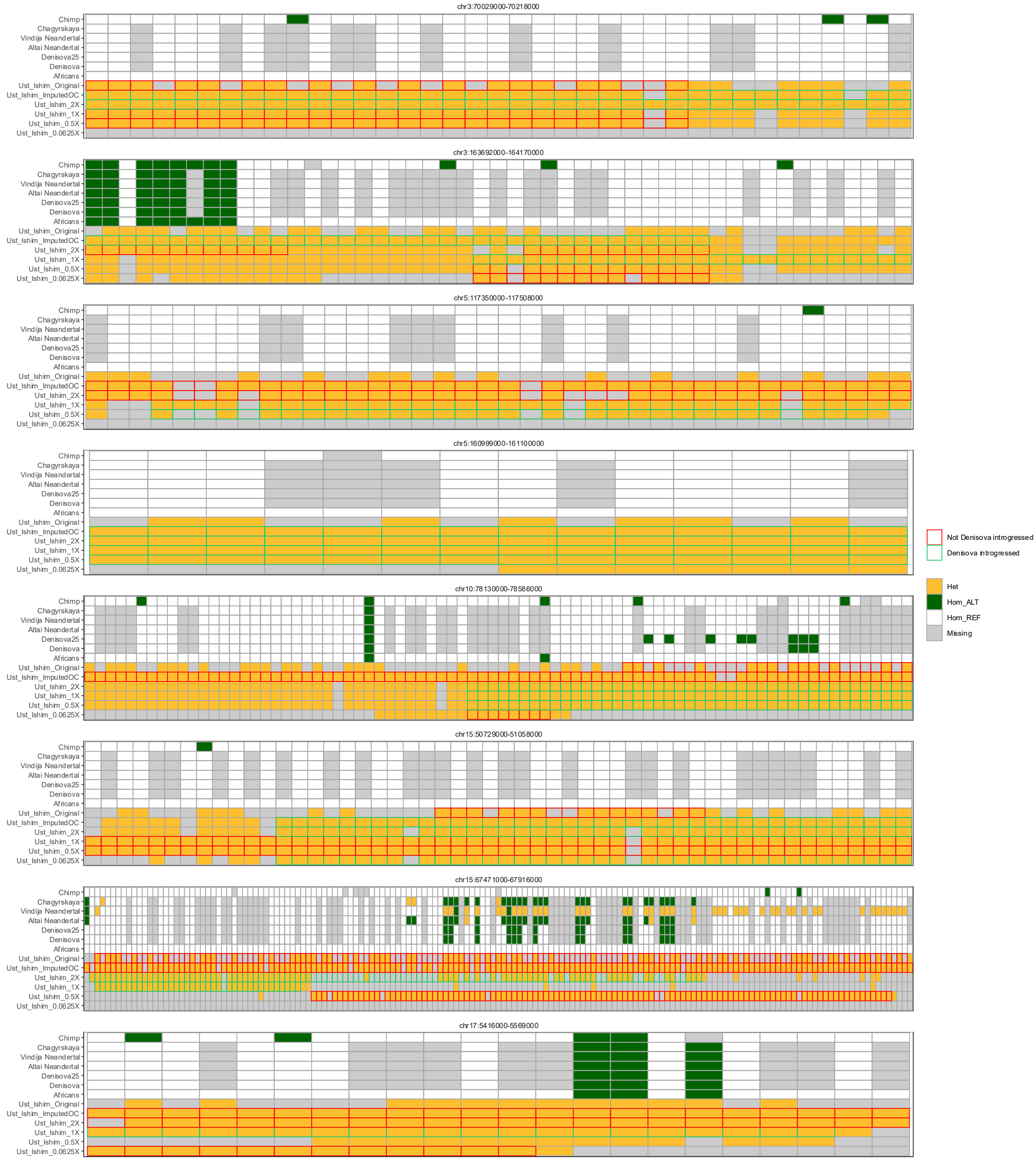

Figure R3.5. *Heatmaps showing genotype states at polymorphic sites within inferred introgressed genomic segments across multiple coverage levels of the Ust'-Ishim genome. SNP positions were selected based on differences between the collapsed African genotypes and at least one imputed Ust'-Ishim genome. Each column represents a single SNP, ordered by genomic position within each segment, and each row corresponds to an individual or dataset, including the collapsed African genotypes, multiple coverage levels of Ust'-Ishim (Original, ImputedOC, 2X, 1X, 0.5X, 0.0625X), high-coverage archaic genomes (Altai Neanderthal, Vindija Neanderthal, Chagyrskaya Neanderthal, Denisovan, and Denisova 25), and the chimpanzee outgroup.*
Genotype states are colour-coded as homozygous reference (white), heterozygous (yellow), homozygous alternate (dark green), and missing data (grey). Facets correspond to distinct inferred introgressed regions, labelled by chromosome and genomic coordinates.

Outlined cells indicate SNPs falling within inferred introgressed segments in Ust'-Ishim. Green outlines denote segments that pass the Denisovan affinity filter, whereas red outlines indicate segments that do not. For archaic genomes, blue outlines indicate SNPs located within callable regions defined by genome-specific masking filters, as reported in the original publications.
Note that we are interested in archaic private archaic alleles shared between Ust-Ishim and one of the archaic individual, i.e. columns where the archaic line and the African line differ while the archaic line and Ust-Ishim have a match (ie both individuals are homozygous for the same allele version, or one individual is homozygous and the other is heterozygous).

The manuscript says: "AKAP1329 is significant in the original data, but not in the imputed data. Specifically, AKAP13 is present in 7 out of 8 Neolithic Europeans but only in 1 out of 3 Mesolithic individuals, failing to meet the minimum threshold for significance in imputed datasets." So what changed in comparison to the original data? Did you 'lose' segment in the Mesolithic individuals?

Answer:
For each coverage, we applied a Bonferroni-corrected significance threshold. Because the number of tested segments determines the multiple-testing burden, a larger number of segments yields a more stringent threshold; consequently, more individuals (i.e., more segment recurrences) are required for a segment to reach significance. As shown in Figure 2B, filtering inevitably removes some true positives. In this specific locus, the segments for individuals KK1, atp016, and 2H10 were filtered out due to sequence similarity ≈ 0.986, which is below our retention cutoff of 0.99. We intentionally set this cutoff conservatively to minimize false positives. In the figure below, identified segments overlapping the gene region are colored by similarity: pink (< 0.99) indicates segments removed by the filter, and blue (≥ 0.99) indicates segments retained.

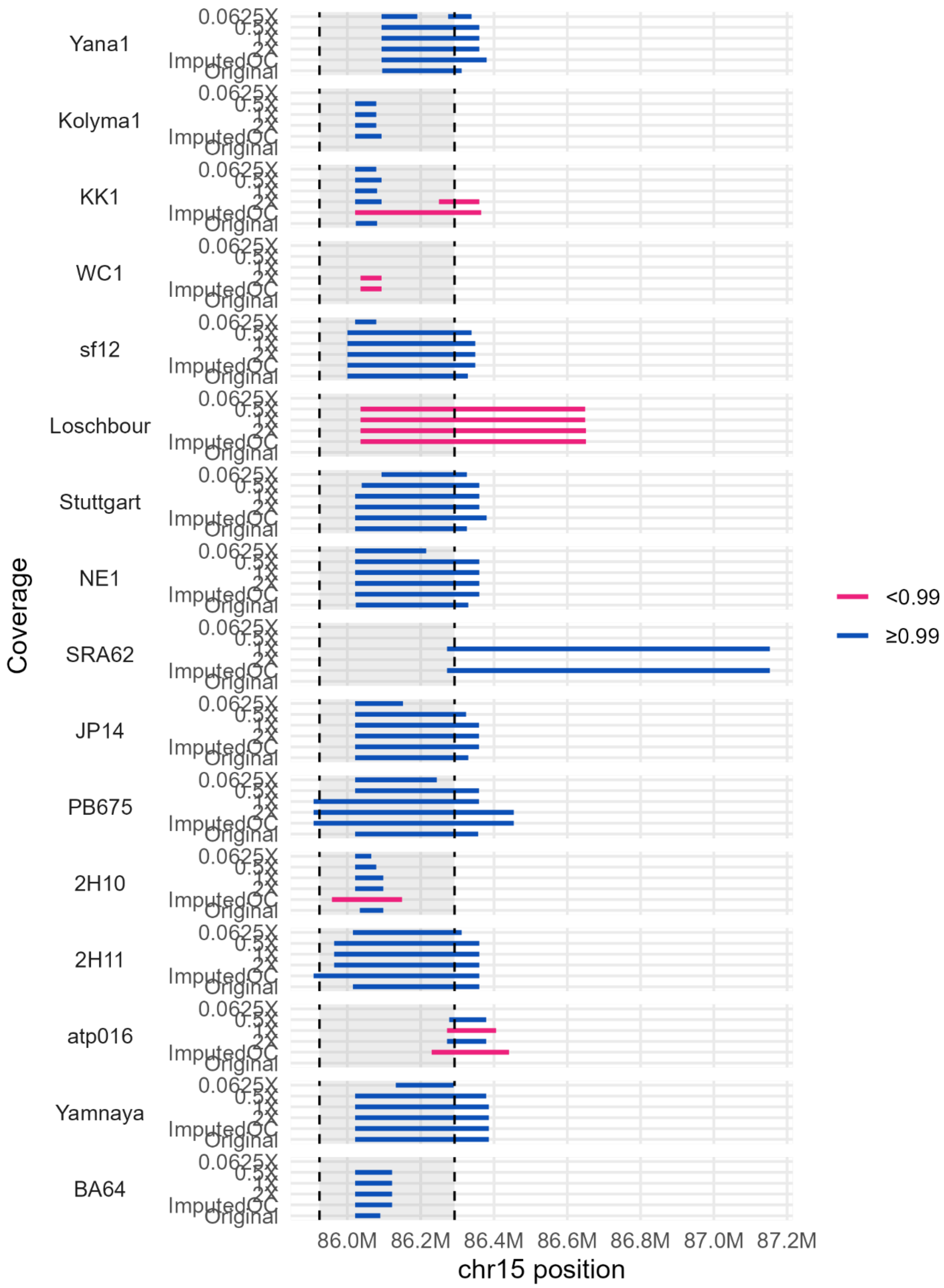

Figure R3.6. *Archaic introgressed segments overlapping the AKAP13 locus across coverage levels and individuals. Horizontal bars indicate archaic introgressed segments overlapping the AKAP13 gene region (chr15: 85.9–86.3 Mb) identified in each individual across the Original, ImputedOC, and downsampled coverage datasets (2×, 1×, 0.5×, 0.0625×). Rows correspond to individuals (facets), and within each individual segments are shown separately for each coverage level. Segment colour denotes similarity to the closest archaic genome, with blue indicating high similarity (≥0.99), corresponding to segments that pass the filtering criteria, and red indicating lower similarity (<0.99), corresponding to segments that do not pass the filter.*

The shaded region marks the genomic span of AKAP13, and dashed vertical lines indicate gene boundaries.

You identified LMED2 and MLN as promising candidates for adaptive introgression. Are they also in high frequency in present-day Europeans? If yes, why did no selection scan in present-day Europeans find them? Maybe you can provide more information on these genes, what are they doing?

Answer:
More information about these two genes is now available in the Supplementary Material.
"Using individuals from the 1000 Genomes Project, this haplotype occurs at relatively high frequency in Europeans (~25%) and populations from the Americas (~15%) and is less frequent in other geographic regions (Africa ~2%, confined to admixed individuals; East Asia ~3%; South Asia ~9%).
LEMD2 (LEM domain nuclear envelope protein 2). Encodes an inner nuclear membrane protein with a LEM domain (LAP2–Emerin–MAN1 family). LEMD2 interacts with chromatin and lamins and is implicated in nuclear envelope integrity, chromatin organization, gene regulation, and signaling. Variants have been associated with cardiomyopathy, cataracts, and other laminopathies.
MLN (motilin). Encodes the motilin peptide hormone, secreted by the small intestine, which regulates gastrointestinal motility, including rhythmic contractions and gastric emptying. It is produced as prepro-motilin and processed to the mature hormone. Alterations in motilin signaling are mainly linked to gastrointestinal motility disorders. Motilin release during the fasted, interdigestive state triggers gastric phase III of the migrating motor complex, which is associated with the "return of hunger" after a meal. Administration of motilin agonists (e.g. erythromycin) increases hunger ratings in experimental settings.
Further analyses including a larger number of ancient individuals and the application of explicit selection tests are needed to determine whether this region was truly under selection in the past. Such analyses would also help to reconstruct the haplotype trajectory and to understand the selective advantage that may have driven its increase before the Last Glacial Maximum and the Neolithic period, followed by its decline to the frequencies observed in present-day individuals."

References

Barros Damgaard, Peter de, Rui Martiniano, Jack Kamm, et al. 2018. “The First Horse Herders and the Impact of Early Bronze Age Steppe Expansions into Asia.” *Science (New York, N.Y.)* 360 (6396). https://doi.org/10.1126/science.aar7711.

Huerta-Sánchez, Emilia, Xin Jin, Asan, et al. 2014. “Altitude Adaptation in Tibetans Caused by Introgression of Denisovan-like DNA.” *Nature* 512 (7513): 194–197.

Kimura, M. 1955. “SOLUTION OF A PROCESS OF RANDOM GENETIC DRIFT WITH A CONTINUOUS MODEL.” *Proceedings of the National Academy of Sciences of the United States of America* 41 (3): 144–150.

Kumar, Kiran H., Simone Rubinacci, and Sebastian Zöllner. 2025. “MetaGLIMPSE: Meta Imputation of Low Coverage Sequencing Data for Modern and Ancient Genomes.” In *bioRxivorg*. September 1. https://doi.org/10.1101/2025.06.24.660721.

Peyrégne, Stéphane, Diyendo Massilani, Yaniv Swiel, et al. 2025. “A High-Coverage Genome from a 200,000-Year-Old Denisovan.” In *bioRxiv*. October 20. https://doi.org/10.1101/2025.10.20.683404.

Prüfer, Kay, Fernando Racimo, Nick Patterson, et al. 2014. “The Complete Genome Sequence of a Neanderthal from the Altai Mountains.” *Nature* 505 (7481): 43–49.

Racimo, Fernando, Davide Marnetto, and Emilia Huerta-Sánchez. 2017. “Signatures of Archaic Adaptive Introgression in Present-Day Human Populations.” *Molecular Biology and Evolution* 34 (2): 296–317.

Racimo, Fernando, Sriram Sankararaman, Rasmus Nielsen, and Emilia Huerta-Sánchez. 2015. “Evidence for Archaic Adaptive Introgression in Humans.” *Nature Reviews. Genetics* 16 (6): 359–371.

Sankararaman, Sriram, Nick Patterson, Heng Li, Svante Pääbo, and David Reich. 2012. “The Date of Interbreeding between Neandertals and Modern Humans.” *PLoS Genetics* 8 (10): e1002947.

Skov, Laurits, Ruoyun Hui, Vladimir Shchur, et al. 2018. “Detecting Archaic Introgression Using an Unadmixed Outgroup.” *PLoS Genetics* 14 (9): e1007641.

Witt, Kelsey E., Alyssa Funk, Valeria Añorve-Garibay, Lesly Lopez Fang, and Emilia Huerta-Sánchez. 2023. “The Impact of Modern Admixture on Archaic Human Ancestry in Human Populations.” *Genome Biology and Evolution* 15 (5). https://doi.org/10.1093/gbe/evad066.

REVIEWERS' COMMENTS

We thank the reviewers for their comments on the code sharing that improved data and code sharing repository. Please find point-by-point responses (in blue) to each of the reviewers' comments.

Reviewer #1

Reviewer #1(Remarks to the Author)

The authors addressed my comments and concerns and I recommend the publication of this study, provided that the authors fix some broken links (see below) and provide more information on how to use their scripts (README page on github).
I do still think that the article is longer than it should be, and, particularly, the discussion can be shortened; it reads a bit repetitive when taking the results' section into account.

Answer:
We thank the reviewer for the good comments about the manuscript. We acknowledge that the discussion is somewhat lengthy; however, we decided to revisit the analyses in order to provide a final synthesis and to better organize the information presented in the paper.

I found some github links that did not lead to the right pages (main text): Page 22, line 38 and Page 23, line 57.

Answer:
We thank the reviewer for spotting these problems, which have now been solved.

Reviewer #1 (Remarks on code availability):

I did not review the code on github, but I tried to assess how straightforward would be to run the scripts. Particularly for Local archaic ancestry inference instructions on the README file (github), I missed instructions of how to prepare the input files: what are and should contain the files *name_vcf_archaic*.vcf.gz and *name_vcf_sample*.vcf.gz? Should all chromosomes be included into a single vcf? Why does it matter to specify a name for the dataset? Do the input files have to be vcfs, meaning not bcf? What do the different steps enumerated in the first code box do?

These pieces of information are essential for anyone trying to use these scripts for their work, and the authors should provide them. My suggestion is to have a small example dataset and the commands the authors would use for that example, but I do not think that is essential, as long as more information is provided in the first place.

Answer:

We have fixed the issues and improved the instructions in the readme. To verify clarity, we asked colleagues to run the script on a different dataset following only the written instructions. They succeeded, suggesting that the current version is sufficiently clear and functional.

Reviewer #2

Reviewer #2 (Remarks to the Author):

I highly appreciate the authors integration of the combined comments from the reviewers. I think the text is now easier to follow.

Answer:
We thank the reviewer for the good comments about our manuscript.

Reviewer #2 (Remarks on code availability):

Scripts for managing the data, including notebooks for reproducing some figures.

Reviewer #3

Reviewer #3 (Remarks to the Author):

I appreciate that the authors have addressed the comments raised in the previous round and the substantial effort that has gone into improving the manuscript. The analyses based on the 1240k capture data are exciting and have the potential to enable to use many more genomes to be analyzed in terms of LAI calling in future work. The more stringent filtering strategy (e.g., using privately shared SNPs), particularly for Denisovan segments, appears appropriate, and the additional analyses support the robustness of the reported calls.

As a general suggestion, it may nevertheless be helpful for the authors to more explicitly highlight in the Discussion that careful filtering is important when working with imputed data and newly identified archaic segments. The additional segments identified here tend to be shorter and somewhat less similar to the archaic references than those present in the original genome; for example, without filtering, the ratio between the proportion of genome inferred as archaic from segment calls and estimates based on f4 statistics increases substantially, whereas comparable levels are recovered after filtering (as noted in the Results). Adding one or two sentences in the Discussion providing such guidance on best practices would be

particularly valuable for readers who may apply similar approaches in future studies and will strengthen the manuscript as a useful reference for the community.

Answer:
We thank the reviewer for the appreciation of the manuscript.
We have now added a section in the discussion addressing the importance of filtering:
"*We also note that inferring introgressed segments from imputed data sometimes produces false positives, and it is important to filter carefully as we have done here. In particular, we applied stringent filters based on segment length, similarity to the archaic genomes and the number of archaic alleles present in the introgressed segment. This approach removed a large proportion of false positives, though more specific filters can be applied depending on the scope of the analysis*."

Overall, I am satisfied with the current state of the manuscript.
